# Simultaneous Stereoinvertive and Stereoselective C(sp^3^)–C(sp^3^) Cross-Coupling of Boronic Esters and Allylic Carbonates

**DOI:** 10.1021/jacs.4c03686

**Published:** 2024-05-09

**Authors:** Hong-Cheng Shen, Ze-Shu Wang, Adam Noble, Varinder K. Aggarwal

**Affiliations:** School of Chemistry, University of Bristol, Cantock’s Close, Bristol BS8 1TS, U.K.

## Abstract

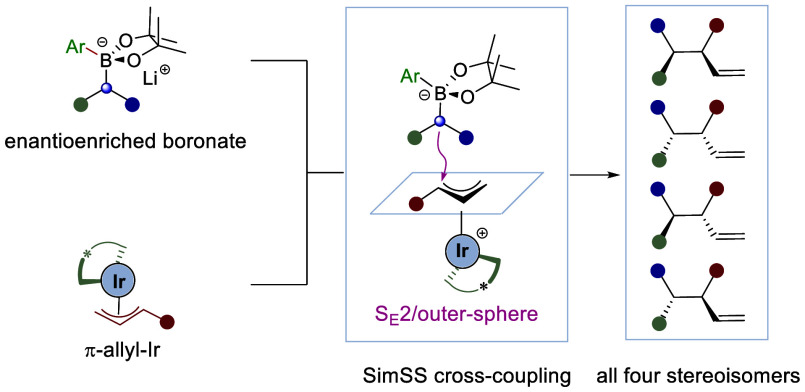

With increasing interest in constructing more three-dimensional entities, there has been growing interest in cross-coupling reactions that forge C(sp^3^)–C(sp^3^) bonds, which leads to additional challenges as it is not just a more difficult bond to construct but issues of stereocontrol also arise. Herein, we report the stereocontrolled cross-coupling of enantioenriched boronic esters with racemic allylic carbonates enabled by iridium catalysis, leading to the formation of C(sp^3^)–C(sp^3^) bonds with single or vicinal stereogenic centers. The method shows broad substrate scope, enabling primary, secondary, and even tertiary boronic esters to be employed, and can be used to prepare any of the four possible stereoisomers of a coupled product with vicinal chiral centers. The new method, which combines the simultaneous enantiospecific reaction of a chiral nucleophile with the enantioselective reaction of a chiral electrophile in a single process, offers a solution for stereodivergent cross-coupling of two C(sp^3^) fragments.

Transition metal catalyzed cross-coupling reactions that create C(sp^2^)–C(sp^2^) bonds have had a dramatic impact on areas as diverse as materials science, agrochemicals, and pharmaceuticals.^1^ Although, such reactions have dominated the pharmaceutical industry over the last 50 years, in recent years it has been recognized that there is greater clinical success with increased numbers of sp^3^ carbons, which in turn has fueled increasing demand for methods to construct C(sp^3^)–C(sp^3^) bonds.^2^ However, this is a much more challenging proposition, since, in addition to C(sp^3^)–C(sp^3^) bonds being inherently more difficult to form via cross-coupling reactions relative to C(sp^2^)–C(sp^2^) bonds (due to reduced reactivity, increased steric hindrance, and deleterious side reactions), issues of controlling both relative and absolute stereochemistry often arise.^3^ This field promises to be the next major challenge in asymmetric synthesis.

Stereocontrolled C(sp^3^)–C(sp^3^) cross-couplings can be divided into two broad categories: (i) enantiospecific cross-couplings and (ii) enantioselective cross-couplings (Scheme 1a). In reactions where a single stereogenic center is generated, numerous examples of both strategies have been reported.^4^ However, it is much more challenging to construct C(sp^3^)–C(sp^3^) bonds where vicinal stereogenic centers are generated, but some success has been achieved. For example, Fu developed a doubly stereoconvergent Negishi-type cross-coupling of racemic alkylzinc reagents (β-zincated amides) with racemic propargylic halides using a chiral nickel/diamine catalyst, where only one of the two possible diastereoisomers could be obtained.^5^ An alternative strategy is to adopt a two-catalyst system with prochiral C(sp^2^) nucleophiles and racemic C(sp^3^) electrophiles, which allows all four stereoisomers to be prepared at will because each stereogenic center is independently controlled by one of the two catalysts.^6^ This stereodivergent dual catalysis was pioneered by Carreira who combined a chiral enamine nucleophile with a racemic allyl electrophile, enabling C(sp^3^)–C(sp^3^) bond formation with excellent stereocontrol. While powerful, both of the above methods require key functional groups on the nucleophile to achieve high reactivity and stereoselectivity, β-zincated amides^5^ or enolizable carbonyl compounds.^7^ The development of methodologies for the formation of C(sp^3^)–C(sp^3^) vicinal chiral centers leading to stereodivergent synthesis, continues to captivate the synthetic community.^8^

**Scheme 1:**
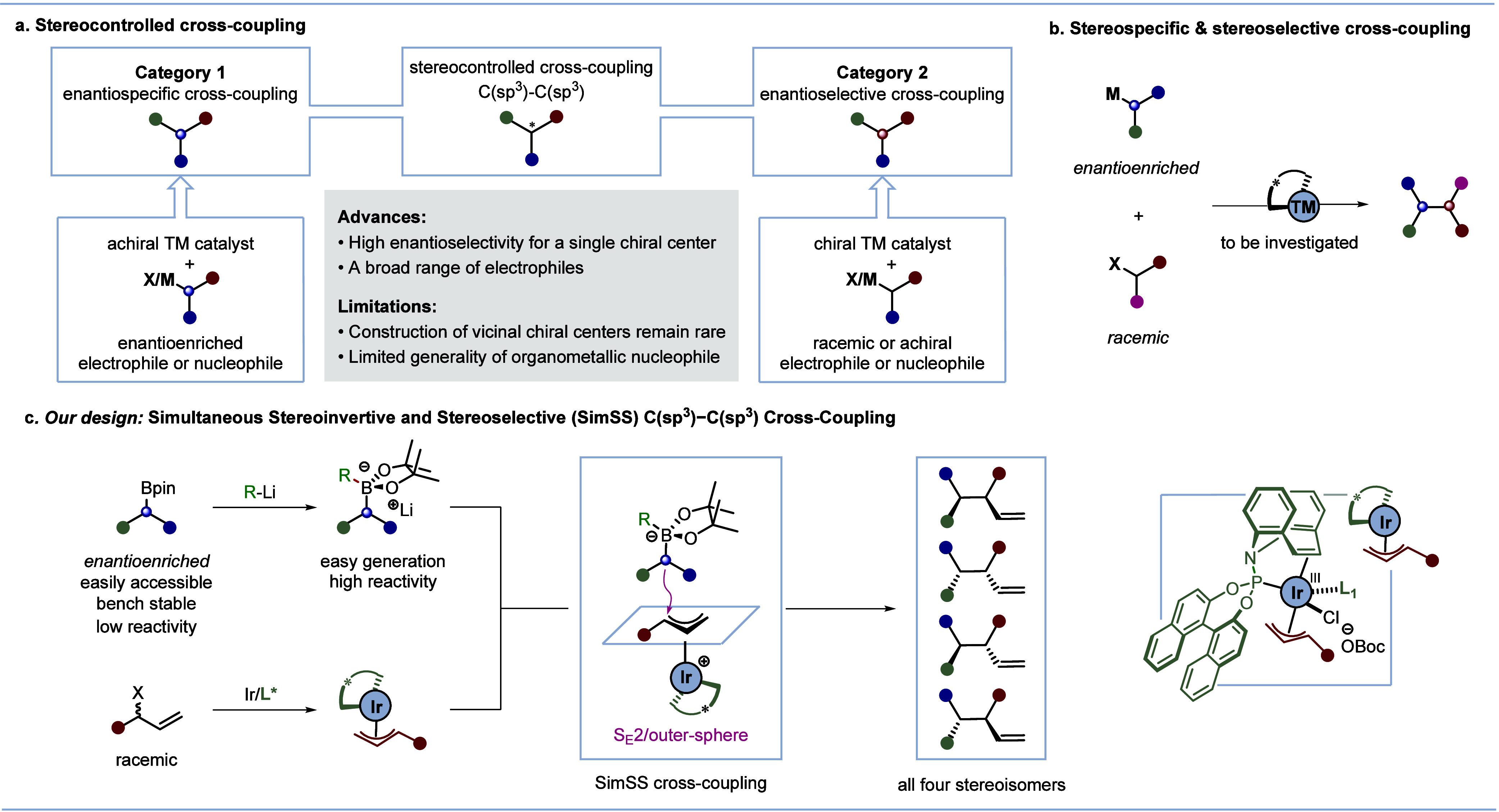
Previous Stereocontrolled Cross-Coupling Reactions and Our Reaction Design

Because of the numerous examples of both enantiospecific and enantioselective C(sp^3^)–C(sp^3^) cross-couplings,^4^ we envisaged a general approach that combined the broad features of the two categories into a single process: the enantiospecific reaction of an enantioenriched nucleophile^9^ with the enantioselective reaction of a racemic electrophile (Scheme 1b). Such a cross-coupling would enable the synthesis of all possible diastereoisomers and enantiomers, because both enantiomers of the chiral nucleophile would be available, as would each enantiomer of the chiral catalyst required to generate the chiral electrophile. To realize this vision, the chiral nucleophile required the following features: (i) easy synthesis, (ii) configurational stability, and (iii) sufficient reactivity. We considered using boronic esters since they are easy to access and configurationally stable.^10,11^ Furthermore, upon reaction with an organolithium, we^12^ (and others^13,14^) have shown that the corresponding boronate complexes behave as nucleophiles, reacting with a broad range of electrophiles with inversion of configuration. For the chiral electrophile, we considered the π-allyl iridium^15−17^ complexes developed by Carreira,^18^ because they had been shown to react with a range of nucleophiles^17−20^ including primary alkyl zinc reagents.^20^

Herein, we describe our success in developing C(sp^3^)–C(sp^3^) cross-coupling reactions of enantioenriched boronates^11a,11b,21^ with π-allyl iridium complexes, which occur through a simultaneous stereoinvertive and stereoselective (SimSS) process, to construct vicinal stereogenic centers and show that this strategy can be used to create any enantiomer and diastereoisomer at will (Scheme 1c).^22^ During the course of this work, Cho, Park, and co-workers reported a stereodivergent allyl–allyl coupling between branched allyllic alcohols and α-silyl-substituted allylboronate esters catalyzed by the same chiral iridium complex.^23^ Nonetheless, a method enabling the use of more easily accessible boronic esters would provide a more general and broader chemical space for the concept of SimSS C(sp^3^)–C(sp^3^) cross-coupling.

We first surveyed the SimSS coupling of enantioenriched benzylic boronic ester (*S*)-**1** with racemic allylic carbonate **3** (Scheme 2, Tables S1 and S2 in the Supporting Information). The boronic ester (*S*)-**1** was treated with different phenyl lithium reagents to form the tetracoordinated boronate complex **2**, which was subsequently subjected to an Ir-catalyzed asymmetric allylation with allylic carbonate **3**. We found that bis(trifluoromethyl)phenyl lithium **Li-1** served as a highly effective activator,^11^ affording the allylation product **3** in 90% isolated yield with >99% ee, 95:5 dr, and a branched-to-linear ratio (b:l) of 98:2. Phenyl lithium **Li-2** behaved similarly, giving nearly identical results; however, the electron-rich anisyllithium **Li-3** failed. Consequently, phenyl lithium **Li-2** was selected as the optimum activator due to its commercial availability. The reaction was selective at the alkyl unit, and no reaction between the aryl group and the electrophile was observed.

**Scheme 2:**
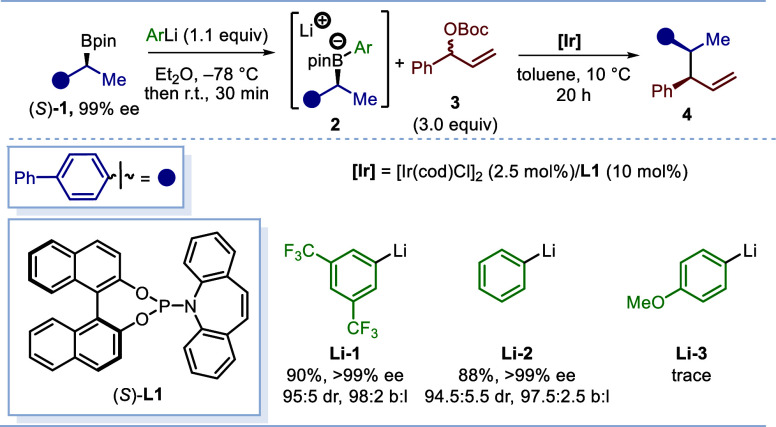
Optimization for Phenyl Lithium Reagents Reaction conditions: **2** was preformed using boronic ester **1** (0.2 mmol); yields are of isolated products; er was determined by HPLC analysis. The dr and b:l were determined by GC analysis.

Having established suitable conditions, we embarked on evaluating the substrate scope (Scheme 3). We first investigated the scope with respect to the racemic allylic carbonate. This showed that electron-rich and -deficient aryl groups on the aryl–allyl carbonate were tolerated, giving similar levels of regio-, enantio-, and diastereoselectivity (**4**, **6**, **8**, **10**). Using the enantiomeric ligand (*R*)-**L1** led to the opposite diastereoisomer with similarly high levels of selectivity (**5**, **7**, **9**, **11**), showing that essentially no matched/mismatched effects are in operation. Outside of dual catalysis,^7,8^ matched/mismatched effects are almost always seen when constructing vicinal stereogenic centers,^24^ but our unique system does not suffer from such effects. Ortho- and meta-substituted aryl–allyl carbonates (**12**–**15**) were also suitable coupling partners, providing products with high reactivity and selectivity. Heteroaromatics could also be employed, including a thiophene-substituted allylic carbonate, which gave **16** in 75% yield with 92.5:7.5 dr and >99% ee when using (*S*)-**L1**, whereas (*R*)-**L1** provided diastereoisomer **17** in 53% yield with 7.5:92.5 dr and >99% ee. Similarly, indole-derived coupling products **18** and **19** could be obtained in good yields and selectivities by using (*S*)-**L1** and (*R*)-**L1**. Notably, an alkynyl-substituted allylic carbonate was readily converted to the corresponding products **20** and **21**, again with similar levels of selectivity.

**Scheme 3:**
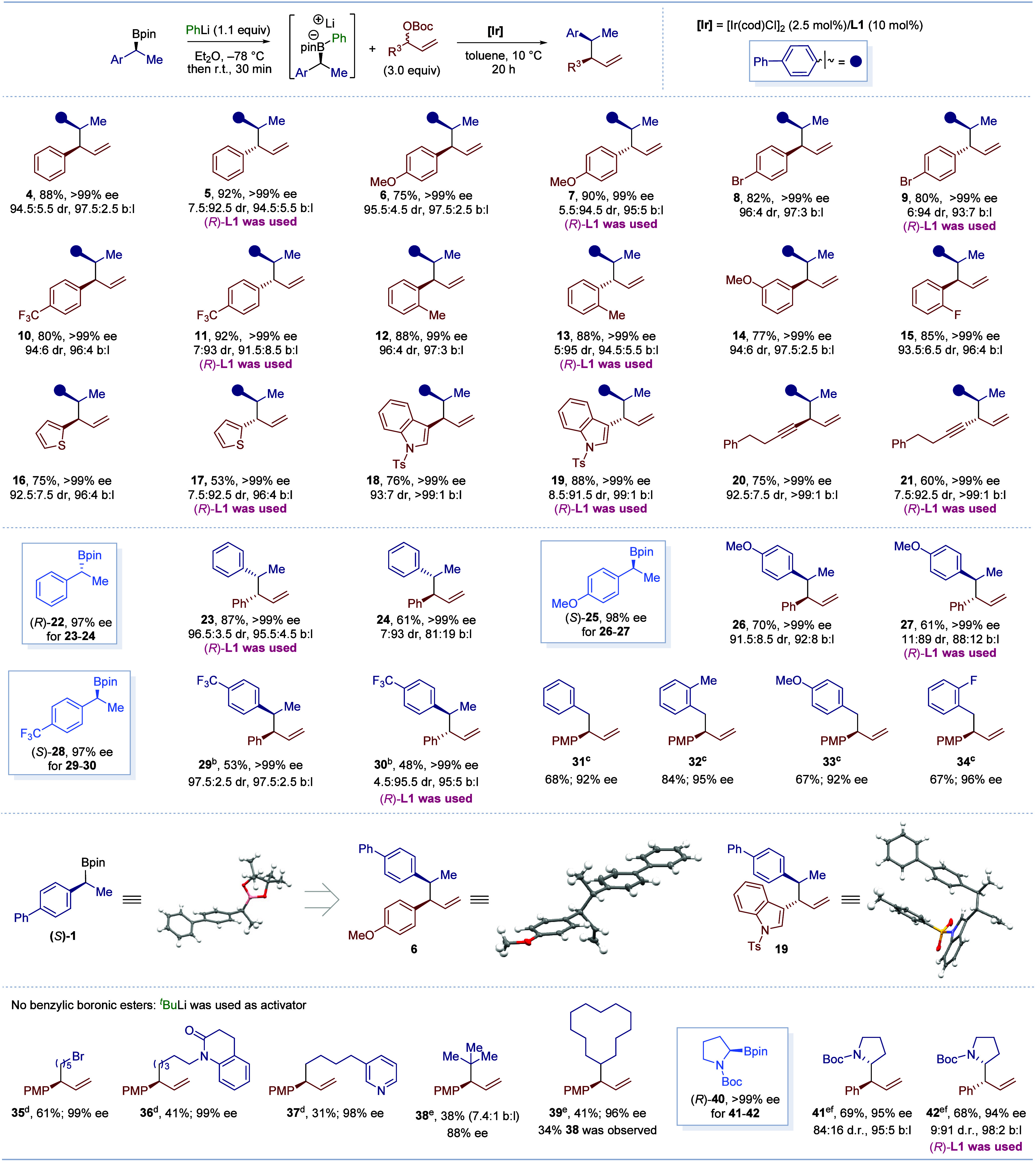
Substrate Scope of Electrophile and Boronic Esters Reaction conditions: Reaction was preformed using boronic esters (0.2 mmol); yields are of isolated products; er was determined by HPLC analysis. The dr and b:l were determined by GC analysis. **Li-1** was used. THF solvent at RT. THF solvent at 40 °C. DCM solvent at RT. [Ir(cod)Cl]_2_ (5 mol %)/**L1** (20 mol %) was used.

We further expanded the reaction scope to various other enantioenriched benzylic boronic esters (**22**, **25**, and **28**). A clear trend emerged when we explored electronic effects of the aromatic ring: higher dr values were observed for electron-withdrawing substituents (4-CF_3_, **28**) compared to electron-rich (4-OMe, **25**) or neutral substituents (**22**). Matched and mismatched effects were again not observed in terms of diastereoselectivity, although in the case of **25**, there was a small difference in the branched/linear ratios for diastereoisomers **26** and **27**. In the case of benzylic substrates bearing electron withdrawing groups (**28**), we found that PhLi resulted in decomposition of the boronate complex before addition of the other components, but the high yields and selectivities could be restored when using 3,5-bis(trifluoromethyl)phenyl lithium (**Li**-**1**) as the activator (for a comparison of the performance of PhLi vs **Li**-**1** with various substrates, see Table S3 in the Supporting Information). We believe that using a more electron-deficient aryl group stabilizes the boronate complex, preventing it from fragmenting to the aryl boronic ester and benzylic lithium species, which subsequently decomposes. In addition to secondary boronic esters, primary benzylic boronic esters could also be employed, leading to products **31**–**34**, with high levels of enantioselectivity. The configurations of enantioenriched boronic ester (*S*)-**1** and products **6** and **19** were determined by X-ray crystallographic analysis, which showed that inversion of configuration had occurred at the boronic ester. Attempts to extend the reaction to non-benzylic boronic esters under the same conditions were initially unsuccessful. However, we found that using *tert*-butyl lithium (^*t*^BuLi) as the activator instead of PhLi for these less reactive substrates now switched on the chemistry. Primary alkyl boronic esters bearing synthetically useful functional groups, for example, bromide **35**, amide **36**, and pyridine **37**, gave the coupled products with excellent enantioselectivity (98–99% ee). The tertiary boronic ester ^*t*^BuBpin **38** could also be used, but we encountered a minor amount of the linear regioisomer. In the case of secondary alkyl boronic esters, activation by ^*t*^BuLi led to a mixed boronate complex housing two different alkyl substituents that both competed in the transfer, yet the desired secondary coupled product **39** was still formed in moderate yield. Importantly, chiral proline derived boronic ester (*R*)-**40** could also be employed, using ^*t*^BuLi as the activator, leading to products **41** and **42** with high levels of selectivity and group transfer.

We then demonstrated the versatility of our method through the high yielding syntheses of all four stereoisomeric products (**8** and **9**, **43** and **44**) of the reaction of boronic ester **1** with allylic carbonate **3**, with excellent regio-, diastereo- and enantioselectivities obtained by simply altering the enantiomer of the boronic ester or ligand employed (Scheme 4a). The reaction could be conducted on a gram scale (1.23 g) without compromising yield or selectivity (Scheme 4b). In addition, the recovered allylic carbonate (*R*)-**3** was obtained in >99% ee, indicating that this reaction underwent efficient kinetic resolution (Scheme 4b).^25,26^ Significantly, we showcased the robustness of our method by achieving a one-pot synthesis of product **4** from alkene **45**. In this process, **37** underwent Cu-catalyzed asymmetric hydroboration^27^ followed by our SimSS cross-coupling in a single reaction flask, giving product **4** in 75% yield and with almost identical selectivity to that obtained with purified boronic ester (Scheme 4c).

**Scheme 4:**
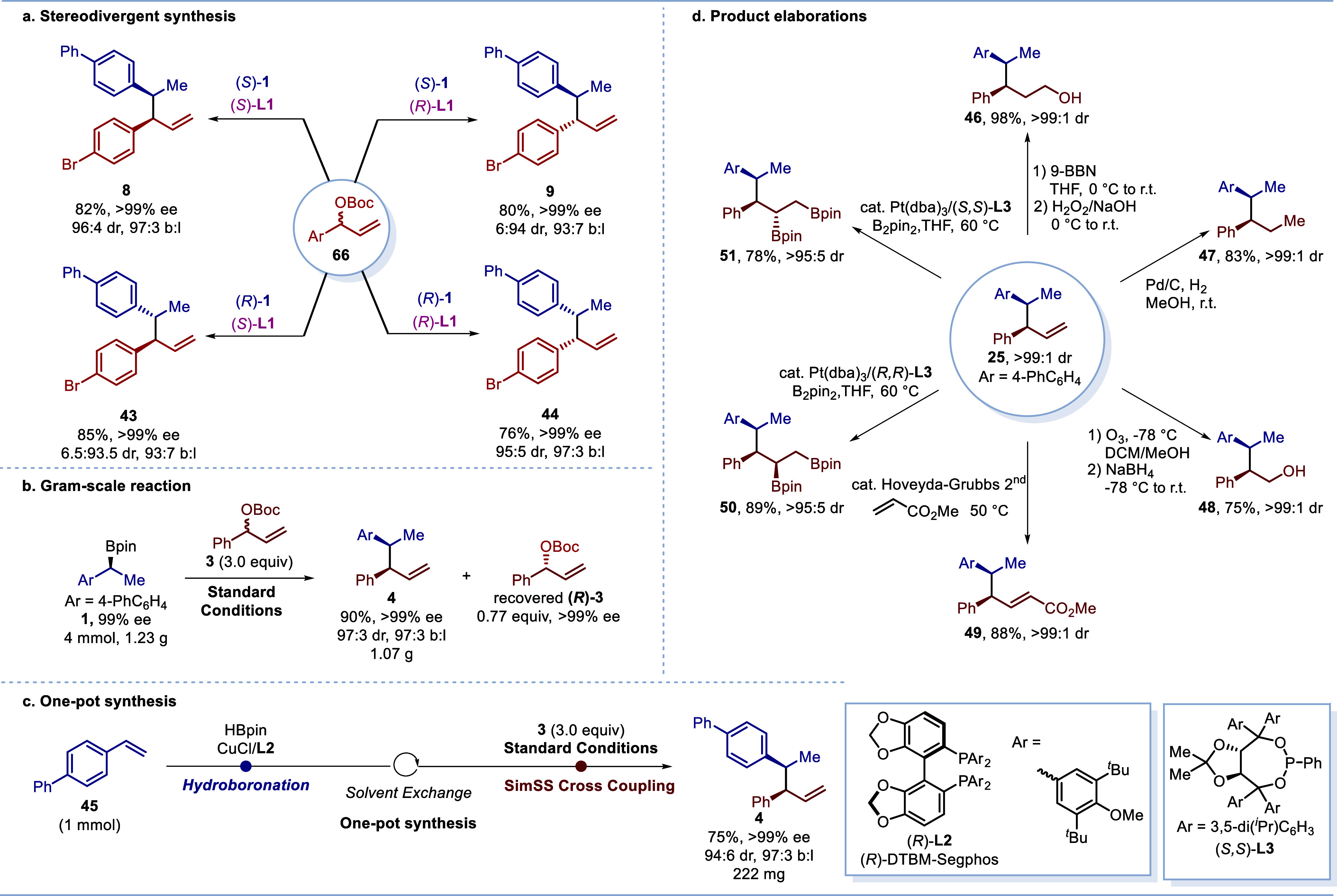
Stereodivergent Synthesis, Gram-Scale Reaction, One-Pot Synthesis, and Product Elaborations

Through further transformations, the synthetic utility of product **4** was demonstrated (Scheme 4d). Hydroboration/oxidation provided alcohol **46** and hydrogenation gave 2,3-diarylpentane **47**, both in excellent yield. Ozonolysis followed by reduction gave alcohol **48**, and olefin metathesis^28^ delivered enoate **49**. Notably, by employing Morken’s asymmetric diboration^29^ chemistry and using both the (*R*,*R*) and (*S*,*S*) ligands, diastereoisomeric diboron compounds **50** and **51** could be obtained with very high selectivity in both cases.

The stereochemistry of the product also showed that the enantioselectivity of allylation was in line with the established model,^17b,18h,18i^ indicating that the enantioenriched boronate complex reacts with the π-allyl iridium complex through an inversion/outer-sphere pathway, leading to SimSS cross-coupling (Scheme 3). Through detailed DFT and extensive kinetic studies, it has been established that the nucleophilic addition step is the turnover limiting step for related tetracoordinated boronate complexes with π-allyl-Ir,^18i,19b^ and so it can be expected that the same would apply here. Additionally, the high kinetic resolution is a result of one enantiomer of the racemic allylic carbonates undergoing oxidative addition at a much higher rate than the other enantiomer (Scheme 4b).^17b^ These observations support the catalytic cycle depicted in (Scheme 5a). The active catalytic species **Ir-1**, generated from an iridium(I) precursor and **2** equiv of phosphoramidite/olefin ligand **L1**, undergoes oxidative addition with high enantioselectivity for the (*S*)-enantiomer of the allylic electrophile to furnish the π-allyl-iridium intermediate **Ir-2**. This highly electrophilic species reacts with the enantioenriched boronate complex through an outer-sphere pathway, enabling the formation of the C(sp^3^)–C(sp^3^) bond with high regio- and stereoselectivity and returning **Ir-1** for further catalysis.

**Scheme 5:**
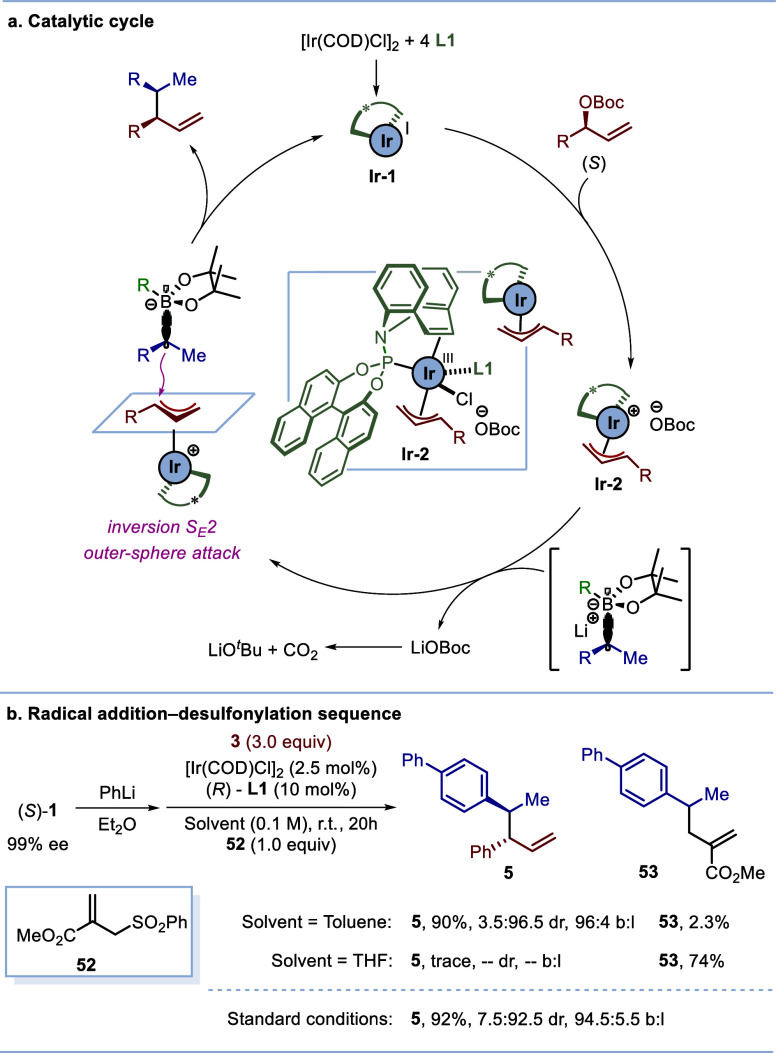
Mechanism Insights

Careful analysis of the stereochemistry of the minor diastereomer established that complete inversion did not occur at the boronic ester. We suspected that some leakage from the invertive pathway may have occurred through a single electron transfer (SET) process, producing lower-than-expected diastereoselectivity. In order to probe this, the reaction was repeated in the presence of the allylic sulfone radical trap **52** (Scheme 5b). This gave product **5** with higher diastereoselectivity, together with a small amount of the radical-trapped acrylate **53**, confirming that the SET process is the origin of erosion of diastereoselectivity. The extent of the radical pathway was highly solvent dependent, as conducting the reaction in THF gave the radical-trapped acrylate **53** exclusively.

In conclusion, we have developed a simultaneous stereoinvertive and stereoselective (SimSS) cross-coupling of boronic esters with racemic allylic carbonates using a chiral iridium catalyst. Success in these endeavors has demonstrated that the C(sp^3^)–C(sp^3^) coupling between enantioenriched organometallics and racemic allylic electrophiles can be leveraged to provide an extremely powerful tool for building vicinal chiral centers with independent control.

## Supporting Information Available

Experimental procedures and characterization data for new compounds (PDF)
