# Supplementary material for: Simultaneous Stereoinvertive and Stereoselective C(sp3)–C(sp3) Cross-Coupling of Boronic Esters and Allylic Carbonates
Source: J Am Chem Soc. 2024 May 9;146(20):13719–26. doi: 10.1021/jacs.4c03686 (PMC11117407; doi:10.1021/jacs.4c03686)

Supplementary Information for

Simultaneous Stereoinvertive and Stereoselective C(sp3)−C(sp3) Cross-Coupling of Boronic Esters and Allylic Carbonates

Hong-Cheng Shen[1†], Ze-Shu Wang[1†], Adam Noble[1], Varinder K. Aggarwal[1*]

Correspondence to: v.aggarwal@bristol.ac.uk

[1]School of Chemistry, University of Bristol, Cantock's Close, Bristol, BS8 1TS, U.K.
[†]These authors contributed equally to this work.
[*]Email: v.aggarwal@bristol.ac.uk.

Table of Contents

1. MATERIALS AND GENERAL METHODS 4

1.1. Glassware, Solvents and Reagents 4

1.2. Instrumentation 4

1.3. Naming of Compounds 5

2. EXPERIMENTAL DATA 6

2.1. Reaction Optimization 6

2.2. General Procedures 9

2.2.1. General Procedure A: SimSS cross coupling (product: 4-21, 23, 24, 26, 27, 43, 44) 9

2.2.2. General Procedure B: 1°Benzylic boronic esters (product: 31-34) 10

2.2.3. General Procedure C: SimSS cross coupling using 3,5-bis(trifluoromethyl)phenyl lithium (product: 4-21, 23, 24, 26, 27, 29, 30, 43, 44) 10

2.2.4. General Procedure D: 1°Alkyl boronic esters (product: 35-37) 12

2.2.5. General Procedure E: 3° And 2° alkyl boronic esters (product: 38, 39, 41, 42). 13

2.2.6. General Procedure F: Hydroboration-oxidation 13

2.3. Synthesis of Starting Materials 14

2.3.1. Synthesis of racemic allylic carbonates 14

2.3.2. Synthesis of previously reported boronic esters used in this project. 14

2.3.3. Characterization data for boronic esters 14

2.4. Procedures for Transformation of Products 17

2.4.1. Procedure G: Hydrogenation of alkene 17

2.4.2. Procedure H: Ozonation followed by reduction of alkene 17

2.4.3. Procedure I: Olefin metathesis 18

2.4.4. Procedure J: Asymmetric diboration of alkene 18

2.4.5. Procedure K: Asymmetric diboration of alkene 19

2.5. Procedures for Scale Up Reaction, One-Pot Synthesis, and Radical addition–desulfonylation sequence 20

2.5.1. Scale up reaction and kinetic resolution of racemic allylic carbonate 20

2.5.2. One-pot synthesis 21

2.5.3. Radical addition–desulfonylation sequence 22

2.6. Unsuccessful Substrates 24

2.7. Crystallography 24

2.8. Characterization Data for Products. 27

3. REFERENCE..**84**

4. NMR SPECTRA..**86**

1. MATERIALS AND GENERAL METHODS

1.1. Glassware, Solvents and Reagents

All manipulations were performed with oven-dried (130 °C for a minimum of 12 h) or flame-dried glassware using standard Schlenk techniques under an atmosphere of nitrogen, unless otherwise stated.

All anhydrous solvents were commercially supplied or dried using an Anhydrous Engineering alumina column drying system (dichloromethane, toluene, diethyl ether, and tetrahydrofuran). Reagents were purchased from commercial sources and used as received. All organolithium reagents were titrated against *N*-benzylbenzamide.[1]

1.2. Instrumentation

Thin layer chromatography (TLC) was performed using Merck Kieselgel 60 F254 fluorescent treated silica, which was visualised under UV light, or by staining with aqueous basic potassium permanganate followed by heating, or Hanessian's stain (CAM stain) followed by heating, or *p*-anisaldehyde solution followed by heating, as stated.

Flash column chromatography (FCC) was carried out using Sigma-Aldrich silica gel (60 Å, 230-400 mesh, 40-63 µm), Biotage Isolera™ flash purification system or boric acid impregnated silica gel.[2] In cases where automated column chromatography was employed the solvent gradient and flow rate are indicated.

NMR spectra were recorded at various field strengths, as indicated, using Bruker 400 MHz, Varian VNMR 400 MHz, Bruker Cryo 500 MHz or Bruker Cryo 600 MHz for ^{1}H, ^{11}B, and ^{13}C acquisitions. All NMR spectra were recorder at 25 °C unless otherwise stated. Chemical shifts (δ) are reported in parts per million (ppm) and referenced to $CDCl_3$ (^{1}H: 7.26 ppm; ^{13}C: 77.16 ppm). Coupling constants (J) are given in Hertz (Hz) and refer to apparent multiplicities (s = singlet, d = doublet, t = triplet, q = quartet, quin = quintet, hex = hextet, h = heptet, m = multiplet, brs = broad signal, dd = doublet of doublets, etc.). The ^{1}H NMR spectra are reported as follows: chemical shift (multiplicity, coupling constants, number of protons).

HPLC analyses were performed on Agilent 1100 system with Daicel Chiralpak columns.

High resolution mass spectra (HRMS) were recorded on a Bruker Daltonics MicrOTOF II by Electrospray Ionisation (ESI); a Thermo Scientific QExactive by Electron Ionisation (EI); a Thermo Scientific Orbitrap Elite by ESI or Atmospheric Pressure Chemical Ionisation (APCI); or a Bruker UltrafleXtreme by Matrix-assisted Laser Desorption/Ionisation (MALDI).

IR spectra were recorded neat as a thin film on a Perkin Elmer Spectrum One FT-IR. Selected absorption maxima (ν_{max}) are reported in wavenumbers (cm^{-1}).

Gas chromatography–mass spectrometry (**GC-MS**) was recorded on an Agilent 6890 Series GC and 5973 detectors using a HP-5MS UI column (15 m × 0.25 mm × 0.25 μm).

1.3. Naming of Compounds

Compound names are those generated by ChemDraw Professional 20.0 software (PerkinElmer), following the IUPAC nomenclature.

2. EXPERIMENTAL DATA

2.1. Reaction Optimization

Table S1: Reaction Optimization for enantioenriched secondary benzylic boronic ester[a]

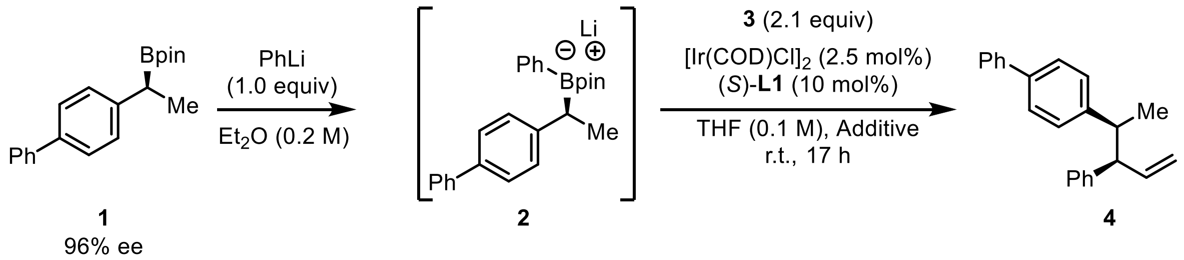

Entry	Conditions	Yield	dr	b:l
1[b]	none	<10%	77:23	67:33
2[b]	1.1 equiv Ar^FLi, THF in Step1	73%	88:12	93:7
3[b]	1.1 equiv Ar^FLi	76%	92:8	93:7
4[b]	1.1 equiv Ar^FLi, 50% styrene	76%	93:7	95:5
5[c]	1.1 equiv Ar^FLi	86%	92:8	93:7
6[c]	1.1 equiv Ar^FLi, 2.5 equiv **3**	87%	92:8	93:7
7[c]	1.1 equiv Ar^FLi, 3.0 equiv **3**	90%	92:8	93:7
8[c]	1.0 equiv Ar^FLi	73%	93:7	95:5
9[c]	1.1 equiv Ar^FLi, DCM for Step 2	85%	91:9	96:4
10[c]	1.1 equiv Ar^FLi, Toluene for Step 2	80%	93:7	96:4
11[c]	1.1 equiv Ar^FLi, 2.0 equiv ^{t}BuOH	55%	94:6	95:5
12[c]	1.1 equiv Ar^FLi, Toluene for Step 2, 10 °C	69%	94:6	96:4
13[c]	1.1 equiv Ar^FLi, Toluene for Step 2, 10 °C, 3.0 equiv **3**	92%	94:6	96:4

Ar^FLi = 3,5-di(CF_3)C_6H_3Li. [a]0.2 mmol **1** was used; The yield was isolated yield; dr and b:l were determined by NMR analysis of product. [b]**1** was added to ArLi in Et_2O. [c]ArLi in Et_2O was added to **1** in Et_2O.

Table S2: Reaction optimization for enantioenriched secondary benzylic boronic esters[a]

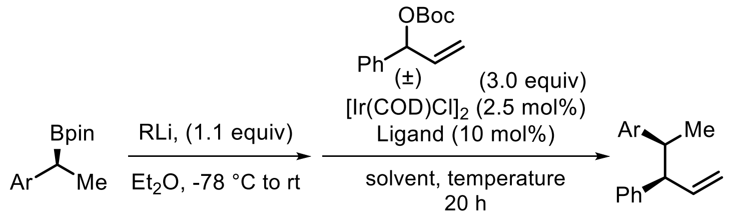

entry	boronic ester	ligand	RLi	solvent	temperature	yield	dr	b:l
1	(*S*)-**1**	(*S*)-**L1**	**Li-1**	toluene	10 °C	90%	95:5	98:2
2	(*S*)-**1**	(*S*)-**L1**	**Li-2**	toluene	10 °C	88%	94.5:5.5	97.5:2.5
3	(*S*)-**1**	(*S*)-**L1**	**Li-3**[b]	toluene	10 °C	trace	--	--
4	(*S*)-**1**	(*S*)-**L1**	**Li-1**	THF	r.t.	84%	96:4	96:4
5	(*S*)-**1**	(*S*)-**L1**	**Li-2**	THF	r.t.	38%[c]	71:29	59:41
6	(*S*)-**1**	(*S*)-**L1**	**Li-3**[b]	THF	r.t.	trace	--	--
7	(*R*)-**22**	(*R*)-**L1**	**Li-1**	toluene	10 °C	72%	94:6	95:5
8	(*R*)-**22**	(*R*)-**L1**	**Li-2**	toluene	10 °C	87%	96.5:3.5	95.5:4.5
9	(*R*)-**22**	(*R*)-**L1**	**Li-2**	THF	r.t.	71%	95:5	93:7
10	(*R*)-**22**	(*R*)-**L1**	**Li-3**[b]	toluene	10 °C	71%	96:4	96.5:3.5

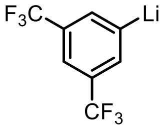

Li-1

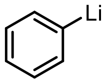

Li-2

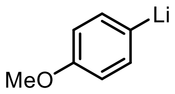

Li-3

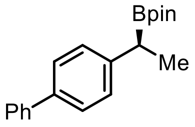

(*S*)-1, 99% ee

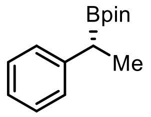

(*R*)-**22**, 97% ee

[a]0.2 mmol boronic ester was used. The yield, dr and b:l was determined by GC analysis. [b]*p*-$MeOC_6H_4Li$ was prepared *in situ* by adding ^{n}BuLi (1.1 equiv) to *p*-$MeOC_6H_4Br$ (1.1 equiv) in THF at −78 °C for 10 min. [c]The major byproduct is 4-ethylbiphenyl.

Table S3: Substrate scope with the utilization of PhLi and 3,5- $(CF_3)_2C_6H_3Li$[a]

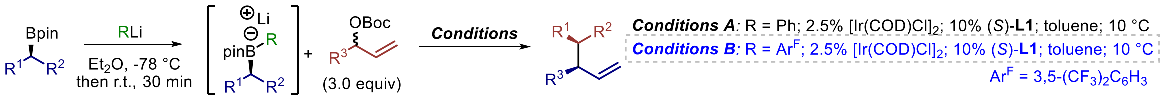

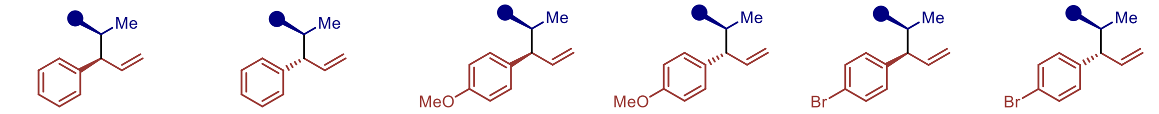

Compound	Conditions A	Conditions B
**4**	88%, >99% ee; 94.5:5.5 dr, 97.5:2.5 b:l	90%, >99% ee; 95:5 dr, 98:2 b:l
**5**	92%, >99% ee; 7.5:92.5 dr, 94.5:5.5 b:l	87%, >99% ee; 7:93 dr, 96:4 b:l; (*R*)-**L1** was used
**6**	75%, >99% ee; 95.5:4.5 dr, 97.5:2.5 b:l	87%, >99% ee; 96:4 dr, 97:3 b:l
**7**	90%, 99% ee; 5.5:94.5 dr, 95:5 b:l	89%, 99% ee; 8:92 dr, 95:5 b:l; (*R*)-**L1** was used
**8**	82%, >99% ee; 96:4 dr, 97:3 b:l	85%, >99% ee; 97:3 dr, 96:4 b:l
**9**	80%, >99% ee; 6:94 dr, 93:7 b:l	87%, >99% ee; 5.5:94.5 dr, 95:5 b:l; (*R*)-**L1** was used

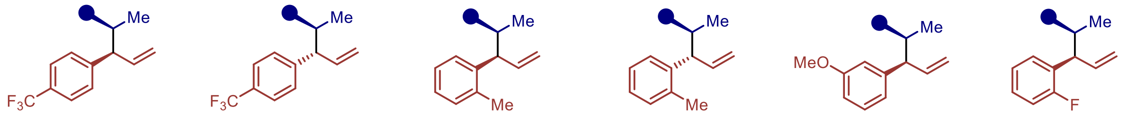

Compound	Conditions A	Conditions B
**10**	80%, >99% ee; 94:6 dr, 96:4 b:l	86%, >99% ee; 95.5:4.5 dr, 96:4 b:l
**11**	92%, >99% ee; 7:93 dr, 91.5:8.5 b:l	84%, >99% ee; 6:94 dr, 92:8 b:l; (*R*)-**L1** was used
**12**	88%, 99% ee; 96:4 dr, 97:3 b:l	91%, 99% ee; 96:4 dr, 96:4 b:l
**13**	88%, >99% ee; 5:95 dr, 94.5:5.5 b:l	80%, >99% ee; 6:94 dr, 95:5 b:l; (*R*)-**L1** was used
**14**	77%, >99% ee; 94:6 dr, 97.5:2.5 b:l	89%, >99% ee; 96:4 dr, 98:2 b:l
**15**	85%, >99% ee; 93.5:6.5 dr, 96:4 b:l	79%, >99% ee; 95.5:4.5 dr, 96:4 b:l

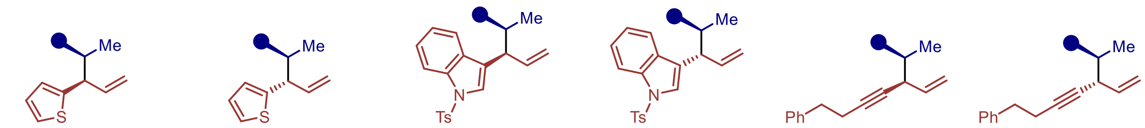

Compound	Conditions A	Conditions B
**16**	75%, >99% ee; 92.5:7.5 dr, 96:4 b:l	72%, >99% ee; 95:5 dr, 98:2 b:l
**17**	53%, >99% ee; 7.5:92.5 dr, 96:4 b:l	50%, >99% ee; 15:85 dr, 94.5:5.5 b:l; (*R*)-**L1** was used
**18**	76%, >99% ee; 93:7 dr, >99:1 b:l	83%, >99% ee; 87:13 dr, 97:3 b:l
**19**	88%, >99% ee; 8.5:91.5 dr, 99:1 b:l	87%, >99% ee; 15:85 dr, 99:1 b:l; (*R*)-**L1** was used
**20**	50%, >99% ee; 90:10 dr, >99:1 b:l	92%, >99% ee; 95:5 dr, 96:4 b:l
**21**	60%, >99% ee; 7.5:92.5 dr, >99:1 b:l	76%, >99% ee; 15:85 dr, 92:8 b:l; (*R*)-**L1** was used

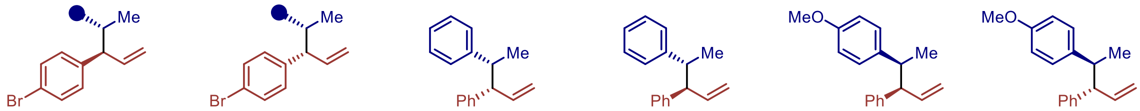

Compound	Conditions A	Conditions B
**35**	85%, >99% ee; 6.5:93.5 dr, 93:7 b:l	86%, >99% ee; 5.5:94.5 dr, 94:6 b:l
**36**	76%, >99% ee; 95:5 dr, 97:3 b:l	86%, >99% ee; 96.5:3.5 dr, 98:2 b:l; (*R*)-**L1** was used
**23**	87%, >99% ee; 96.5:3.5 dr, 95.5:4.5 b:l	72%, >99% ee; 94:6 dr, 95:5 b:l; (*R*)-**L1** was used
**24**	61%, >99% ee; 7:93 dr, 81:19 b:l	70%, >99% ee; 12:88 dr, 72:28 b:l
**26**	70%, >99% ee; 91.5:8.5 dr, 92:8 b:l	72%, >99% ee; 93:7 dr, 93:7 b:l
**27**	61%, >99% ee; 11:89 dr, 88:12 b:l	63%, >99% ee; 15:85 dr, 86.5:13.5 b:l; (*R*)-**L1** was used

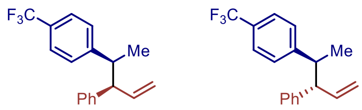

Compound	Conditions A	Conditions B
**30**	trace	53%, >99% ee; 97.5:2.5 dr, 97.5:2.5 b:l
**31**	trace	48%, >99% ee; 4.5:95.5 dr, 95:5 b:l; (*R*)-**L1** was used

[a]Isolated yields are reported. The enantiomeric excess (ee) was determined by HPLC analysis of the reaction products or their derivatives. The diastereoisomeric ratios (dr) and regioselectivity (branched:linear = b:l) were determined by GC analysis of the crude reaction products.

2.2. General Procedures

2.2.1. General Procedure A: SimSS cross coupling (product: 4-21, 23, 24, 26, 27, 43, 44)

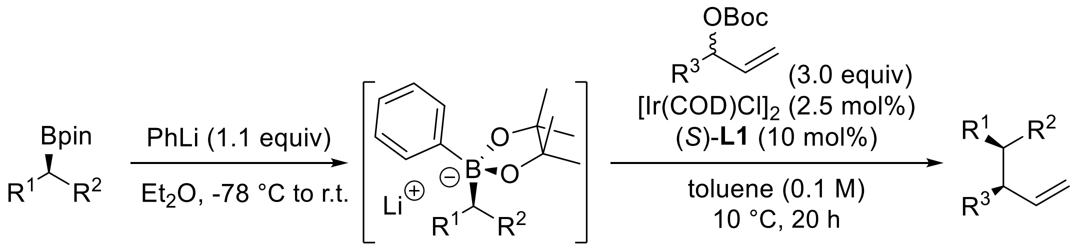

Step 1 boronate complex generation: To an oven-dried 10 mL Schlenk tube equipped with a magnetic stir bar was back up filled with N_2 3 times, and then chiral *sec*-boronic ester (0.2 mmol, 1.0 equiv) and 2 mL anhydrous Et_2O was added under N_2. The solution was cooled to −78 °C (dry ice/acetone) followed by addition of PhLi (1.67 M in pentane, 0.13 mL, 0.22 mmol) dropwise. The mixture was allowed to stir for 10 min at −78 °C and then warmed to ambient temperature and further stirred for 30 min to form boronate complex completely. The solvent was removed under high vacuum and refilled with N_2. **Step 2 asymmetric allylation:** In glovebox, to an oven-dried 7.0 mL vial equipped with a magnetic stir bar was added $[Ir(cod)Cl]_2$ (0.005 mmol, 2.5 mol%, 3.4 mg) and (*S*)-**L1** or (*R*)-**L1** (0.02 mmol, 10 mol%, 10.1 mg). The vial was sealed with a septum and removed from the glovebox, and then anhydrous toluene (1.0 mL) was added under N_2. The mixture was stirred for 30 min at ambient temperature and then added with allyl *tert*-butyl carbonate (0.6 mmol, 3.0 equiv) to form π-allyl iridium solution (This should be prepared during forming boronate complex at ambient temperature). The π-allyl iridium solution was then added to above boronate complex. The π-allyl iridium vial was washed with 1.0 mL toluene and then added to Schlenk tube. Finally, Schlenk tube was moved to 10 °C bath (cooled down with cryostat) and stirred for 20 h at this temperature. The crude reaction mixture was filtered through a pad of silica gel and filtrate was concentrated under reduced pressure. The crude material was purified by flash column chromatography (*n*-hexane/DCM) to afford the desired product.

2.2.2. General Procedure B: 1°Benzylic boronic esters (product: 31-34)

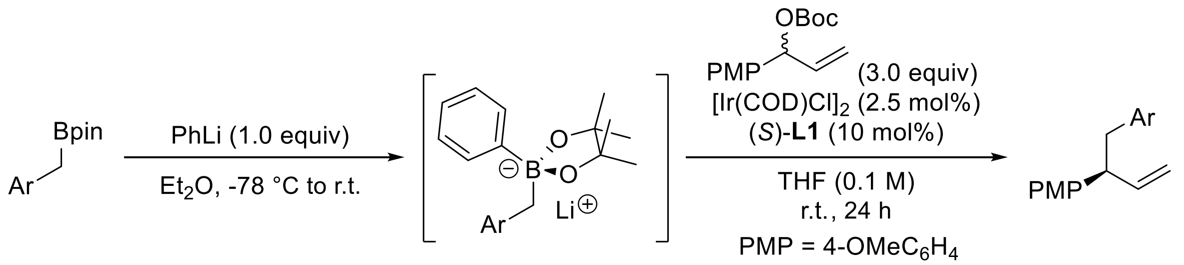

Step 1 boronate complex generation: An oven-dried 10 mL Schlenk tube equipped with a magnetic stir bar was back up filled with N_2 3 times, and then boronic ester (0.2 mmol, 1.0 equiv) and 1 mL anhydrous Et_2O was added under N_2. The solution was cooled to −78 °C (dry ice/acetone) and PhLi (1.67 M in pentane, 0.12 mL, 0.2 mmol) solution was added to the solution dropwise. The mixture was allowed to stir for 10 min at −78 °C and then warmed to ambient temperature and further stirred for 30 min to form boronate complex completely. The solvent was removed under high vacuum and refilled with N_2. **Step 2 asymmetric allylation:** In glovebox, to an oven-dried 7.0 mL vial equipped with a magnetic stir bar was added $[Ir(cod)Cl]_2$ (0.005 mmol, 2.5 mol%, 3.4 mg) and (*S*)-**L1** (0.02 mmol, 10 mol%, 10.1 mg). The vial was sealed with a septum and removed from the glovebox, and then anhydrous THF (1.0 mL) was added under N_2. The mixture was stirred for 30 min at ambient temperature and then added with allyl *tert*-butyl carbonate (0.6 mmol, 3.0 equiv) to form π-allyl iridium solution (This should be prepared during forming boronate complex at ambient temperature). The π-allyl iridium solution was then added to above boronate complex. The π-allyl iridium vial was washed with 1.0 mL THF and then added to Schlenk tube. Finally, Schlenk tube was allowed to be stirred at ambient temperature for 24 hours. The crude reaction mixture was filtered through a pad of silica gel and filtrate was concentrated under reduced pressure. The crude material was purified by flash column chromatography to afford the desired product.

2.2.3. General Procedure C: SimSS cross coupling using 3,5-bis(trifluoromethyl)phenyl

lithium (product: 4-21, 23, 24, 26, 27, 29, 30, 43, 44)

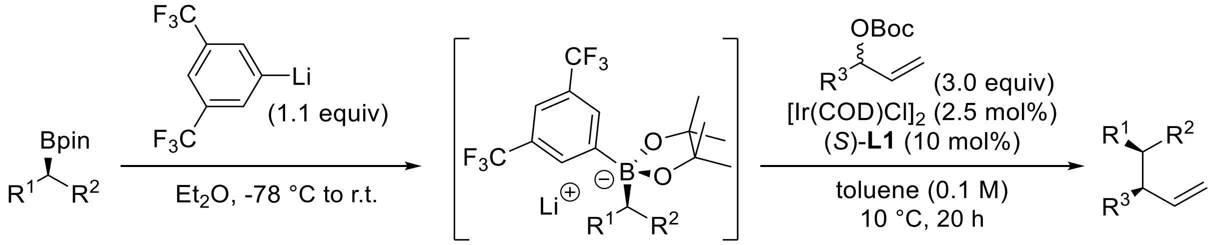

Step 1 boronate complex generation: In glovebox, to an oven-dried 7.0 mL vial equipped with a magnetic stir bar was sealed with a septum and removed from the glovebox and then 1-bromo-3,5-bis(trifluoromethyl)benzene (0.22 mmol, 1.1 equiv, 38 uL) and 1 mL anhydrous Et_2O were added sequentially under N_2. The solution was cooled to −78 °C (dry ice/acetone) and ^{n}BuLi (2.5 M in hexane, 0.22 mmol, 1.1 equiv) was added dropwise. The mixture was allowed to stir for 1 hour at −78 °C to form the Ar^{F}Li solution. Meanwhile, to an oven-dried 10 mL Schlenk tube equipped with a magnetic stir bar was back up filled with N_2 3 times, and then chiral *sec*-boronic ester (0.2 mmol, 1.0 equiv) and 1 mL anhydrous Et_2O was added under N_2. The solution was cooled to −78 °C (dry ice/acetone) and Ar^{F}Li solution was warmed to ambient temperature for 5 min and added to boronic ester solution dropwise. The mixture was allowed to stir for 10 min at −78 °C and then warmed to ambient temperature and further stirred for 30 min to form boronate complex completely. The solvent was removed under high vacuum and refilled with N_2. **Step 2 asymmetric allylation:** In glovebox, to an oven-dried 7.0 mL vial equipped with a magnetic stir bar was added $[Ir(cod)Cl]_2$ (0.005 mmol, 2.5 mol%, 3.4 mg) and (*S*)-**L1** or (*R*)-**L1** (0.02 mmol, 10 mol%, 10.1 mg). The vial was sealed with a septum and removed from the glovebox, and then anhydrous toluene (1.0 mL) was added under N_2. The mixture was stirred for 30 min at ambient temperature and then added with allyl *tert*-butyl carbonate (0.6 mmol, 3.0 equiv) to form *π*-allyl iridium solution (This should be prepared during forming boronate complex at ambient temperature). The *π*-allyl iridium solution was then added to above boronate complex. The *π*-allyl iridium vial was washed with 1.0 mL toluene and then added to Schlenk tube. Finally, Schlenk tube was moved to 10 °C bath (cooled down with cryostat) and stirred for 20 h at this temperature. The crude reaction mixture was filtered through a pad of silica gel and filtrate was concentrated under reduced pressure. The crude material was purified by flash column chromatography (*n*-hexane/DCM) to afford the

desired product.

2.2.4. General Procedure D: 1°Alkyl boronic esters (product: 35-37).

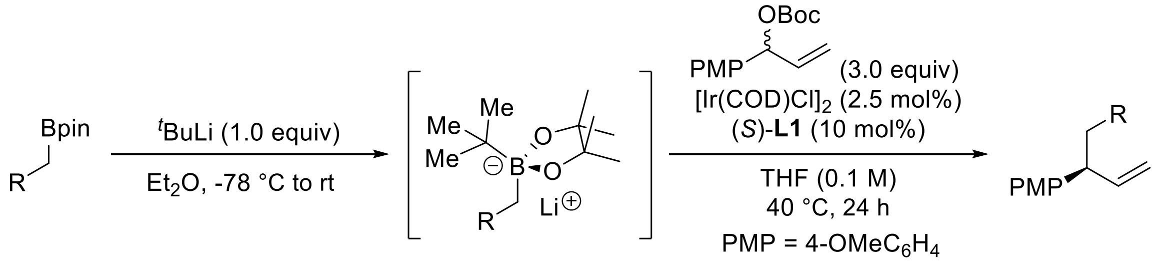

Step 1 boronate complex generation: An oven-dried 10 mL Schlenk tube equipped with a magnetic stir bar was back up filled with N_2 3 times, and then boronic ester (0.2 mmol, 1.0 equiv) and 1 mL anhydrous Et_2O was added under N_2. The solution was cooled to −78 °C (dry ice/acetone) and ^{t}BuLi (1.67 M in pentane, 0.12 mL, 0.2 mmol) solution was added to the solution dropwise. The mixture was allowed to stir for 10 min at −78 °C and then warmed to ambient temperature and further stirred for 30 min to form boronate complex completely. The solvent was removed under high vacuum and refilled with N_2. **Step 2 asymmetric allylation:** In glovebox, to an oven-dried 7.0 mL vial equipped with a magnetic stir bar was added $[Ir(cod)Cl]_2$ (0.005 mmol, 2.5 mol%, 3.4 mg) and (*S*)-**L1** (0.02 mmol, 10 mol%, 10.1 mg). The vial was sealed with a septum and removed from the glovebox, and then anhydrous THF (1.0 mL) was added under N_2. The mixture was stirred for 30 min at ambient temperature and then added with allyl *tert*-butyl carbonate (0.6 mmol, 3.0 equiv) to form π-allyl iridium solution (This should be prepared during forming boronate complex at ambient temperature). The π-allyl iridium solution was then added to above boronate complex. The π-allyl iridium vial was washed with 1.0 mL THF and then added to Schlenk tube. Finally, Schlenk tube was allowed to be stirred at 40 °C for 24 hours. The crude reaction mixture was filtered through a pad of silica gel and filtrate was concentrated under reduced pressure. The crude material was purified by flash column chromatography to afford the desired product.

2.2.5. General Procedure E: 3° And 2° alkyl boronic esters (product: 38, 39, 41, 42).

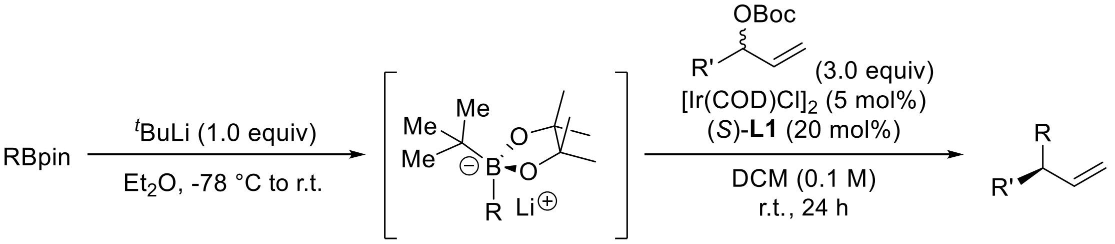

Step 1 boronate complex generation: An oven-dried 10 mL Schlenk tube equipped with a magnetic stir bar was back up filled with N_2 3 times, and then boronic ester (0.2 mmol, 1.0 equiv) and 1 mL anhydrous Et_2O was added under N_2. The solution was cooled to −78 °C (dry ice/acetone) and ^{t}BuLi (1.67 M in pentane, 0.12 mL, 0.2 mmol) solution was added to the solution dropwise. The mixture was allowed to stir for 10 min at −78 °C and then warmed to ambient temperature and further stirred for 30 min to form boronate complex completely. The solvent was removed under high vacuum and refilled with N_2. **Step 2 asymmetric allylation:** In glovebox, to an oven-dried 7.0 mL vial equipped with a magnetic stir bar was added $[Ir(cod)Cl]_2$ (0.01 mmol, 5 mol%, 6.8 mg) and (*S*)-**L1** (0.04 mmol, 20 mol%, 20.2 mg). The vial was sealed with a septum and removed from the glovebox, and then anhydrous DCM (1.0 mL) was added under N_2. The mixture was stirred for 30 min at ambient temperature and then added with allyl *tert*-butyl carbonate (0.6 mmol, 3.0 equiv) to form *π*-allyl iridium solution (This should be prepared during forming boronate complex at ambient temperature). The *π*-allyl iridium solution was then added to above boronate complex. The *π*-allyl iridium vial was washed with 1.0 mL anhydrous DCM and then added to Schlenk tube. Finally, Schlenk tube was allowed to be stirred at ambient temperature for 24 hours. The crude reaction mixture was filtered through a pad of silica gel and filtrate was concentrated under reduced pressure. The crude material was purified by flash column chromatography to afford the desired product.

2.2.6. General Procedure F: Hydroboration-oxidation

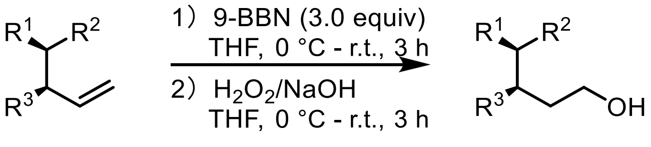

9-BBN (0.5 M in THF, 3 equiv) was added a solution of the alkene (0.1 mmol, 1.0 equiv) in THF (0.1 M) at 0 °C. The mixture was stirred for 15 min at 0 °C and 3 hours at room temperature. The reaction was cooled to 0 °C, then 3 N aqueous NaOH (1 mL) and 30% aqueous H_2O_2 (1 mL) were added. The reaction was allowed to warm to room temperature and stirring continued for 3 h before quenching with aqueous $Na_2S_2O_3$ at 0 °C and extracting with Et_2O three times. The organic layer was dried over $MgSO_4$, filtered, and concentrated under reduced pressure. The crude mixture was purified by flash column chromatography with hexanes/EtOAc as eluent to give the corresponding product.

2.3. Synthesis of Starting Materials

2.3.1. Synthesis of racemic allylic carbonates

All racemic allylic carbonates were synthesized according to relevant literatures.[3-4]

2.3.2. Synthesis of previously reported boronic esters used in this project.

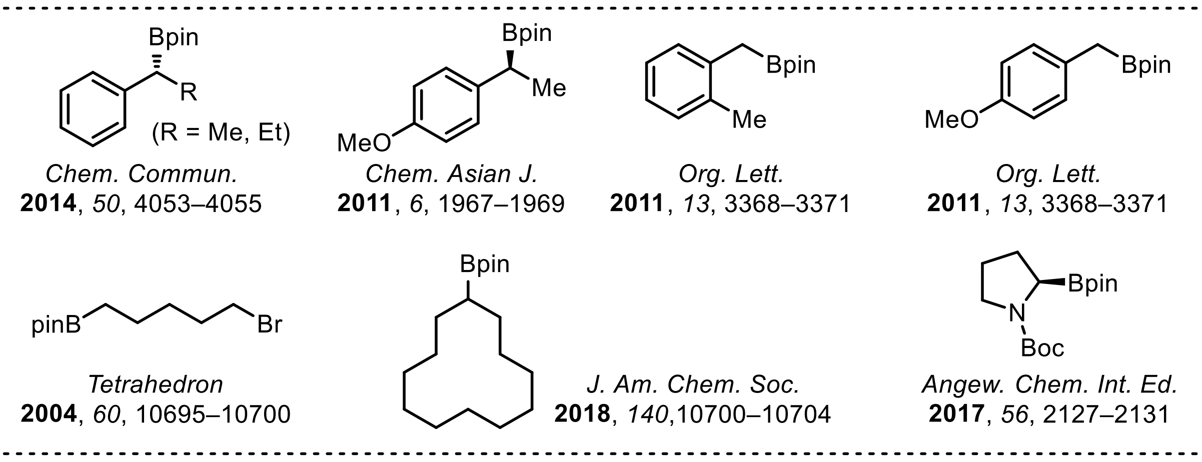

Scheme S1. Previously reported boronic ester substrates

2.3.3. Characterization data for boronic esters

(*S*)-2-(1-([1,1'-biphenyl]-4-yl)ethyl)-4,4,5,5-tetramethyl-1,3,2-dioxaborolane ((*S*)-1)

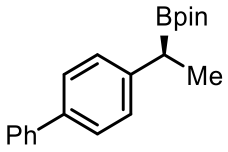

(*S*)-**1**

Prepared according to the referenced literature procedure.[5] **^{1}H NMR** (400 MHz, $CDCl_3$) δ 7.62 – 7.56 (m, 2H), 7.53 – 7.49 (m, 2H), 7.42 (dd, *J* = 8.4, 6.9 Hz, 2H), 7.34 – 7.27 (m, 3H), 2.49 (q, J = 7.5 Hz, 1H), 1.37 (d, *J* = 7.5 Hz, 3H), 1.23 (d, *J* = 4.8 Hz, 12H); **^{13}C NMR** (151 MHz, $CDCl_3$) δ 144.3, 141.4, 138.1, 128.8, 128.3, 127.2, 127.1, 127.0, 83.5, 24.8, 24.8, 17.2 ppm. **Specific rotation** $[\alpha]_D^{21}$ = +8 (c = 0.24, CH_2Cl_2). **HRMS** (ESI) m/z calculated for $C_{22}H_{29}BO_2$ $[M+H]^+$, 337.2333; found, 337.2342. **IR (neat)** 2976,1486, 1275, 1142, 750, 698 cm^{-1}. The enantiomeric excess (ee) was determined after oxidation to alcohol. **HPLC conditions**: Chiral column IB, hexane: isopropanol = 90:10, flow rate = 1.0 mL/min, wavelength = 210 nm, t_R = 8.97 min for major isomer, t_R = 9.9 min for minor isomer.

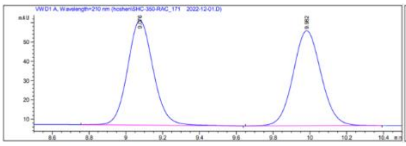

Peak #	RetTime [min]	Type	Width [min]	Area [mAU*s]	Height [mAU]	Area %
1	9.076	VB R	0.1500	525.33069	54.28094	50.1912
2	9.982	BV R	0.1654	521.32898	49.23934	49.8088

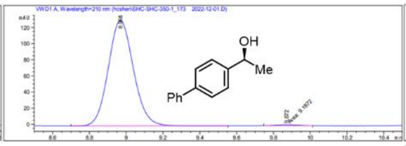

Peak #	RetTime [min]	Type	Width [min]	Area [mAU*s]	Height [mAU]	Area %
1	8.968	BV R	0.1455	1244.23438	130.91968	99.2670
2	9.872	MM	0.1453	9.18720	1.05408	0.7330

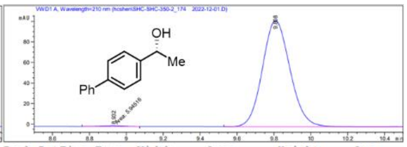

Peak #	RetTime [min]	Type	Width [min]	Area [mAU*s]	Height [mAU]	Area %
1	8.932	MM	0.1387	5.94516	7.14285e-1	0.5466
2	9.808	BV R	0.1646	1081.79797	103.44130	99.4534

(*S*)-4,4,5,5-tetramethyl-2-(1-(4-(trifluoromethyl)phenyl)ethyl)-1,3,2-dioxaborolane (28)

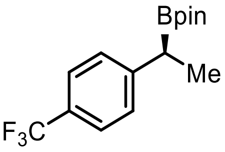

28

Prepared according to the referenced literature procedure.[5] **^{1}H NMR** (600 MHz, $CDCl_3$) δ 7.51 (d, *J* = 7.8 Hz, 2H), 7.32 (d, *J* = 8.6 Hz, 2H), 2.50 (q, *J* = 7.5 Hz, 1H), 1.35 (d, *J* = 7.5 Hz, 3H), 1.21 (d, *J* = 6.2 Hz, 12H) ppm; **^{13}C NMR** (151 MHz, $CDCl_3$) δ 149.4, 128.1, 127.5 (q, *J* = 32.3 Hz), 125.3 (q, *J* = 3.8 Hz), 124.7 (q, *J* = 271.7 Hz), 83.7, 24.8, 24.7, 16.9 ppm. **Specific rotation** $[\alpha]_D^{21}$ = +13 (c = 0.46, CH_2Cl_2). **HRMS** (ESI) m/z calculated for $C_{15}H_{21}BF_3O_2$ $[M+H]^+$, 301.1581; found, 301.1584. **IR (neat)** 2979, 2934, 1618, 1460, 1323, 1118, 1069, 1017, 847 cm^{-1}. The enantiomeric excess (ee) was determined after oxidation to alcohol. **HPLC conditions**: Chiral column OJ, hexane: isopropanol = 99:1, flow rate = 1.0 mL/min, wavelength = 210 nm, t_R = 45.6 min for major isomer, t_R = 39.5 min for minor isomer.

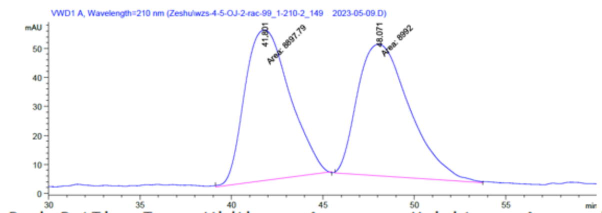

Peak #	RetTime [min]	Type	Width [min]	Area [mAU*s]	Height [mAU]	Area %
1	41.801	MM	2.8562	8897.79199	51.92065	49.7367
2	48.071	MM	3.3056	8992.00000	45.33740	50.2633

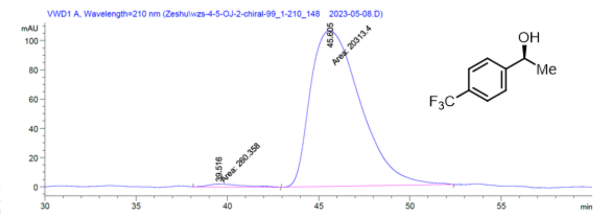

Peak #	RetTime [min]	Type	Width [min]	Area [mAU*s]	Height [mAU]	Area %
1	39.516	MM	2.1912	260.35846	1.98038	1.2655
2	45.605	MM	3.1808	2.03134e4	106.43853	98.7345

1-(5-(4,4,5,5-tetramethyl-1,3,2-dioxaborolan-2-yl)pentyl)-3,4-dihydroquinolin-2(1*H*)-one (S1)

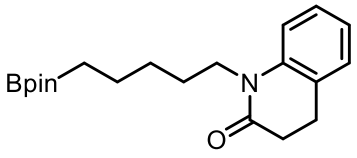

S1

Prepared according to the referenced literature procedure.[6] **^{1}H NMR** (400 MHz, $CDCl_3$) δ 7.21 (ddd, *J* = 8.2, 7.4, 1.6 Hz, 1H), 7.17 – 7.10 (m, 1H), 7.02 – 6.93 (m, 2H), 3.96 – 3.84 (m, 2H), 2.86 (dd, *J* = 8.7, 6.0 Hz, 2H), 2.65 – 2.57 (m, 2H), 1.69 – 1.57 (m, 2H), 1.50 – 1.30 (m, 4H), 1.22 (s, 12H), 0.77 (t, *J* = 7.6 Hz, 2H). **^{13}C NMR** (101 MHz, $CDCl_3$) δ 170.1, 139.7, 128.0, 127.5, 126.7, 122.7, 115.0, 83.0, 42.2, 32.1, 29.7, 27.1, 25.7, 24.9, 23.9. **HRMS** (ESI) m/z calculated for $C_{20}H_{30}BNO_3$ $[M+H]^+$, 344.2392; found, 344.2388. **IR (neat)** 2931, 1672, 1377, 1145, 753 cm^{-1}.

3-(4-(4,4,5,5-tetramethyl-1,3,2-dioxaborolan-2-yl)butyl)pyridine (S2)

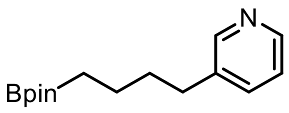

S2

Prepared according to the referenced literature procedure.[6] **^{1}H NMR** (400 MHz, $CDCl_3$) δ 8.48 – 8.36 (m, 2H), 7.54 – 7.44 (m, 1H), 7.19 (ddd, *J* = 7.8, 4.8, 0.9 Hz, 1H), 2.67 – 2.50 (m, 2H), 1.70 – 1.58 (m, 2H), 1.53 – 1.41 (m, 2H), 1.24 (s, 12H), 0.81 (t, *J* = 7.7 Hz, 2H). **^{13}C NMR** (101 MHz, $CDCl_3$) δ 150.1, 147.3, 138.1, 135.9, 123.4, 83.1, 33.9, 33.0, 25.0, 23.7.

HRMS (ESI) m/z calculated for $C_{15}H_{26}BNO_2$ $[M+H]^+$, 262.1973; found, 262.1970. **IR (neat)** 2930, 1575, 1371, 1144, 750 cm^{-1}.

2.4. Procedures for Transformation of Products

2.4.1. Procedure G: Hydrogenation of alkene

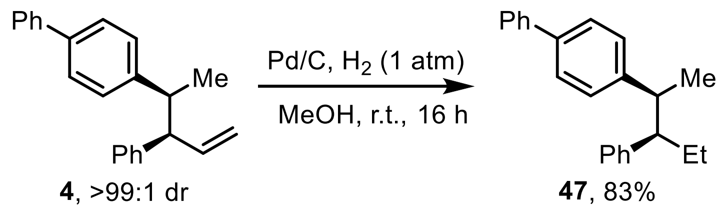

To the solution of the alkene **4** (29.8 mg, 0.1 mmol, 1.0 equiv, >99:1 dr) in MeOH (2.0 mL) in a 10-mL Schlenk tube was added 10% Pd/C (10.0 mg, 0.01 mmol). Then the vial was charged with 1 atm of H_2 by freeze-pump-thaw (FPT) method and the reaction mixture was stirred at room temperature for 16 h. Then the reaction mixture was filtered through a plug of silica gel. After removal of the solvent under reduced pressure, the residue was purified by column chromatography (0–1% EtOAc in *n*-hexane) to afford **47** as a white solid (24.8 mg, 83%, >99:1 dr)

2.4.2. Procedure H: Ozonation followed by reduction of alkene

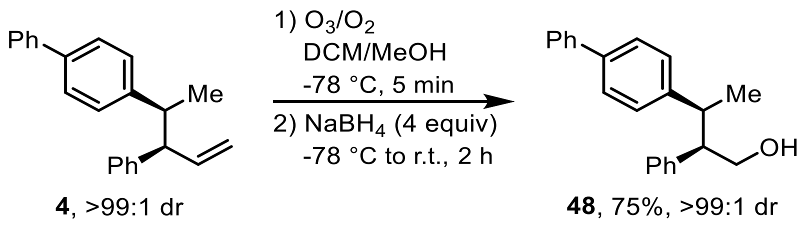

To the solution of the alkene **4** (29.8 mg, 0.1 mmol, 1.0 equiv, >99:1 dr) in CH_2Cl_2/MeOH (2.0 mL, 3:1) and cooled to –78 °C before a stream of O_3–$O_{2(g)}$ was gently bubbled through the solution for ~5 min. The reaction was then purged by bubbling a gentle stream of $N_{2(g)}$ through the solution for 20 min, which was followed by the portion wise addition of $NaBH_4$ (15.2 mg, 0.4 mmol, 4.0 equiv). The mixture was stirred at –78 °C for 15 min followed by 2 h at room temperature before quenching with brine (5 mL) and extracting with EtOAc (3 × 5 mL). The organic phases were combined, dried over $MgSO_4$, filtered, and concentrated under reduced

pressure to give a crude material that was then purified by column chromatography (0–30% EtOAc in *n*-hexane) to give alcohol **48** as a white solid (22.8 mg, 75%, >99:1 dr).

2.4.3. Procedure I: Olefin metathesis

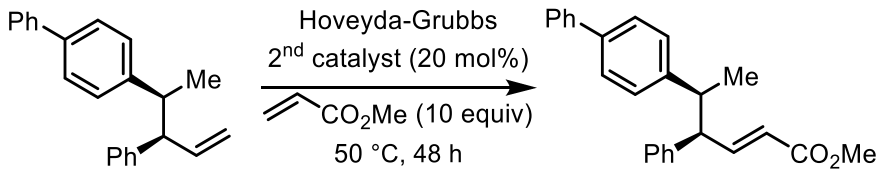

4, >99:1 dr　　**49**, 88%, >99:1 dr

In a glove box, to an oven-dried 7-mL vial equipped with a magnetic stir bar was added alkene **4** (29.8 mg, 0.1 mmol, 1.0 equiv, >99:1 dr), methyl acrylate (90 uL, 1.0 mmol, 10 equiv), Hoveyda-Grubbs 2nd catalyst (6.3 mg, 0.01 mmol, 10 mol%) and dichloromethane (1.0 mL). The vial was sealed with a black cap and removed from the glove box. Outside the glove box, the reaction was stirred at 50 °C for 24 hours. After that, the reaction was cooled down to room temperature. Second portion of Hoveyda-Grubbs 2nd catalyst (6.3 mg, 0.01 mmol, 10 mol%) was added to the reaction mixture in a glove box. The vial stirred at 50 °C for another 24 hours outside the glove box. The reaction mixture was filtered through a silica gel plug using diethyl ether as eluent and the filtrate was concentrated under reduced pressure. The crude product was purified by column chromatography (0–5% EtOAc in *n*-hexane) to give the title compound **49** (31.5 mg, 88%, >99:1 dr).

2.4.4. Procedure J: Asymmetric diboration of alkene

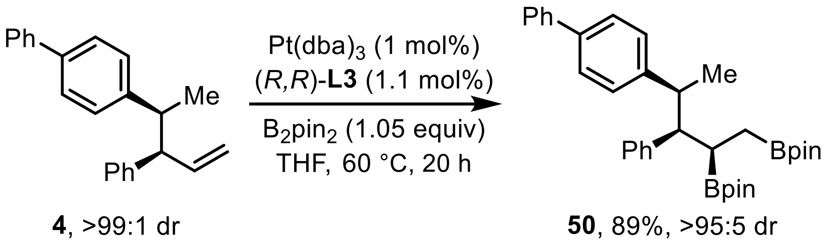

4, >99:1 dr　　**50**, 89%, >95:5 dr

The compound **50** was synthesized according to Morken's asymmetric diboration chemistry with modification.[15] To an oven-dried 7-mL vial with magnetic stir bar in the glove box was added $Pt(dba)_3$ (0.9 mg, 1.0 µmol, 1 mol %), (*R*, *R*)-**L3** (1.1 mg, 1.1 µmol, 1.1 mol %), $B_2(pin)_2$ (26.7 mg, 0.105 mmol, 1.05 equiv) and THF (1 mL). The vial was sealed with a black cap, removed from the glove box, and heated to 80 ºC in an oil bath for 30 minutes. The vial was

cooled to room temperature, returned to the glove box and charged with alkene **4** (29.8 mg, 0.1 mmol, 1.0 equiv, >99:1 dr). The vial was sealed, removed from the glove box and stirred at 60 ºC for 20 h. The reaction mixture was filter through a pad of silica gel and the filtrate was concentrated under reduced pressure. The crude product was purified by column chromatography (0–10% EtOAc in *n*-hexane) to give the title compound **50** (48.9 mg, 89%, >95:5 dr).

2.4.5. Procedure K: Asymmetric diboration of alkene

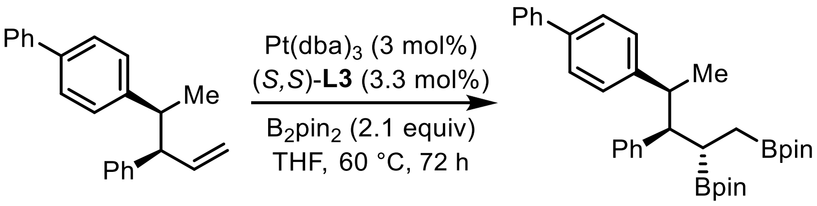

4, >99:1 dr   **51**, 78%, >95:5 dr

The compound **51** was synthesized according to above reaction condition with modification. To an oven-dried 7-mL vial with magnetic stir bar in the glove box was added $Pt(dba)_3$ (2.7 mg, 3.0 µmol, 3 mol %), (*S*, *S*)-**L3** (3.3 mg, 3.3 µmol, 3.3 mol %), $B_2(pin)_2$ (53.4 mg, 0.21 mmol, 2.1 equiv) and THF (1 mL). The vial was sealed with a black cap, removed from the glove box, and heated to 80 ºC in an oil bath for 30 minutes. The vial was cooled to room temperature, returned to the glove box and charged with alkene **4** (29.8 mg, 0.1 mmol, 1.0 equiv, >95:5 dr). The vial was sealed, removed from the glove box and stirred at 60 ºC for 72 h. The reaction mixture was filter through a pad of silica gel and the filtrate was concentrated under reduced pressure. The crude product was purified by column chromatography (0–10% EtOAc in *n*-hexane) to give the title compound **51** (42.9 mg, 78%, >95:5 dr).

2.5. Procedures for Scale Up Reaction, One-Pot Synthesis, and Radical Addition–Desulfonylation Sequence

2.5.1. Scale up reaction and kinetic resolution of racemic allylic carbonate

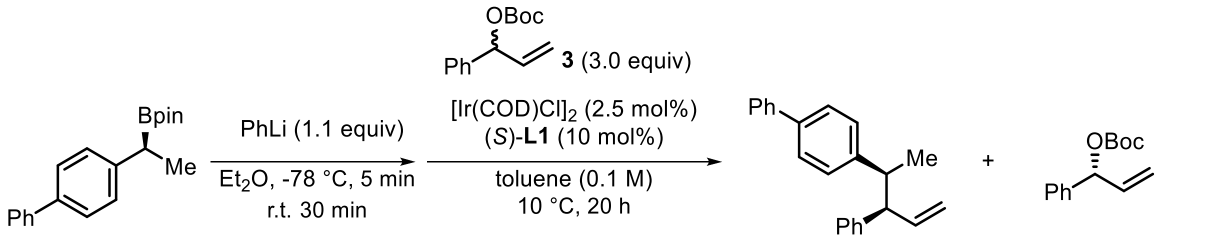

(*S*)-**1**
4 mmol, 99% ee
1.233 g

4, 90%, >99% ee
97:3 dr, 97:3 b:l
1.069 g

(*R*)-**3**: 0.77 equiv
26% yield, >99% ee

The scale up reaction was operated following **General Procedure A** with modifications. **Step 1 boronate complex generation:** To an oven-dried long type of 100 mL Schlenk tube equipped with a magnetic stir bar was back up filled with N_2 3 times, and then chiral (*S*)-**1** (4.0 mmol, 1.0 equiv, 1.23 g) and 20 mL anhydrous Et_2O was added under N_2. The solution was cooled to −78 °C (dry ice/acetone) followed by addition of PhLi (1.67 M in pentane, 2.63 mL, 4.4 mmol) dropwise. The mixture was allowed to stir for 10 min at −78 °C and then warmed to ambient temperature and further stirred for 30 min to form boronate complex completely. The solvent was removed under high vacuum and refilled with N_2. **Step 2 asymmetric allylation:** In glovebox, to an oven-dried 50 mL round-bottomed flask equipped with a magnetic stir bar was added $[Ir(cod)Cl]_2$ (0.1 mmol, 2.5 mol%, 67.2 mg) and (*S*)-**L1** (0.4 mmol, 10 mol%, 201.4 mg). The vial was sealed with a septum and removed from the glovebox, and then anhydrous toluene (20 mL) was added under N_2. The mixture was stirred for 30 min at ambient temperature and then added with allyl *tert*-butyl carbonate **3** (12 mmol, 3.0 equiv, 2.81 g) to form π-allyl iridium solution. The π-allyl iridium solution was then added to above boronate complex. The π-allyl iridium vial was washed with 20 mL toluene and then added to Schlenk tube. Finally, Schlenk tube was moved to 10 °C bath (cooled down with cryostat) and stirred for 20 h at this temperature. The crude reaction mixture was filtered through a pad of silica gel and filtrate was concentrated under reduced pressure. The crude material was purified by flash column chromatography (0–1% DCM in *n*-hexane) gave the title compound (1.069 g, 90%, >99% ee, 97:3 dr, 97:3 b:l) as a white solid. The unreacted allyl *tert*-butyl carbonate was purified by flash

column chromatography (0–10% EtOAc in *n*-hexane) gave the title compound (718.5 mg, >99% ee) as a colorless oil. [HPLC conditions of *tert*-butyl carbonate: Chiral column OJ, hexane: isopropanol = 99:1, flow rate = 1.0 mL/min, wavelength = 210 nm, t_R = 9.73 min for major isomer.].

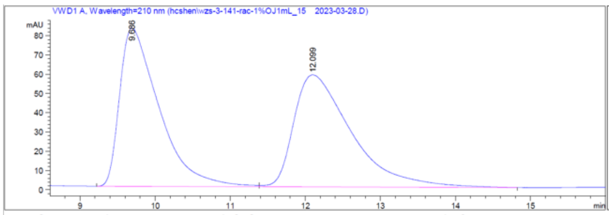

Peak #	RetTime [min]	Type	Width [min]	Area [mAU*s]	Height [mAU]	Area %
1	9.686	BV	0.4580	3000.11523	82.09584	49.1493
2	12.099	VV R	0.6347	3103.96973	58.25694	50.8507

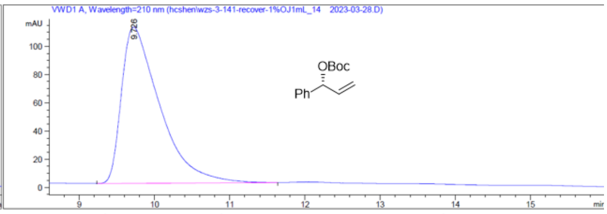

Peak #	RetTime [min]	Type	Width [min]	Area [mAU*s]	Height [mAU]	Area %
1	9.726	BV R	0.4459	3916.76685	110.70155	100.0000

2.5.2. One-pot synthesis

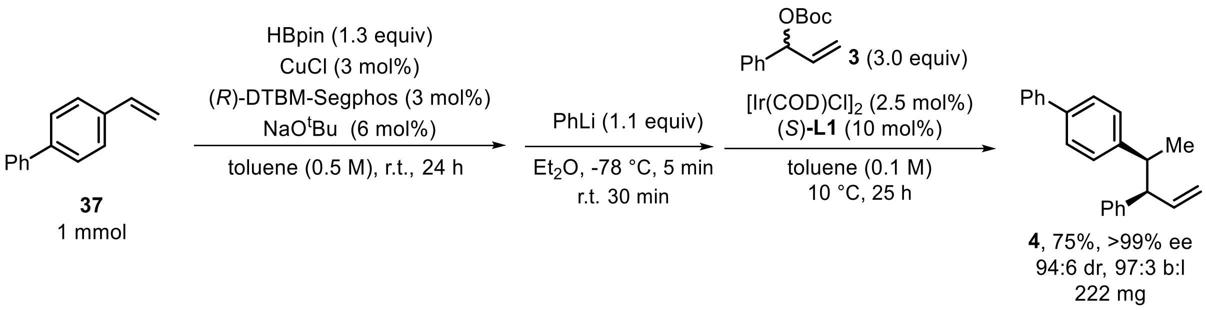

Step 1 Hrdroboronation: In glovebox, to an oven-dried 50 mL round-bottomed flask equipped with a magnetic stir bar was charged with CuCl (3.0 mg, 3 mol %), NaO*t*Bu (5.8 mg, 6 mol %), (*R*)-DTBM-SEGPHOS (35.4 mg, 3 mol %) and toluene (1 mL). The mixture was allowed to stir at ambient temperature for 10 minutes before the addition of pinacolborane (0.22 mL, 1.3 mmol, 1.3 equiv). After brief stirring (10 minutes), the solution was added the alkene (0.18 g, 1 mmol, 1 equiv) and 1 mL toluene. The vial was then capped, sealed with electrical tape, and removed from the box. After 24 h of stirring at rt, the solvent was removed under high vacuum and refilled with N_2. **Step 2 boronate complex generation:** To the reactor from **step 1**, 5 mL anhydrous Et_2O were added sequentially under N_2. The solution was cooled to −78 °C (dry ice/acetone) and followed by addition of PhLi (0.64 mL, 1.67 M, 1.1 equiv) dropwise. The mixture was allowed to stir for 5 min at −78 °C and then warmed to ambient temperature and further stirred for 30 min to form boronate complex completely. The solvent was removed under high vacuum and refilled with N_2. **Step 3 asymmetric allylation:** In

glovebox, to an oven-dried 50 mL round-bottomed flask equipped with a magnetic stir bar was added $[Ir(cod)Cl]_2$ (0.025 mmol, 2.5 mol%, 17.0 mg) and (*S*)-**L1** (0.01 mmol, 10 mol%, 50.5 mg). The vial was sealed with a septum and removed from the glovebox, and then anhydrous toluene (5 mL) was added under N_2. The mixture was stirred for 30 min at ambient temperature and then added with allyl *tert*-butyl carbonate (3 mmol, 3.0 equiv, 0.74 mL) to form π-allyl iridium solution. The π-allyl iridium solution was then added to above boronate complex. The π-allyl iridium vial was washed with 5 mL toluene and then added to Schlenk tube. Finally, Schlenk tube was moved to 10 °C bath (cooled down with cryostat) and stirred for 25 h at this temperature. The crude reaction mixture was filtered through a pad of silica gel and filtrate was concentrated under reduced pressure. The crude material was purified by flash column chromatography (3% DCM in *n*-hexane) gave the title compound (222.0 mg, 75%, >99% ee, 94:6 dr, 97:3 b:l) as a white solid.

2.5.3. Radical addition–desulfonylation sequence

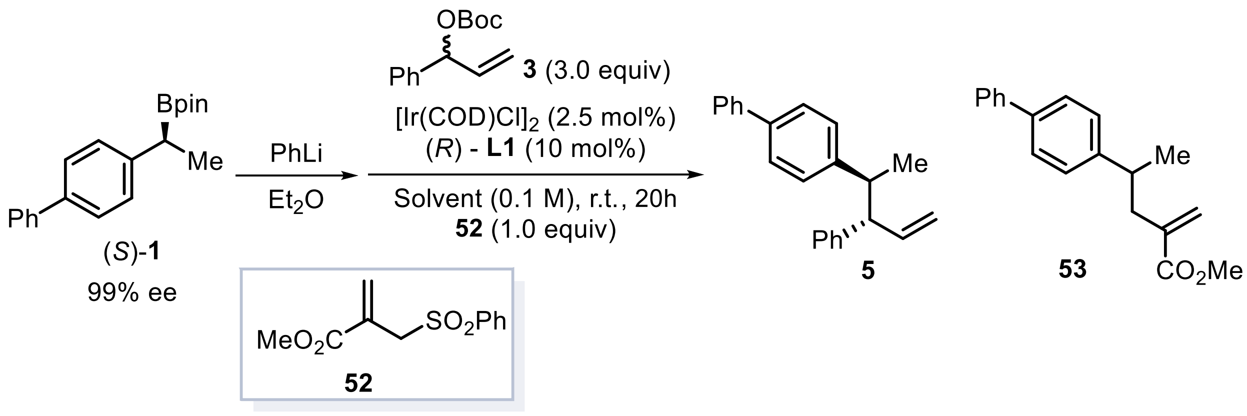

Step 1 boronate complex generation: To an oven-dried 10 mL Schlenk tube equipped with a magnetic stir bar was back up filled with N_2 3 times, and then chiral *sec*-boronic ester (0.2 mmol, 1.0 equiv) and 2 mL anhydrous Et_2O was added under N_2. The solution was cooled to −78 °C (dry ice/acetone) followed by addition of PhLi (1.67 M in pentane, 0.13 mL, 0.22 mmol) dropwise. The mixture was allowed to stir for 10 min at −78 °C and then warmed up to ambient temperature and further stirred for 30 min to form boronate complex completely. The solvent was removed under high vacuum and refilled with N_2. The methyl 2-((phenylsulfonyl)methyl)acrylate solid **52** (0.2 mmol, 1.0 equiv) was added in one portion, and

then Schlenk tube was back up filled with N_2 3 times. **Step 2 asymmetric allylation:** In glovebox, to an oven-dried 7.0 mL vial equipped with a magnetic stir bar was added $[Ir(cod)Cl]_2$ (0.005 mmol, 2.5 mol%, 3.4 mg) and (*R*)-**L1** (0.02 mmol, 10 mol%, 10.1 mg). The vial was sealed with a septum and removed from the glovebox, and then anhydrous toluene or THF (1.0 mL) was added under N_2. The mixture was stirred for 30 min at ambient temperature and then added with allyl *tert*-butyl carbonate (0.6 mmol, 3.0 equiv) to form π-allyl iridium solution (This should be prepared during forming boronate complex at ambient temperature). The π-allyl iridium solution was then added to above boronate complex. The π-allyl iridium vial was washed with 1.0 mL toluene or THF and then added to Schlenk tube. Finally, Schlenk tube was moved to 10 °C bath (cooled down with cryostat) and stirred for 20 h at this temperature. The crude reaction mixture was filtered through a pad of silica gel and filtrate was concentrated under reduced pressure. The crude material was purified by flash column chromatography (*n*-hexane/DCM = 5/1) to afford the desired product **5**, and (*n*-hexane/EA = 10/1) to afford the desired product **53**.

2.6. Unsuccessful Substrates.

R-Bpin	Conditions	Results	
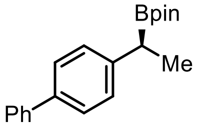<br>99% ee	**Li-1**, **3c**<br>5%**Ir**, 10% **L1**<br>Tol (0.1 M), 10 °C	with (*S*)-**L1**<br>trace<br>83:17 dr, 86:14 b:l	with (*R*)-**L1**<br>trace<br>26:74 dr, 82:18 b:l
	**Li-1**, **3d**<br>5%**Ir**, 10% **L1**<br>Tol (0.1 M), 10 °C	with (*S*)-**L1**<br>47%, (impure in NMR)<br>2.5:1 dr, 8:1 rr	with (*R*)-**L1**<br>90%, (impure in NMR)<br>1:2.3 dr, 2.8:1 rr
	**Li-1**, **3e**<br>5%**Ir**, 10% **L1**<br>Tol (0.1 M), 10 °C	with (*S*)-**L1**<br>64%, (impure in NMR)<br>13:13:37:37 (GC)	with (*R*)-**L1**<br>33%, (impure in NMR)<br>17:8:18:57 (GC)
	**Li-1**, **3f**<br>5%**Ir**, 10% **L1**<br>Tol (0.1 M), 10 °C	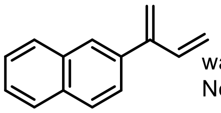 was observed<br>No desirted product	
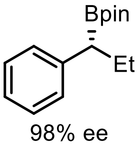<br>98% ee	**Li-1**, **3a**<br>5%**Ir**, 10% (*R*)-**L1**<br>THF (0.1 M), r.t.	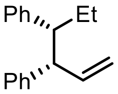<br>**S3**, 53%, 96% ee<br>89:11 dr, 79:21 b:l	
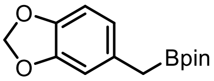	**Li-1**, **3b**<br>5%**Ir**, 10% (*S*)-**L1**<br>THF (0.1 M)<br>40 °C	PhLi: complex<br>Ar^FLi: complex	

Li-1: 3,5-$(CF_3)_2C_6H_3Li$

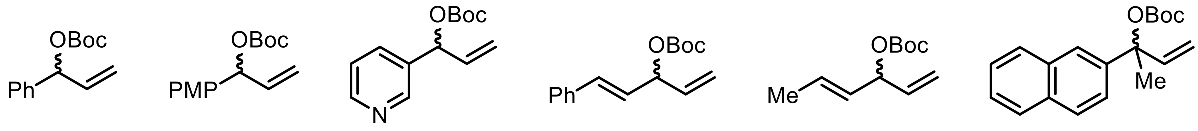

3a **3b** **3c** **3d** **3e** **3f**

2.7. Crystallography

X-ray diffraction experiments on (*S*)-**1**, **6** and **19** were carried out at 100(2) K on a Bruker D8 Venture using Cu-K_α (λ = 1.54178 Å) radiation. Intensities were integrated in SAINT[7] and absorption corrections based on equivalent reflections were applied using SADABS.[8] Structures (*S*)-**1** and **19** were solved using ShelXT[9] while **6** was solved using Superflip[10-11], all of the structures were refined by full matrix least squares against F^2 in ShelXL[12-13] using Olex2[14]. All of the non-hydrogen atoms were refined anisotropically. While all of the

hydrogen atoms were located geometrically and refined using a riding model. In the case of **6** the molecule displayed disorder, the occupancies of the fragments was determined by refining them against a free variable with the sum of the two sites set to equal 1. Restraints were used to maintain similar geometric parameters and constraints to maintain sensible thermal parameters. Crystal structure and refinement data are given in Table S5. In the case of **6** the absolute structure was not able to be determined. Crystallographic data for compounds (*S*)-**1**, **6** and **19** have been deposited with the Cambridge Crystallographic Data Centre as supplementary publication CCDC 2285737-2285739. Copies of the data can be obtained free of charge on application to CCDC, 12 Union Road, Cambridge CB2 1EZ, UK [fax(+44) 1223 336033, e-mail: deposit@ccdc.cam.ac.uk].

Table S8 Crystal data and structure refinement for(*S*)-1, 6 and 19.

Identification code	(*S*)-**1**	**6**	**19**
**CCDC**	**2285737**	**2285738**	**2285739**
Empirical formula	$C_{20}H_{25}BO_2$	$C_{24}H_{24}O$	$C_{32}H_{29}NO_2S$
Formula weight	308.21	328.43	491.62
Temperature/K	100(2)	100(2)	100(2)
Crystal system	orthorhombic	monoclinic	orthorhombic
Space group	$P2_12_12_1$	$P2_1$	$P2_12_12_1$
a/Å	7.3129(2)	7.5652(2)	10.2216(2)
b/Å	9.7826(3)	5.6069(2)	14.7528(3)
c/Å	24.2296(7)	21.3825(6)	17.1033(3)
α/°	90	90	90
β/°	90	93.2881(11)	90
γ/°	90	90	90
Volume/Å^3	1733.37(9)	905.50(5)	2579.13(9)
Z	4	2	4
ρ_{calc}g/cm^3	1.181	1.205	1.266
μ/mm^{-1}	0.569	0.547	1.341
F(000)	664.0	352.0	1040.0
Crystal size/mm^3	0.56 × 0.34 × 0.16	0.308 × 0.264 × 0.06	0.297 × 0.205 × 0.188
Radiation	CuKα (λ = 1.54178)	CuKα (λ = 1.54178)	CuKα (λ = 1.54178)
2θ range for data collection/°	9.75 to 144.158	4.14 to 144.194	7.914 to 145.846
Index ranges	$-9 \le h \le 9$, $-12 \le k \le 12$, $-29 \le l \le 28$	$-9 \le h \le 9$, $-6 \le k \le 5$, $-26 \le l \le 26$	$-12 \le h \le 12$, $-16 \le k \le 18$, $-21 \le l \le 21$
Reflections collected	31857	32402	56197
Independent reflections	3397 [R_{int} = 0.0265, R_{sigma} = 0.0153]	3373 [R_{int} = 0.0628, R_{sigma} = 0.0348]	5125 [R_{int} = 0.0893, R_{sigma} = 0.0383]
Data/restraints/parameters	3397/0/213	3373/6/235	5125/0/327
Goodness-of-fit on F^2	1.089	1.123	1.077
Final R indexes [I>=2σ (I)]	R_1 = 0.0284, wR_2 = 0.0714	R_1 = 0.0682, wR_2 = 0.2314	R_1 = 0.0563, wR_2 = 0.1611
Final R indexes [all data]	R_1 = 0.0286, wR_2 = 0.0716	R_1 = 0.0689, wR_2 = 0.2335	R_1 = 0.0572, wR_2 = 0.1628
Largest diff. peak/hole / e Å^{-3}	0.17/-0.21	0.32/-0.33	0.60/-0.99
Flack parameter	0.02(4)	Absolute structure not determined	-0.010(15)

2.8. Characterization Data for Products.

4-((2*S*,3*R*)-3-phenylpent-4-en-2-yl)-1,1'-biphenyl (4)

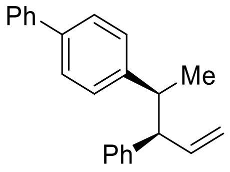

4

Prepared following **General Procedure A** using (*S*)-**1** and (*S*)-**L1**. Purification by flash column chromatography (0–1% DCM in *n*-hexane) gave the title compound (52.7 mg, 88%, >99% ee, 94.5:5.5 dr, 97.5:2.5 b:l) as a white solid.

^{1}H NMR (500 MHz, $CDCl_3$) δ 7.64 – 7.59 (m, 2H), 7.54 (d, *J* = 8.2 Hz, 2H), 7.43 (dd, *J* = 8.4, 7.0 Hz, 2H), 7.33 (td, *J* = 7.2, 1.1 Hz, 3H), 7.28 – 7.19 (m, 5H), 5.89 (ddd, *J* = 17.0, 10.3, 8.0 Hz, 1H), 4.87 (dt, *J* = 10.3, 1.3 Hz, 1H), 4.78 (dt, *J* = 17.0, 1.4 Hz, 1H), 3.48 – 3.42 (m, 1H), 3.17 – 3.07 (m, 1H), 1.13 (d, *J* = 6.9 Hz, 3H) ppm. **^{13}C NMR** (126 MHz, $CDCl_3$) δ 144.8, 143.4, 141.2, 140.7, 139.1, 128.8, 128.6, 128.5, 128.4, 127.2, 127.1, 127.0, 126.5, 115.7, 57.5, 45.2, 20.9 ppm. **Specific rotation** $[\alpha]_D^{24}$ = +41 (c = 2.0, CH_2Cl_2). **HRMS** (EI) m/z calculated for $C_{23}H_{22}$ $[M]^+$, 298.1716, found: 298.1710. **IR (neat)** 3028, 2923, 1597, 1486, 1459, 1409, 902, 841, 760 cm^{-1}.

The enantiomeric excess (ee) was determined after hydroboration/oxidation to alcohol **38**.

(3*R*,4*S*)-4-([1,1'-biphenyl]-4-yl)-3-phenylpentan-1-ol (46)

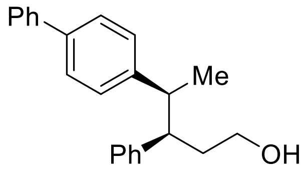

46

Prepared following **General Procedure F** (0.1 mmol **4**, 94.5:5.5 dr, 97.5:2.5 b:l). Purification by flash column chromatography (0–30% EtOAc in *n*-hexane) gave the title compound (28.6 mg, 98%, >99% ee) based on major isomer as a white solid.

^{1}H NMR (400 MHz, $CDCl_3$) δ 7.65 – 7.60 (m, 2H), 7.59 – 7.55 (m, 2H), 7.49 – 7.42 (m, 2H), 7.39 – 7.29 (m, 5H), 7.29 – 7.22 (m, 3H), 3.41 – 3.20 (m, 2H), 2.99 – 2.80 (m, 2H), 1.82 – 1.63 (m, 2H), 1.04 (d, *J* = 6.7 Hz, 3H), 0.97 (t, *J* = 5.3 Hz, 1H) ppm. **^{13}C NMR** (101 MHz, $CDCl_3$) δ 145.6, 143.7, 141.2, 139.3, 128.9, 128.7, 128.4, 128.1, 127.3, 127.2, 127.1, 126.6, 61.5, 49.9, 46.2, 37.6, 21.2 ppm. **Specific rotation** $[\alpha]_D^{23}$ = −3 (c = 3.0, CH_2Cl_2). **HRMS** (APCI) m/z calculated for $C_{23}H_{22}$ $[M+H-H_2O]^+$, 299.1794, found: 299.1800. **IR (neat)** 3338, 3026, 2953, 2924, 1600, 1486, 1451, 1029, 839, 763, 732, 697 cm^{-1}. **HPLC conditions**: Chiral column IB, hexane: isopropanol = 95:5, flow rate = 1.0 mL/min, wavelength = 210 nm, t_R = 11.3 min for minor isomer, t_R = 14.8 min for major isomer.

4-((2*S*,3*S*)-3-phenylpent-4-en-2-yl)-1,1'-biphenyl (5)

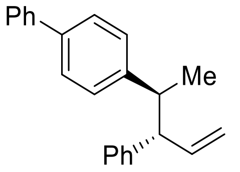

5

Prepared following **General Procedure A** using (*S*)-**1** and (*R*)-**L1**. Purification by flash column chromatography (0–1% DCM in *n*-hexane) gave the title compound (55.1 mg, 92%, >99% ee, 7.5:92.5 dr, 94.5:5.5 b:l) as a white solid.

^{1}H NMR (500 MHz, $CDCl_3$) δ 7.56 – 7.52 (m, 2H), 7.42 – 7.37 (m, 4H), 7.32 – 7.28 (m, 1H), 7.18 – 7.13 (m, 2H), 7.11 – 7.02 (m, 5H), 6.12 (ddd, *J* = 16.9, 10.1, 9.2 Hz, 1H), 5.16 – 5.08 (m, 2H), 3.48 – 3.42 (m, 1H), 3.18 – 3.08 (m, 1H), 1.37 (d, *J* = 7.0 Hz, 3H) ppm. **^{13}C NMR** (126 MHz, $CDCl_3$) δ 144.6, 143.5, 141.1, 140.7, 138.6, 128.8, 128.3, 128.2(4), 128.1(7), 127.1, 127.0, 126.7, 126.1, 115.8, 58.3, 44.8, 20.4 ppm. **Specific rotation** $[\alpha]_D^{24}$ = +4 (c = 1.0, CH_2Cl_2). **HRMS** (EI) m/z calculated for $C_{23}H_{22}$ $[M]^+$, 298.1716, found: 298.1710. **IR (neat)** 3059, 3027, 2925, 1600, 1486, 1451, 1261, 1008, 910, 765, 697 cm^{-1}.

The enantiomeric excess (ee) was determined after hydroboration/oxidation to alcohol **5'**.

(3*S*,4*S*)-4-([1,1'-biphenyl]-4-yl)-3-phenylpentan-1-ol (5')

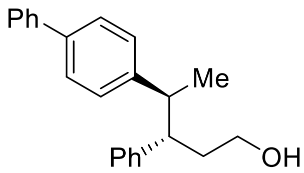

5'

Prepared following **General Procedure F (**0.1 mmol **5**, 7.5:92.5 dr, 94.5:5.5 b:l). Purification by flash column chromatography (0–30% EtOAc in *n*-hexane) gave the title compound (27.1 mg, 98%, >99% ee) based on major isomer as a pale yellow oil.

^{1}H NMR (500 MHz, $CDCl_3$) δ 7.57 – 7.52 (m, 2H), 7.44 – 7.37 (m, 4H), 7.34 – 7.27 (m, 1H), 7.21 – 7.09 (m, 3H), 7.08 – 7.02 (m, 2H), 7.02 – 6.97 (m, 2H), 3.57 – 3.46 (m, 1H), 3.45 – 3.36 (m, 1H), 3.10 – 3.02 (m, 1H), 3.02 – 2.95 (m, 1H), 2.21 – 2.10 (m, 1H), 1.99 – 1.87 (m, 1H), 1.36 (d, *J* = 7.0 Hz, 3H), 1.11 (s, 1H) ppm. **^{13}C NMR** (126 MHz, $CDCl_3$) δ 144.4, 142.6, 141.1, 138.7, 128.8(4), 128.8(0), 128.6, 128.1, 127.1, 127.0, 126.6, 126.3, 61.6, 49.3, 45.4, 35.2, 18.9 ppm. **Specific rotation** $[\alpha]_D^{23}$ = +34 (c = 1.0, CH_2Cl_2). **HRMS** (APCI) m/z calculated for $C_{23}H_{22}$ $[M+H–H_2O]^+$, 299.1794, found: 299.1793. **IR (neat)** 3344, 3026, 2929, 1600, 1486, 1452, 1057, 837, 765, 733, 698 cm^{-1}. **HPLC conditions**: Chiral column IB, hexane: isopropanol = 95:5, flow rate = 1.0 mL/min, wavelength = 210 nm, t_R = 12.6 min for major isomer.

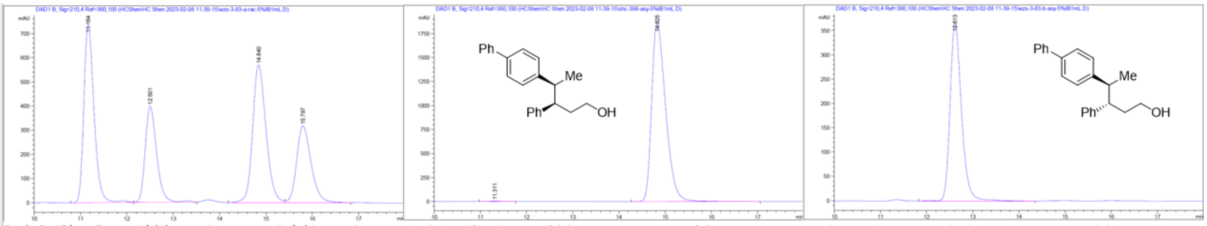

Peak #	RetTime [min]	Type	Width [min]	Area [mAU*s]	Height [mAU]	Area %
1	11.164	BV R	0.2323	1.12531e4	737.29468	30.9910
2	12.501	BV R	0.2594	6794.98535	397.46906	18.7134
3	14.840	BV	0.3078	1.14070e4	569.27643	31.4149
4	15.797	VB	0.3322	6855.73877	317.18970	18.8807

Peak #	RetTime [min]	Type	Width [min]	Area [mAU*s]	Height [mAU]	Area %
1	11.311	BB	0.2293	61.73697	4.20592	0.1578
2	14.825	BB	0.3256	3.90591e4	1855.32703	99.8422

Peak #	RetTime [min]	Type	Width [min]	Area [mAU*s]	Height [mAU]	Area %
1	12.613	VB R	0.2658	6334.41992	365.50980	100.0000

4-((2*S*,3*R*)-3-(4-methoxyphenyl)pent-4-en-2-yl)-1,1'-biphenyl (6)

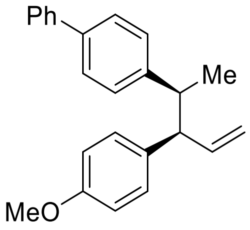

6

Prepared following **General Procedure A** using (*S*)-**1** and (*S*)-**L1**. Purification by flash column chromatography (0–20% DCM in *n*-hexane) gave the title compound (51.6 mg, 75%, >99% ee, 95.5:4.5 dr, 97.5:2.5 b:l) as a white solid. Note: PhBpin was challenging to remove via column chromatography. However, an effective method involved mixing it with $MeB(OH)_2$ in a 5% TFA/DCM solution, followed by subsequent flash column chromatography (0–20% DCM in *n*-hexane).[16]

^{1}H NMR (400 MHz, $CDCl_3$) δ 7.64 – 7.58 (m, 2H), 7.56 – 7.51 (m, 2H), 7.46 – 7.40 (m, 2H), 7.36 – 7.30 (m, 1H), 7.26 – 7.21 (m, 2H), 7.16 – 7.09 (m, 2H), 6.91 – 6.85 (m, 2H), 5.87 (ddd, *J* = 17.0, 10.3, 7.9 Hz, 1H), 4.90 – 4.83 (m, 1H), 4.80 – 4.74 (m, 1H), 3.82 (s, 3H), 3.46 – 3.37 (m, 1H), 3.12 – 3.00 (m, 1H), 1.13 (d, *J* = 7.0 Hz, 3H) ppm. **^{13}C NMR** (101 MHz, $CDCl_3$) δ 158.2, 144.9, 141.2, 141.0, 139.0, 135.4, 129.3, 128.8, 128.5, 127.2, 127.1, 127.0, 115.4, 114.0, 56.5, 55.4, 45.4, 20.8 ppm. **Specific rotation** $[\alpha]_D^{24}$ = +45 (c = 2.0, CH_2Cl_2). **HRMS** (EI) m/z calculated for $C_{24}H_{24}O$ $[M]^+$, 328.1822, found: 328.1817. **IR (neat)** 3005, 2953, 1614, 1514, 1260, 1177, 1033, 763, 750 cm^{-1}. **HPLC conditions**: Chiral column IB, hexane: isopropanol = 99:1, flow rate = 1.0 mL/min, wavelength = 254 nm, t_R = 5.2 min for major enantiomer, t_R = 5.7 min for minor diastereomer, 6.5 min for minor diastereomer.

4-((2*S*,3*S*)-3-(4-methoxyphenyl)pent-4-en-2-yl)-1,1'-biphenyl (7)

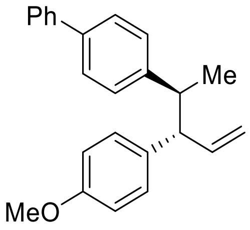

7

Prepared following **General Procedure A** using (*S*)-**1** and (*R*)-**L1**. Purification by flash column chromatography (0–20% DCM in *n*-hexane) gave the title compound (59.1 mg, 90%, 99% ee, 5.5:94.5 dr, 95:5 b:l) as a white solid. Note: PhBpin was challenging to remove via column chromatography. However, an effective method involved mixing it with $MeB(OH)_2$ in a 5% TFA/DCM solution, followed by subsequent flash column chromatography (0–20% DCM in *n*-hexane).[16]

^{1}H NMR (500 MHz, $CDCl_3$) δ 7.60 – 7.55 (m, 2H), 7.46 – 7.40 (m, 4H), 7.36 – 7.31 (m, 1H), 7.12 (d, J = 8.3 Hz, 2H), 7.00 – 6.95 (m, 2H), 6.76 – 6.71 (m, 2H), 6.12 (ddd, J = 16.9, 10.2, 9.2 Hz, 1H), 5.18 – 5.08 (m, 2H), 3.74 (s, 3H), 3.49 – 3.40 (m, 1H), 3.17 – 3.07 (m, 1H), 1.39 (d, J = 7.0 Hz, 3H) ppm. **^{13}C NMR** (126 MHz, $CDCl_3$) δ 157.8, 144.7, 141.1, 141.0, 138.6, 135.6, 129.0, 128.8, 128.3, 127.0(4), 127.0(0), 126.7, 115.4, 113.6, 57.3, 55.2, 44.8, 20.4 ppm. **Specific rotation** $[\alpha]_D^{24}$ = +10 (c = 2.0, CH_2Cl_2). **HRMS** (EI) m/z calculated for $C_{24}H_{24}O$ $[M]^+$, 328.1822, found: 328.1815. **IR (neat)** 3027, 2961, 2928, 1609, 1510, 1486, 1243, 1177, 1035, 827, 764, 750 cm^{-1}. **HPLC conditions**: Chiral column IB, hexane: isopropanol = 99:1, flow rate = 1.0 mL/min, wavelength = 254 nm, t_R = 5.3 min for minor diastereomer, t_R = 5.7 min for major enantiomer, 6.0 min for minor diastereomer, 6.5 min for minor enantiomer.

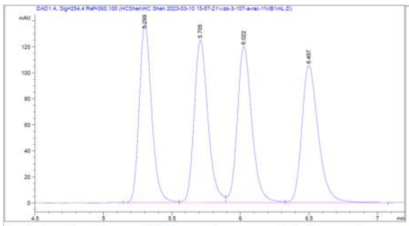

Peak #	RetTime [min]	Type	Width [min]	Area [mAU*s]	Height [mAU]	Area %
1	5.299	BV	0.0975	868.24103	137.74133	24.7484
2	5.705	VV	0.1075	874.14655	125.18552	24.9168
3	6.022	VV	0.1120	885.89673	120.20620	25.2517
4	6.497	VB	0.1273	879.98151	105.54084	25.0831

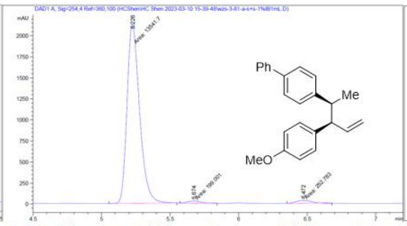

Peak #	RetTime [min]	Type	Width [min]	Area [mAU*s]	Height [mAU]	Area %
1	5.226	MM	0.1056	1.35417e4	2136.50342	96.7715
2	5.674	MM	0.1198	199.00133	27.68651	1.4221
3	6.472	MM	0.1205	252.78296	34.95523	1.8064

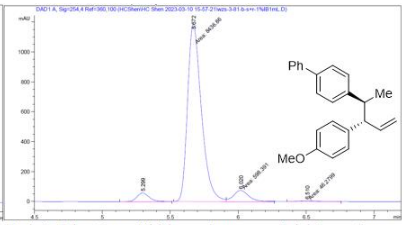

Peak #	RetTime [min]	Type	Width [min]	Area [mAU*s]	Height [mAU]	Area %
1	5.299	BV	0.0968	358.93320	57.51847	3.8021
2	5.672	MM	0.1183	8436.85840	1188.24011	89.3691
3	6.020	MM	0.1321	598.39136	75.49942	6.3386
4	6.510	MM	0.1566	46.27995	4.92565	0.4902

4-((2*S*,3*R*)-3-(4-bromophenyl)pent-4-en-2-yl)-1,1'-biphenyl (8)

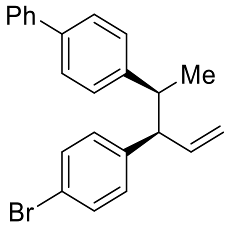

8

Prepared following **General Procedure A** using (*S*)-**1** and (*S*)-**L1**. Purification by flash column chromatography (0–10% DCM in *n*-hexane) gave the title compound (61.9 mg, 82%, >99% ee, 96:4 dr, 97:3 b:l) as a white solid.

^{1}H NMR (500 MHz, $CDCl_3$) δ 7.64 – 7.58 (m, 2H), 7.57 – 7.52 (m, 2H), 7.48 – 7.41 (m, 4H), 7.36 – 7.32 (m, 1H), 7.22 (d, *J* = 8.2 Hz, 2H), 7.08 (d, *J* = 8.4 Hz, 2H), 5.85 (ddd, *J* = 17.1, 10.3, 7.8 Hz, 1H), 4.90 (dt, *J* = 10.3, 1.3 Hz, 1H), 4.79 (dt, *J* = 17.1, 1.4 Hz, 1H), 3.47 – 3.40 (m, 1H), 3.13 – 3.03 (m, 1H), 1.13 (d, *J* = 6.9 Hz, 3H) ppm. **^{13}C NMR** (126 MHz, $CDCl_3$) δ 144.2, 142.3, 141.1, 140.0, 139.2, 131.7, 130.2, 128.9, 128.5, 127.2, 127.1, 127.0, 120.2, 116.2, 56.8, 45.1, 20.7 ppm. **Specific rotation** $[\alpha]_D^{24}$ = +41 (c = 2.0, CH_2Cl_2). **HRMS** (EI) m/z calculated for $C_{23}H_{21}Br$ $[M]^+$, 376.0821, found: 376.0813. **IR (neat)** 3026, 2971, 2925, 1609, 1486, 1261, 1008, 827, 764, 750 cm^{-1}.

The enantiomeric excess (ee) was determined after hydroboration/oxidation to alcohol **8'**.

(3*R*,4*S*)-4-([1,1'-biphenyl]-4-yl)-3-(4-bromophenyl)pentan-1-ol (8')

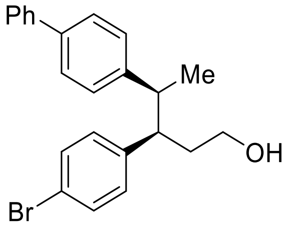

8'

Prepared following **General Procedure F (**0.1 mmol **8**, 96:4 dr, 97:3 b:l). Purification by flash column chromatography (0–30% EtOAc in *n*-hexane) gave the title compound (30.2 mg, 82%, >99% ee) based on major isomer as a white solid.

^{1}H NMR (400 MHz, $CDCl_3$) δ 7.63 – 7.58 (m, 2H), 7.58 – 7.52 (m, 2H), 7.50 – 7.40 (m, 4H), 7.38 – 7.31 (m, 1H), 7.30 – 7.26 (m, 2H), 7.15 – 7.08 (m, 2H), 3.42 – 3.30 (m, 1H), 3.29 – 3.16 (m, 1H), 2.95 – 2.80 (m, 2H), 1.82 – 1.71 (m, 1H), 1.71 – 1.59 (m, 1H), 1.03 (d, *J* = 6.4 Hz, 3H), 0.95 (t, *J* = 5.3 Hz, 1H) ppm. **^{13}C NMR** (126 MHz, $CDCl_3$) δ 145.1, 142.7, 141.1, 139.4, 131.7, 130.2, 128.9, 128.1, 127.4, 127.3, 127.1, 120.3, 61.1, 49.2, 46.0, 37.4, 21.0 ppm. **Specific rotation** $[\alpha]_D^{23}$ = –4 (c = 2.0, CH_2Cl_2). **HRMS** (APCI) m/z calculated for $C_{23}H_{22}Br$ $[M+H-H_2O]^+$, 377.0899, found: 377.0921. **IR (neat)** 3322, 3026, 2926, 1501, 1486, 1451, 1406, 1072, 1039, 1009, 838, 769, 734, 697. **HPLC conditions**: Chiral column IB, hexane: isopropanol = 95:5, flow rate = 1.0 mL/min, wavelength = 210 nm, t_R = 12.0 min for major isomer.

4-((2*S*,3*S*)-3-(4-bromophenyl)pent-4-en-2-yl)-1,1'-biphenyl (9)

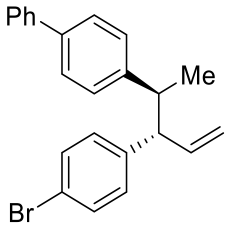

9

Prepared following **General Procedure A** using (*S*)-**1** and (*R*)-**L1**. Purification by flash column chromatography (0–1% DCM in *n*-hexane) gave the title compound (60.4 mg, 80%, >99% ee, 6:94 dr, 93:7 b:l) as a white solid.

^{1}H NMR (500 MHz, $CDCl_3$) δ 7.57 – 7.53 (m, 2H), 7.44 – 7.38 (m, 4H), 7.33 – 7.29 (m, 1H), 7.29 – 7.26 (m, 2H), 7.07 (d, *J* = 8.2 Hz, 2H), 6.93 – 6.88 (m, 2H), 6.11 – 6.00 (m, 1H), 5.16 – 5.08 (m, 2H), 3.45 – 3.38 (m, 1H), 3.13 – 3.03 (m, 1H), 1.36 (d, *J* = 7.0 Hz, 3H) ppm. **^{13}C NMR** (126 MHz, $CDCl_3$) δ 144.2, 142.5, 141.0, 140.2, 138.9, 131.3, 129.9, 128.8, 128.2, 127.2, 127.0, 126.8, 119.8, 116.2, 57.6, 44.7, 20.5 ppm. **Specific rotation** $[\alpha]_D^{24}$ = +30 (c = 2.0, CH_2Cl_2). **HRMS** (EI) m/z calculated for $C_{23}H_{21}Br$ $[M]^+$, 376.0821, found: 376.0814. **IR (neat)** 3026, 2965, 1636, 1600, 1486, 1402, 1073, 1009, 914, 833, 764, 733, 696 cm^{-1}.

The enantiomeric excess (ee) was determined after hydroboration/oxidation to alcohol **9'**.

(3*S*,4*S*)-4-([1,1'-biphenyl]-4-yl)-3-(4-bromophenyl)pentan-1-ol (9')

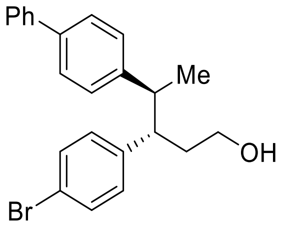

9'

Prepared following **General Procedure F (**0.1 mmol **9**, 6:94 dr, 93:7 b:l). Purification by flash column chromatography (0–30% EtOAc in *n*-hexane) gave the title compound (32.1 mg, 93%, >99% ee) based on major isomer as a colorless oil.

^{1}H NMR (400 MHz, $CDCl_3$) δ 7.59 – 7.51 (m, 2H), 7.45 – 7.36 (m, 4H), 7.34 – 7.27 (m, 3H), 7.05 – 6.97 (m, 2H), 6.88 – 6.80 (m, 2H), 3.57 – 3.46 (m, 1H), 3.42 – 3.30 (m, 1H), 3.07 – 2.91 (m, 2H), 2.23 – 2.10 (m, 1H), 1.91 – 1.78 (m, 1H), 1.35 (d, *J* = 6.7 Hz, 3H), 1.11 (t, *J* = 5.1 Hz, 1H) ppm. **^{13}C NMR** (126 MHz, $CDCl_3$) δ 143.9, 141.6, 141.0, 138.9, 131.2, 130.6, 128.8, 128.6, 127.2, 127.1, 126.7, 120.0, 61.2, 48.6, 45.3, 35.5, 19.2 ppm. **Specific rotation** $[\alpha]_D^{23}$ = +32 (c = 2.0, CH_2Cl_2). **HRMS** (APCI) m/z calculated for $C_{23}H_{22}Br$ $[M+H-H_2O]^+$, 377.0899, found: 377.0917. **IR (neat)** 3328, 3027, 2928, 2876, 1600, 1486, 1450, 1407, 1073, 1008, 836, 767, 735, 697 cm^{-1}. **HPLC conditions**: Chiral column IB, hexane: isopropanol = 95:5, flow rate = 1.0 mL/min, wavelength = 210 nm, t_R = 15.0 min for major isomer.

4-((2*R*,3*R*)-3-(4-bromophenyl)pent-4-en-2-yl)-1,1'-biphenyl (43)

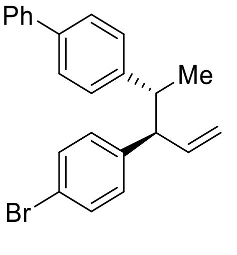

43

Prepared following **General Procedure A** using (*R*)-**1** and (*S*)-**L1**. Purification by flash column chromatography (0–1% DCM in *n*-hexane) gave the title compound (64.1 mg, 85%, >99% ee, 6.5:93.5 dr, 93:7 b:l) as a white solid.

Specific rotation $[\alpha]_D^{24} = -30$ (c = 2.0, CH_2Cl_2). **HRMS** (EI) m/z calculated for $C_{23}H_{21}Br$ $[M]^+$, 376.0821, found: 376.0817. **IR (neat)** 3026, 2964, 2925, 1636, 1600, 1486, 1402, 1073, 1009, 913, 833, 764, 732, 696 cm^{-1}.

The enantiomeric excess (ee) was determined after hydroboration/oxidation to alcohol **43'**.

(3*R*,4*R*)-4-([1,1'-biphenyl]-4-yl)-3-(4-bromophenyl)pentan-1-ol (43')

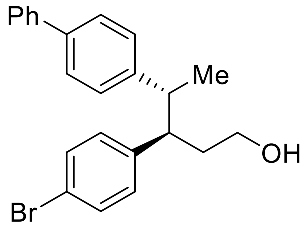

43'

Prepared following **General Procedure F (**0.1 mmol **43**, 6.5:93.5 dr, 93:7 b:l). Purification by flash column chromatography (0–30% EtOAc in *n*-hexane) gave the title compound (26.5 mg, 77%, >99% ee) based on major isomer as a colorless oil.

Specific rotation $[\alpha]_D^{23} = -34$ (c = 2.0, CH_2Cl_2). **HRMS** (APCI) m/z calculated for $C_{23}H_{22}Br$ $[M+H-H_2O]^+$, 377.0899, found: 377.0924. **IR (neat)** 3346, 3027, 2962, 1486, 1407, 1073, 1039, 1009, 836, 767, 735, 697 cm^{-1}. **HPLC conditions**: Chiral column IB, hexane: isopropanol = 95:5, flow rate = 1.0 mL/min, wavelength = 210 nm, t_R = 16.0 min for major isomer.

4-((2*R*,3*S*)-3-(4-bromophenyl)pent-4-en-2-yl)-1,1'-biphenyl (44)

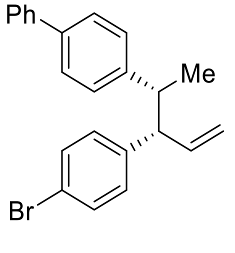

44

Prepared following **General Procedure A** using (*R*)-**1** and (*R*)-**L1**. Purification by flash column chromatography (0–10% DCM in *n*-hexane) gave the title compound (57.4 mg, 76%, >99% ee,

95:5 dr, 97:3 b:l) as a white solid.

Specific rotation $[\alpha]_D^{24}$ = −49 (c = 2.0, CH_2Cl_2). **HRMS** (EI) m/z calculated for $C_{23}H_{21}Br$ $[M]^+$, 376.0821, found: 376.0817. **IR (neat)** 3028, 2972, 2923, 1638, 1599, 1486, 1401, 1073, 1008, 903, 828, 763, 729, 693 cm^{-1}.

The enantiomeric excess (ee) was determined after hydroboration/oxidation to alcohol **44'**.

(3*S*,4*S*)-4-([1,1'-biphenyl]-4-yl)-3-(4-bromophenyl)pentan-1-ol (44')

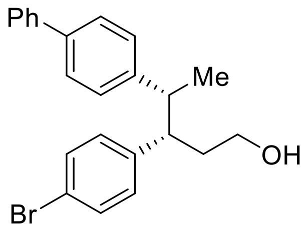

44'

Prepared following **General Procedure F (**0.1 mmol **44**, 95:5 dr, 97:3 b:l). Purification by flash column chromatography (0–30% EtOAc in *n*-hexane) gave the title compound (26.2 mg, 72%, >99% ee) based on major isomer as a white solid.

Specific rotation $[\alpha]_D^{23}$ = +5 (c = 2.0, CH_2Cl_2). **HRMS** (APCI) m/z calculated for $C_{23}H_{22}Br$ $[M+H-H_2O]^+$, 377.0899, found: 377.0912. **IR (neat)** 3324, 3026, 2923, 1601, 1486, 1450, 1406, 1072, 1040, 1008, 837, 767, 733, 697 cm^{-1}. **HPLC conditions**: Chiral column IC, hexane: isopropanol = 97:3, flow rate = 0.8 mL/min, wavelength = 254 nm, t_R = 13.0 min for major isomer, t_R = 11.9 min for minor isomer.

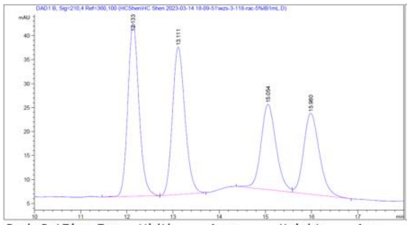

Peak #	RetTime [min]	Type	Width [min]	Area [mAU*s]	Height [mAU]	Area %
1	12.133	BB	0.2581	605.95905	36.04464	30.5191
2	13.111	BB	0.2891	576.43439	30.69910	29.0321
3	15.054	BV	0.3490	403.95529	17.78340	20.3452
4	15.980	VB	0.3635	399.15979	16.90289	20.1037

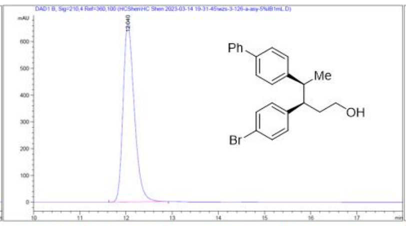

Peak #	RetTime [min]	Type	Width [min]	Area [mAU*s]	Height [mAU]	Area %
1	12.040	BB	0.2597	1.12617e4	664.56085	100.0000

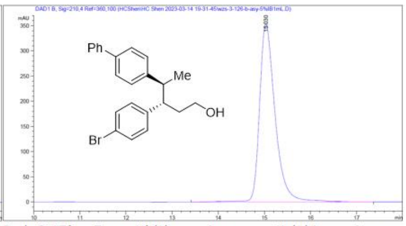

Peak #	RetTime [min]	Type	Width [min]	Area [mAU*s]	Height [mAU]	Area %
1	15.030	BB	0.3556	8285.66895	353.26224	100.0000

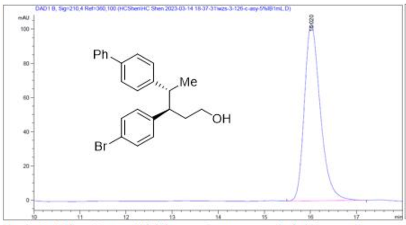

Peak #	RetTime [min]	Type	Width [min]	Area [mAU*s]	Height [mAU]	Area %
1	16.020	BB	0.3660	2485.84204	103.58686	100.0000

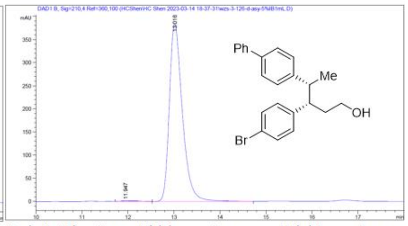

Peak #	RetTime [min]	Type	Width [min]	Area [mAU*s]	Height [mAU]	Area %
1	11.947	BB	0.3202	32.65133	1.39928	0.4311
2	13.016	BB	0.2990	7541.49951	384.17368	99.5689

4-((2*S*,3*R*)-3-(4-(trifluoromethyl)phenyl)pent-4-en-2-yl)-1,1'-biphenyl (10)

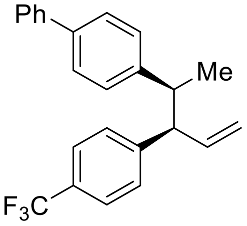

10

Prepared following **General Procedure A** using (*S*)-**1** and (*S*)-**L1**. Purification by flash column chromatography (0–1% DCM in *n*-hexane) gave the title compound (58.6 mg, 80%, >99% ee, 94:6 dr, 96:4 b:l) as a white solid.

^{1}H NMR (400 MHz, $CDCl_3$) δ 7.63 – 7.57 (m, 4H), 7.56 – 7.52 (m, 2H), 7.47 – 7.41 (m, 2H), 7.36 – 7.29 (m, 3H), 7.25 – 7.20 (m, 2H), 5.88 (ddd, *J* = 17.0, 10.3, 7.9 Hz, 1H), 4.92 (ddd, *J* = 10.3, 1.5, 0.9 Hz, 1H), 4.80 (dt, *J* = 17.1, 1.3 Hz, 1H), 3.57 – 3.48 (m, 1H), 3.18 – 3.06 (m, 1H), 1.13 (d, *J* = 7.0 Hz, 3H) ppm. **^{13}C NMR** (126 MHz, $CDCl_3$) δ 147.5 (q, *J* = 1.2 Hz), 144.0, 141.0, 139.7, 139.3, 128.9, 128.8(3) (q, *J* = 32.8 Hz), 128.7(9), 128.5, 127.3, 127.1(2), 127.1(0), 125.5 (q, *J* = 3.8 Hz), 124.5 (q, *J* = 272.2 Hz), 116.6, 57.3, 45.1, 20.7 ppm. **Specific rotation** $[\alpha]_D^{24}$ = +39 (c = 2.0, CH_2Cl_2). **HRMS** (EI) m/z calculated for $C_{24}H_{21}F_3$ $[M]^+$, 366.1590, found:

366.1582. **IR (neat)** 3029, 2969, 2927, 1616, 1486, 1324, 1162, 1120, 1067, 840, 763, 697 cm^{-1}.

The enantiomeric excess (ee) was determined after hydroboration/oxidation to alcohol **10'**.

(3*R*,4*S*)-4-([1,1'-biphenyl]-4-yl)-3-(4-(trifluoromethyl)phenyl)pentan-1-ol (10')

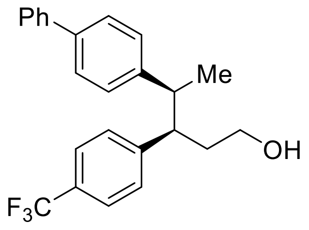

10'

Prepared following **General Procedure F (**0.1 mmol **10**, 94:6 dr, 96:4 b:l). Purification by flash column chromatography (0–30% EtOAc in *n*-hexane) gave the title compound (30.2 mg, 87%, >99% ee) based on major isomer as a colorless oil.

^{1}H NMR (500 MHz, $CDCl_3$) δ 7.63 – 7.58 (m, 4H), 7.58 – 7.55 (m, 2H), 7.48 – 7.42 (m, 2H), 7.39 – 7.32 (m, 3H), 7.32 – 7.27 (m, 2H), 3.35 (d, *J* = 6.5 Hz, 1H), 3.21 (d, *J* = 8.2 Hz, 1H), 3.02 – 2.89 (m, 2H), 1.85 – 1.75 (m, 1H), 1.76 – 1.64 (m, 1H), 1.03 (d, *J* = 6.5 Hz, 3H), 0.95 (s, 1H) ppm. **^{13}C NMR** (126 MHz, $CDCl_3$) δ 148.0 (d, *J* = 1.4 Hz), 144.9, 141.0, 139.5, 129.0 (q, *J* = 32.8 Hz), 128.9, 128.8, 128.1, 127.4, 127.3, 127.2, 125.6 (q, *J* = 3.7 Hz), 124.4 (q, *J* = 272.2 Hz), 61.0, 49.5, 46.0, 37.4, 21.1 ppm. **Specific rotation** $[\alpha]_D^{23}$ = −6 (c = 2.0, CH_2Cl_2). **HRMS** (APCI) m/z calculated for $C_{24}H_{22}F_3$ $[M+H-H_2O]^+$, 367.1668, found: 367.1660. **IR (neat)** 3325, 3028, 2927, 1618, 1486, 1323, 1162, 1115, 1067, 1017, 841, 767, 697 cm^{-1}. **HPLC conditions**: Chiral column IB, hexane: isopropanol = 97:3, flow rate = 1.0 mL/min, wavelength = 254 nm, t_R = 21.2 min for major isomer, t_R = 24.4 min for minor isomer.

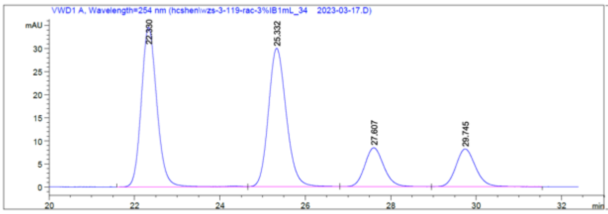

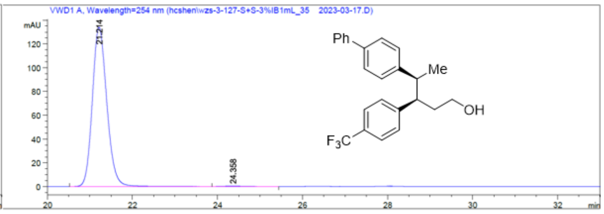

Peak #	RetTime [min]	Type	Width [min]	Area [mAU*s]	Height [mAU]	Area %
1	22.330	BV R	0.3896	878.84796	34.58737	38.5577
2	25.332	VV	0.4505	874.59033	30.02455	38.3709
3	27.607	VB	0.4804	263.30209	8.44039	11.5519
4	29.745	BB	0.4855	262.56305	8.17302	11.5194

Peak #	RetTime [min]	Type	Width [min]	Area [mAU*s]	Height [mAU]	Area %
1	21.214	BV R	0.3695	3171.96606	133.91953	99.6297
2	24.358	VB E	0.3991	11.78830	3.68839e-1	0.3703

4-((2*S*,3*S*)-3-(4-(trifluoromethyl)phenyl)pent-4-en-2-yl)-1,1'-biphenyl (11)

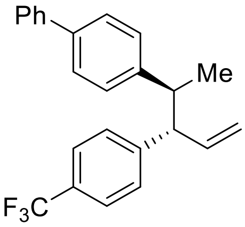

11

Prepared following **General Procedure A** using (*S*)-**1** and (*R*)-**L1**. Purification by flash column chromatography (0–1% DCM in *n*-hexane) gave the title compound (67.4 mg, 92%, >99% ee, 7:93 dr, 91.5:8.5 b:l) as a white solid.

^{1}H NMR (500 MHz, $CDCl_3$) δ 7.55 – 7.51 (m, 2H), 7.43 – 7.37 (m, 6H), 7.34 – 7.28 (m, 1H), 7.13 (d, *J* = 8.1 Hz, 2H), 7.09 – 7.04 (m, 2H), 6.09 (dt, *J* = 17.3, 9.4 Hz, 1H), 5.19 – 5.12 (m, 2H), 3.55 – 3.47 (m, 1H), 3.18 – 3.08 (m, 1H), 1.38 (d, *J* = 7.0 Hz, 3H) ppm. **^{13}C NMR** (126 MHz, $CDCl_3$) δ 147.6 (q, *J* = 1.4 Hz), 143.9, 140.9, 139.9, 139.0, 128.8, 128.5, 128.2, 127.4 (q, *J* = 31.5 Hz), 127.2, 127.0, 126.9, 125.2 (q, *J* = 3.8 Hz), 124.4 (q, *J* = 272.2 Hz), 116.7, 58.1, 44.7, 20.5 ppm. **Specific rotation** $[\alpha]_D^{24}$ = +19 (c = 2.0, CH_2Cl_2). **HRMS** (EI) m/z calculated for $C_{24}H_{21}F_3$ $[M]^+$, 366.1590, found: 366.1581. **IR (neat)** 3029, 2969, 2928, 1616, 1487, 1323, 1162, 1119, 1067, 837, 764, 733, 697 cm^{-1}.

The enantiomeric excess (ee) was determined after hydroboration/oxidation to alcohol **11'**.

(3*S*,4*S*)-4-([1,1'-biphenyl]-4-yl)-3-(4-(trifluoromethyl)phenyl)pentan-1-ol (11')

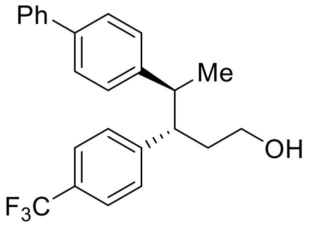

11'

Prepared following **General Procedure F (**0.1 mmol **11**, 7:93 dr, 91.5:8.5 b:l). Purification by flash column chromatography (0–30% EtOAc in *n*-hexane) gave the title compound (24.5 mg, 75%, >99% ee) based on major isomer as a colorless oil.

^{1}H NMR (500 MHz, $CDCl_3$) δ 7.58 – 7.51 (m, 2H), 7.45 – 7.37 (m, 6H), 7.35 – 7.28 (m, 1H), 7.09 (d, *J* = 8.6 Hz, 2H), 7.04 – 6.98 (m, 2H), 3.56 – 3.47 (m, 1H), 3.39 – 3.30 (m, 1H), 3.12 – 3.01 (m, 2H), 2.26 – 2.16 (m, 1H), 1.96 – 1.85 (m, 1H), 1.37 (d, *J* = 6.6 Hz, 3H), 1.16 (s, 1H) ppm. **^{13}C NMR** (126 MHz, $CDCl_3$) δ 146.9 (d, *J* = 1.4 Hz), 143.7, 140.9, 139.0, 129.2, 128.9, 128.6 (q, *J* = 32.8), 128.5, 127.2, 127.0, 126.7, 125.0 (q, *J* = 3.8 Hz), 124.4 (q, *J* = 272.2 Hz), 61.1, 49.0, 45.3, 35.3, 19.2 ppm. **Specific rotation** $[\alpha]_D^{22}$ = +26 (c = 2.0, CH_2Cl_2). **HRMS** (APCI) m/z calculated for $C_{24}H_{22}F_3$ $[M+H-H_2O]^+$, 367.1668, found: 367.1663. **IR (neat)** 3347, 2964, 1618, 1486, 1420, 1325, 1163, 1117, 1068, 838, 698 cm^{-1}. **HPLC conditions**: Chiral column IB, hexane: isopropanol = 97:3, flow rate = 1.0 mL/min, wavelength = 254 nm, t_R = 26.1 min for major isomer.

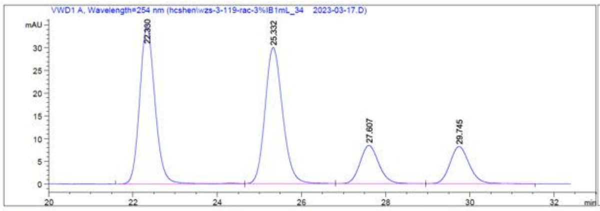

Peak #	RetTime [min]	Type	Width [min]	Area [mAU*s]	Height [mAU]	Area %
1	22.330	BV R	0.3896	878.84796	34.58737	38.5577
2	25.332	VV	0.4505	874.59033	30.02455	38.3709
3	27.607	VB	0.4804	263.30209	8.44039	11.5519
4	29.745	BB	0.4855	262.56305	8.17302	11.5194

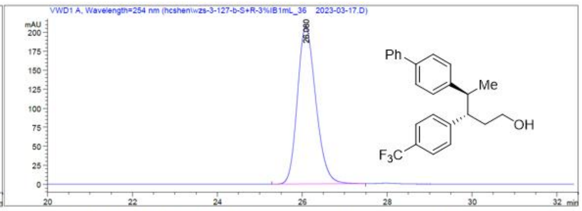

Peak #	RetTime [min]	Type	Width [min]	Area [mAU*s]	Height [mAU]	Area %
1	26.080	BB	0.4328	6241.22119	206.87437	100.0000

4-((2*S*,3*R*)-3-(*o*-tolyl)pent-4-en-2-yl)-1,1'-biphenyl (12)

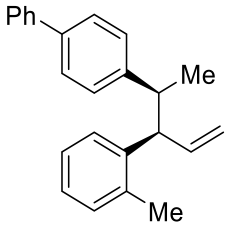

12

Prepared following **General Procedure A** using (*S*)-**1** and (*S*)-**L1**. Purification by flash column chromatography (0–1% DCM in *n*-hexane) gave the title compound (55.0 mg, 88%, 99% ee, 96:4 dr, 97:3 b:l) as a white solid.

^{1}H NMR (500 MHz, $CDCl_3$) δ 7.63 (d, *J* = 7.4 Hz, 2H), 7.56 (d, *J* = 7.9 Hz, 2H), 7.50 – 7.41 (m, 2H), 7.38 – 7.31 (m, 1H), 7.29 (d, *J* = 8.0 Hz, 2H), 7.27 – 7.18 (m, 3H), 7.19 – 7.10 (m, 1H), 5.87 – 5.76 (m, 1H), 4.83 (d, *J* = 10.2 Hz, 1H), 4.71 (d, *J* = 17.0 Hz, 1H), 3.82 – 3.75 (m, 1H), 3.21 – 3.12 (m, 1H), 2.40 (s, 3H), 1.14 (d, *J* = 6.9 Hz, 3H) ppm. **^{13}C NMR** (126 MHz, $CDCl_3$) δ 145.0, 141.5, 141.2, 140.6, 139.1, 136.3, 130.6, 128.9, 128.5, 127.3, 127.2, 127.1, 127.0, 126.3, 126.0, 115.4, 52.0, 45.0, 20.6, 20.2 ppm. **Specific rotation** $[\alpha]_D^{24}$ = +39 (c = 2.0, CH_2Cl_2). **HRMS** (EI) m/z calculated for $C_{24}H_{24}$ $[M]^+$, 312.1873, found: 312.1865. **IR (neat)** 3025, 2961, 2927, 1635, 1601, 1486, 1452, 1008, 911, 844, 755, 729, 696 cm^{-1}.

The enantiomeric excess (ee) was determined after hydroboration/oxidation to alcohol **12'**.

(3*R*,4*S*)-4-([1,1'-biphenyl]-4-yl)-3-(*o*-tolyl)pentan-1-ol (12')

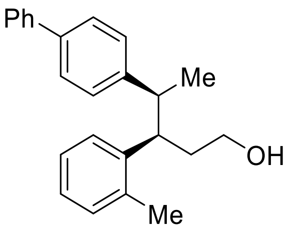

12'

Prepared following **General Procedure F (**0.1 mmol **12**, 96:4 dr, 97:3 b:l). Purification by flash column chromatography (0–30% EtOAc in *n*-hexane) gave the title compound (25.5 mg, 83%, 99% ee) based on major isomer as a colorless oil.

^{1}H NMR (500 MHz, $CDCl_3$) δ 7.65 – 7.59 (m, 2H), 7.59 – 7.53 (m, 2H), 7.50 – 7.41 (m, 2H), 7.38 – 7.29 (m, 3H), 7.30 – 7.21 (m, 2H), 7.20 – 7.16 (m, 1H), 7.16 – 7.10 (m, 1H), 3.39 – 3.18 (m, 3H), 2.97 – 2.87 (m, 1H), 2.41 (s, 3H), 1.81 – 1.64 (m, 2H), 1.03 (d, J = 6.9 Hz, 3H), 0.89 (dd, J = 6.2, 4.5 Hz, 1H) ppm. **^{13}C NMR** (126 MHz, $CDCl_3$) δ 145.8, 142.4, 141.2, 139.3, 137.3, 130.4, 128.9, 128.2, 127.4, 127.2(1), 127.1(5), 126.6, 126.0(1), 125.9(8), 61.4, 47.0, 43.2, 38.1, 20.6, 20.5 ppm. **Specific rotation** $[\alpha]_D^{23}$ = −1 (c = 2.0, CH_2Cl_2). **HRMS** (APCI) m/z calculated for $C_{24}H_{25}$ $[M+H-H_2O]^+$, 313.1951, found: 313.1953. **IR (neat)** 3344, 3025, 2926, 1687, 1601, 1486, 1450, 1037, 838, 766, 731, 697 cm^{-1}. **HPLC conditions**: Chiral column IA, hexane: isopropanol = 95:5, flow rate = 1.0 mL/min, wavelength = 254 nm, t_R = 18.9 min for minor isomer, t_R = 20.8 min for major isomer.

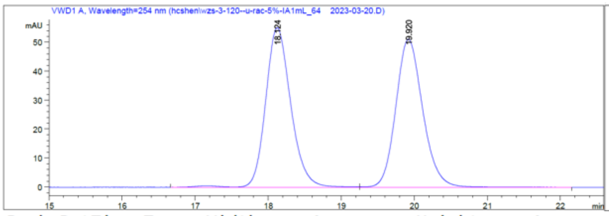

Peak #	RetTime [min]	Type	Width [min]	Area [mAU*s]	Height [mAU]	Area %
1	18.124	VV R	0.3617	1322.56714	55.14793	50.1801
2	19.920	VB	0.3924	1313.07324	51.19326	49.8199

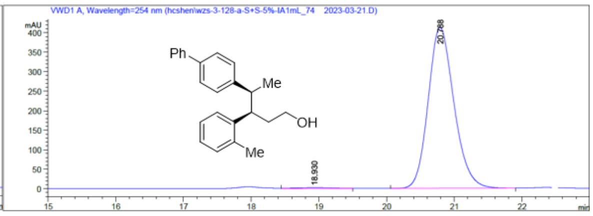

Peak #	RetTime [min]	Type	Width [min]	Area [mAU*s]	Height [mAU]	Area %
1	18.930	VV R	0.4029	71.30492	2.08162	0.6488
2	20.788	BV R	0.3745	1.09194e4	412.34683	99.3512

4-((2*S*,3*S*)-3-(*o*-tolyl)pent-4-en-2-yl)-1,1'-biphenyl (13)

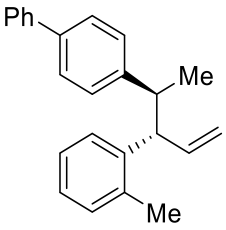

13

Prepared following **General Procedure A** using (*S*)-**1** and (*R*)-**L1**. Purification by flash column chromatography (0–1% DCM in *n*-hexane) gave the title compound (54.9 mg, 88%, >99% ee, 5:95 dr, 94.5:5.5 b:l) as a white solid.

^{1}H NMR (500 MHz, $CDCl_3$) δ 7.55 – 7.49 (m, 2H), 7.42 – 7.34 (m, 4H), 7.31 – 7.28 (m, 1H), 7.25 – 7.21 (m, 1H), 7.14 – 7.07 (m, 3H), 7.00 – 6.92 (m, 2H), 6.09 – 5.97 (m, 1H), 5.13 – 5.02

(m, 2H), 3.75 – 3.68 (m, 1H), 3.22 – 3.12 (m, 1H), 2.14 (s, 3H), 1.40 (d, *J* = 6.9 Hz, 3H) ppm. **^{13}C NMR** (126 MHz, $CDCl_3$) δ 145.0, 141.7, 141.1, 140.7, 138.6, 135.6, 130.4, 128.8, 128.0, 127.2, 127.1, 127.0, 126.7, 125.9, 125.7, 115.6, 52.9, 43.9, 20.6, 19.8 ppm. **Specific rotation** $[\alpha]_D^{24}$ = –22 (c = 2.0, CH_2Cl_2). **HRMS** (EI) m/z calculated for $C_{24}H_{24}$ $[M]^+$, 312.1873, found: 312.1867. **IR (neat)** 3026, 2969, 2927, 1634, 1601, 1486, 1452, 1008, 911, 838, 764, 728, 697 cm^{-1}.

The enantiomeric excess (ee) was determined after hydroboration/oxidation to alcohol **13'**.

(3*S*,4*S*)-4-([1,1'-biphenyl]-4-yl)-3-(*o*-tolyl)pentan-1-ol (13')

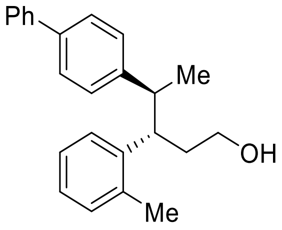

13'

Prepared following **General Procedure F (**0.1 mmol **13**, 5:95 dr, 94.5:5.5 b:l). Purification by flash column chromatography (0–30% EtOAc in *n*-hexane) gave the title compound (19.8 mg, 66%, >99% ee) based on major isomer as a colorless oil.

^{1}H NMR (500 MHz, $CDCl_3$) δ 7.58 – 7.51 (m, 2H), 7.44 – 7.36 (m, 4H), 7.34 – 7.27 (m, 1H), 7.18 – 7.06 (m, 4H), 7.04 – 6.96 (m, 2H), 3.53 – 3.44 (m, 1H), 3.38 – 3.23 (m, 2H), 3.08 – 2.99 (m, 1H), 2.21 – 2.11 (m, 4H), 2.01 – 1.90 (m, 1H), 1.40 (d, *J* = 7.0 Hz, 3H), 1.06 (dd, *J* = 5.7, 4.6 Hz, 1H) ppm. **^{13}C NMR** (126 MHz, $CDCl_3$) δ 145.0, 141.6, 141.1, 138.7, 136.8, 130.4, 128.8, 128.2, 127.1(0), 127.0(4), 126.6, 125.9, 125.8, 61.6, 44.8, 34.9, 20.0, 17.9 ppm. **Specific rotation** $[\alpha]_D^{23}$ = +47 (c = 2.0, CH_2Cl_2). **HRMS** (APCI) m/z calculated for $C_{24}H_{25}$ $[M+H-H_2O]^+$, 313.1951, found: 313.1950. **IR (neat)** 3344, 2930, 1486, 1033, 838, 766, 732, 697 cm^{-1}. **HPLC conditions**: Chiral column IA, hexane: isopropanol = 95:5, flow rate = 1.0 mL/min, wavelength = 254 nm, t_R = 17.6 min for major isomer.

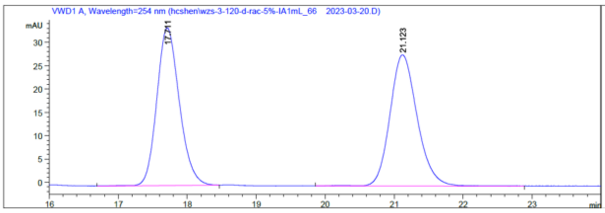

Peak #	RetTime [min]	Type	Width [min]	Area [mAU*s]	Height [mAU]	Area %
1	17.711	BB	0.3543	766.79449	33.87653	49.7034
2	21.123	BB	0.4291	775.94507	28.17472	50.2966

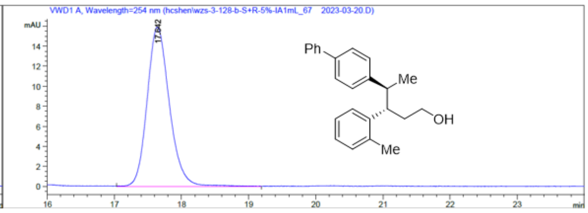

Peak #	RetTime [min]	Type	Width [min]	Area [mAU*s]	Height [mAU]	Area %
1	17.642	BB	0.3496	365.03296	15.88653	100.0000

4-((2*S*,3*R*)-3-(3-methoxyphenyl)pent-4-en-2-yl)-1,1'-biphenyl (14)

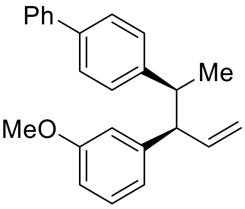

14

Prepared following **General Procedure A** using (*S*)-**1** and (*S*)-**L1**. Purification by flash column chromatography (0–20% DCM in *n*-hexane) gave the title compound (50.6 mg, 77%, >99% ee, 94:6 dr, 97.5:2.5 b:l) as a pale yellow solid. Note: PhBpin was challenging to remove via column chromatography. However, an effective method involved mixing it with $MeB(OH)_2$ in a 5% TFA/DCM solution, followed by subsequent flash column chromatography (0–20% DCM in *n*-hexane).[16]

^{1}H NMR (500 MHz, $CDCl_3$) δ 7.62 – 7.58 (m, 2H), 7.55 – 7.51 (m, 2H), 7.45 – 7.39 (m, 2H), 7.35 – 7.29 (m, 1H), 7.27 – 7.22 (m, 3H), 6.82 (dt, *J* = 7.5, 1.3 Hz, 1H), 6.80 – 6.76 (m, 1H), 6.76 – 6.73 (m, 1H), 5.86 (ddd, *J* = 17.0, 10.3, 8.1 Hz, 1H), 4.89 – 4.83 (m, 1H), 4.79 (dt, *J* = 17.1, 1.4 Hz, 1H), 3.80 (s, 3H), 3.45 – 3.38 (m, 1H), 3.14 – 3.04 (m, 1H), 1.13 (d, *J* = 6.9 Hz, 3H) ppm. **^{13}C NMR** (126 MHz, $CDCl_3$) δ 159.8, 145.1, 144.7, 141.2, 140.5, 139.1, 129.5, 128.8, 128.5, 127.2, 127.1, 127.0, 120.8, 115.7, 114.3, 111.6, 57.5, 55.3, 45.2, 20.9 ppm. **Specific rotation** $[\alpha]_D^{24}$ = +43 (c = 2.0, CH_2Cl_2). **HRMS** (EI) m/z calculated for $C_{24}H_{24}O$ $[M]^+$, 328.1822, found: 328.1814. **IR (neat)** 3027, 2960, 1599, 1583, 1485, 1452, 1260, 1156, 1046, 752, 696 cm^{-1}.

The enantiomeric excess (ee) was determined after hydroboration/oxidation to alcohol **14'**.

(3*R*,4*S*)-4-([1,1'-biphenyl]-4-yl)-3-(3-methoxyphenyl)pentan-1-ol (14')

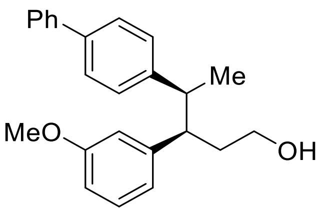

14'

Prepared following **General Procedure F (**0.1 mmol **14**, 94:6 dr, 97.5:2.5 b:l). Purification by flash column chromatography (0–30% EtOAc in *n*-hexane) gave the title compound (26.4 mg, 83%, >99% ee) based on major isomer as a colorless oil.

^{1}H NMR (500 MHz, $CDCl_3$) δ 7.63 – 7.58 (m, 2H), 7.58 – 7.53 (m, 2H), 7.47 – 7.40 (m, 2H), 7.37 – 7.24 (m, 4H), 6.88 – 6.83 (m, 1H), 6.82 – 6.77 (m, 2H), 3.82 (s, 3H), 3.41 – 3.32 (m, 1H), 3.32 – 3.23 (m, 1H), 2.95 – 2.77 (m, 2H), 1.79 – 1.62 (m, 2H), 1.05 (d, *J* = 6.8 Hz, 3H), 0.95 (dd, *J* = 6.0, 4.9 Hz, 1H) ppm. **^{13}C NMR** (126 MHz, $CDCl_3$) δ 159.9, 145.5(4), 145.4(5), 141.2, 139.3, 129.6, 128.9, 128.1, 127.4, 127.2(0), 127.1(5), 120.8, 114.3, 111.5, 61.5, 55.3, 49.9, 46.2, 37.6, 21.2 ppm. **Specific rotation** $[\alpha]_D^{23}$ = −4 (c = 2.0, CH_2Cl_2). **HRMS** (APCI) m/z calculated for $C_{24}H_{25}O$ $[M+H-H_2O]^+$, 329.1896, found: 329.1900. **IR (neat)** 3347, 3027, 2928, 1599, 1583, 1485, 1452, 1257, 1154, 1039, 838, 767, 738, 697 cm^{-1}. **HPLC conditions**: Chiral column IB, hexane: isopropanol = 95:5, flow rate = 1.0 mL/min, wavelength = 254 nm, t_R = 46.7 min for major isomer, t_R = 39.4 min for minor isomer.

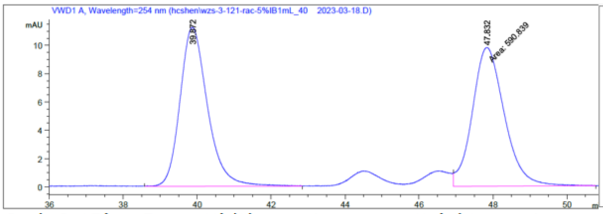

Peak #	RetTime [min]	Type	Width [min]	Area [mAU*s]	Height [mAU]	Area %
1	39.872	BB	0.7721	580.23151	11.22302	49.5471
2	47.832	MM	1.0045	590.83856	9.80342	50.4529

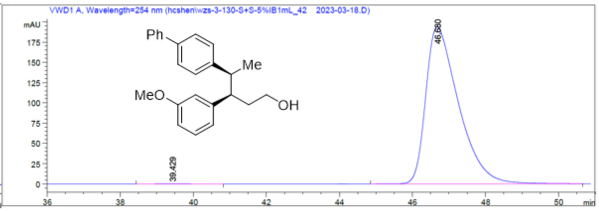

Peak #	RetTime [min]	Type	Width [min]	Area [mAU*s]	Height [mAU]	Area %
1	39.429	BB	0.6358	20.89890	3.87052e-1	0.1731
2	46.680	BB	0.9179	1.20514e4	193.75383	99.8269

4-((2*S*,3*R*)-3-(2-fluorophenyl)pent-4-en-2-yl)-1,1'-biphenyl (15)

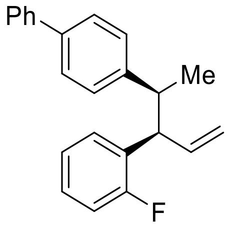

15

Prepared following **General Procedure A** using (*S*)-**1** and (*S*)-**L1**. Purification by flash column chromatography (0–1% DCM in *n*-hexane) gave the title compound (53.8 mg, 85%, >99% ee, 93.5:6.5 dr, 96:4 b:l) as a white solid.

^{1}H NMR (500 MHz, $CDCl_3$) δ 7.65 – 7.59 (m, 2H), 7.58 – 7.53 (m, 2H), 7.48 – 7.42 (m, 2H), 7.37 – 7.32 (m, 1H), 7.28 (d, *J* = 8.2 Hz, 2H), 7.25 – 7.20 (m, 2H), 7.16 – 7.05 (m, 2H), 5.97 – 5.83 (m, 1H), 4.90 (dt, *J* = 10.2, 1.2 Hz, 1H), 4.82 (dt, *J* = 17.0, 1.5 Hz, 1H), 3.88 – 3.81 (m, 1H), 3.23 – 3.13 (m, 1H), 1.16 (d, *J* = 6.9 Hz, 3H) ppm. **^{13}C NMR** (126 MHz, $CDCl_3$) δ 161.0 (d, *J* = 244.7 Hz), 144.5, 141.1, 139.2, 139.1, 130.3 (d, *J* = 14.5 Hz), 129.6 (d, *J* = 5.1 Hz), 128.9, 128.5, 127.9 (d, *J* = 8.3 Hz), 127.2, 127.1, 127.0, 124.3 (d, *J* = 3.4 Hz), 116.3, 115.7 (d, *J* = 23.1 Hz), 50.2, 44.3 (d, *J* = 1.6 Hz), 20.9 ppm. **Specific rotation** $[\alpha]_D^{24}$ = +42 (c = 2.0, CH_2Cl_2). **HRMS** (EI) m/z calculated for $C_{23}H_{21}F$ $[M]^+$, 316.1622, found: 316.1612. **IR (neat)** 3028, 2960, 2925, 1582, 1488, 1452, 1227, 905, 831, 758, 726, 690 cm^{-1}.

The enantiomeric excess (ee) was determined after hydroboration/oxidation to alcohol **15'**.

(3*R*,4*S*)-4-([1,1'-biphenyl]-4-yl)-3-(2-fluorophenyl)pentan-1-ol (15')

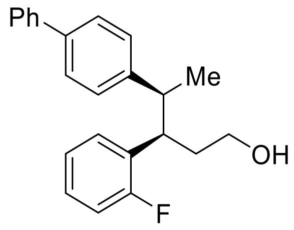

15'

Prepared following **General Procedure F (**0.1 mmol **15**, 93.5:6.5 dr, 96:4 b:l). Purification by flash column chromatography (0–30% EtOAc in *n*-hexane) gave the title compound (17.7 mg,

59%, >99% ee) based on major isomer as a white solid.

^{1}H NMR (500 MHz, $CDCl_3$) δ 7.64 – 7.59 (m, 2H), 7.57 (d, *J* = 8.2 Hz, 2H), 7.48 – 7.41 (m, 2H), 7.38 – 7.32 (m, 3H), 7.32 – 7.20 (m, 2H), 7.19 – 7.13 (m, 1H), 7.11 – 7.04 (m, 1H), 3.42 – 3.32 (m, 1H), 3.32 – 3.20 (m, 2H), 3.07 – 2.96 (m, 1H), 1.80 – 1.70 (m, 2H), 1.07 (d, *J* = 6.9 Hz, 3H), 1.05 – 0.97 (m, 1H) ppm. **^{13}C NMR** (126 MHz, $CDCl_3$) δ 161.8 (d, *J* = 244.1 Hz), 145.3, 141.1, 139.4, 130.4 (d, *J* = 14.6 Hz), 129.4, 128.9, 128.1, 128.0 (d, *J* = 8.4 Hz), 127.4, 127.2(2), 127.1(6), 124.5 (d, *J* = 3.4 Hz), 115.7 (d, *J* = 23.3 Hz), 61.4, 45.2, 36.5, 21.2 ppm. **Specific rotation** $[\alpha]_D^{23}$ = −12 (c = 2.0, CH_2Cl_2). HRMS (EI) m/z calculated for $C_{23}H_{22}F$ $[M+H-H_2O]^+$, 317.1700, found: 317.1699. **IR (neat)** 3338, 3028, 2928, 1582, 1487, 1453, 1227, 1033, 838, 757, 736, 697. **HPLC conditions**: Chiral column IB, hexane: isopropanol = 90:10, flow rate = 1.0 mL/min, wavelength = 254 nm, t_R = 7.1 min for major isomer, t_R = 6.3 min for minor isomer.

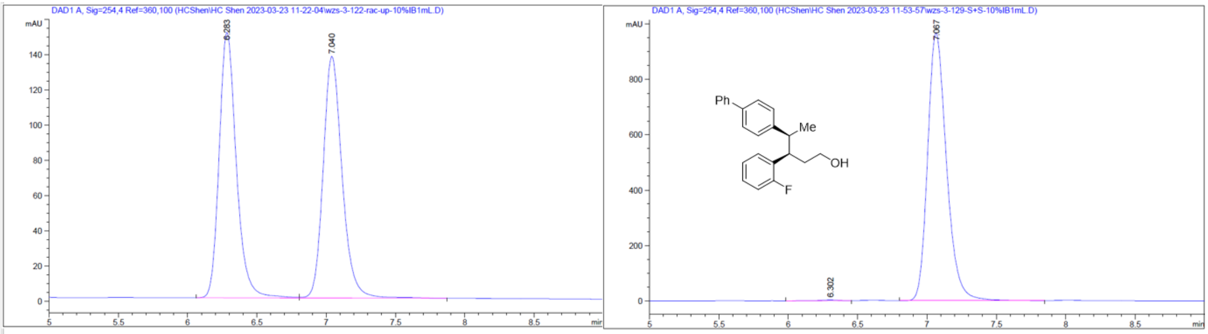

Peak #	RetTime [min]	Type	Width [min]	Area [mAU*s]	Height [mAU]	Area %
1	6.283	BV	0.1325	1300.72900	150.97272	49.8934
2	7.040	VB	0.1449	1306.28674	137.41864	50.1066

Peak #	RetTime [min]	Type	Width [min]	Area [mAU*s]	Height [mAU]	Area %
1	6.302	BV	0.1382	28.07437	3.02647	0.3059
2	7.067	BB	0.1443	9149.13281	967.31592	99.6941

2-((3*R*,4*S*)-4-([1,1'-biphenyl]-4-yl)pent-1-en-3-yl)thiophene (16)

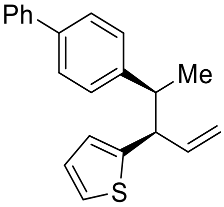

16

Prepared following **General Procedure A** using (*S*)-**1** and (*S*)-**L1**. Purification by flash column

chromatography (0–1% DCM in *n*-hexane) gave the title compound (43.8 mg, 75%, >99% ee, 92.5:7.5 dr, 96:4 b:l) as a white solid.

^{1}H NMR (400 MHz, $CDCl_3$) δ 7.64 – 7.57 (m, 2H), 7.55 – 7.50 (m, 2H), 7.47 – 7.39 (m, 2H), 7.36 – 7.30 (m, 1H), 7.25 – 7.18 (m, 3H), 6.97 (dd, *J* = 5.1, 3.4 Hz, 1H), 6.85 – 6.79 (m, 1H), 5.84 (ddd, *J* = 16.9, 10.2, 8.1 Hz, 1H), 4.98 – 4.86 (m, 2H), 3.84 – 3.73 (m, 1H), 3.17 – 3.04 (m, 1H), 1.25 (d, *J* = 7.0 Hz, 3H) ppm. **^{13}C NMR** (126 MHz, $CDCl_3$) δ 146.6, 144.0, 141.1, 140.0, 139.2, 128.9, 128.5, 127.2, 127.1, 127.0, 126.7, 124.5, 123.5, 116.1, 52.4, 46.4, 20.5 ppm. **Specific rotation** $[\alpha]_D^{24}$ = +53 (c = 2.0, CH_2Cl_2). **HRMS** (EI) m/z calculated for $C_{21}H_{20}S$ $[M]^+$, 304.1280, found: 304.1274. **IR (neat)** 3028, 2965, 2923, 1636, 1598, 1486, 1450, 1408, 1008, 913, 835, 759, 728, 691 cm^{-1}. **HPLC conditions**: Chiral column IB, hexane: isopropanol = 95:5, flow rate = 1.0 mL/min, wavelength = 254 nm, t_R = 17.7 min for minor enantiomer, t_R = 19.7 min for minor diastereomer, t_R = 22.7 min for minor diastereomer, t_R = 25.1 min for major enantiomer.

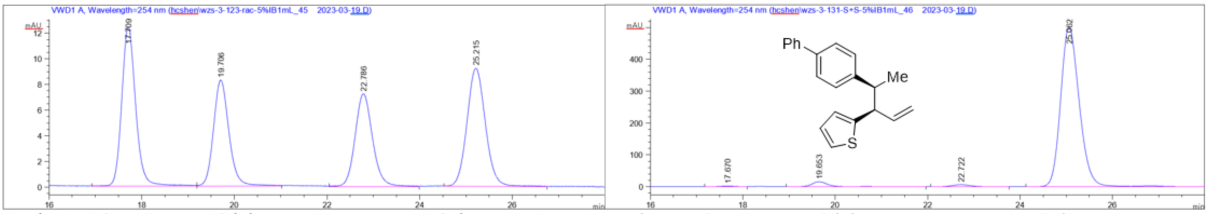

Peak #	RetTime [min]	Type	Width [min]	Area [mAU*s]	Height [mAU]	Area %
1	17.709	BV	0.3196	258.48257	12.36588	29.0334
2	19.706	VB	0.3494	189.00949	8.23138	21.2300
3	22.786	BB	0.3976	187.86963	7.19920	21.1020
4	25.215	BB	0.4228	254.93239	9.18625	28.6346

Peak #	RetTime [min]	Type	Width [min]	Area [mAU*s]	Height [mAU]	Area %
1	17.670	BV	0.3113	33.81427	1.67423	0.2313
2	19.653	BV R	0.3506	366.28268	15.44908	2.5060
3	22.722	BB	0.4071	166.42052	6.30271	1.1386
4	25.062	BV R	0.4346	1.40498e4	501.38828	96.1241

2-((3*S*,4*S*)-4-([1,1'-biphenyl]-4-yl)pent-1-en-3-yl)thiophene (17)

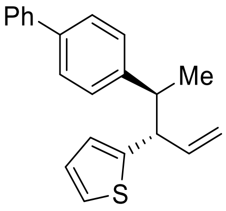

17

Prepared following **General Procedure A** using (*S*)-**1** and (*R*)-**L1**. Purification by flash column

chromatography (0–1% DCM in *n*-hexane) gave the title compound (32.0 mg, 53%, >99% ee, 7.5:92.5 dr, 96:4 b:l) as a pale yellow oil.

^{1}H NMR (500 MHz, $CDCl_3$) δ 7.59 – 7.54 (m, 2H), 7.48 – 7.43 (m, 2H), 7.43 – 7.38 (m, 2H), 7.34 – 7.27 (m, 1H), 7.21 – 7.15 (m, 2H), 7.05 (dd, J = 5.1, 1.2 Hz, 1H), 6.80 (dd, J = 5.1, 3.5 Hz, 1H), 6.61 (dt, J = 3.5, 1.0 Hz, 1H), 6.05 (ddd, J = 16.8, 10.1, 9.2 Hz, 1H), 5.17 – 5.08 (m, 2H), 3.77 (t, J = 8.8 Hz, 1H), 3.17 – 3.07 (m, 1H), 1.36 (d, J = 7.1 Hz, 3H) ppm. **^{13}C NMR** (126 MHz, $CDCl_3$) δ 147.0, 144.4, 141.1, 139.6, 139.0, 128.8, 128.3, 127.1(4), 127.0(7), 126.8, 126.5, 124.2, 123.2, 116.5, 53.0, 45.9, 19.7 ppm. **Specific rotation** $[\alpha]_D^{24}$ = +2 (c = 2.0, CH_2Cl_2). **HRMS** (EI) m/z calculated for $C_{21}H_{20}S$ $[M]^+$, 304.1280, found: 304.1276. **IR (neat)** 3027, 2969, 2925, 1637, 1486, 1450, 1411, 1232, 1008, 917, 835, 764, 733, 695 cm^{-1}. **HPLC conditions**: Chiral column IB, hexane: isopropanol = 95:5, flow rate = 1.0 mL/min, wavelength = 254 nm, t_R = 17.4 min for minor diastereomer, t_R = 19.3 min for major enantiomer, t_R = 22.5 min for minor enantiomer, t_R = 24.8 min for minor diastereomer.

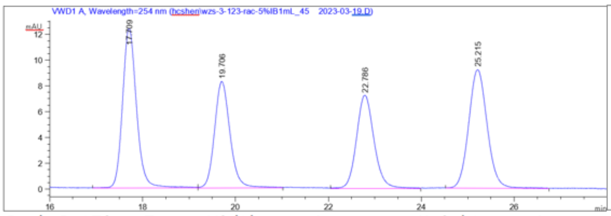

Peak #	RetTime [min]	Type	Width [min]	Area [mAU*s]	Height [mAU]	Area %
1	17.709	BV	0.3196	258.48257	12.36588	29.0334
2	19.706	VB	0.3494	189.00949	8.23138	21.2300
3	22.786	BB	0.3976	187.86963	7.19920	21.1020
4	25.215	BB	0.4228	254.93239	9.18625	28.6346

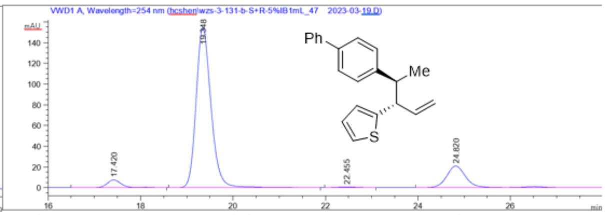

Peak #	RetTime [min]	Type	Width [min]	Area [mAU*s]	Height [mAU]	Area %
1	17.420	BV R	0.3127	157.88478	7.41474	3.7357
2	19.348	BB	0.3414	3437.07349	154.31427	81.3248
3	22.455	BB	0.3989	15.17545	5.57940e-1	0.3591
4	24.820	BV R	0.4340	616.22052	20.77639	14.5804

3-((3*R*,4*S*)-4-([1,1'-biphenyl]-4-yl)pent-1-en-3-yl)-1-tosyl-1*H*-indole (18)

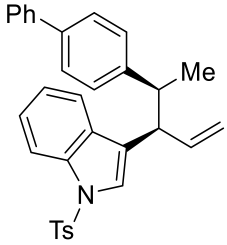

18

Prepared following **General Procedure A** using (*S*)-**1** and (*S*)-**L1**. Purification by flash column

chromatography (0–30% DCM in *n*-hexane) gave the title compound (75.1 mg, 76%, >99% ee, 93:7 dr, >99:1 b:l) as a pale yellow solid.

^{1}H NMR (500 MHz, $CDCl_3$) δ 8.01 (d, *J* = 8.4 Hz, 1H), 7.71 (d, *J* = 8.3 Hz, 2H), 7.59 (d, *J* = 7.6 Hz, 2H), 7.50 (d, *J* = 8.3 Hz, 1H), 7.48 – 7.41 (m, 4H), 7.38 – 7.29 (m, 3H), 7.26 – 7.19 (m, 1H), 7.19 – 7.13 (m, 2H), 7.11 (d, *J* = 8.3 Hz, 2H), 5.88 (ddd, *J* = 16.7, 10.2, 7.8 Hz, 1H), 5.01 – 4.95 (m, 1H), 4.91 (dt, *J* = 17.0, 1.5 Hz, 1H), 3.77 – 3.69 (m, 1H), 3.33 – 3.22 (m, 1H), 2.30 (s, 3H), 1.23 (d, *J* = 6.9 Hz, 3H) ppm. **^{13}C NMR** (126 MHz, $CDCl_3$) δ 144.9, 143.6, 141.2, 139.3, 138.0, 135.5, 135.4, 130.6, 129.9, 128.9, 128.5, 127.2, 127.1, 126.9(3), 126.8(8), 124.8, 124.1, 123.7, 123.1, 120.3, 116.5, 114.1, 48.0, 43.6, 21.7, 20.2 ppm. **Specific rotation** $[\alpha]_D^{24}$ = +15 (c = 3.0, CH_2Cl_2). **HRMS** (ESI) m/z calculated for $C_{32}H_{30}NO_2S$ $[M+H]^+$, 492.1992, found: 492.1990. **IR (neat)** 3028, 2955, 2925, 1598, 1486, 1446, 1367, 1172, 1120, 976, 734, 697, 662 cm^{-1}.

The enantiomeric excess (ee) was determined after hydroboration/oxidation to alcohol **18'**.

(3*R*,4*S*)-4-([1,1'-biphenyl]-4-yl)-3-(1-tosyl-1*H*-indol-3-yl)pentan-1-ol (18')

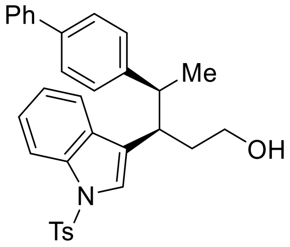

18'

Prepared following **General Procedure F (**0.1 mmol **18**, 93:7 dr, >99:1 b:l). Purification by flash column chromatography (0–30% EtOAc in *n*-hexane) gave the title compound (33.7 mg, 71%, >99% ee) based on major isomer as a white solid.

^{1}H NMR (500 MHz, $CDCl_3$) δ 8.01 (d, *J* = 8.3 Hz, 1H), 7.73 (d, *J* = 8.5 Hz, 2H), 7.61 – 7.55 (m, 2H), 7.53 – 7.41 (m, 5H), 7.39 (s, 1H), 7.37 – 7.29 (m, 2H), 7.23 – 7.16 (m, 5H), 3.43 – 3.34 (m, 1H), 3.31 – 3.21 (m, 1H), 3.21 – 3.05 (m, 2H), 2.32 (s, 3H), 1.90 – 1.75 (m, 2H), 1.07 (d, *J* = 6.7 Hz, 3H), 0.96 (dd, *J* = 5.9, 4.8 Hz, 1H) ppm. **^{13}C NMR** (126 MHz, $CDCl_3$) δ 145.0,

144.8, 141.1, 139.5, 135.8, 135.2, 130.9, 129.9, 128.9, 128.1, 127.3(1), 127.2(6), 127.2, 126.8, 124.9, 124.8, 124.0, 123.3, 120.3, 114.3, 61.3, 44.6, 40.6, 35.9, 21.7, 20.5 ppm. **Specific rotation** $[\alpha]_D^{23}$ = −10 (c = 2.0, CH_2Cl_2). **HRMS** (ESI) m/z calculated for $C_{32}H_{32}NO_3S$ $[M+H]^+$, 510.2097, found: 510.2119. **IR (neat)** 3387, 2964, 2925, 1598, 1486, 1447, 1366, 1173, 1123, 737, 672 cm^{-1}. **HPLC conditions**: Chiral column IB, hexane: isopropanol = 90:10, flow rate = 1.0 mL/min, wavelength = 254 nm, t_R = 14.3 min for major isomer.

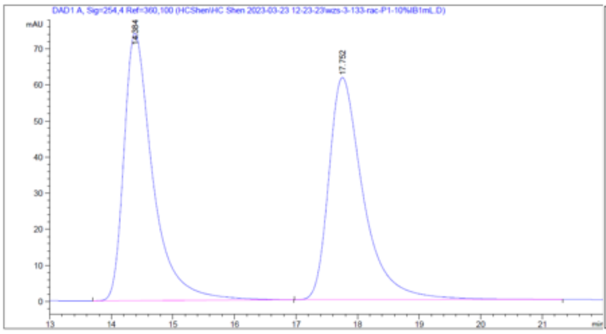

Peak #	RetTime [min]	Type	Width [min]	Area [mAU*s]	Height [mAU]	Area %
1	14.384	BB	0.4851	2418.27368	74.03778	50.2269
2	17.752	BB	0.5846	2396.42627	61.50496	49.7731

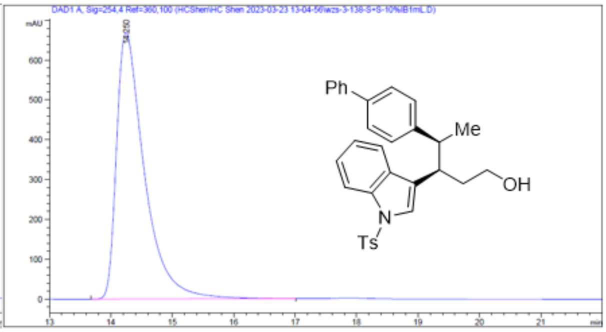

Peak #	RetTime [min]	Type	Width [min]	Area [mAU*s]	Height [mAU]	Area %
1	14.250	BB	0.4699	2.11125e4	669.54413	100.0000

3-((3*S*,4*S*)-4-([1,1'-biphenyl]-4-yl)pent-1-en-3-yl)-1-tosyl-1*H*-indole (19)

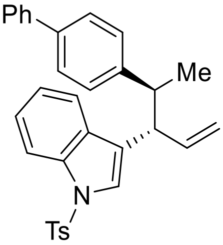

19

Prepared following **General Procedure A** using (*S*)-**1** and (*R*)-**L1**. Purification by flash column chromatography (0–30% DCM in *n*-hexane) gave the title compound (86.1 mg, 88%, >99% ee, 8.5:91.5 dr, 99:1 b:l) as a white solid.

^{1}H NMR (500 MHz, $CDCl_3$) δ 7.83 (d, *J* = 7.7 Hz, 1H), 7.58 – 7.50 (m, 3H), 7.46 – 7.37 (m, 4H), 7.36 – 7.30 (m, 3H), 7.25 – 7.14 (m, 5H), 6.86 (d, *J* = 8.1 Hz, 2H), 6.02 (ddd, *J* = 17.0, 10.0, 8.8 Hz, 1H), 5.22 – 5.12 (m, 2H), 3.75 (t, *J* = 9.3 Hz, 1H), 3.34 – 3.25 (m, 1H), 2.07 (s, 3H), 1.39 (d, *J* = 6.9 Hz, 3H) ppm. **^{13}C NMR** (126 MHz, $CDCl_3$) δ 145.1, 144.5, 140.8, 138.9,

138.6, 135.2, 135.1, 130.6, 129.8, 128.9, 127.9, 127.3, 126.9(3), 126.9(2), 126.5, 124.5, 123.9(3), 123.9(2), 123.0, 120.1, 116.8, 113.8, 48.2, 43.0, 21.4, 21.1 ppm. **Specific rotation** $[\alpha]_D^{24}$ = +9 (c = 3.0, CH_2Cl_2). **HRMS** (ESI) m/z calculated for $C_{32}H_{30}NO_2S$ $[M+H]^+$, 492.1992, found: 492.1988. **IR (neat)** 3028, 2964, 2925, 1598, 1486, 1447, 1367, 1278, 1172, 1130, 975, 734, 696 cm^{-1}.

The enantiomeric excess (ee) was determined after hydroboration/oxidation to alcohol **19'**.

(3*S*,4*S*)-4-([1,1'-biphenyl]-4-yl)-3-(1-tosyl-1*H*-indol-3-yl)pentan-1-ol (19')

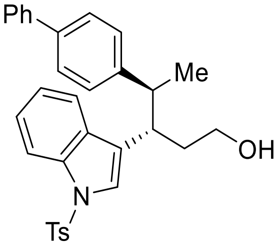

19'

Prepared following **General Procedure F (**0.1 mmol **19**, 8.5:91.5 dr, 99:1 b:l). Purification by flash column chromatography (0–30% EtOAc in *n*-hexane) gave the title compound (35.0 mg, 76%, >99% ee) based on major isomer as a colorless oil.

^{1}H NMR (500 MHz, $CDCl_3$) δ 7.90 – 7.85 (m, 1H), 7.58 – 7.48 (m, 3H), 7.48 – 7.39 (m, 4H), 7.37 – 7.29 (m, 3H), 7.28 – 7.23 (m, 1H), 7.22 – 7.17 (m, 1H), 7.12 (s, 1H), 7.06 (d, *J* = 8.2 Hz, 2H), 7.02 – 6.95 (m, 2H), 3.60 – 3.49 (m, 1H), 3.44 – 3.35 (m, 1H), 3.34 – 3.18 (m, 2H), 2.25 – 2.16 (m, 1H), 2.15 (s, 3H), 2.02 – 1.92 (m, 1H), 1.35 (d, *J* = 6.9 Hz, 3H), 1.18 – 1.11 (m, 1H) ppm. **^{13}C NMR** (126 MHz, $CDCl_3$) δ 144.7, 144.6, 141.0, 138.8, 135.3(1), 135.2(8), 130.9, 129.8, 128.9, 128.2, 127.3, 127.0, 126.8, 126.6, 124.6, 124.1, 123.9, 123.1, 120.2, 113.9, 61.2, 43.6, 40.0, 34.6, 21.5, 19.1 ppm. **Specific rotation** $[\alpha]_D^{23}$ = +36 (c = 1.0, CH_2Cl_2). **HRMS** (ESI) m/z calculated for $C_{32}H_{32}NO_3S$ $[M+H]^+$, 510.2097, found: 510.2120. **IR (neat)** 3383, 2960, 2927, 1597, 1447, 1365, 1172, 1123, 734, 671 cm^{-1}. **HPLC conditions**: Chiral column IC, hexane: isopropanol = 80:20, flow rate = 1.0 mL/min, wavelength = 254 nm, t_R = 20.5 min for major isomer.

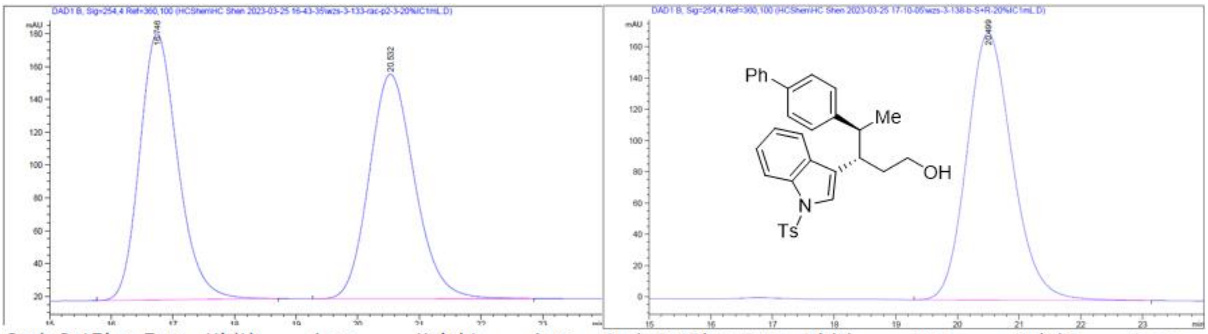

Peak #	RetTime [min]	Type	Width [min]	Area [mAU*s]	Height [mAU]	Area %
1	16.746	BB	0.6616	6974.91650	162.22534	49.9266
2	20.532	BB	0.7887	6995.43115	136.78378	50.0734

Peak #	RetTime [min]	Type	Width [min]	Area [mAU*s]	Height [mAU]	Area %
1	20.499	BB	0.7904	8732.55566	170.82150	100.0000

4-((2*S*,3*R*)-7-phenyl-3-vinylhept-4-yn-2-yl)-1,1'-biphenyl (20)

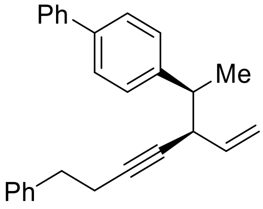

20

Prepared following **General Procedure A** using (*S*)-**1** and (*S*)-**L1**. Purification by flash column chromatography (0–1% DCM in *n*-hexane) gave the title compound (52.4 mg, 75%, >99% ee, 92.5:7.5 dr, >99:1 b:l) as a colorless oil.

^{1}H NMR (500 MHz, $CDCl_3$) δ 7.62 – 7.57 (m, 2H), 7.53 – 7.49 (m, 2H), 7.46 – 7.40 (m, 2H), 7.36 – 7.26 (m, 5H), 7.25 – 7.18 (m, 3H), 5.69 (ddd, *J* = 16.9, 10.0, 5.9 Hz, 1H), 5.25 (dt, *J* = 16.9, 1.7 Hz, 1H), 5.05 (dt, *J* = 10.0, 1.6 Hz, 1H), 3.32 – 3.24 (m, 1H), 2.92 – 2.81 (m, 3H), 2.61 – 2.52 (m, 2H), 1.34 (d, *J* = 7.0 Hz, 3H) ppm. **^{13}C NMR** (126 MHz, $CDCl_3$) δ 144.1, 141.2, 141.0, 139.3, 137.4, 128.9, 128.6, 128.5, 128.3, 127.1(8), 127.1(5), 127.0, 126.3, 116.0, 84.9, 79.6, 44.1, 43.3, 35.6, 21.1, 18.2 ppm. **Specific rotation** $[\alpha]_D^{24}$ = −1 (c = 2.0, CH_2Cl_2). **GC-MS** (EI) m/z calculated for $C_{27}H_{26}$ $[M]^+$, 350.2, found: 350.2. Note: HRMS (EI, ESI and APCI) was tried, but didn't find desired mass. **IR (neat)** 3027, 2966, 2926, 1638, 1602, 1486, 1453, 1008, 919, 838, 764, 733, 696 cm^{-1}.

The enantiomeric excess (ee) was determined after hydroboration/oxidation to alcohol **20'**.

(*R*)-3-((*S*)-1-([1,1'-biphenyl]-4-yl)ethyl)-7-phenylhept-4-yn-1-ol (20')

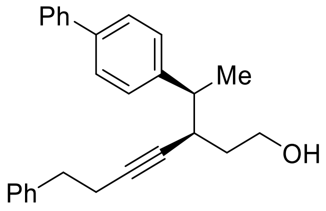

20'

Prepared following **General Procedure F** (0.1 mmol **20**, 92.5:7.5 dr, >99:1 b:l). Purification by flash column chromatography (0–30% EtOAc in *n*-hexane) gave the title compound (14.0 mg, 41%, >99% ee) based on major isomer as a white solid.

^{1}H NMR (500 MHz, $CDCl_3$) δ 7.60 – 7.55 (m, 2H), 7.53 – 7.48 (m, 2H), 7.46 – 7.40 (m, 2H), 7.35 – 7.28 (m, 3H), 7.25 – 7.18 (m, 5H), 3.73 – 3.65 (m, 2H), 2.84 (t, *J* = 7.5 Hz, 2H), 2.77 – 2.68 (m, 1H), 2.70 – 2.62 (m, 1H), 2.58 – 2.48 (m, 2H), 1.64 – 1.55 (m, 1H), 1.53 – 1.44 (m, 1H), 1.37 (d, *J* = 6.9 Hz, 3H) ppm. **^{13}C NMR** (126 MHz, $CDCl_3$) δ 144.5, 141.1, 140.9, 139.4, 128.9, 128.6, 128.5, 128.0, 127.2(1), 127.2(0), 127.1(5), 126.4, 83.3, 82.3, 61.7, 44.2, 36.5, 36.3, 35.5, 21.0, 19.6 ppm. **Specific rotation** $[\alpha]_D^{23}$ = −11 (c = 2.0, CH_2Cl_2). **HRMS** (APCI) m/z calculated for $C_{27}H_{29}O$ $[M+H]^+$, 369.2213, found: 369.2206. **IR (neat)** 3351, 3027, 2925, 1602, 1486, 1453, 1008, 839, 766, 735, 696 cm^{-1}. **HPLC conditions**: Chiral column IB, hexane: isopropanol = 95:5, flow rate = 1.0 mL/min, wavelength = 210 nm, t_R = 15.0 min for major isomer.

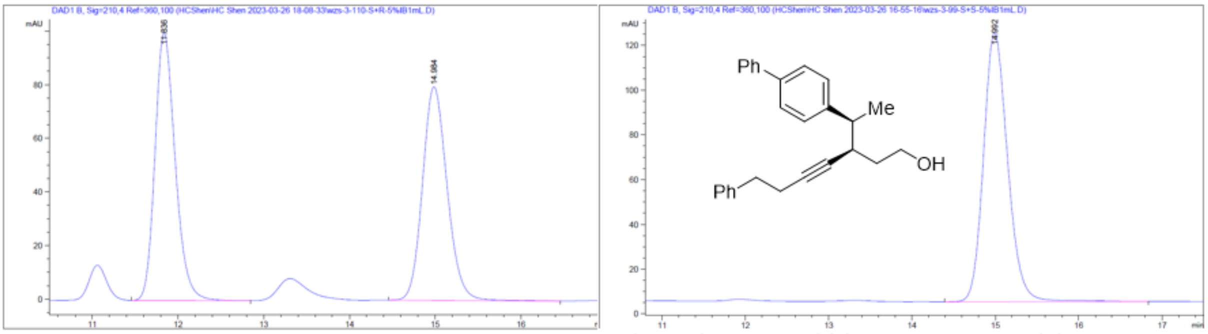

Peak #	RetTime [min]	Type	Width [min]	Area [mAU*s]	Height [mAU]	Area %
1	11.836	BB	0.2513	1638.43933	99.91245	50.7780
2	14.984	BB	0.3066	1588.23022	79.65270	49.2220

Peak #	RetTime [min]	Type	Width [min]	Area [mAU*s]	Height [mAU]	Area %
1	14.992	BB	0.3109	2420.95801	120.23963	100.0000

4-((2*S*,3*S*)-7-phenyl-3-vinylhept-4-yn-2-yl)-1,1'-biphenyl (21)

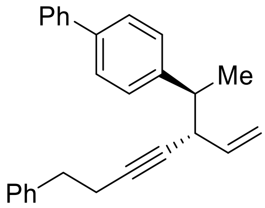

21

Prepared following **General Procedure A** using (*S*)-**1** and (*R*)-**L1**. Purification by flash column chromatography (0–1% DCM in *n*-hexane) gave the title compound (42.1 mg, 60%, >99% ee, 7.5:92.5 dr, >99:1 b:l) as a colorless oil.

^{1}H NMR (500 MHz, $CDCl_3$) δ 7.61 – 7.57 (m, 2H), 7.52 – 7.48 (m, 2H), 7.45 – 7.40 (m, 2H), 7.35 – 7.30 (m, 1H), 7.29 – 7.25 (m, 4H), 7.22 – 7.15 (m, 3H), 5.67 (ddd, *J* = 16.8, 10.0, 6.6 Hz, 1H), 5.19 (dt, *J* = 16.9, 1.6 Hz, 1H), 5.07 (dt, *J* = 10.0, 1.4 Hz, 1H), 3.32 – 3.25 (m, 1H), 3.01 – 2.92 (m, 1H), 2.78 (t, *J* = 7.5 Hz, 2H), 2.49 (td, *J* = 7.5, 2.3 Hz, 2H), 1.34 (d, *J* = 7.1 Hz, 3H) ppm. **^{13}C NMR** (126 MHz, $CDCl_3$) δ 143.2, 141.2, 141.0, 139.3, 136.5, 128.8, 128.7, 128.6, 128.5, 127.2, 126.7, 126.3, 116.5, 84.4, 80.4, 43.7, 43.3, 35.5, 21.1, 18.3 ppm. **Specific rotation** $[\alpha]_D^{24}$ = −78 (c = 2.0, CH_2Cl_2). **GC-MS** (EI) m/z calculated for $C_{27}H_{26}$ $[M]^+$, 350.2, found: 350.2. Note: HRMS (EI, ESI and APCI) was tried, but didn't find desired mass. **IR (neat)** 3027, 2967, 2926, 1638, 1602, 1486, 1453, 1076, 1008, 919, 842, 764, 733, 696 cm^{-1}.

The enantiomeric excess (ee) was determined after hydroboration/oxidation to alcohol **21'**

(*S*)-3-((*S*)-1-([1,1'-biphenyl]-4-yl)ethyl)-7-phenylhept-4-yn-1-ol (21')

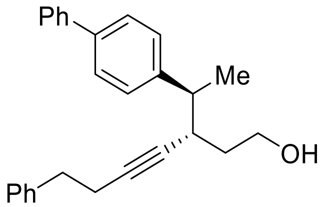

21'

Prepared following **General Procedure F (**0.1 mmol **21**, 7.5:92.5 dr, >99:1 b:l). Purification by flash column chromatography (0–30% EtOAc in *n*-hexane) gave the title compound (25.2

mg, 74%, >99% ee) based on major isomer as a pale yellow oil.

^{1}H NMR (500 MHz, $CDCl_3$) δ 7.61 – 7.56 (m, 2H), 7.52 – 7.46 (m, 2H), 7.45 – 7.40 (m, 2H), 7.35 – 7.31 (m, 1H), 7.31 – 7.26 (m, 4H), 7.24 – 7.16 (m, 3H), 3.76 – 3.58 (m, 2H), 2.92 – 2.83 (m, 1H), 2.83 – 2.72 (m, 3H), 2.54 – 2.43 (m, 2H), 1.72 – 1.61 (m, 1H), 1.48 (dd, *J* = 6.2, 5.0 Hz, 1H), 1.45 – 1.39 (m, 1H), 1.38 (d, *J* = 7.1 Hz, 3H) ppm. **^{13}C NMR** (126 MHz, $CDCl_3$) δ 143.1, 141.2, 140.9, 139.4, 128.9, 128.8, 128.6, 128.5, 127.1(7), 127.1(5), 126.8, 126.4, 83.6, 81.9, 61.6, 43.1, 35.8, 35.6, 35.4, 21.0, 19.1 ppm. **Specific rotation** $[\alpha]_D^{23}$ = −19 (c = 2.0, CH_2Cl_2). HRMS (APCI) m/z calculated for $C_{27}H_{29}O$ $[M+H]^+$, 369.2213, found: 369.2205. **IR (neat)** 3367, 3027, 2926, 1602, 1486, 1453, 1041, 1008, 841, 766, 736, 697 cm^{-1}. **HPLC conditions**: Chiral column IB, hexane: isopropanol = 95:5, flow rate = 1.0 mL/min, wavelength = 254 nm, t_R = 11.9 min for major isomer.

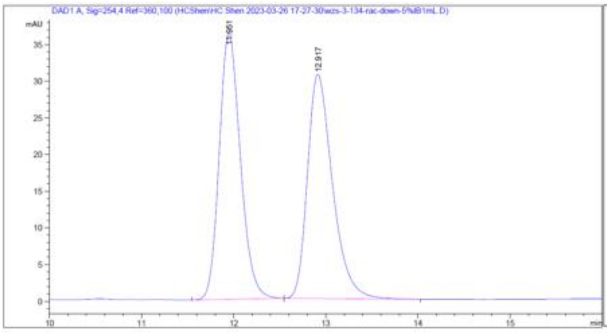

Peak #	RetTime [min]	Type	Width [min]	Area [mAU*s]	Height [mAU]	Area %
1	11.951	BB	0.2460	578.76990	36.29997	50.0671
2	12.917	BB	0.2883	577.21759	30.56258	49.9329

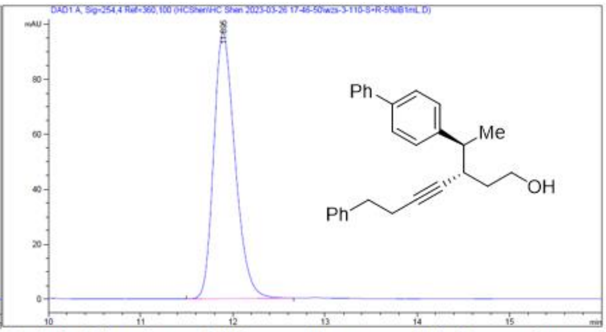

Peak #	RetTime [min]	Type	Width [min]	Area [mAU*s]	Height [mAU]	Area %
1	11.895	BB	0.2478	1556.12427	96.65546	100.0000

((2*R*,3*S*)-pent-4-ene-2,3-diyl)dibenzene (23)

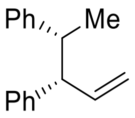

23

Prepared following **General Procedure A** using (*R*)-**22** and (*R*)-**L1**. Purification by flash column chromatography (0–1% DCM in *n*-hexane) gave the title compound (38.5 mg, 87%, >99% ee, 96.5:3.5 dr, 95.5:4.5 b:l) as a white solid.

^{1}H NMR (500 MHz, $CDCl_3$) δ 7.36 – 7.27 (m, 4H), 7.25 – 7.16 (m, 6H), 5.85 (ddd, *J* = 17.0, 10.3, 8.0 Hz, 1H), 4.87 – 4.81 (m, 1H), 4.78 – 4.71 (m, 1H), 3.45 – 3.38 (m, 1H), 3.11 – 3.02 (m, 1H), 1.10 (d, *J* = 7.0 Hz, 3H) ppm. **^{13}C NMR** (126 MHz, $CDCl_3$) δ 145.7, 143.5, 140.8, 128.6, 128.4, 128.3, 128.1, 126.4, 126.3, 115.5, 57.6, 45.6, 20.9 ppm. Specific rotation $[\alpha]_D^{24}$ = −71 (c = 2.0, CH_2Cl_2). **HRMS** (EI) m/z calculated for $C_{17}H_{18}$ $[M]^+$, 222.1403, found: 222.1401. **IR (neat)** 3024, 2957, 2925, 1637, 1600, 1492, 1450, 1003, 926, 753, 698 cm^{-1}.

The enantiomeric excess (ee) was determined after hydroboration/oxidation to alcohol **23'**.

(3*S*,4*R*)-3,4-diphenylpentan-1-ol (23')

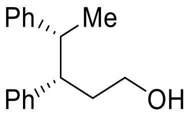

23'

Prepared following **General Procedure F (**0.1 mmol **23**, 96.5:3.5 dr, 95.5:4.5 b:l). Purification by flash column chromatography (0–30% EtOAc in *n*-hexane) gave the title compound (12.4 mg, 56%, >99% ee) based on major isomer as a white solid.

^{1}H NMR (500 MHz, $CDCl_3$) δ 7.36 – 7.29 (m, 4H), 7.26 – 7.19 (m, 6H), 3.37 – 3.28 (m, 1H), 3.28 – 3.18 (m, 1H), 2.92 – 2.77 (m, 2H), 1.74 – 1.60 (m, 2H), 1.00 (d, *J* = 6.7 Hz, 3H), 0.90 (t, *J* = 5.4 Hz, 1H) ppm. **^{13}C NMR** (126 MHz, $CDCl_3$) δ 146.5, 143.8, 128.6(4), 128.6(3), 128.4, 127.7, 126.6, 126.4, 61.5, 49.9, 46.5, 37.6, 21.2 ppm. **Specific rotation** $[\alpha]_D^{23}$ = −3 (c = 2.0, CH_2Cl_2). **HRMS** (APCI) m/z calculated for $C_{17}H_{17}$ $[M-H_2O-H_2+H]^+$, 221.1325, found: 221.1326. **IR (neat)** 3252, 3026, 2956, 2907, 1601, 1493, 1451, 1081, 1027, 756, 697 cm^{-1}. **HPLC conditions**: Chiral column IB, hexane: isopropanol = 97:3, flow rate = 1.0 mL/min, wavelength = 254 nm, t_R = 24.2 min for major isomer.

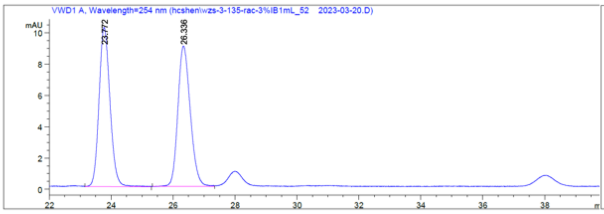

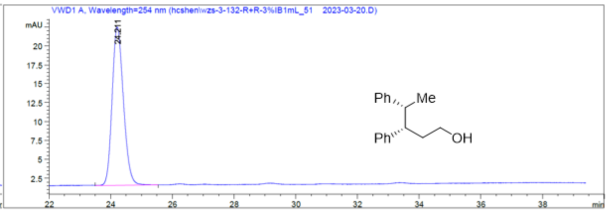

Peak #	RetTime [min]	Type	Width [min]	Area [mAU*s]	Height [mAU]	Area %
1	23.772	BB	0.3836	250.60646	10.17089	49.9683
2	26.336	BV	0.4250	250.92444	8.98127	50.0317

Peak #	RetTime [min]	Type	Width [min]	Area [mAU*s]	Height [mAU]	Area %
1	24.211	BB	0.3932	532.86450	20.92463	100.0000

((2*R*,3*R*)-pent-4-ene-2,3-diyl)dibenzene (24)

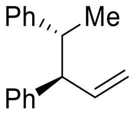

24

Prepared following **General Procedure A** using (*R*)-**22** and (*S*)-**L1**. Purification by flash column chromatography (0–1% DCM in *n*-hexane) gave the title compound (27.1 mg, 61%, >99% ee, 7:93 dr, 81:19 b:l) as a colorless oil.

^{1}H NMR (500 MHz, $CDCl_3$) δ 7.15 – 7.10 (m, 4H), 7.07 – 6.97 (m, 6H), 6.14 – 6.04 (m, 1H), 5.13 – 5.04 (m, 2H), 3.40 (t, *J* = 9.3 Hz, 1H), 3.11 – 3.02 (m, 1H), 1.33 (d, *J* = 7.0 Hz, 3H) ppm. **^{13}C NMR** (126 MHz, $CDCl_3$) δ 145.5, 143.6, 140.7, 128.2, 128.1, 128.0, 127.9, 126.0, 125.9, 115.8, 58.3, 45.1, 20.4 ppm. **Specific rotation** $[\alpha]_D^{22}$ = −9 (c = 2.0, CH_2Cl_2). **HRMS** (EI) m/z calculated for $C_{17}H_{18}$ $[M]^+$, 222.1403, found: 222.1400. **IR (neat)** 3027, 2952, 2925, 1601, 1493, 1451, 1375, 1325, 965, 911, 760, 696 cm^{-1}.

The enantiomeric excess (ee) was determined after hydroboration/oxidation to alcohol **24'**.

(3*R*,4*R*)-3,4-diphenylpentan-1-ol (24')

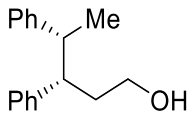

24'

Prepared following **General Procedure F (**0.1 mmol **24**, 7:93 dr, 81:19 b:l). Purification by flash column chromatography (0–30% EtOAc in *n*-hexane) gave the title compound (16.8 mg, 93%, >99% ee) based on major isomer as a colorless oil.

^{1}H NMR (400 MHz, $CDCl_3$) δ 7.20 – 7.03 (m, 6H), 7.00 – 6.91 (m, 4H), 3.56 – 3.44 (m, 1H), 3.43 – 3.31 (m, 1H), 3.05 – 2.87 (m, 2H), 2.20 – 2.07 (m, 1H), 1.94 – 1.81 (m, 1H), 1.33 (d, *J* = 6.8 Hz, 3H), 1.09 (t, *J* = 5.3 Hz, 1H) ppm. **^{13}C NMR** (101 MHz, $CDCl_3$) δ 145.3, 142.7, 128.8, 128.1, 128.0, 127.9, 126.2, 126.0, 61.6, 49.3, 45.8, 35.3, 18.9 ppm. **Specific rotation** $[\alpha]_D^{22}$ = −34 (c = 1.0, CH_2Cl_2). **HRMS** (APCI) m/z calculated for $C_{17}H_{17}$ $[M-H_2O-H_2+H]^+$, 221.1325, found: 221.1327. **IR (neat)** 3060, 3026, 2928, 1602, 1493, 1451, 1055, 1029, 762, 697 cm^{-1}. **HPLC conditions**: Chiral column IB, hexane: isopropanol = 97:3, flow rate = 1.0 mL/min, wavelength = 254 nm, t_R = 17.1 min for major isomer.

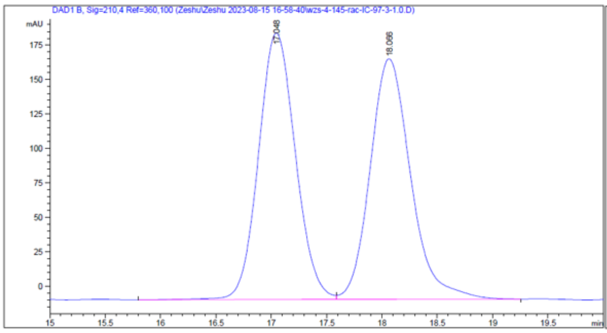

Peak #	RetTime [min]	Type	Width [min]	Area [mAU*s]	Height [mAU]	Area %
1	17.048	BV	0.3603	4484.86230	193.59477	50.2539
2	18.066	VB	0.3926	4439.54688	174.60744	49.7461

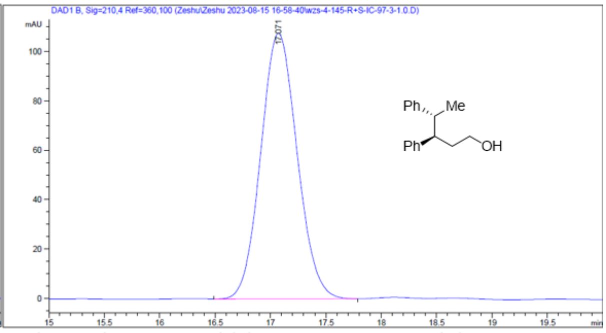

Peak #	RetTime [min]	Type	Width [min]	Area [mAU*s]	Height [mAU]	Area %
1	17.071	BB	0.3562	2454.76245	107.59516	100.0000

1-methoxy-4-((2*S*,3*R*)-3-phenylpent-4-en-2-yl)benzene (26)

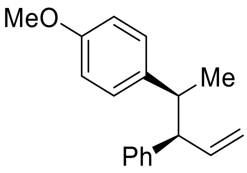

26

Prepared following **General Procedure A** using (*S*)-**25** and (*S*)-**L1**. Purification by flash column chromatography (0–20% DCM in *n*-hexane) gave the title compound (35.3 mg, 70%, >99% ee, 91.5:8.5 dr, 92:8 b:l) as a pale yellow solid.

^{1}H NMR (600 MHz, $CDCl_3$) δ 7.34 – 7.29 (m, 2H), 7.23 (d, *J* = 7.3 Hz, 1H), 7.20 – 7.16 (m, 2H), 7.11 – 7.06 (m, 2H), 6.87 – 6.82 (m, 2H), 5.85 (ddd, *J* = 17.0, 10.3, 8.1 Hz, 1H), 4.85 (ddd, *J* = 10.3, 1.7, 0.9 Hz, 1H), 4.75 (dt, *J* = 17.0, 1.3 Hz, 1H), 3.80 (s, 3H), 3.40 – 3.34 (m, 1H), 3.06 – 2.98 (m, 1H), 1.07 (d, *J* = 6.9 Hz, 3H) ppm. **^{13}C NMR** (151 MHz, $CDCl_3$) δ 158.0, 143.6, 140.9, 137.7, 128.9, 128.5, 128.4, 126.4, 115.5, 113.7, 57.7, 55.3, 44.7, 21.0 ppm. **Specific rotation** $[\alpha]_D^{21}$ = +62 (c = 2.0, CH_2Cl_2). **HRMS** (APCI) m/z calculated for $C_{18}H_{21}O$ $[M+H]^+$, 252.1587, found: 252.1585. **IR (neat)** 2958, 2834, 1611, 1512, 1453, 1247, 1177, 1036, 913, 838, 754, 700 cm^{-1}.

The enantiomeric excess (ee) was determined after hydroboration/oxidation to alcohol **26'**.

(3*R*,4*S*)-4-(4-methoxyphenyl)-3-phenylpentan-1-ol (26')

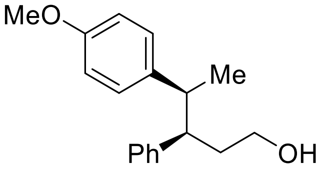

26'

Prepared following **General Procedure F (**0.1 mmol **26**, 91.5:8.5 dr, 92:8 b:l). Purification by flash column chromatography (0–30% EtOAc in *n*-hexane) gave the title compound (10.2 mg, 45%, >99% ee) based on major isomer as a white solid.

^{1}H NMR (600 MHz, $CDCl_3$) δ 7.35 – 7.29 (m, 2H), 7.25 – 7.18 (m, 3H), 7.18 – 7.12 (m, 2H), 6.91 – 6.84 (m, 2H), 3.81 (s, 3H), 3.36 – 3.30 (m, 1H), 3.27 – 3.20 (m, 1H), 2.87 – 2.79 (m, 1H), 2.79 – 2.71 (m, 1H), 1.74 – 1.60 (m, 2H), 1.27 – 1.13 (m, 1H), 0.97 (d, *J* = 6.8 Hz, 3H) ppm. **^{13}C NMR** (151 MHz, $CDCl_3$) δ 158.1, 143.9, 138.5, 128.6(1), 128.5(5), 128.4, 126.5, 114.0, 61.5, 55.4, 50.1, 45.6, 37.6, 21.3 ppm. **Specific rotation** $[\alpha]_D^{23}$ = +4 (c = 1.0, CH_2Cl_2). **HRMS** (APCI) m/z calculated for $C_{18}H_{21}O$ $[M-H_2O+H]^+$, 253.1587, found: 253.1584. **IR**

(neat) 3360, 2956, 2928, 1610, 1511, 1453, 1245, 1178, 1036, 830, 701 cm^{-1}. **HPLC conditions**: Chiral column IC, hexane: isopropanol = 90:10, flow rate = 1.0 mL/min, wavelength = 210 nm, t_R = 13.6 min for major isomer, t_R = 10.6 min for minor isomer.

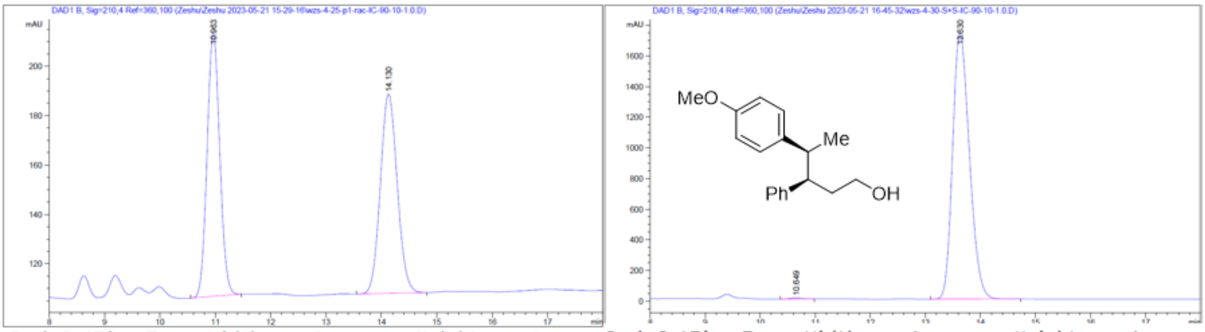

Peak #	RetTime [min]	Type	Width [min]	Area [mAU*s]	Height [mAU]	Area %
1	10.963	BB	0.2470	1692.95081	106.73961	49.6816
2	14.130	BB	0.3297	1714.65369	80.74683	50.3184

Peak #	RetTime [min]	Type	Width [min]	Area [mAU*s]	Height [mAU]	Area %
1	10.649	BB	0.2307	113.39633	7.75226	0.3058
2	13.630	BB	0.3302	3.69656e4	1737.26929	99.6942

1-methoxy-4-((2*S*,3*S*)-3-phenylpent-4-en-2-yl)benzene (27)

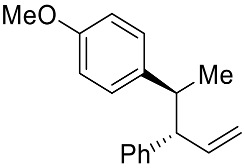

27

Prepared following **General Procedure A** using (*S*)-**25** and (*R*)-**L1**. Purification by flash column chromatography (0–20% DCM in *n*-hexane) gave the title compound (30.9 mg, 61%, >99% ee, 11:89 dr, 88:12 b:l) as a colorless oil.

^{1}H NMR (600 MHz, $CDCl_3$) δ 7.15 (t, *J* = 7.5 Hz, 2H), 7.06 (s, 1H), 7.00 (d, *J* = 8.2 Hz, 2H), 6.92 (d, *J* = 8.6 Hz, 2H), 6.69 (d, *J* = 8.6 Hz, 2H), 6.14 – 6.04 (m, 1H), 5.12 – 5.05 (m, 2H), 3.72 (s, 3H), 3.40 – 3.33 (m, 1H), 3.08 – 2.99 (m, 1H), 1.31 (d, *J* = 7.0 Hz, 3H) ppm. **^{13}C NMR** (151 MHz, $CDCl_3$) δ 157.7, 143.6, 140.8, 137.5, 128.7, 128.2, 126.0, 115.6, 113.4, 58.5, 55.2, 44.3, 20.5 ppm. **Specific rotation** $[\alpha]_D^{21}$ = −13 (c = 2.0, CH_2Cl_2). **HRMS** (APCI) m/z calculated for $C_{18}H_{21}O$ $[M+H]^+$, 252.1587, found: 252.1583. **IR (neat)** 3028, 2960, 1611, 1512, 1452, 1246, 1178, 1037, 913, 829, 760, 699 cm^{-1}.

The enantiomeric excess (ee) was determined after hydroboration/oxidation to alcohol **27'**.

(3*S*,4*S*)-4-(4-methoxyphenyl)-3-phenylpentan-1-ol (27')

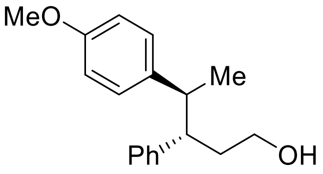

27'

Prepared following **General Procedure F (**0.1 mmol **27**, 11:89 dr, 88:12 b:l). Purification by flash column chromatography (0–30% EtOAc in *n*-hexane) gave the title compound (8.9 mg, 42%, >99% ee) based on major isomer as a colorless oil.

^{1}H NMR (600 MHz, $CDCl_3$) δ 7.19 – 7.14 (m, 2H), 7.14 – 7.08 (m, 1H), 6.94 (d, J = 6.9 Hz, 2H), 6.90 – 6.84 (m, 2H), 6.72 – 6.67 (m, 2H), 3.74 (s, 3H), 3.53 – 3.46 (m, 1H), 3.41 – 3.34 (m, 1H), 2.99 – 2.92 (m, 1H), 2.92 – 2.85 (m, 1H), 2.15 – 2.06 (m, 1H), 1.92 – 1.83 (m, 1H), 1.33 (s, 1H), 1.29 (d, J = 7.0 Hz, 3H) ppm. **^{13}C NMR** (151 MHz, $CDCl_3$) δ 157.8, 142.7, 137.3, 129.0, 128.9, 128.0, 126.2, 113.3, 61.6, 55.3, 49.4, 44.8, 35.3, 19.1 ppm. **Specific rotation** $[\alpha]_D^{23}$ = +18 (c = 1.0, CH_2Cl_2). **HRMS** (APCI) m/z calculated for $C_{18}H_{21}O$ $[M-H_2O+H]^+$, 253.1587, found: 253.1585. **IR (neat)** 3354, 3027, 2932, 1611, 1511, 1462, 1245, 1178, 1036, 829, 701 cm^{-1}. **HPLC conditions**: Chiral column IC, hexane: isopropanol = 95:5, flow rate = 1.0 mL/min, wavelength = 254 nm, t_R = 18.0 min for major isomer.

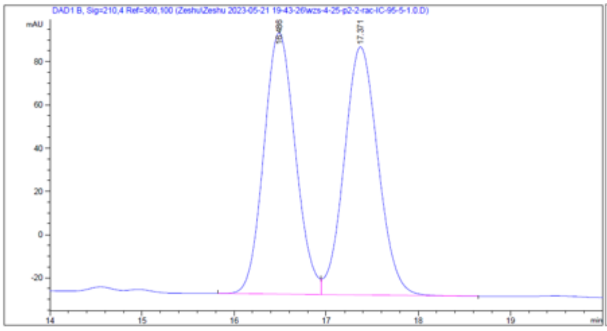

Peak #	RetTime [min]	Type	Width [min]	Area [mAU*s]	Height [mAU]	Area %
1	16.486	BV	0.3696	2875.55664	120.85841	49.5762
2	17.371	VB	0.3953	2924.72095	114.75753	50.4238

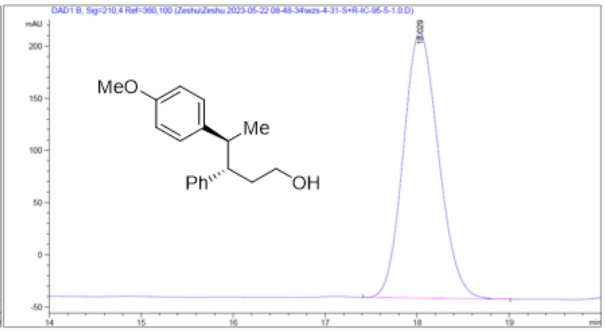

Peak #	RetTime [min]	Type	Width [min]	Area [mAU*s]	Height [mAU]	Area %
1	18.029	BB	0.4142	6759.77490	254.22166	100.0000

1-((2*S*,3*R*)-3-phenylpent-4-en-2-yl)-4-(trifluoromethyl)benzene (29)

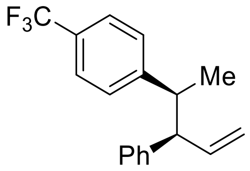

29

Prepared following **General Procedure C** using (*S*)-**28** and (*S*)-**L1**. Purification by flash column chromatography (0–1% DCM in *n*-hexane) gave the title compound (31.0 mg, 53%, >99% ee, 97.5:2.5 dr, 97.5:2.5 b:l) as a colorless oil.

^{1}H NMR (600 MHz, $CDCl_3$) δ 7.54 (d, *J* = 8.0 Hz, 2H), 7.35 – 7.31 (m, 2H), 7.28 (d, *J* = 8.1 Hz, 2H), 7.26 – 7.22 (m, 1H), 7.21 – 7.17 (m, 2H), 5.80 (ddd, *J* = 17.0, 10.3, 8.2 Hz, 1H), 4.85 (ddd, *J* = 10.3, 1.5, 0.8 Hz, 1H), 4.76 (dt, *J* = 17.0, 1.3 Hz, 1H), 3.44 – 3.38 (m, 1H), 3.17 – 3.09 (m, 1H), 1.10 (d, *J* = 6.9 Hz, 3H) ppm. **^{13}C NMR** (151 MHz, $CDCl_3$) δ 149.8, 142.9, 140.1, 128.7, 128.6 (q, *J* = 31.7 Hz), 128.4, 128.3, 126.7, 125.3 (q, *J* = 3.7 Hz), 124.5 (q, *J* = 271.8 Hz), 116.1, 57.4, 45.5, 20.6 ppm. **Specific rotation** $[\alpha]_D^{21}$ = +57 (c = 2.0, CH_2Cl_2). **HRMS** (EI) m/z calculated for $C_{18}H_{17}F_3$ $[M]^+$, 290.1277, found: 290.1273. **IR (neat)** 3029, 2974, 1619, 1419, 1325, 1163, 1120, 1069, 1016, 847, 700 cm^{-1}.

The enantiomeric excess (ee) was determined after hydroboration/oxidation to alcohol **29'**.

(3*R*,4*S*)-3-phenyl-4-(4-(trifluoromethyl)phenyl)pentan-1-ol (29')

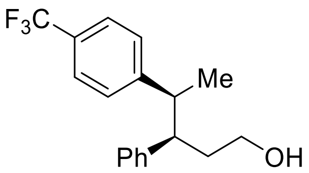

29'

Prepared following **General Procedure F (**0.1 mmol **29**, 97.5:2.5 dr, 97.5:2.5 b:l). Purification by flash column chromatography (0–30% EtOAc in *n*-hexane) gave the title compound (24.6 mg, 84%, >99% ee) based on major isomer as a white solid.

^{1}H NMR (600 MHz, $CDCl_3$) δ 7.58 (d, *J* = 8.0 Hz, 2H), 7.38 – 7.31 (m, 4H), 7.27 – 7.23 (m, 1H), 7.21 (d, *J* = 6.9 Hz, 2H), 3.38 – 3.31 (m, 1H), 3.27 – 3.20 (m, 1H), 2.99 – 2.91 (m, 1H), 2.85 (td, *J* = 10.6, 4.0 Hz, 1H), 1.63 (s, 2H), 1.33 – 1.15 (m, 1H), 1.01 (d, *J* = 6.9 Hz, 3H) ppm. **^{13}C NMR** (151 MHz, $CDCl_3$) δ 150.6, 143.0, 128.7(4), 128.7(2) (q, *J* = 33.2 Hz), 128.4, 128.1, 126.8, 125.6 (q, *J* = 3.8 Hz), 124.5 (q, *J* = 271.8 Hz), 61.2, 49.5, 46.4, 37.3, 21.0 ppm. **Specific rotation** $[\alpha]_D^{23}$ = +4 (c = 2.0, CH_2Cl_2). **LC-MS** (EI) m/z calculated for $C_{18}H_{17}F_3$ $[M-H_2O]^+$, 290.1, found: 290.1. Note: HRMS (EI, ESI and APCI) was tried, but didn't find desired mass. **IR (neat)** 3318, 3028, 2931, 1618, 1454, 1420, 1324, 1162, 1119, 1068, 841, 701. **HPLC conditions**: Chiral column IC, hexane: isopropanol = 95:5, flow rate = 1.0 mL/min, wavelength = 210 nm, t_R = 10.5 min for major isomer, t_R = 8.1 min for minor isomer.

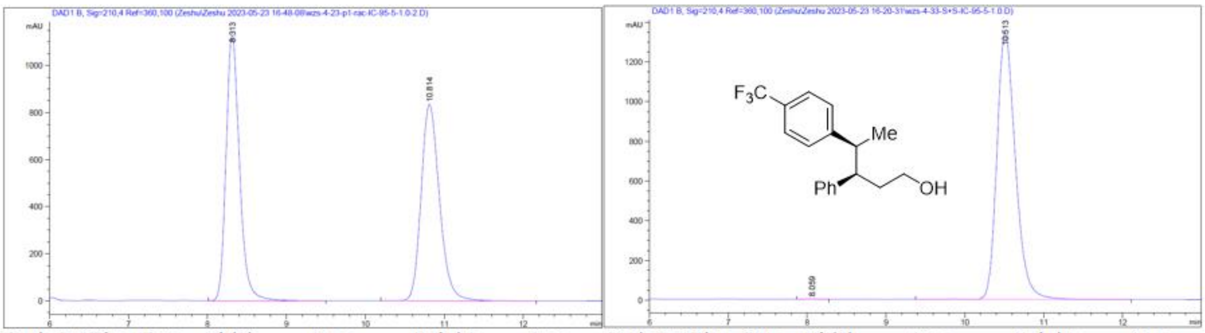

Peak #	RetTime [min]	Type	Width [min]	Area [mAU*s]	Height [mAU]	Area %
1	8.313	BB	0.1828	1.34267e4	1128.53186	49.9019
2	10.814	BB	0.2503	1.34795e4	835.02472	50.0981

Peak #	RetTime [min]	Type	Width [min]	Area [mAU*s]	Height [mAU]	Area %
1	8.059	BB	0.1619	21.41159	2.08304	0.0973
2	10.513	VB R	0.2531	2.19768e4	1340.28540	99.9027

1-((2*S*,3*S*)-3-phenylpent-4-en-2-yl)-4-(trifluoromethyl)benzene (30)

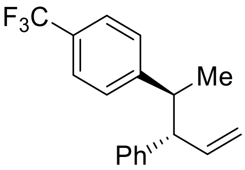

30

Prepared following **General Procedure C** using (*S*)-**28** and (*R*)-**L1**. Purification by flash column chromatography (0–1% DCM in *n*-hexane) gave the title compound (27.8 mg, 48%, >99% ee, 4.5:95.5 dr, 95:5 b:l) as a colorless oil.

^{1}H NMR (600 MHz, $CDCl_3$) δ 7.38 (d, *J* = 8.1 Hz, 2H), 7.17 – 7.09 (m, 4H), 7.09 – 7.04 (m, 1H), 7.00 – 6.95 (m, 2H), 6.07 (ddd, *J* = 16.7, 10.3, 9.2 Hz, 1H), 5.14 – 5.08 (m, 2H), 3.42 – 3.36 (m, 1H), 3.18 – 3.09 (m, 1H), 1.34 (d, *J* = 6.9 Hz, 3H) ppm. **^{13}C NMR** (151 MHz, $CDCl_3$) δ 149.7, 142.9, 140.2, 128.4, 128.1(9) (q, *J* = 31.7 Hz), 128.1(6), 128.0, 126.3, 125.0 (q, *J* = 3.7 Hz), 124.4 (q, *J* = 271.8 Hz), 116.2, 58.1, 45.1, 20.3 ppm. **Specific rotation** $[\alpha]_D^{21}$ = −19 (c = 2.0, CH_2Cl_2). **HRMS** (EI) m/z calculated for $C_{18}H_{17}F_3$ $[M]^+$, 290.1277, found: 290.1273. **IR (neat)** 3029, 2974, 2930, 1619, 1453, 1420, 1324, 1162, 1118, 1069, 1017, 839, 699 cm^{-1}.

The enantiomeric excess (ee) was determined after hydroboration/oxidation to alcohol **30'**.

(3*S*,4*S*)-3-phenyl-4-(4-(trifluoromethyl)phenyl)pentan-1-ol (30')

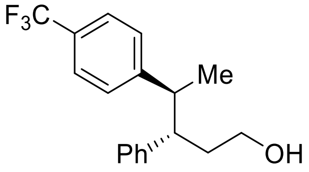

30'

Prepared following **General Procedure F (**0.08 mmol **30**, 4.5:95.5 dr, 95:5 b:l). Purification by flash column chromatography (0–30% EtOAc in *n*-hexane) gave the title compound (3.2 mg, 14%, >99% ee) based on major isomer as a colorless oil.

^{1}H NMR (600 MHz, $CDCl_3$) δ 7.38 (d, *J* = 8.1 Hz, 2H), 7.18 – 7.13 (m, 2H), 7.12 – 7.08 (m, 1H), 7.05 (d, *J* = 8.0 Hz, 2H), 6.95 – 6.90 (m, 2H), 3.55 – 3.47 (m, 1H), 3.41 – 3.34 (m, 1H), 3.09 – 3.01 (m, 1H), 2.97 – 2.90 (m, 1H), 2.20 – 2.12 (m, 1H), 1.92 – 1.83 (m, 1H), 1.35 (d, *J* = 7.0 Hz, 3H), 1.11 (s, 1H) ppm. **^{13}C NMR** (151 MHz, $CDCl_3$) δ 149.6, 142.1, 128.6, 128.4, 128.2(4), 128.2(2) (q, *J* = 31.7 Hz), 126.5, 124.9 (q, *J* = 3.7 Hz), 124.4 (q, *J* = 271.8 Hz), 61.3, 49.2, 45.9, 35.5, 19.2 ppm. **Specific rotation** $[\alpha]_D^{23}$ = +14 (c = 1.0, CH_2Cl_2). **LC-MS** (EI) m/z calculated for $C_{18}H_{17}F_3$ $[M-H_2O]^+$, 290.1, found: 290.1. Note: HRMS (EI, ESI and APCI) was tried, but didn't find desired mass. **IR (neat)** 3336, 2931, 2875, 1618, 1453, 1420, 1325, 1153, 1118, 1069, 839, 701 cm^{-1}. **HPLC conditions**: Chiral column IB, hexane: isopropanol = 97:3, flow rate = 1.0 mL/min, wavelength = 210 nm, t_R = 16.3 min for major isomer.

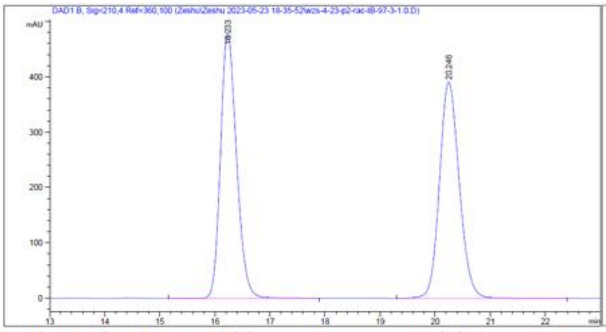

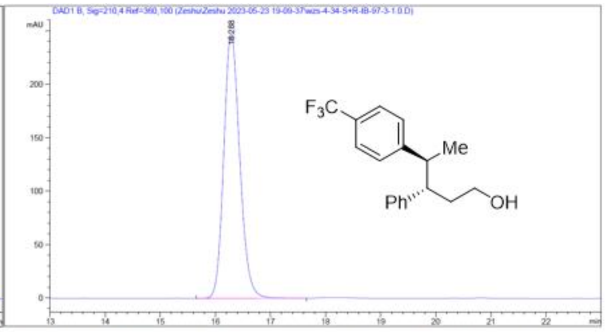

Peak #	RetTime [min]	Type	Width [min]	Area [mAU*s]	Height [mAU]	Area %
1	16.233	BB	0.3116	9681.85645	479.39713	49.8465
2	20.246	BB	0.3849	9741.49414	390.67099	50.1535

Peak #	RetTime [min]	Type	Width [min]	Area [mAU*s]	Height [mAU]	Area %
1	16.288	BB	0.3109	4996.11084	248.10095	100.0000

(*S*)-1-methoxy-4-(1-phenylbut-3-en-2-yl)benzene (31)

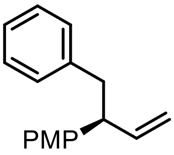

31

Prepared following **General Procedure B**. Purification by flash column chromatography (5% EtOAc in *n*-hexane) gave the title compound (33.1 mg, 68%, 92% ee) as a colorless oil.

^{1}H NMR (400 MHz, $CDCl_3$): δ 7.28 – 7.19 (m, 2H), 7.21 – 7.12 (m, 1H), 7.11 – 7.05 (m, 4H), 6.88 – 6.80 (m, 2H), 6.03 (ddd, *J* = 17.3, 10.3, 7.2 Hz, 1H), 5.02 (ddd, *J* = 10.3, 1.6, 1.1 Hz, 1H), 4.96 (dt, *J* = 17.1, 1.5 Hz, 1H), 3.80 (s, 3H), 3.55 (q, *J* = 7.4 Hz, 1H), 3.08 – 2.95 (m, 2H) ppm; **^{13}C NMR** (101 MHz, $CDCl_3$): δ 158.2, 141.8, 140.3, 135.8, 129.4, 128.9, 128.2, 126.0, 114.5, 113.9, 55.4, 50.8, 42.4 ppm. **Specific rotation** $[\alpha]_D^{24}$ = −44 (c =1.41, CH_2Cl_2). **HRMS** (EI) m/z calculated for $C_{17}H_{18}O$ $[M]^+$, 238.1352; found, 238.1349. **IR (neat)** 3027, 2934, 2834, 1636, 1610, 1510, 1454, 1246, 1177, 1034, 829, 698, 534 cm^{-1}. **HPLC conditions**: Chiral column IC, hexane: isopropanol = 99:1, flow rate = 0.5 mL/min, wavelength = 230 nm, t_R = 9.6 min for major isomer, t_R = 9.0 min for minor isomer.

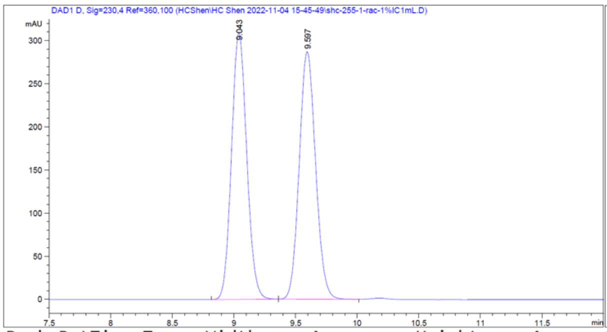

Peak #	RetTime [min]	Type	Width [min]	Area [mAU*s]	Height [mAU]	Area %
1	9.043	BB	0.1342	2655.39331	309.06516	50.0675
2	9.597	BB	0.1436	2648.23022	287.06830	49.9325

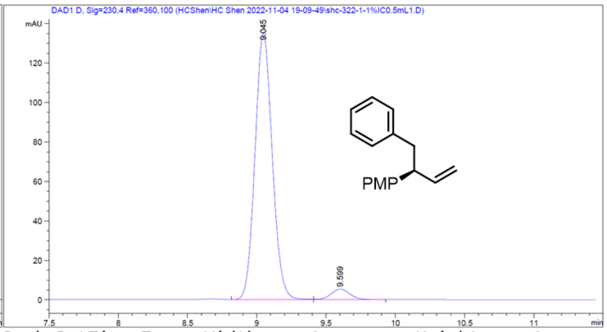

Peak #	RetTime [min]	Type	Width [min]	Area [mAU*s]	Height [mAU]	Area %
1	9.045	BV	0.1351	1172.51440	135.29562	95.9444
2	9.599	VB	0.1450	49.56245	5.30400	4.0556

(*S*)-1-(2-(4-methoxyphenyl)but-3-en-1-yl)-2-methylbenzene (32)

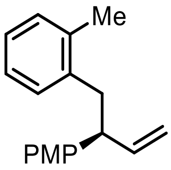

32

Prepared following **General Procedure B**. Purification by flash column chromatography (5% EtOAc in *n*-hexane) gave the title compound (42.2 mg, 84%, 95% ee) as a colorless oil.

^{1}H NMR (400 MHz, $CDCl_3$): δ 7.14 – 7.00 (m, 5H), 7.01 – 6.92 (m, 1H), 6.86 – 6.78 (m, 2H), 6.05 (ddd, *J* = 17.3, 10.3, 7.2 Hz, 1H), 5.01 (dt, *J* = 10.3, 1.4 Hz, 1H), 4.93 (dt, *J* = 17.1, 1.5 Hz, 1H), 3.79 (s, 3H), 3.56 – 3.46 (m, 1H), 3.08 – 2.88 (m, 2H), 2.24 (s, 3H) ppm; **^{13}C NMR** (101 MHz, $CDCl_3$): δ 158.2, 141.7, 138.5, 136.4, 136.1, 130.2, 128.8, 126.1, 125.6, 114.5, 113.9, 55.4, 49.6, 39.7, 19.7 ppm. **HRMS** (EI) m/z calculated for $C_{18}H_{20}O$ $[M]^+$, 252.1509; found, 252.1507. **IR (neat)** 2934, 2834, 1610, 1510, 1463, 1248, 1177, 1034, 828, 747, 537 cm^{-1}.

The enantiomeric excess (ee) was determined after hydroboration/oxidation to alcohol **32'**.

(*S*)-3-(4-methoxyphenyl)-4-(*o*-tolyl)butan-1-ol (32')

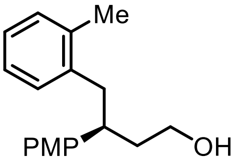

32'

Prepared following **General Procedure F** (0.17 mmol **32**). Purification by flash column chromatography (30% EtOAc in *n*-hexane) gave the title compound (37.2 mg, 81%, 95% ee) as a colorless oil.

^{1}H NMR (400 MHz, $CDCl_3$): δ 7.14 – 7.00 (m, 5H), 6.99 – 6.94 (m, 1H), 6.86 – 6.78 (m, 2H), 3.79 (s, 3H), 3.53 – 3.51 (m, 1H), 3.44 – 3.42 (m, 1H), 2.99 – 2.88 (m, 1H), 2.91 – 2.79 (m, 2H), 2.24 (s, 3H), 2.02 – 1.97 (m, 1H), 1.93 – 1.83 (m, 1H) ppm; **^{13}C NMR** (101 MHz, $CDCl_3$): δ 158.2, 138.8, 136.7, 136.3, 130.3, 130.2, 128.6, 126.2, 125.7, 114.0, 61.4, 55.4, 42.6, 41.5, 38.4, 19.7 ppm. **Specific rotation** $[\alpha]_D^{24}$ = −69 (c = 0.75, CH_2Cl_2). **HRMS** (EI) m/z calculated for $C_{18}H_{22}O_2$ $[M]^+$, 270.1614; found, 270.1611. **IR (neat)** 3350, 2932, 1610, 1583, 1511, 1301, 1246, 1033, 828, 744, 578, 455 cm^{-1}. **HPLC conditions**: Chiral column IA, hexane: isopropanol = 90:10, flow rate = 1.0 mL/min, wavelength = 230 nm, t_R = 7.1 min for major isomer, t_R = 7.7 min for minor isomer.

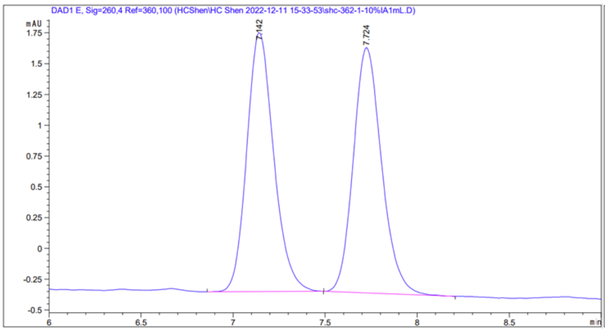

Peak #	RetTime [min]	Type	Width [min]	Area [mAU*s]	Height [mAU]	Area %
1	7.142	BB	0.1566	21.37178	2.10246	50.0810
2	7.724	BB	0.1647	21.30266	1.99249	49.9190

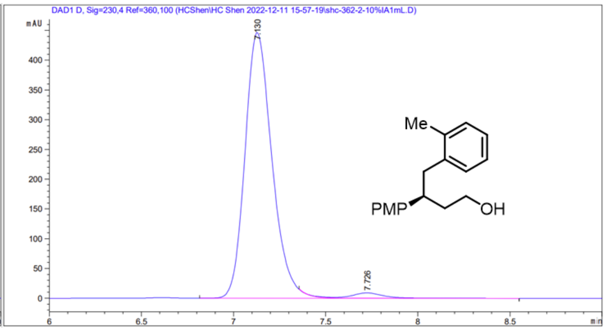

Peak #	RetTime [min]	Type	Width [min]	Area [mAU*s]	Height [mAU]	Area %
1	7.130	BV R	0.1568	4547.64648	446.77533	97.4857
2	7.726	VB E	0.1913	117.28883	9.04669	2.5143

(*S*)-4,4'-(but-3-ene-1,2-diyl)bis(methoxybenzene) (33)

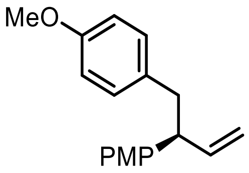

33

Prepared following **General Procedure B**. Purification by flash column chromatography (30% EtOAc in *n*-hexane) gave the title compound (35.9 mg, 67%, 92% ee) as a colorless oil.

^{1}H NMR (400 MHz, $CDCl_3$): δ 7.11 – 7.03 (m, 2H), 7.02 – 6.94 (m, 2H), 6.87 – 6.79 (m, 2H), 6.81 – 6.73 (m, 2H), 6.02 (ddd, *J* = 17.3, 10.3, 7.2 Hz, 1H), 5.02 (ddd, *J* = 10.3, 1.6, 1.1 Hz, 1H), 4.98 – 4.93 (m, 1H), 3.79 (s, 3H), 3.77 (s, 3H), 3.50 (q, *J* = 7.5 Hz, 1H), 3.03 – 2.87 (m, 2H) ppm; **^{13}C NMR** (101 MHz, $CDCl_3$): δ 158.1, 157.9, 141.9, 135.9, 132.4, 130.3, 128.9, 114.4, 113.9, 113.6, 55.3(4), 55.2(9), 51.0, 41.5 ppm. **Specific rotation** $[\alpha]_D^{24}$ = −50 (c = 0.75, CH_2Cl_2). **HRMS** (EI) m/z calculated for $C_{18}H_{20}O_2$ $[M]^+$, 268.1458; found, 268.1457. **IR (neat)** 2934, 2834, 1635, 1811, 1509, 1464, 1243, 1176, 1033, 829, 654, 536 cm^{-1}. **HPLC conditions**: Chiral column IB, hexane: isopropanol = 99:1, flow rate = 1.0 mL/min, wavelength = 210 nm, t_R = 6.1 min for major isomer, t_R = 6.6 min for minor isomer.

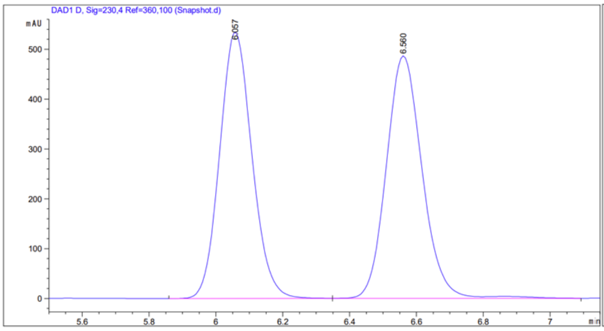

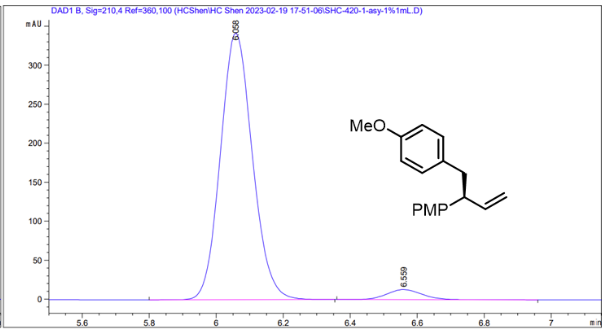

Peak #	RetTime [min]	Type	Width [min]	Area [mAU*s]	Height [mAU]	Area %
1	6.057	BB	0.1041	3575.51904	534.26398	49.6916
2	6.560	BV R	0.1160	3619.89429	486.07764	50.3084

Peak #	RetTime [min]	Type	Width [min]	Area [mAU*s]	Height [mAU]	Area %
1	6.058	BB	0.1033	2266.97559	342.07538	96.0224
2	6.559	BB	0.1133	93.90578	12.83717	3.9776

(*S*)-1-fluoro-2-(2-(4-methoxyphenyl)but-3-en-1-yl)benzene (34)

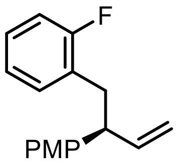

34

Prepared following **General Procedure B**. Purification by flash column chromatography (5% EtOAc in *n*-hexane) gave the title compound (34.1 mg, 61%, 96% ee) as a colorless oil.

^{1}H NMR (400 MHz, $CDCl_3$): δ 7.18 – 7.07 (m, 3H), 7.02-6.96 (m, 3H), 6.84-6.82 (m, 2H), 6.09 – 5.95 (m, 1H), 5.03-5.00 (m, 1H), 4.98-4.93 (m, 1H), 3.79 (d, *J* = 2.6 Hz, 3H), 3.59 (q, *J* = 7.5 Hz, 1H), 3.12 – 2.93 (m, 2H) ppm;**^{13}C NMR** (101 MHz, $CDCl_3$): δ 161.4 (d, *J* = 244.6 Hz), 158.2, 141.4, 135.7, 131.6 (d, *J* = 5.0 Hz), 128.7, 127.8 (d, *J* = 8.1 Hz), 127.2 (d, *J* = 15.5 Hz), 123.7 (d, *J* = 3.5 Hz), 115.2 (d, *J* = 22.4 Hz), 114.7, 113.9, 55.4, 49.5 (d, *J* = 1.3 Hz), 35.5 (d, *J* = 1.4 Hz) ppm. **HRMS** (EI) m/z calculated for $C_{17}H_{19}$ $[M]^+$, 256.1258; found, 256.1256. **IR (neat)** 3357, 2936, 2844, 1611, 1584, 1512, 1247, 1033, 829, 756, 544 cm^{-1}.

The enantiomeric excess (ee) was determined after hydroboration/oxidation to alcohol **34'**.

(*S*)-4-(2-fluorophenyl)-3-(4-methoxyphenyl)butan-1-ol (34')

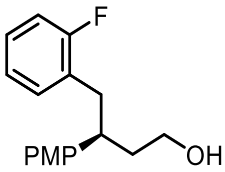

34'

Prepared following **General Procedure F (**0.13 mmol **34**). Purification by flash column chromatography (30% EtOAc in *n*-hexane) gave the title compound (32.7 mg, 64%, 96% ee) as a colorless oil.

^{1}H NMR (400 MHz, $CDCl_3$): δ 7.16 – 7.08 (m, 1H), 7.07 (dd, *J* = 9.0, 2.5 Hz, 2H), 7.01 – 6.91 (m, 3H), 6.84 – 6.77 (m, 2H), 3.78 (s, 3H), 3.57 – 3.50 (m, 1H), 3.48 – 3.40 (m, 1H), 3.07 – 2.91 (m, 2H), 2.87 – 2.82 (m, 1H), 1.99 – 1.92 (m, 1H), 1.92 – 1.81 (m, 1H) ppm; **^{13}C NMR** (101 MHz, $CDCl_3$): δ 161.3 (d, *J* = 244.6 Hz), 158.2, 136.1, 131.6 (d, *J* = 5.0 Hz), 128.6, 127.8 (d, *J* = 8.1 Hz), 127.4 (d, *J* = 15.7 Hz), 123.8 (d, *J* = 3.5 Hz), 115.2 (d, *J* = 22.4 Hz), 113.9, 61.3, 55.3, 42.5 (d, *J* = 1.2 Hz), 38.4, 37.0 (d, *J* = 1.6 Hz) ppm. **Specific rotation** $[\alpha]_D^{24}$ = −72 (c = 0.67, CH_2Cl_2). **HRMS** (EI) m/z calculated for $C_{17}H_{19}O_2F$ $[M]^+$, 274.1364; found, 274.1362. **IR (neat)** 3350, 2935, 1611, 1512, 1247, 1179, 1033, 829, 755, 544 cm^{-1}. **HPLC conditions**: Chiral column IC, hexane: isopropanol = 90:10, flow rate = 1.0 mL/min, wavelength = 210 nm, t_R = 10.4 min for major isomer, t_R = 9.3 min for minor isomer.

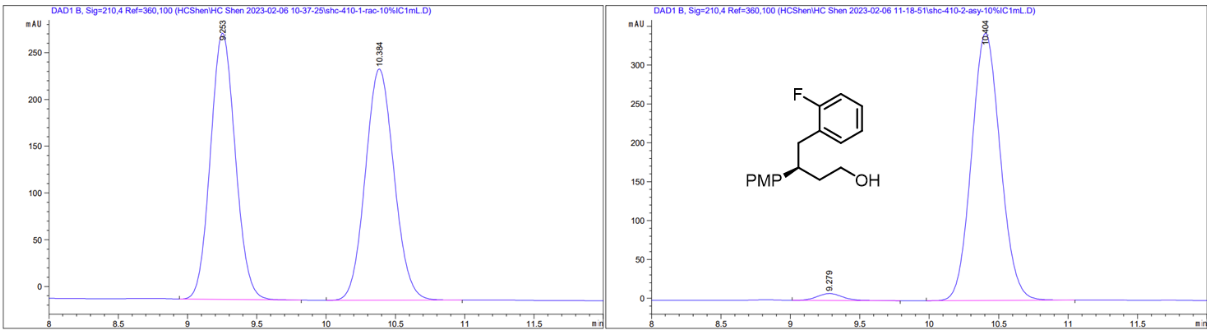

Peak #	RetTime [min]	Type	Width [min]	Area [mAU*s]	Height [mAU]	Area %
1	9.253	BB	0.1933	3545.35669	284.61749	49.9435
2	10.384	BB	0.2237	3553.37842	247.16794	50.0565

Peak #	RetTime [min]	Type	Width [min]	Area [mAU*s]	Height [mAU]	Area %
1	9.279	BB	0.1969	111.33693	8.84237	2.2037
2	10.404	BB	0.2237	4940.85938	343.73541	97.7963

(*S*)-1-(8-bromooct-1-en-3-yl)-4-methoxybenzene (35)

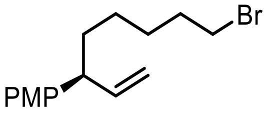

35

Prepared following **General Procedure D**. Purification by flash column chromatography (15% DCM in *n*-hexane) gave the title compound (35.8 mg, 61%, 99% ee) as a colorless oil.

^{1}H NMR (400 MHz, $CDCl_3$): δ 7.14 – 7.06 (m, 2H), 6.89 – 6.81 (m, 2H), 5.99 – 5.85 (m, 1H), 5.05 – 4.99 (m, 1H), 5.01 – 4.95 (m, 1H), 3.79 (s, 3H), 3.38 (t, *J* = 6.8 Hz, 2H), 3.19 (q, *J* = 7.4 Hz, 1H), 1.89 – 1.77 (m, 2H), 1.77 – 1.60 (m, 2H), 1.52 – 1.36 (m, 2H), 1.36 – 1.15 (m, 2H) ppm; **^{13}C NMR** (101 MHz, $CDCl_3$): δ 158.1, 142.8, 136.6, 128.6, 114.0, 113.8, 55.4, 49.0, 35.4, 34.0, 32.8, 28.2, 26.8 ppm. **HRMS** (EI) m/z calculated for $C_{15}H_{21}OBr$ $[M]^+$, 296.0770; found, 296.0767. **IR (neat)** 2932, 2857, 1610, 1511, 1463, 1246, 1178, 1034, 829, 750, 656, 541 cm^{-1}.

The enantiomeric excess (ee) was determined after hydroboration/oxidation to alcohol **35'**.

(*S*)-8-bromo-3-(4-methoxyphenyl)octan-1-ol (35')

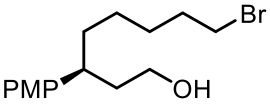

35'

Prepared following **General Procedure F** (0.12 mmol **35**). Purification by flash column

chromatography (30% EtOAc in *n*-hexane) gave the title compound (29.7 mg, 79%, 99% ee) as a colorless oil.

^{1}H NMR (400 MHz, $CDCl_3$): δ 7.11 – 7.03 (m, 2H), 6.88 – 6.80 (m, 2H), 3.79 (s, 3H), 3.55 – 3.42 (m, 2H), 3.34 (t, *J* = 6.8 Hz, 2H), 2.69 – 2.58 (m, 1H), 1.94-1.86 (m, 1H), 1.83 – 1.70 (m, 3H), 1.67 – 1.50 (m, 2H), 1.43 – 1.29 (m, 2H), 1.28 – 1.08 (m, 2H) ppm; **^{13}C NMR** (101 MHz, $CDCl_3$): δ 158.1, 137.0, 128.5, 114.0, 61.3, 55.4, 41.7, 39.9, 37.0, 34.1, 32.8, 28.3, 26.8 ppm. **Specific rotation** $[\alpha]_D^{24}$ = −10 (c = 0.62, CH_2Cl_2). **HRMS** (EI) m/z calculated for $C_{15}H_{23}O_2Br$ $[M]^+$, 314.0876; found, 314.0872. **IR (neat)** 3351, 2929, 2856, 1610, 1510, 1462, 1245, 1033, 830, 749, 644, 559 cm^{-1}. **HPLC conditions**: Chiral column IC, hexane: isopropanol = 95:5, flow rate = 1.0 mL/min, wavelength = 214 nm, t_R = 20.8 min for major isomer.

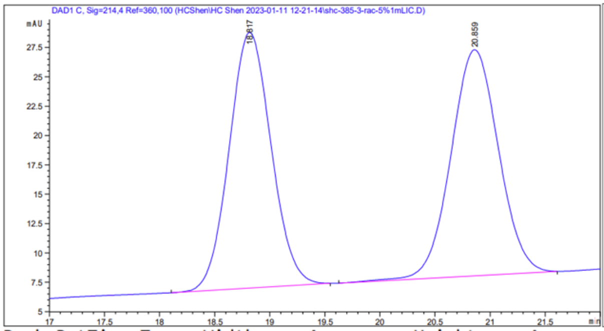

Peak #	RetTime [min]	Type	Width [min]	Area [mAU*s]	Height [mAU]	Area %
1	18.817	BB	0.4132	577.01947	21.76971	49.9717
2	20.859	BB	0.4680	577.67395	19.25664	50.0283

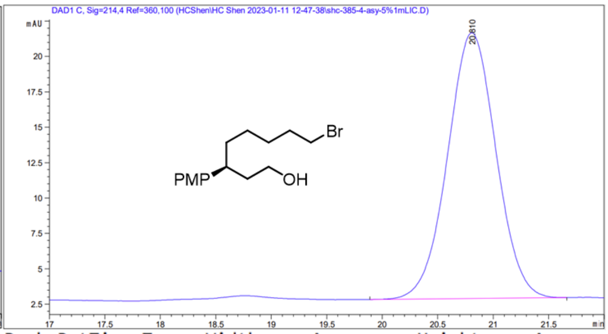

Peak #	RetTime [min]	Type	Width [min]	Area [mAU*s]	Height [mAU]	Area %
1	20.810	BB	0.4699	569.13043	18.76432	100.0000

1-((*S*)-6-(4-methoxyphenyl)oct-7-en-1-yl)-2-methylene-1,2,3,4-tetrahydronaphthalene (36)

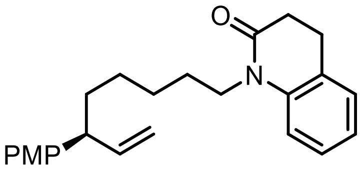

36

Prepared following **General Procedure D**. Purification by flash column chromatography (15% DCM in *n*-hexane) gave the title compound (29.7 mg, 41%, 99% ee) as a white solid.

^{1}H NMR (400 MHz, $CDCl_3$): δ 7.25 – 7.19 (m, 1H), 7.16 (d, J = 7.5 Hz, 1H), 7.12 – 7.04 (m, 2H), 7.03 – 6.92 (m, 2H), 6.87 – 6.79 (m, 2H), 5.97 – 5.84 (m, 1H), 5.03 – 4.97 (m, 1H), 4.97 – 4.95 (m, 1H), 3.93 – 3.85 (m, 2H), 3.78 (s, 3H), 3.17 (q, *J* = 7.5 Hz, 1H), 2.89 – 2.85 (m, 2H), 2.68 – 2.57 (m, 2H), 1.76 – 1.57 (m, 4H), 1.42 – 1.17 (m, 4H) ppm; **^{13}C NMR** (101 MHz, $CDCl_3$): δ 170.2, 158.1, 142.8, 139.8, 136.7, 128.6, 128.1, 127.5, 126.8, 122.8, 115.0, 114.0, 113.8, 55.4, 49.1, 42.3, 35.5, 32.1, 27.4, 27.3, 27.0, 25.8 ppm. **Specific rotation** $[\alpha]_D^{24}$ = +9 (c = 0.85, CH_2Cl_2). **HRMS** (ESI) m/z calculated for $C_{24}H_{29}NO_2$ $[M+H]^+$, 364.2271; found, 364.2260. **IR (neat)** 2934, 2836, 1650, 1602, 1510, 1463, 1246, 1175, 1033, 829, 755, 589, 540 cm^{-1}. **HPLC conditions**: Chiral column IC, hexane: isopropanol = 80:20, flow rate = 1.0 mL/min, wavelength = 214 nm, t_R = 24.1 min for major isomer, t_R = 7.6 min for minor isomer.

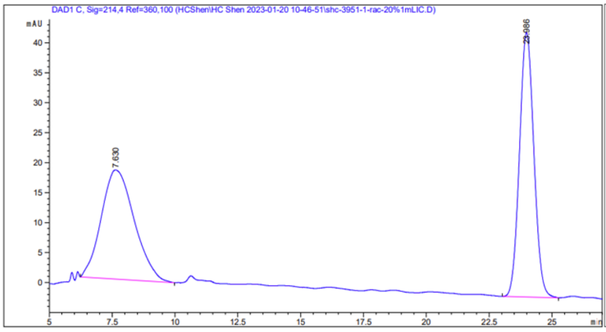

Peak #	RetTime [min]	Type	Width [min]	Area [mAU*s]	Height [mAU]	Area %
1	7.630	BB	1.1917	1660.86804	18.18267	48.6411
2	23.986	BB	0.6176	1753.66553	44.12734	51.3589

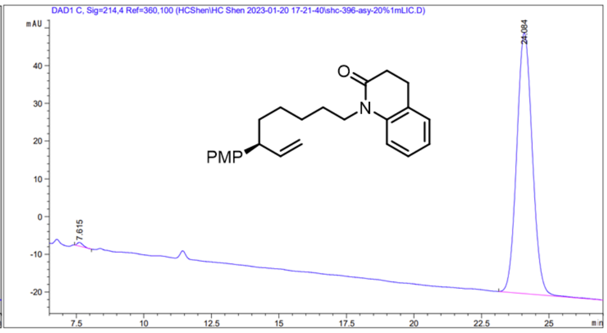

Peak #	RetTime [min]	Type	Width [min]	Area [mAU*s]	Height [mAU]	Area %
1	7.615	BB	0.2237	14.66649	1.00837	0.5245
2	24.084	BBA	0.6254	2781.52979	69.12838	99.4755

(*S*)-3-(5-(4-methoxyphenyl)hept-6-en-1-yl)pyridine (37)

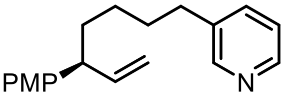

37

Prepared following **General Procedure D**. Purification by flash column chromatography (15% DCM in *n*-hexane) gave the title compound (17.7 mg, 31%, 98% ee) as a yellow oil.

^{1}H NMR (400 MHz, $CDCl_3$): δ 8.45 – 8.39 (m, 2H), 7.47 – 7.40 (m, 1H), 7.17 (ddd, *J* = 7.8, 4.8, 0.9 Hz, 1H), 7.13 – 7.04 (m, 2H), 6.88 – 6.80 (m, 2H), 5.98 – 5.84 (m, 1H), 5.01 – 5.0 (m, 1H), 5.00 – 4.94 (m, 1H), 3.79 (s, 3H), 3.18 (q, *J* = 7.5 Hz, 1H), 2.61 – 2.52 (m, 2H), 1.76 – 1.65 (m, 2H), 1.66 – 1.56 (m, 2H), 1.42 – 1.19 (m, 2H) ppm; **^{13}C NMR** (101 MHz, $CDCl_3$): δ

158.1, 150.2, 147.4, 142.7, 137.9, 136.5, 135.8, 128.5, 123.3, 114.0, 113.8, 55.4, 49.0, 35.3, 33.0, 31.2, 27.2 ppm. **Specific rotation** $[\alpha]_D^{24}$ = +24 (c = 0.59, CH_2Cl_2). **HRMS** (ESI) m/z calculated for $C_{19}H_{23}NO$ $[M+H]^+$, 282.1852; found, 282.1844. **IR (neat)** 2933, 2858, 1610, 1510, 1421, 1247, 1178, 1033, 913, 830, 714 cm^{-1}. **HPLC conditions**: Chiral column IC, hexane: isopropanol = 90:10, flow rate = 1.0 mL/min, wavelength = 210 nm, t_R = 29.0 min for major isomer, t_R = 27.2 min for minor isomer.

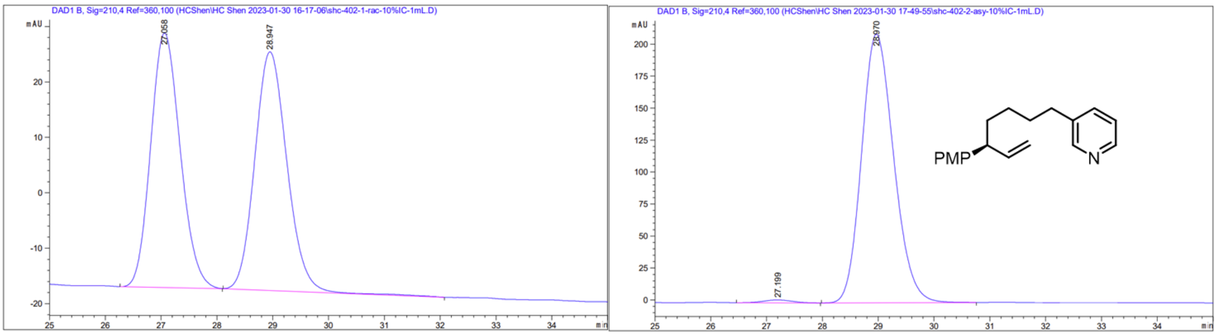

Peak #	RetTime [min]	Type	Width [min]	Area [mAU*s]	Height [mAU]	Area %
1	27.058	BB	0.5717	1683.60461	45.92091	49.3905
2	28.947	BB	0.6214	1725.16016	43.05820	50.6095

Peak #	RetTime [min]	Type	Width [min]	Area [mAU*s]	Height [mAU]	Area %
1	27.199	BB	0.5392	79.10429	2.24412	0.9335
2	28.970	BB	0.6225	8394.45410	209.95081	99.0665

(*R*)-1-(4,4-dimethylpent-1-en-3-yl)-4-methoxybenzene (38)

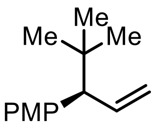

38

Prepared following **General Procedure E**. Purification by flash column chromatography (5% EtOAc in *n*-hexane) gave the title compound (15.5 mg, 38%, 88% ee, 7.4:1 b:l) as a colorless oil.

^{1}H NMR (400 MHz, $CDCl_3$): δ 7.13 – 7.05 (m, 2H), 6.86 – 6.78 (m, 2H), 6.30 – 6.18 (m, 1H), 5.11 – 4.97 (m, 2H), 3.79 (s, 3H), 2.98 (d, *J* = 9.7 Hz, 1H), 0.88 (s, 9H) ppm; **^{13}C NMR** (101 MHz, $CDCl_3$): δ 158.0, 139.2, 135.1, 130.1, 116.0, 113.3, 60.8, 55.3, 34.0, 28.1 ppm. **Specific rotation** $[\alpha]_D^{24}$ = +7 (c = 0.30, CH_2Cl_2). **HRMS** (EI) m/z calculated for $C_{14}H_{20}O$ $[M]^+$, 204.1509; found, 204.1506. **IR (neat)** 2955, 1610, 1512, 1464, 1247, 1179, 1040, 829, 750 cm^{-1}.

The enantiomeric excess (ee) was determined after hydroboration/oxidation to alcohol **38'**.

(*R*)-3-(4-methoxyphenyl)-4,4-dimethylpentan-1-ol (38')

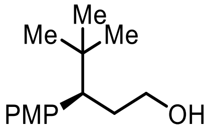

38'

Prepared following **General Procedure F** (0.1 mmol **38**). Purification by flash column chromatography (30% EtOAc in *n*-hexane) gave the title compound (19.3 mg, 87%, 88% ee) as a colorless oil.

^{1}H NMR (400 MHz, $CDCl_3$): δ 7.11 – 7.02 (m, 2H), 6.86 – 6.77 (m, 2H), 3.79 (s, 3H), 3.49 – 3.43 (m, 1H), 3.38 – 3.32 (m, 1H), 2.42 (dd, J = 12.2, 3.1 Hz, 1H), 2.09 – 2.01 (m, 1H), 1.94 – 1.84 (m, 1H), 0.87 (s, 9H) ppm; **^{13}C NMR** (101 MHz, $CDCl_3$): δ 158.1, 134.4, 130.4, 113.3, 62.2, 55.3, 52.2, 33.9, 32.7, 28.3 ppm. **Specific rotation** $[\alpha]_D^{24}$ = +12 (c = 0.62, CH_2Cl_2). **HRMS** (EI) m/z calculated for $C_{14}H_{22}O_2$ $[M]^+$, 222.1614; found, 222.1611. **IR (neat)** 3343, 2950, 2868, 1610, 1512, 1364, 1247, 1033, 831, 769, 564 cm^{-1}. **HPLC conditions**: Chiral column IC, hexane: isopropanol = 95:5, flow rate = 1.0 mL/min, wavelength = 214 nm, t_R = 13.7 min for major isomer, t_R = 12.9 min for minor isomer.

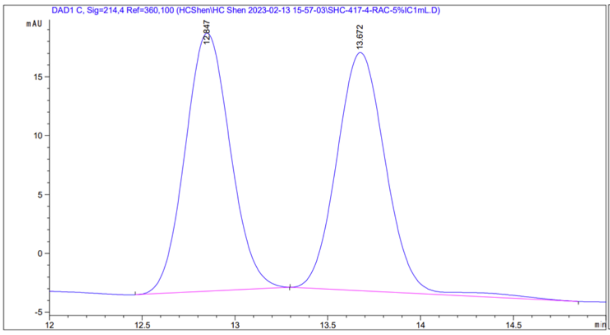

Peak #	RetTime [min]	Type	Width [min]	Area [mAU*s]	Height [mAU]	Area %
1	12.847	BB	0.2649	370.25745	21.94126	49.8364
2	13.672	BB	0.2868	372.68860	20.24461	50.1636

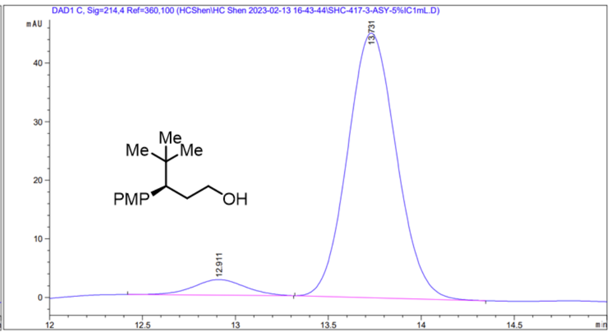

Peak #	RetTime [min]	Type	Width [min]	Area [mAU*s]	Height [mAU]	Area %
1	12.911	BB	0.2983	51.46751	2.65300	5.8879
2	13.731	BB	0.2844	822.65222	45.18330	94.1121

(*R*)-(1-(4-methoxyphenyl)allyl)cyclododecane (39)

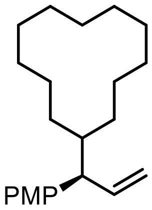

39

Prepared following **General Procedure E**. Purification by flash column chromatography (5% EtOAc in *n*-hexane) gave the title compound (39.8 mg, 41%, 96% ee; and 34% **38**) as a colorless oil.

^{1}H NMR (400 MHz, $CDCl_3$): δ 7.12 – 7.06 (m, 2H), 6.87 – 6.80 (m, 2H), 5.93 (ddd, *J* = 16.9, 10.1, 9.2 Hz, 1H), 5.04 – 4.94 (m, 2H), 3.79 (s, 3H), 3.04 (t, *J* = 9.3 Hz, 1H), 1.82 – 1.76 (m, 1H), 1.51 – 1.09 (m, 22H) ppm; **^{13}C NMR** (101 MHz, $CDCl_3$): δ 157.9, 142.2, 136.7, 128.8, 114.4, 113.9, 55.3, 53.6, 39.9, 26.3, 26.2, 25.7, 25.6, 25.1, 23.2, 23.1(4), 23.1(3), 23.0, 21.4, 21.2 ppm. **HRMS** (EI) m/z calculated for $C_{22}H_{34}O$ $[M]^+$, 314.2604; found, 314.2601. **IR (neat)** 2930, 2862, 1610, 1511, 1470, 1245, 1145, 1034, 912, 828, 750, 650, 539 cm^{-1}.

The enantiomeric excess (ee) was determined after hydroboration/oxidation to alcohol **39'**.

(*R*)-3-cyclododecyl-3-(4-methoxyphenyl)propan-1-ol (39')

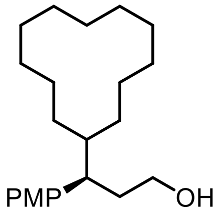

39'

Prepared following **General Procedure F** (0.08 mmol **39**). Purification by flash column chromatography (30% EtOAc in *n*-hexane) gave the title compound (22.3 mg, 82%, 96% ee) as a colorless oil.

^{1}H NMR (400 MHz, $CDCl_3$): δ 7.09 – 7.01 (m, 2H), 6.86 – 6.78 (m, 2H), 3.79 (s, 1H), 3.51 – 3.42 (m, 1H), 3.38 (t, *J* = 5.9 Hz, 1H), 2.54 (ddd, *J* = 11.7, 8.2, 3.8 Hz, 1H), 2.10 – 2.02 (m, 1H), 1.79 – 1.70 (m, 1H), 1.65 – 1.60 (m, 1H), 1.42 – 1.00 (m, 22H) ppm; **^{13}C NMR** (101 MHz, $CDCl_3$): δ 158.0, 136.3, 129.4, 113.7, 62.0, 55.3, 44.3, 40.4, 36.5, 27.0, 25.8, 25.4, 25.3, 24.8, 23.6, 23.5, 23.0(1), 23.9(8), 21.9, 21.3 ppm. **Specific rotation** $[\alpha]_D^{24}$ = −15 (c = 0.39, CH_2Cl_2). **HRMS** (ESI) m/z calculated for $C_{22}H_{36}O_2$ $[M-H_2O+H]^+$, 315.2682; found, 315.2678. **IR (neat)** 3364, 2935, 2863, 1610, 1511, 1670, 1246, 1033, 828 cm^{-1}. **HPLC conditions**: Chiral column IC, hexane: isopropanol = 95:5, flow rate = 1.0 mL/min, wavelength = 230 nm, t_R = 15.3 min for major isomer, t_R = 13.5 min for minor isomer.

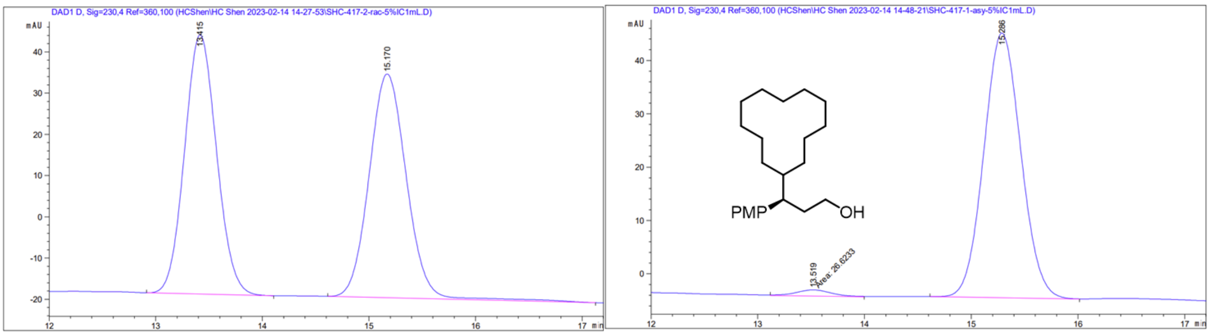

Peak #	RetTime [min]	Type	Width [min]	Area [mAU*s]	Height [mAU]	Area %
1	13.415	BB	0.3253	1311.94312	62.90770	49.7452
2	15.170	BB	0.3787	1325.38525	54.31709	50.2548

Peak #	RetTime [min]	Type	Width [min]	Area [mAU*s]	Height [mAU]	Area %
1	13.519	MM	0.3837	26.62325	1.15638	2.1756
2	15.286	BB	0.3754	1197.10278	49.62535	97.8244

***tert*-butyl (*R*)-2-((*S*)-1-(4-methoxyphenyl)allyl)pyrrolidine-1-carboxylate (41)**

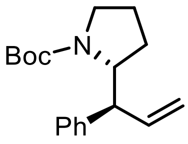

41

Prepared following **General Procedure E** using (*R*)-**40** and (*S*)-**L1** with 2.5 mol% $[Ir(COD)Cl]_2$ and 10 mol% (*S*)-**L1**. Purification by flash column chromatography (15% EtOAc in *n*-hexane) gave the title compound (38.9 mg, 69%, 95% ee; 84:16 dr; 95:5 b:l) as a colorless oil.

^{1}H NMR (400 MHz, $CDCl_3$): δ 7.29 (d, J = 6.1 Hz, 3H), 7.25 – 7.16 (m, 2H), 6.18 (dt, *J* = 16.5, 9.7 Hz, 1H), 5.19 (dd, *J* = 10.2, 2.0 Hz, 1H), 5.13 (d, *J* = 17.1 Hz, 1H), 4.21 – 3.81 (m, 2H),

3.63 – 3.39 (m, 1H) , 3.33 – 3.19 (m, 1H), 1.98 – 1.67 (m, 4H), 1.44 (s, 9H) ppm; **^{13}C NMR** (101 MHz, $CDCl_3$): δ 154.6, 142.0, 139.4, 136.4, (128.5) 128.4, 126.5 (126.4), 118.3, 79.5 (79.1), 62.3, 52.5 (51.2), (47.5) 46.9, 28.6, 27.9 (27.1), (24.2) 23.4 ppm. **Specific rotation** $[\alpha]_D^{24}$ = +38 (c = 0.53, CH_2Cl_2). **HRMS** (ESI) m/z calculated for $C_{18}H_{25}NO_2$ $[M+Na]^+$, 310.1778; found, 310.1771. **IR (neat)** 2974, 1690, 1601, 1392, 1254, 1169, 918, 701, 547 cm^{-1}. Note: Due to the presence of rotamers, N-Boc pyrrole often show a broad C–H peak in the ^{1}H NMR and carbons near the Boc group can appear as broad peaks with low intensity or broad peaks with shoulders in the ^{13}C NMR.

The enantiomeric excess (ee) was determined after hydroboration/oxidation to alcohol.

***tert*-butyl (*R*)-2-((*R*)-1-(4-methoxyphenyl)allyl)pyrrolidine-1-carboxylate (42)**

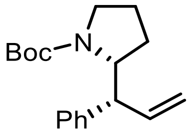

42

Prepared following **General Procedure E** using (*R*)-**40** and (*R*)-**L1** with 2.5 mol% $[Ir(COD)Cl]_2$ and 10% (*R*)-**L1**. Purification by flash column chromatography (15% EtOAc in *n*-hexane) gave the title compound (38.5 mg, 68%, 94% ee; 91:9 dr; 98:2 b:l) as a colorless oil.

^{1}H NMR (400 MHz, $CDCl_3$): δ 7.31 – 7.28 (m, 2H), 7.25 – 7.12 (m, 3H), 6.22 – 6.10 (m, 1H), 5.15 – 4.98 (m, 2H), 4.31 – 4.10 (m, 1H), 3.63 (dt, *J* = 75.1, 7.8 Hz, 1H), 3.33 (dq, *J* = 56.9, 8.9 Hz, 1H), 3.15 – 2.95 (m, 1H), 1.82 – 1.74 (m, 1H), 1.69 – 1.62 (m, 2H), 1.54 – 1.49 (m, 9H), 1.38 – 1.31 (m, 1H) ppm; **^{13}C NMR** (101 MHz, $CDCl_3$): δ 155.1 (154.9), 141.7 (141.4), 139.4, 128.8 (128.6), 128.6 (128.5), 126.8 (126.6), 116.0 (115.7), 79.6 (79.1), 61.5 (61.3), 53.6 (52.9), 46.6 (46.1), 28.7, 28.2 (27.3), 23.4 (22.5) ppm. **Specific rotation** $[\alpha]_D^{24}$ = +23 (c = 0.26, CH_2Cl_2). **HRMS** (ESI) m/z calculated for $C_{18}H_{25}NO_2$ $[M+Na]^+$, 310.1778; found, 310.1774. **IR (neat)** 2974, 1691, 1453, 1392, 1171, 1033, 915, 757, 703 cm^{-1}. Note: Due to the presence of rotamers, N-Boc pyrrole often show a broad C–H peak in the ^{1}H NMR and carbons near the

Boc group can appear as broad peaks with low intensity or broad peaks with shoulders in the ^{13}C NMR.

The enantiomeric excess (ee) was determined after hydroboration/oxidation to alcohol.

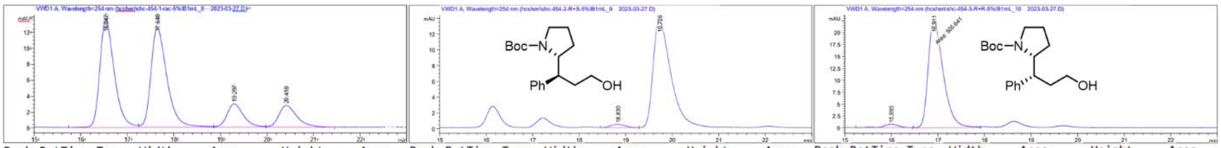

Peak #	RetTime [min]	Type	Width [min]	Area [mAU*s]	Height [mAU]	Area %
1	16.542	BV	0.3351	292.39563	13.45284	39.5059
2	17.648	VV	0.3680	299.62289	12.45791	40.4824
3	19.297	VV	0.3799	73.30965	2.95301	9.9050
4	20.418	VB	0.4080	74.80282	2.72173	10.1067

Peak #	RetTime [min]	Type	Width [min]	Area [mAU*s]	Height [mAU]	Area %
1	18.830	BV	0.3159	9.47407	4.29060e-1	2.4748
2	19.726	VV R	0.4073	373.35355	13.61253	97.5252

Peak #	RetTime [min]	Type	Width [min]	Area [mAU*s]	Height [mAU]	Area %
1	15.995	BB	0.3334	16.45293	7.28412e-1	3.1513
2	16.911	MM	0.3734	505.64081	22.57187	96.8487

((3*S*,4*R*)-hex-1-ene-3,4-diyl)dibenzene (S3)

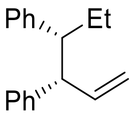

S3

Prepared following **General Procedure A** using (*R*)-4,4,5,5-tetramethyl-2-(1-phenylpropyl)-1,3,2-dioxaborolane and (*R*)-**L1** and THF was used as the solvent. Purification by flash column chromatography (0–1% DCM in *n*-hexane) gave the title compound (25.1 mg, 53%, 96% ee, 89:11 dr, 79:21 b:l) as a white solid.

^{1}H NMR (400 MHz, $CDCl_3$) δ 7.35 – 7.26 (m, 4H), 7.25 – 7.16 (m, 4H), 7.15 – 7.11 (m, 2H), 5.80 (ddd, *J* = 17.0, 10.2, 8.2 Hz, 1H), 4.80 (ddd, *J* = 10.3, 1.8, 0.9 Hz, 1H), 4.72 (ddd, *J* = 17.1, 1.8, 1.1 Hz, 1H), 3.52 – 3.43 (m, 1H), 2.78 (td, *J* = 10.2, 4.1 Hz, 1H), 1.56 – 1.38 (m, 2H), 0.62 (t, *J* = 7.3 Hz, 3H) ppm. **^{13}C NMR** (101 MHz, $CDCl_3$) δ 143.8, 143.3, 140.9, 128.9, 128.6, 128.4, 128.2, 126.4, 126.3, 115.3, 56.6, 53.4, 27.1, 12.2 ppm. **Specific rotation** $[\alpha]_D^{21}$ = −38 (c = 2.0, CH_2Cl_2). **HRMS** (EI) m/z calculated for $C_{18}H_{20}$ $[M]^+$, 236.1560, found: 236.1556. **IR (neat)** 3027, 2961, 1601, 1494, 1452, 965, 914, 756, 699 cm^{-1}.

The enantiomeric excess (ee) was determined after hydroboration/oxidation to alcohol **S3'**.

(3*S*,4*R*)-3,4-diphenylhexan-1-ol (S3')

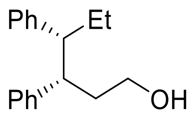

S3'

Prepared following **General Procedure F (**0.1 mmol **S3**, 89:11 dr, 79:21 b:l). Purification by flash column chromatography (0–30% EtOAc in *n*-hexane) gave the title compound (9.3 mg, 52%, >99% ee) based on major isomer as a white solid.

^{1}H NMR (600 MHz, $CDCl_3$) δ 7.36 – 7.31 (m, 4H), 7.26 – 7.18 (m, 6H), 3.33 – 3.27 (m, 1H), 3.24 – 3.17 (m, 1H), 2.87 (td, *J* = 10.6, 4.4 Hz, 1H), 2.60 (td, *J* = 10.7, 3.8 Hz, 1H), 1.68 – 1.56 (m, 2H), 1.44 – 1.28 (m, 2H), 1.25 – 1.18 (m, 1H), 0.54 (t, *J* = 7.3 Hz, 3H) ppm. **^{13}C NMR** (151 MHz, $CDCl_3$) δ 144.1, 128.7, 128.6, 128.5, 128.4, 126.5, 126.4, 61.4, 54.4, 49.0, 37.7, 27.5, 12.3 ppm. **Specific rotation** $[\alpha]_D^{23}$ = −2 (c = 1.0, CH_2Cl_2). **GC-MS** (EI) m/z calculated for $C_{18}H_{22}O$ $[M]^+$, 254.1, found: 253.9. Note: HRMS (EI, ESI and APCI) was tried, but didn't find desired mass. **IR (neat)** 3290, 2955, 2871, 1600, 1494, 1452, 1092, 1035, 875, 750, 700. **HPLC conditions**: Chiral column IB, hexane: isopropanol = 95:5, flow rate = 1.0 mL/min, wavelength = 210 nm, t_R = 10.2 min for major isomer, t_R = 11.7 min for minor isomer.

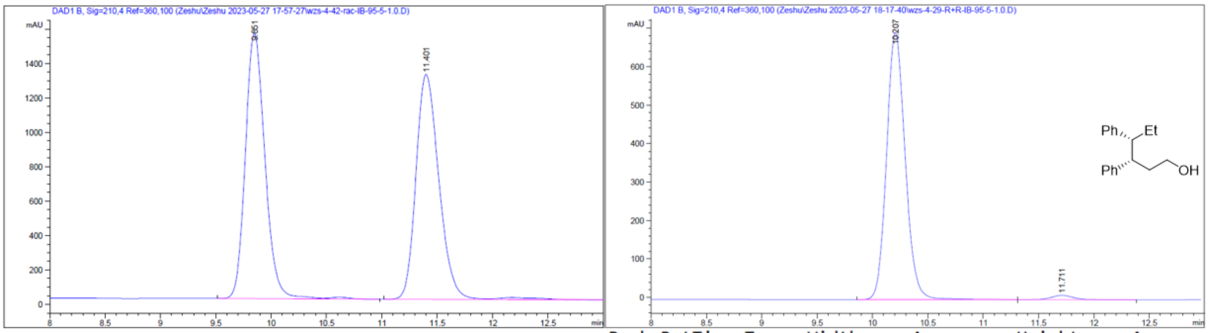

Peak #	RetTime [min]	Type	Width [min]	Area [mAU*s]	Height [mAU]	Area %
1	9.851	BV R	0.1915	1.90730e4	1541.18225	50.0307
2	11.401	BV R	0.2208	1.90495e4	1308.03796	49.9693

Peak #	RetTime [min]	Type	Width [min]	Area [mAU*s]	Height [mAU]	Area %
1	10.207	BB	0.1825	8121.70654	694.10107	98.1470
2	11.711	BB	0.2143	153.33492	11.02425	1.8530

4-((2*S*,3*R*)-3-phenylpentan-2-yl)-1,1'-biphenyl (47)

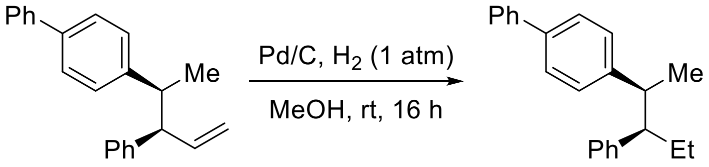

4, >99:1 dr **47**, 83%

Prepared following **Procedure G** (0.1 mmol **4**, >99:1 dr). Purification by flash column chromatography (0–1% EtOAc in *n*-hexane) gave the title compound **47** (24.8 mg, 83%, >99:1 dr) as a white solid.

^{1}H NMR (500 MHz, $CDCl_3$) δ 7.65 – 7.59 (m, 2H), 7.58 – 7.52 (m, 2H), 7.47 – 7.41 (m, 2H), 7.37 – 7.27 (m, 5H), 7.26 – 7.17 (m, 3H), 2.96 – 2.86 (m, 1H), 2.57 (td, J = 10.6, 3.7 Hz, 1H), 1.58 – 1.35 (m, 2H), 1.02 (d, J = 6.9 Hz, 3H), 0.59 (t, J = 7.4 Hz, 3H) ppm. **^{13}C NMR** (126 MHz, $CDCl_3$) δ 146.2, 144.2, 141.2, 139.0, 128.9, 128.6, 128.3, 128.2, 127.1(7), 127.1(5), 127.1, 126.2, 55.3, 46.1, 27.5, 21.3, 12.5 ppm. **Specific rotation** $[\alpha]_D^{23}$ = −15 (c = 2.0, CH_2Cl_2). **HRMS** (EI) m/z calculated for $C_{23}H_{24}$ $[M]^+$, 300.1873, found: 300.1868. **IR (neat)** 3028, 2923, 2869, 1599, 1486, 1451, 1007, 829, 761, 726, 695 cm^{-1}.

(2*R*,3*S*)-3-([1,1'-biphenyl]-4-yl)-2-phenylbutan-1-ol (48)

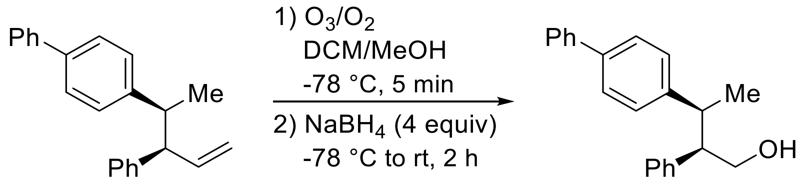

4, >99:1 dr **48**, 75%, >99:1 dr

Prepared following **Procedure H** (0.1 mmol **4**, >99:1 dr). Purification by flash column chromatography (0–30% EtOAc in *n*-hexane) to give alcohol **48** as a white solid (22.8 mg, 75%, >99:1 dr).

^{1}H NMR (500 MHz, $CDCl_3$) δ 7.64 – 7.60 (m, 2H), 7.60 – 7.56 (m, 2H), 7.48 – 7.43 (m, 2H), 7.43 – 7.38 (m, 2H), 7.38 – 7.28 (m, 6H), 3.68 – 3.54 (m, 2H), 3.11 – 3.01 (m, 1H), 3.01 – 2.93 (m, 1H), 1.13 – 1.04 (m, 3H) ppm. **^{13}C NMR** (126 MHz, $CDCl_3$) δ 144.8, 141.6, 141.1, 139.6, 128.9(2), 128.8(9), 128.8, 127.9, 127.5, 127.3, 127.2, 66.1, 55.8, 42.3, 21.1 ppm. **Specific**

rotation $[\alpha]_D^{23}$ = −7 (c = 2.0, CH_2Cl_2). **HRMS** (APCI) m/z calculated for $C_{22}H_{21}$ $[M-H_2O+H]^+$, 285.1638, found: 285.1631. **IR (neat)** 3586, 3029, 2967, 1599, 1486, 1276, 1261, 1049, 1012, 836, 763, 696 cm^{-1}.

methyl (4*R*,5*S*,*E*)-5-([1,1'-biphenyl]-4-yl)-4-phenylhex-2-enoate (49)

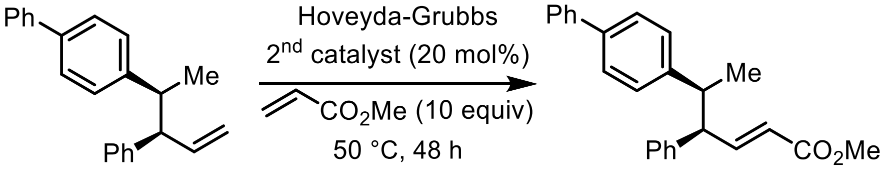

4, >99:1 dr **49**, 88%, >99:1 dr

Prepared following **Procedure I** (0.1 mmol **4**, >99:1 dr). Purification by flash column chromatography (0–5% EtOAc in *n*-hexane) to give the title compound **49** (31.5 mg, 88%, >99:1 dr).

^{1}H NMR (400 MHz, $CDCl_3$) δ 7.66 – 7.60 (m, 2H), 7.59 – 7.53 (m, 2H), 7.49 – 7.43 (m, 2H), 7.41 – 7.33 (m, 3H), 7.32 – 7.22 (m, 5H), 7.04 (dd, *J* = 15.6, 8.4 Hz, 1H), 5.57 (dd, *J* = 15.6, 1.2 Hz, 1H), 3.63 (s, 3H), 3.62 – 3.56 (m, 1H), 3.27 – 3.15 (m, 1H), 1.17 (d, *J* = 7.0 Hz, 3H) ppm **^{13}C NMR** (101 MHz, $CDCl_3$) δ 166.9, 150.6, 143.8, 141.4, 141.0, 139.5, 128.9(1), 128.8(7), 128.5, 128.2, 127.2(9), 127.2(5), 127.1, 121.7, 56.2, 51.5, 44.9, 20.8 ppm. **Specific rotation** $[\alpha]_D^{23}$ = +39 (c = 2.0, CH_2Cl_2). **HRMS** (APCI) m/z calculated for $C_{25}H_{25}O_2$ $[M+H]^+$, 357.1849, found: 357.1848. **IR (neat)** 3027, 2953, 1718, 1652, 1486, 1434, 1270, 1238, 1165, 842, 765, 733, 697 cm^{-1}.

2,2'-((2*R*,3*R*,4*S*)-4-([1,1'-biphenyl]-4-yl)-3-phenylpentane-1,2-diyl)bis(4,4,5,5-tetramethyl-1,3,2-dioxaborolane) (50)

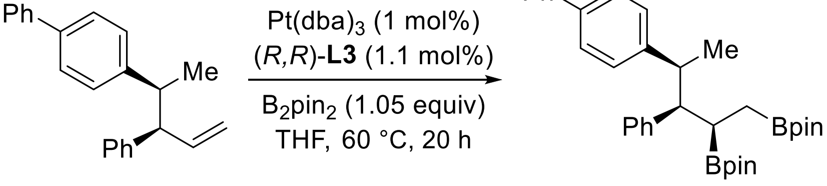

4, >99:1 dr **50**, 89%, >95:5 dr

Prepared following **Procedure J** (0.1 mmol **4**, >99:1 dr). Purification by flash column

chromatography (0–10% EtOAc in *n*-hexane) to give the title compound **50** (48.9 mg, 89%, >95:5 dr).

^{1}H NMR (400 MHz, $CDCl_3$) δ 7.63 – 7.55 (m, 2H), 7.50 – 7.45 (m, 2H), 7.45 – 7.39 (m, 2H), 7.35 – 7.29 (m, 1H), 7.22 – 7.10 (m, 5H), 7.10 – 7.04 (m, 2H), 3.34 – 3.23 (m, 1H), 3.13 – 3.04 (m, 1H), 1.67 – 1.52 (m, 1H), 1.23 – 1.12 (m, 24H), 1.10 (d, *J* = 7.0 Hz, 3H), 0.92 – 0.69 (m, 2H) ppm. **^{13}C NMR** (101 MHz, $CDCl_3$) δ 145.9, 141.7, 141.5, 138.6, 129.9, 128.8, 128.3, 127.6, 127.1, 127.0, 126.8, 126.0, 83.1, 82.8, 55.1, 42.5, 25.2, 25.1(0), 25.0(6), 24.7, 18.0 ppm. **Specific rotation** $[\alpha]_D^{23}$ = −17 (c = 3.0, CH_2Cl_2). **HRMS** (ESI) m/z calculated for $C_{35}H_{47}B_2O_4$ $[M+H]^+$, 553.3655, found: 553.3680. **IR (neat)** 2976, 2928, 1601, 1486, 1369, 1311, 1142, 968, 888, 838, 764, 734, 699 cm^{-1}.

2,2'-((2*S*,3*R*,4*S*)-4-([1,1'-biphenyl]-4-yl)-3-phenylpentane-1,2-diyl)bis(4,4,5,5-tetramethyl-1,3,2-dioxaborolane) (51)

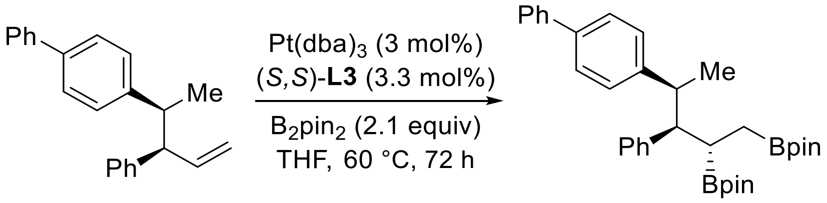

4, >99:1 dr　　**51**, 78%, >95:5 dr

Prepared following **Procedure K** (0.1 mmol **4**, >99:1 dr). Purification by flash column chromatography (0–10% EtOAc in *n*-hexane) to give the title compound **51** (42.9 mg, 78%, >95:5 dr).

^{1}H NMR (600 MHz, $CDCl_3$) δ 7.60 (d, *J* = 8.3 Hz, 2H), 7.46 (d, *J* = 8.2 Hz, 2H), 7.44 – 7.40 (m, 2H), 7.34 – 7.29 (m, 1H), 7.20 – 7.05 (m, 7H), 3.61 – 3.54 (m, 1H), 2.87 – 2.81 (m, 1H), 1.65 – 1.58 (m, 1H), 1.24 (s, 12H), 1.06 (d, *J* = 7.0 Hz, 3H), 1.03 (d, *J* = 15.7 Hz, 12H), 0.91 – 0.84 (m, 2H) ppm. **^{13}C NMR** (151 MHz, $CDCl_3$) δ 146.3, 142.8, 141.5, 138.5, 130.1, 128.8, 128.6, 127.5, 127.1, 127.0, 126.6, 126.0, 83.0, 82.9, 56.6, 40.7, 25.2, 24.9(9), 24.9(5), 24.8, 17.4 ppm. **Specific rotation** $[\alpha]_D^{23}$ = –5 (c = 4.0, CH_2Cl_2). **HRMS** (ESI) m/z calculated for

$C_{35}H_{47}B_2O_4$ [M+H]$^+$, 553.3655, found: 553.3674. **IR (neat)** 2976, 2928, 1601, 1486, 1370, 1311, 1139, 967, 846, 765, 734, 699 cm^{-1}.

Methyl 4-([1,1'-biphenyl]-4-yl)-2-methylenepentanoate (53)

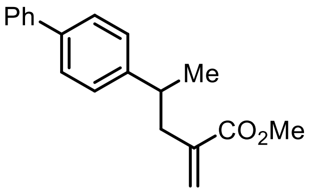

53

Purification by flash column chromatography (0–10% EtOAc in n-hexane) to give the title compound **53** (41.5 mg, 74%).

^{1}**H NMR** (600 MHz, $CDCl_3$) δ 7.63 – 7.58 (m, 2H), 7.56 – 7.52 (m, 2H), 7.45-7.42 (m, 2H), 7.36 – 7.31 (m, 1H), 7.27 (d, J = 8.2 Hz, 2H), 6.13 (s, 1H), 5.42 (s, 1H), 3.75 (s, 3H), 3.07-3.02 (m, 1H), 2.67 (dd, J = 13.9, 7.3, 1H), 2.57 (dd, J = 13.8, 7.5, , 0H), 1.31 (d, J = 7.0 Hz, 3H). ppm. ^{13}**C NMR** (151 MHz, CDC13) δ 167.9, 145.8, 141.2, 139.1, 138.9, 128.8, 127.6, 127.2, 127.2, 127.1, 126.8, 51.9, 41.0, 38.5, 21.5 ppm. **HRMS** (ESI) m/z calculated for $C_{19}H_{20}O_2$ [M+H]$^+$, 281.1536, found: 281.1538. **IR** (neat) 2956, 1904, 1717, 1438, 1149, 765, 697 cm-1.

3. REFERENCE

[1] A. F. Burchat, J. M. Chong, N. Nielsen, *J. Organomet. Chem.* **1997**, *542*, 281−283.

[2] J. Štambaský, A. V. Malkov, R. Kočovský, *J. Org. Chem.* **2008**, *73*, 9148−9150.

[3] C. R. Davis, I. K. Luvaga, J. M. Ready, *J. Am. Chem. Soc.* **2021**, *143*, 4921−4927.

[4] S. H. Bennett, A. Fawcett, E. Denton, T. Biberger, V. Fasano, N. Winter, V. K. Aggarwal, *J. Am. Chem. Soc.*, **2020**, *142*, 16766−16775.

[5] Y. Xi, J. F. Hartwig, *J. Am. Chem. Soc.* **2016**, *138*, 6703–6706.

[6] R. Larouche-Gauthier, T. G. Elford, V. K. Aggarwal, *J. Am. Chem. Soc.* **2011**, *133*, 16794–16797.

[7] Bruker, SAINT+ v8.38A Integration Engine, Data Reduction Software, Bruker Analytical X-ray Instruments Inc., Madison, WI, USA **2015**.

[8] Bruker, SADABS 2014/5, Bruker AXS area detector scaling and absorption correction, Bruker Analytical X-ray Instruments Inc., Madison, Wisconsin, USA **2014/5**.

[9] G. M. Sheldrick, SHELXT - Integrated space-group and crystal-structure determination. **2015**, *71*, 3–8.

[10] L. Palatinus, G. Chapuis, SUPERFLIP - a computer program for the solution of crystal structures by charge flipping in arbitrary dimensions. *J. Appl. Crystallogr.* **2007**, 40, 786–790.

[11] L. Palatinus, S. J. Prathapa, S. van Smaalen, EDMA: a computer program for topological analysis of discrete electron densities. *J. Appl. Crystallogr.* **2012**, 45, 575–580.

[12] G. M. Sheldrick, A short history of SHELX. *Acta Crystallogr.* **2008**, *64*, 112–122.

[13] G. M. Sheldrick, Crystal structure refinement with SHELXL. *Acta Crystallogr. C Struct. Chem.* **2015**, *71*, 3–8.

[14] O. V. Dolomanov, L. J. Bourhis, R. J. Gildea, J. A. K. Howard, H. Puschmann, OLEX2: a complete structure solution, refinement and analysis program. *J. Appl. Crystallogr.* **2009**, *42*, 339–341.

[15] S. N. Mlynarski, C. H. Schuster, J. P. Morken, *Nature* **2014**, *505*, 386–390.

[16] S. P. A. Hinkes, C. D. P. Klein, *Org. Lett.* **2019**, *21*, 3048−3052.

4. NMR SPECTRA

Compound **S1**

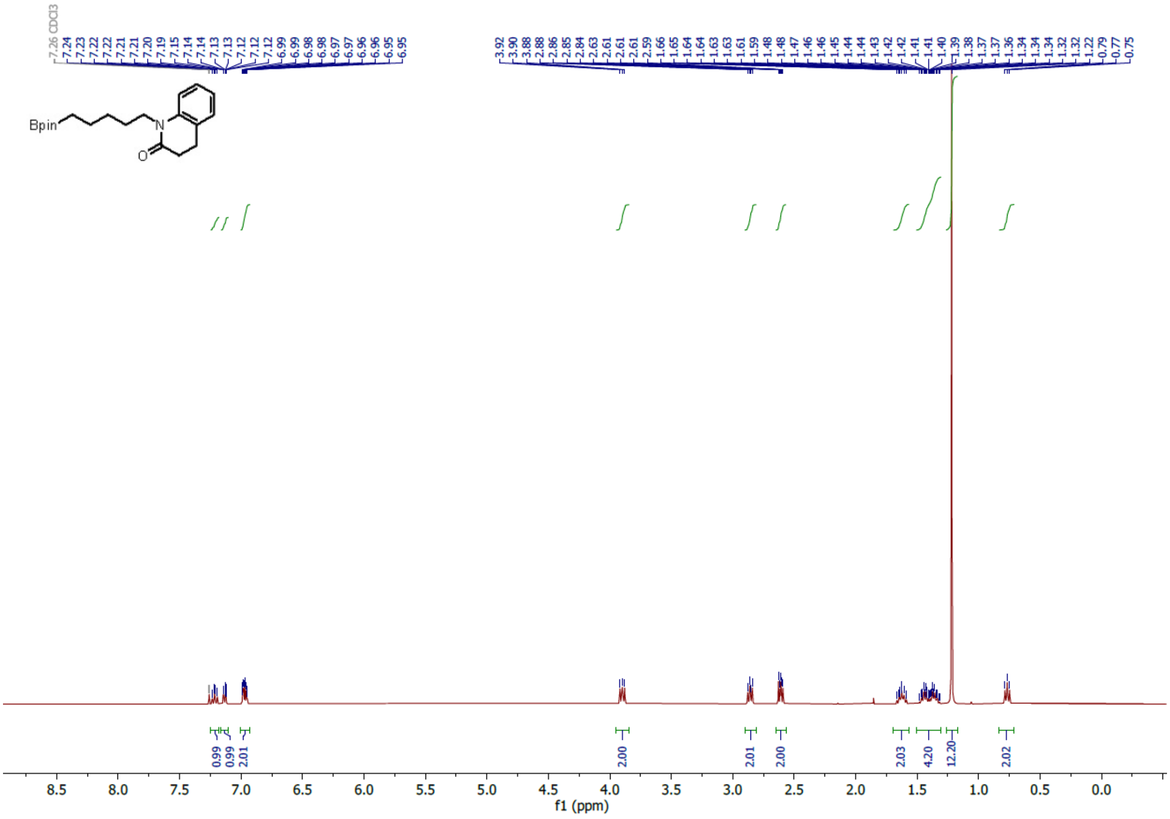

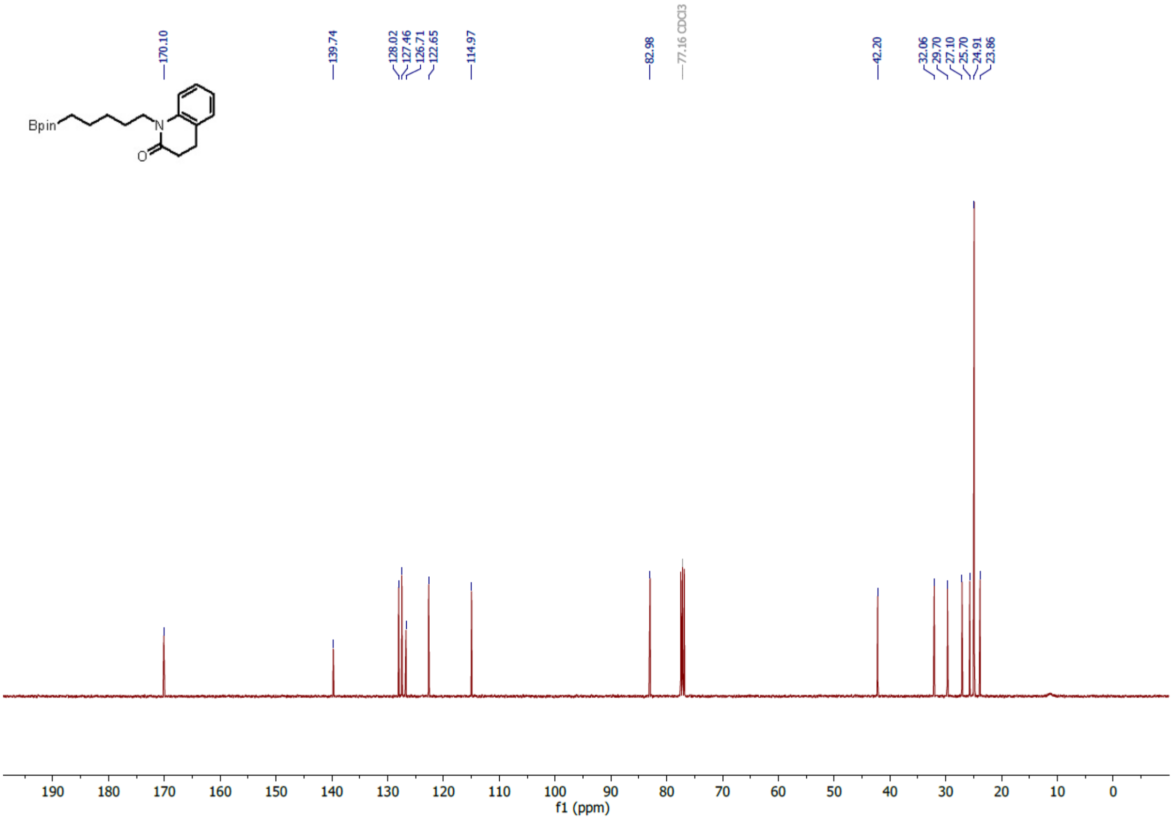

Compound **S2**

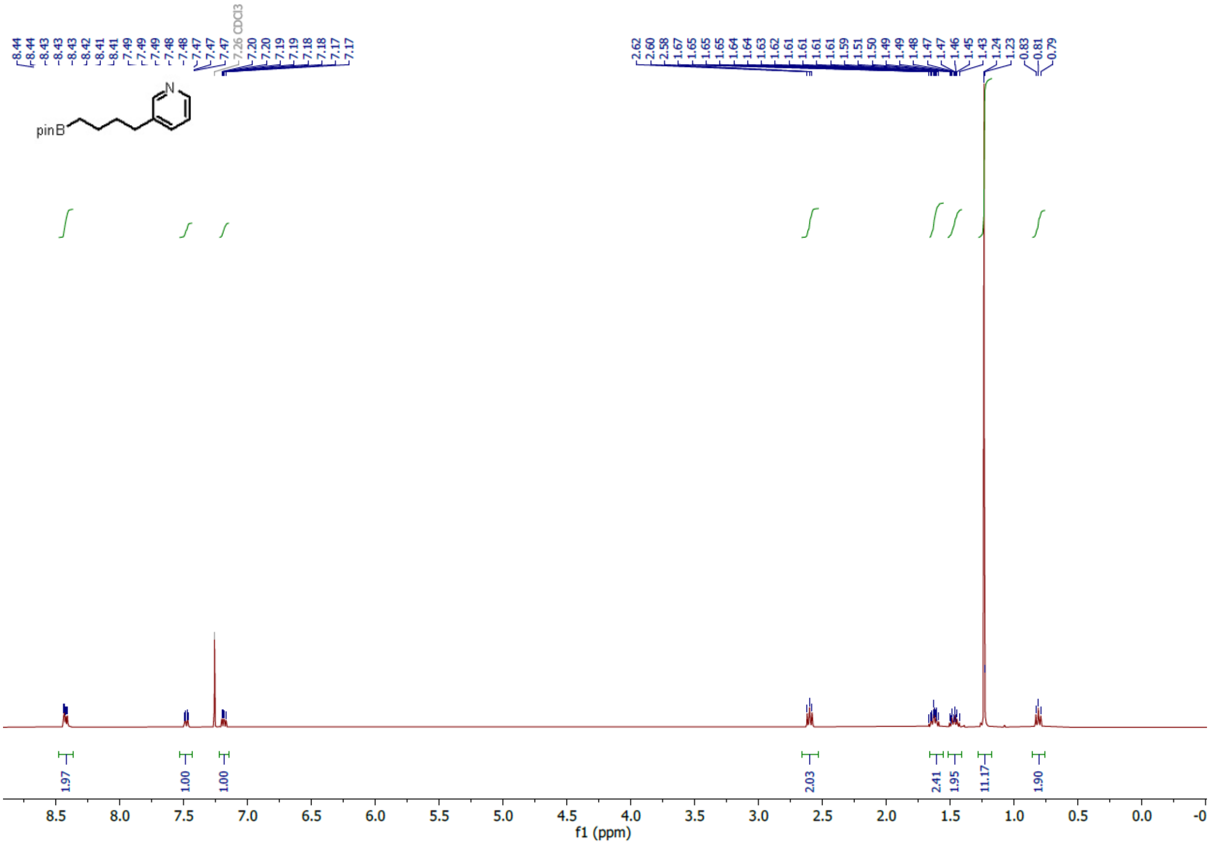

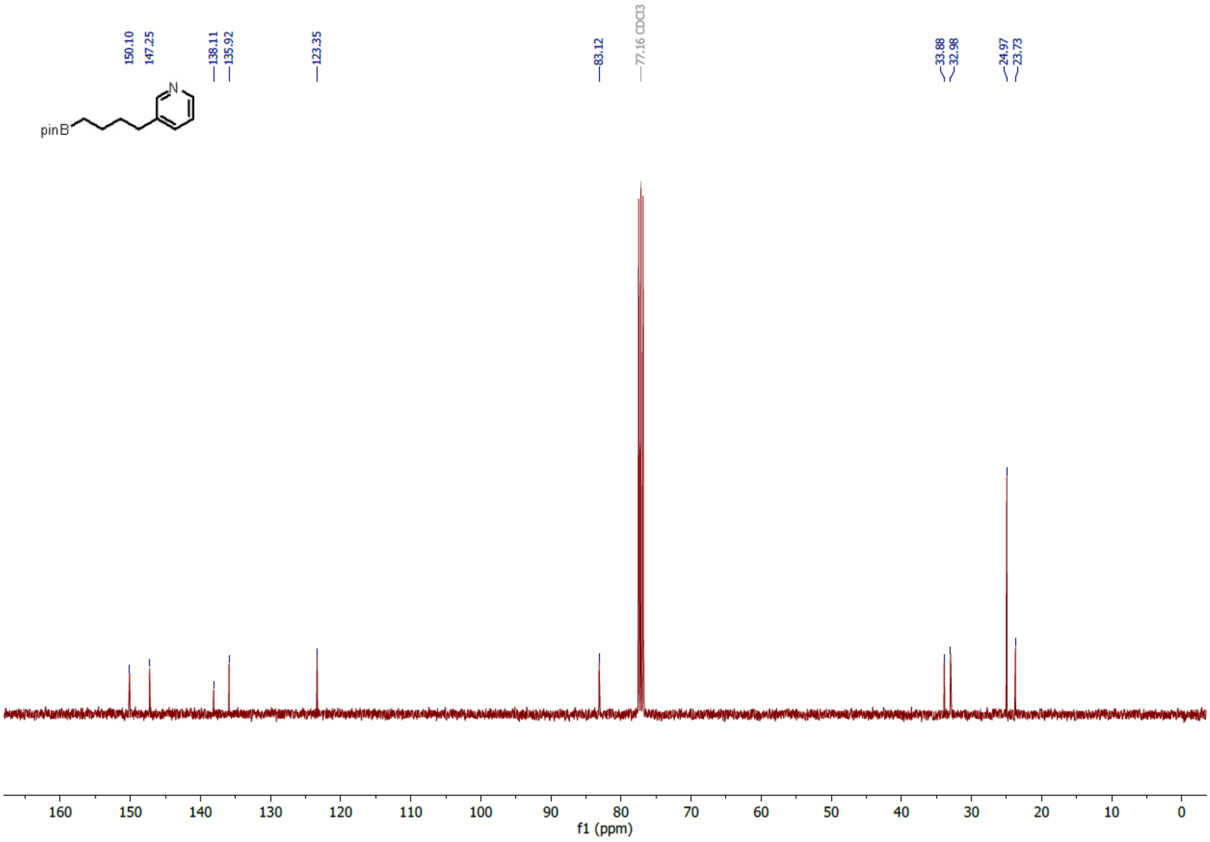

Compound **1**

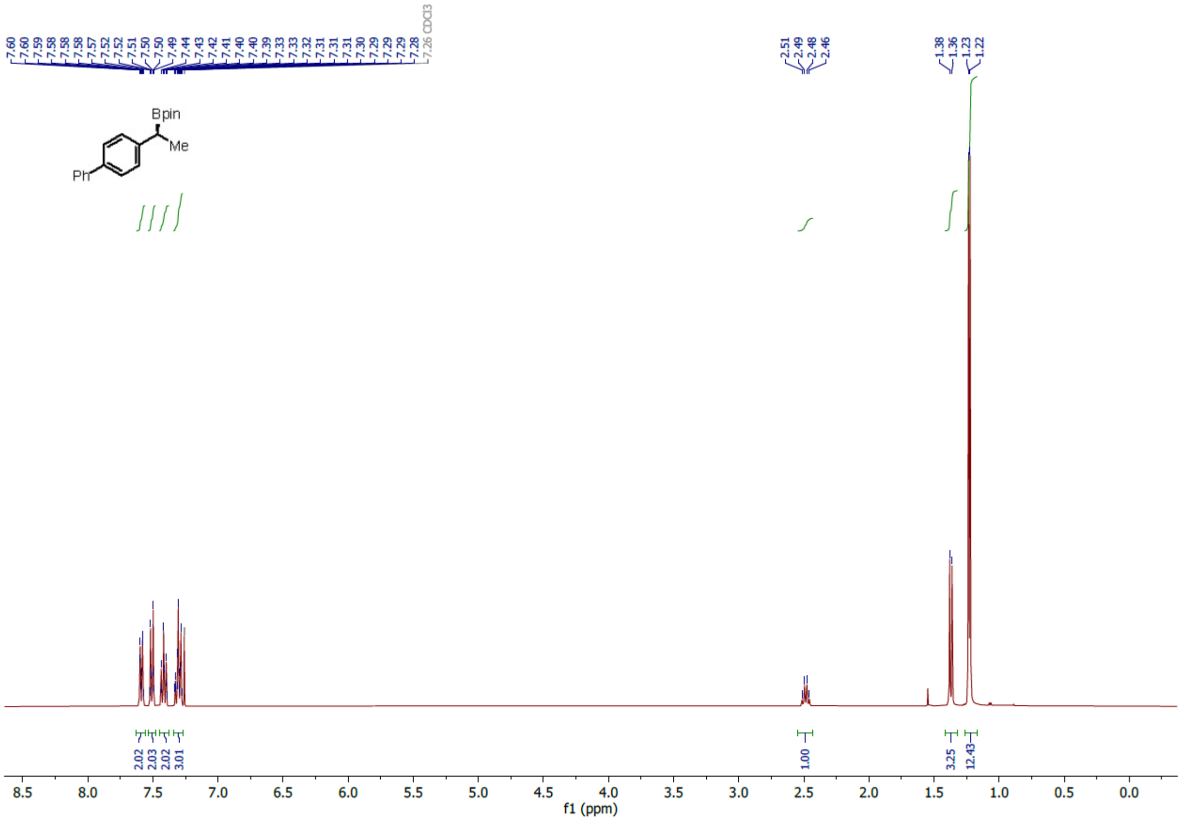
7.60 7.60 7.59 7.58 7.58 7.58 7.57 7.52 7.52 7.51 7.50 7.50 7.49 7.44 7.43 7.42 7.41 7.40 7.40 7.39 7.33 7.33 7.32 7.31 7.31 7.30 7.29 7.29 7.29 7.28 7.26 CDCl3
2.51 2.49 2.48 2.46
1.38 1.36 1.23 1.22
Bpin
Me
Ph
2.02 2.03 2.02 3.01
1.00
3.25 12.43
f1 (ppm)

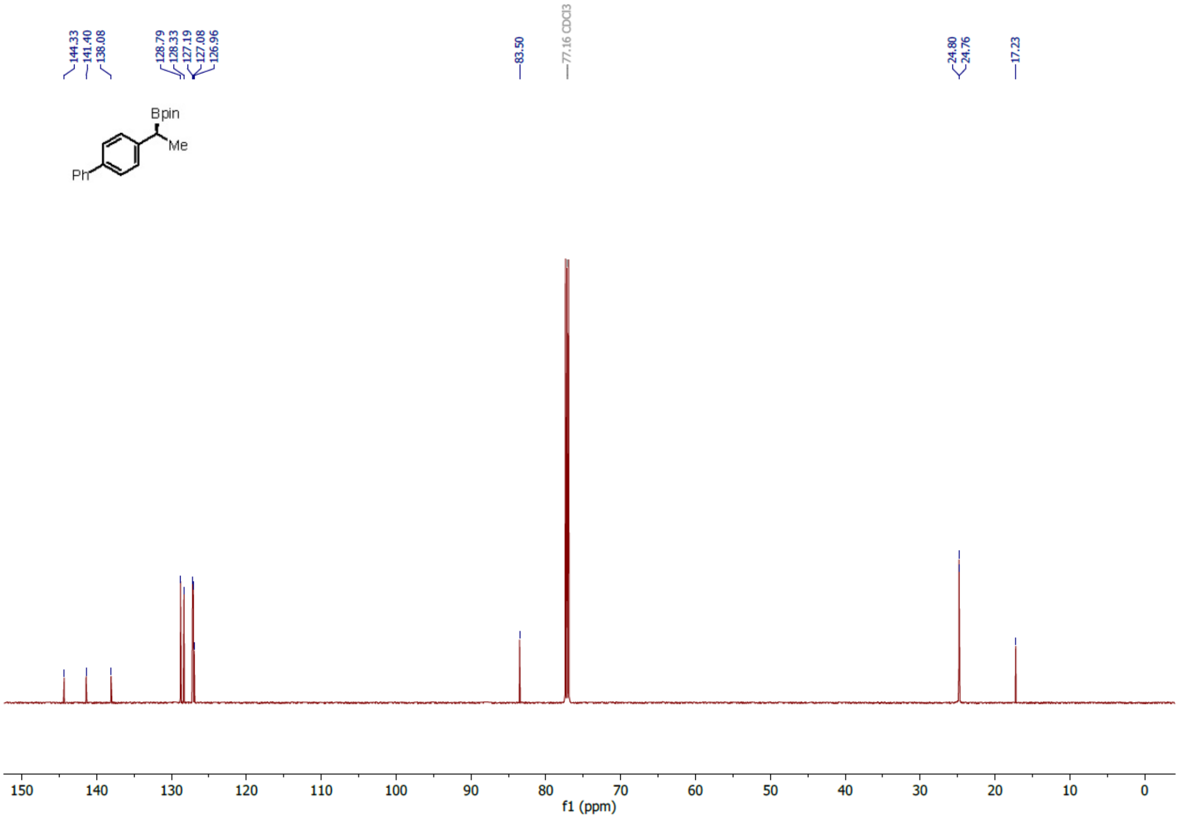
144.33 141.40 138.08
128.79 128.33 127.19 127.08 126.96
83.50
77.16 CDCl3
24.80 24.76
17.23
Bpin
Me
Ph
f1 (ppm)

Compound **28**

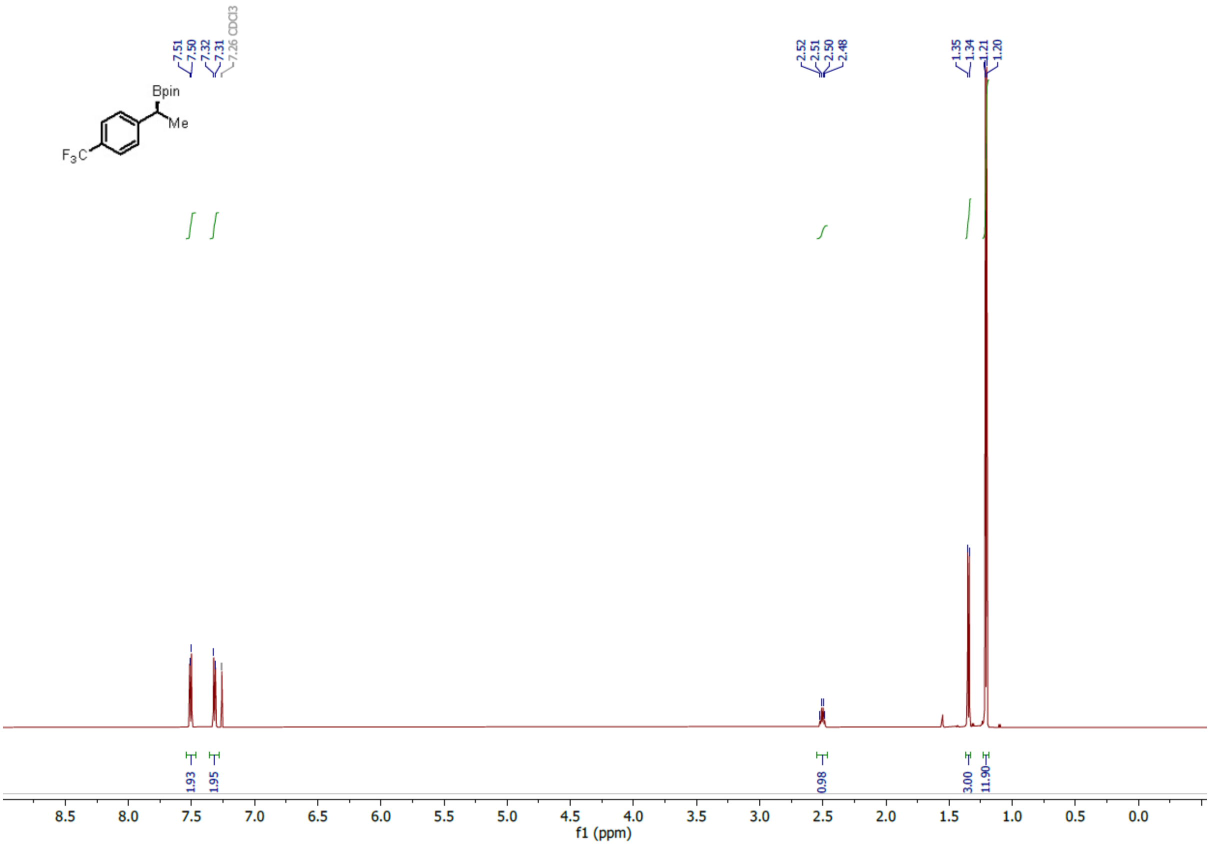

7.51
7.50
7.32
7.31
7.26 CDCl3
2.52
2.51
2.50
2.48
1.35
1.34
1.21
1.20
Bpin
Me
F3C
1.93
1.95
0.98
3.00
11.90
8.5 8.0 7.5 7.0 6.5 6.0 5.5 5.0 4.5 4.0 3.5 3.0 2.5 2.0 1.5 1.0 0.5 0.0
f1 (ppm)

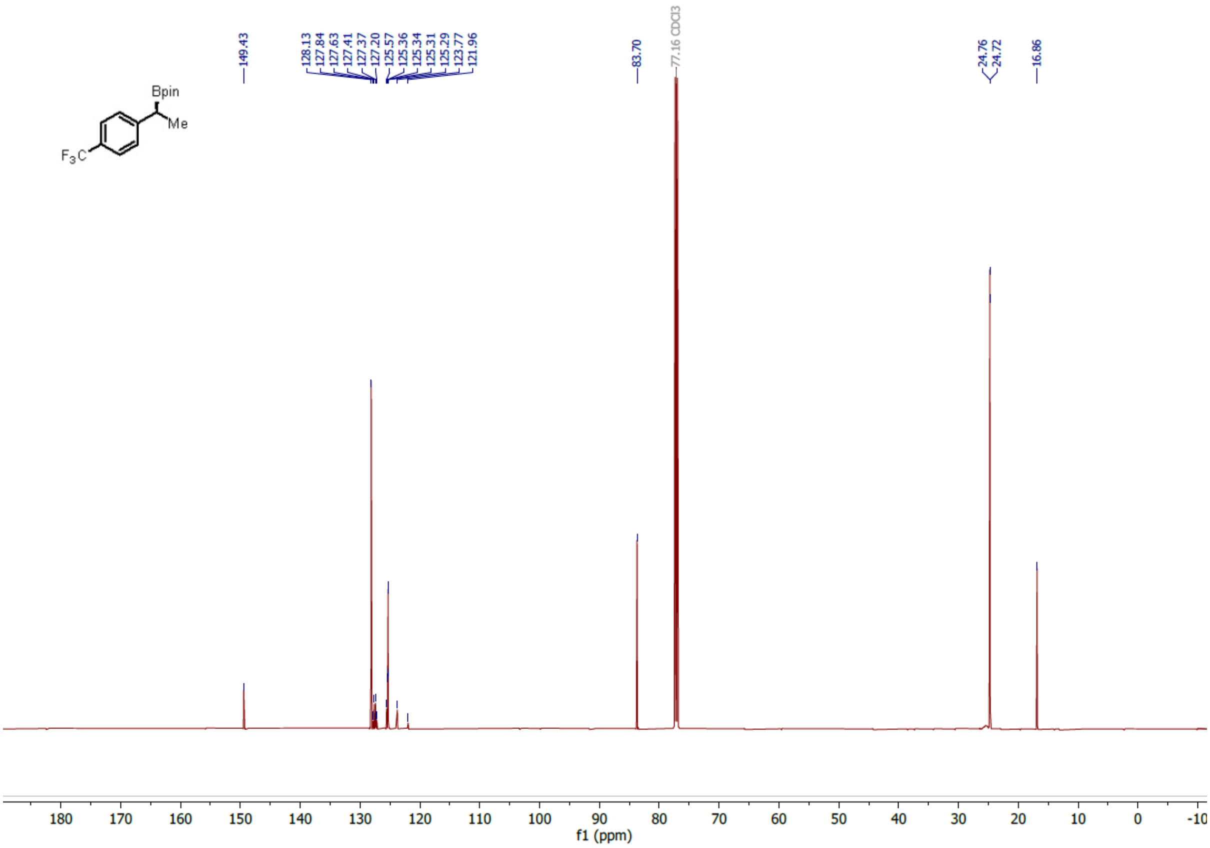

149.43
128.13
127.84
127.63
127.41
127.37
127.20
125.57
125.36
125.34
125.31
125.29
123.77
121.96
83.70
77.16 CDCl3
24.76
24.72
16.86
Bpin
Me
F3C
180 170 160 150 140 130 120 110 100 90 80 70 60 50 40 30 20 10 0 -10
f1 (ppm)

Compound **4**

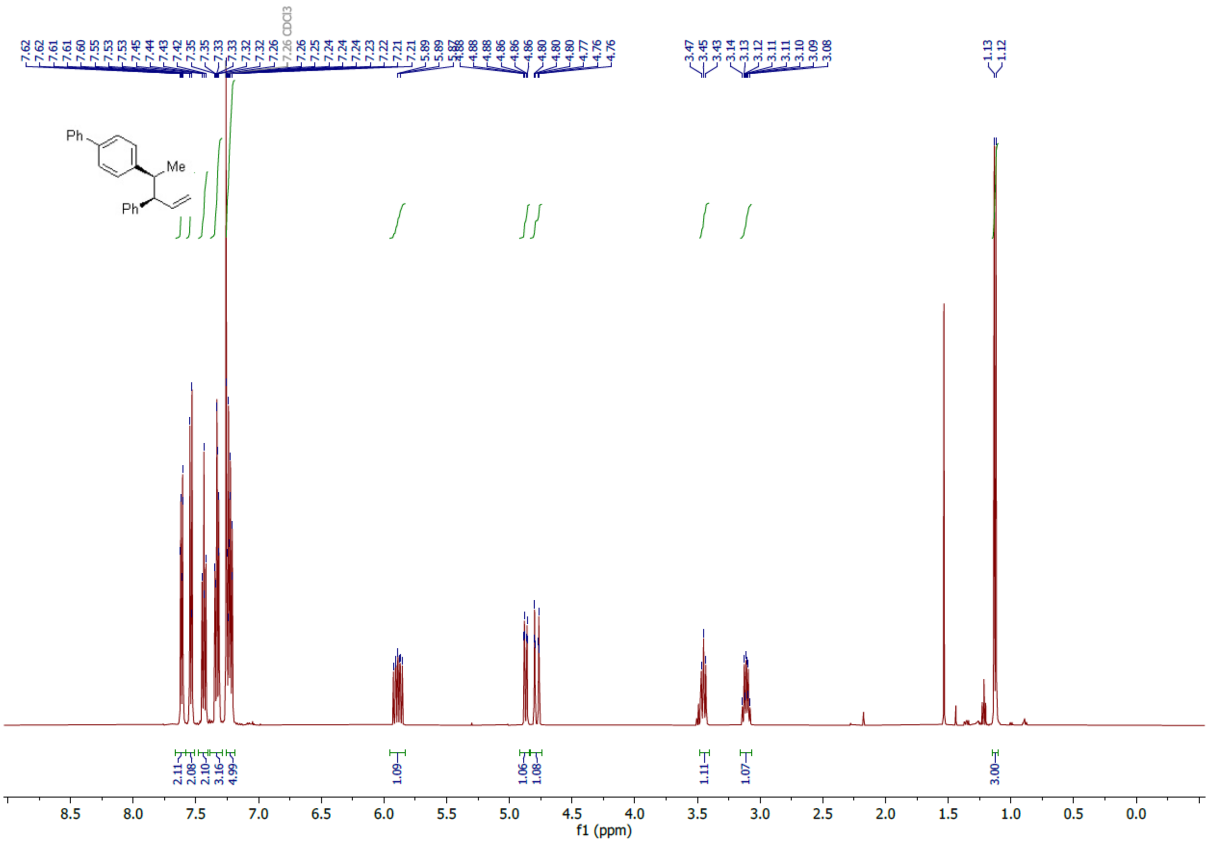

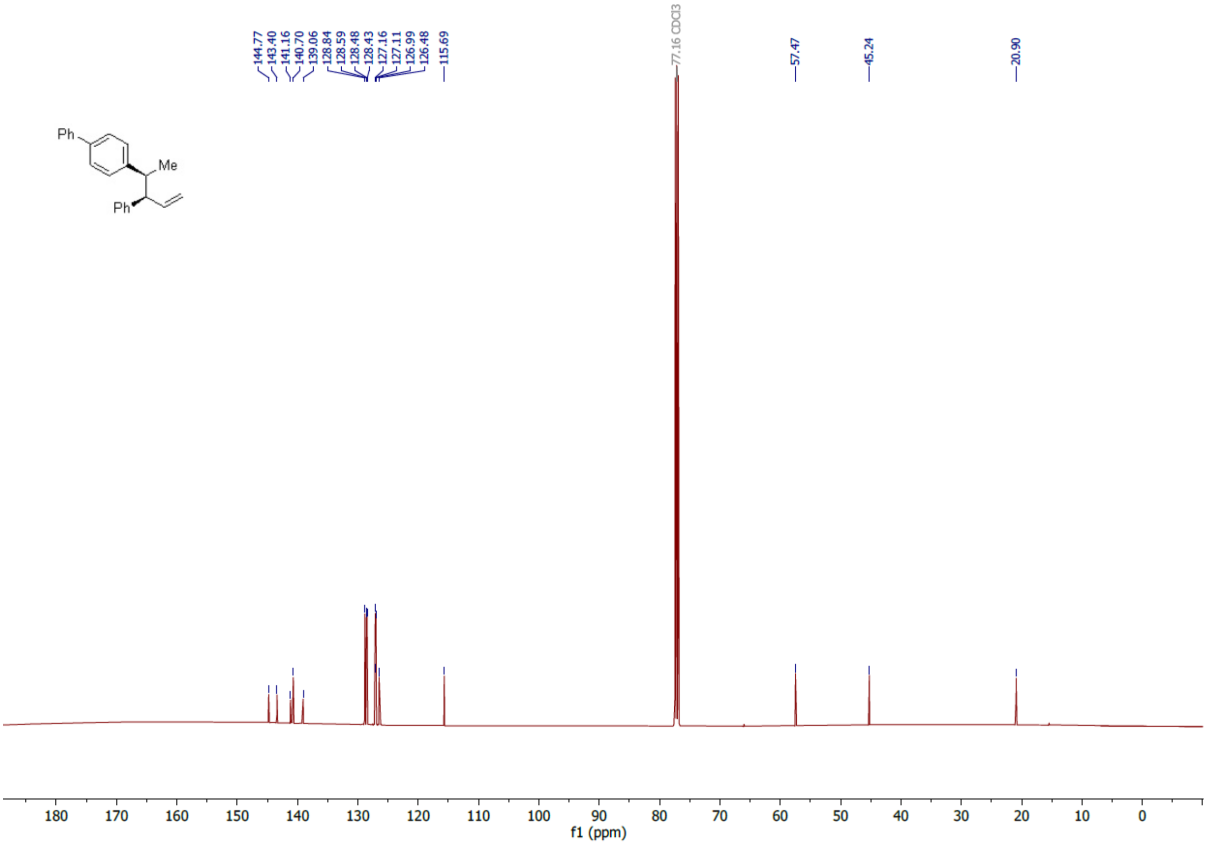

Compound **46**

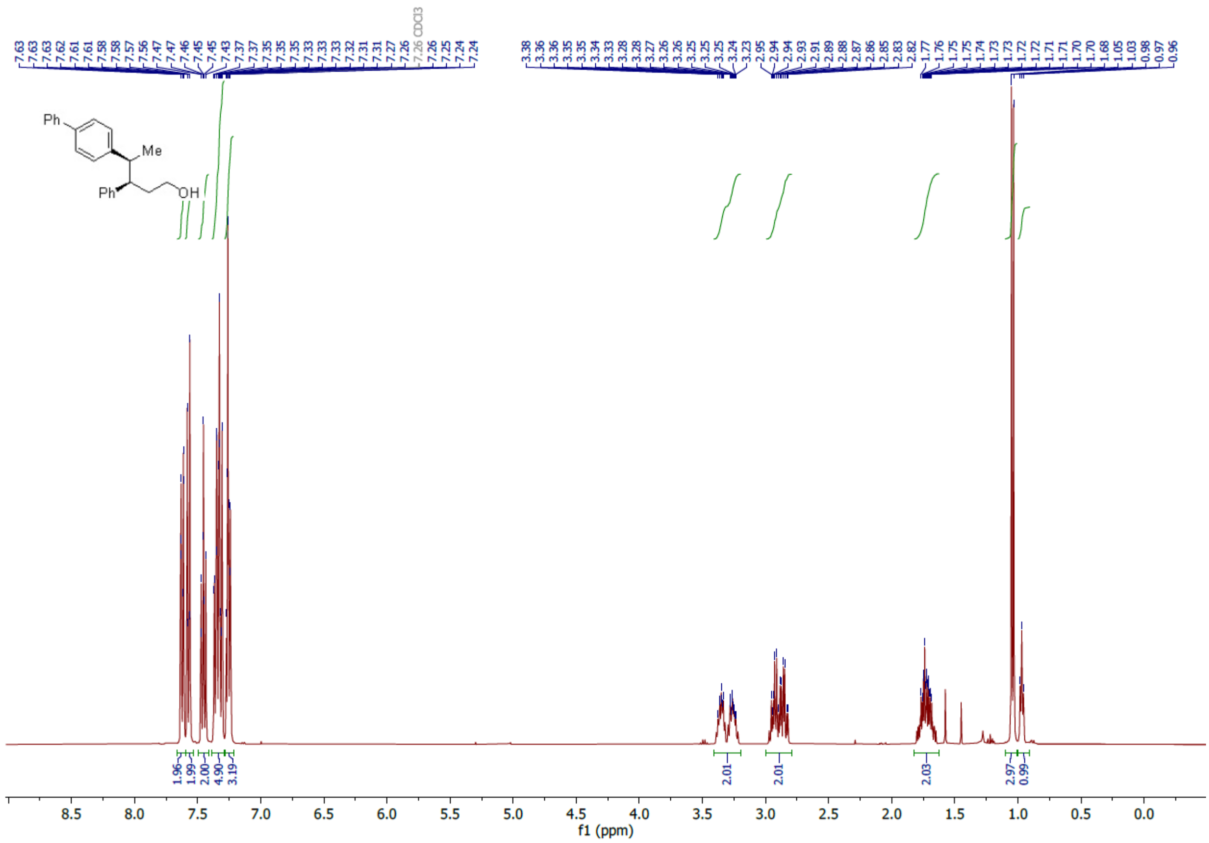

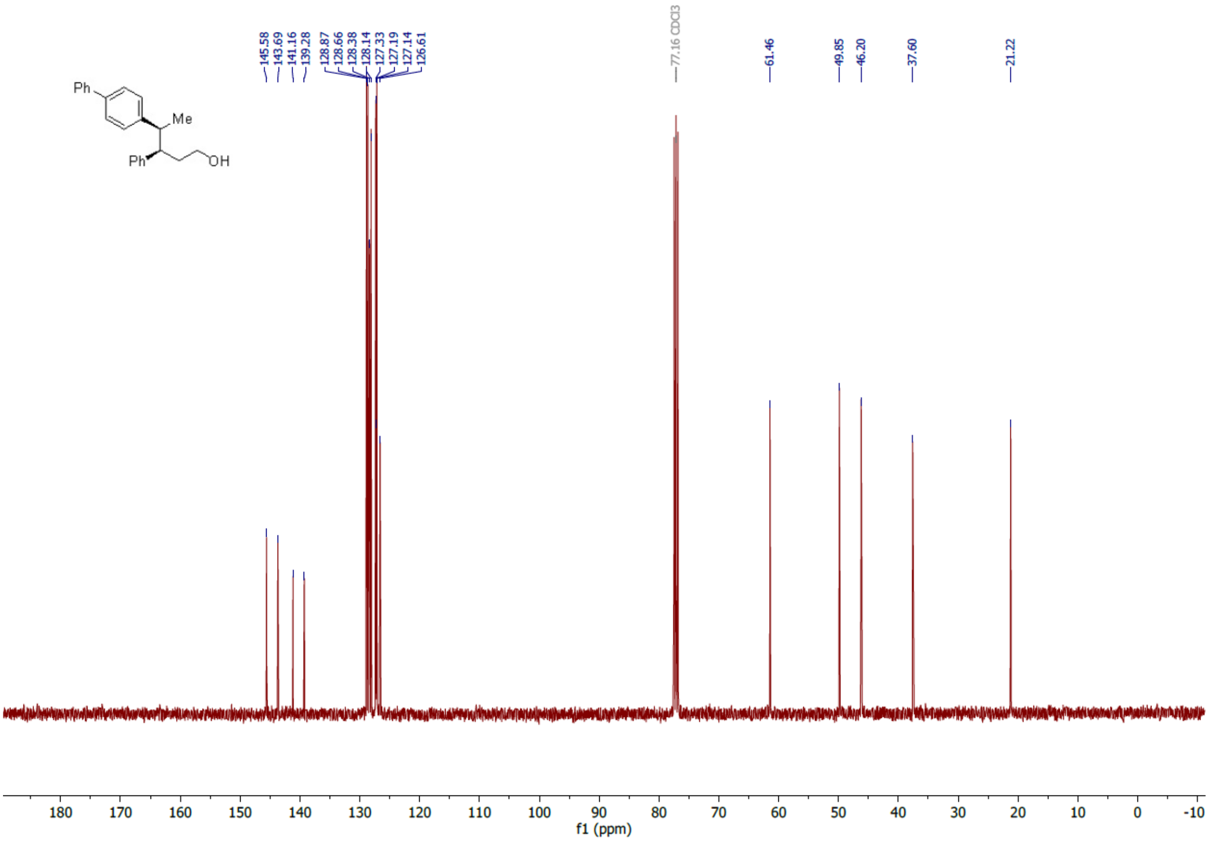

Compound **5**

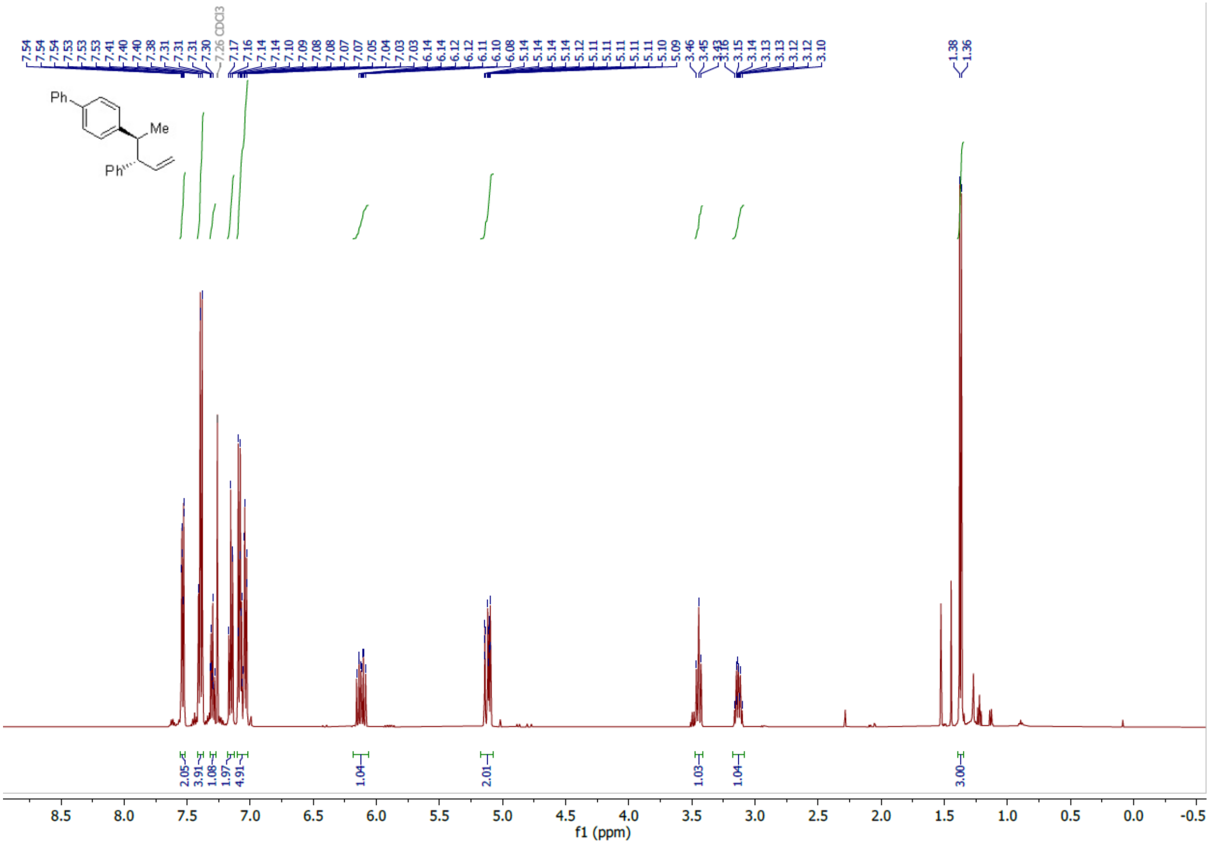

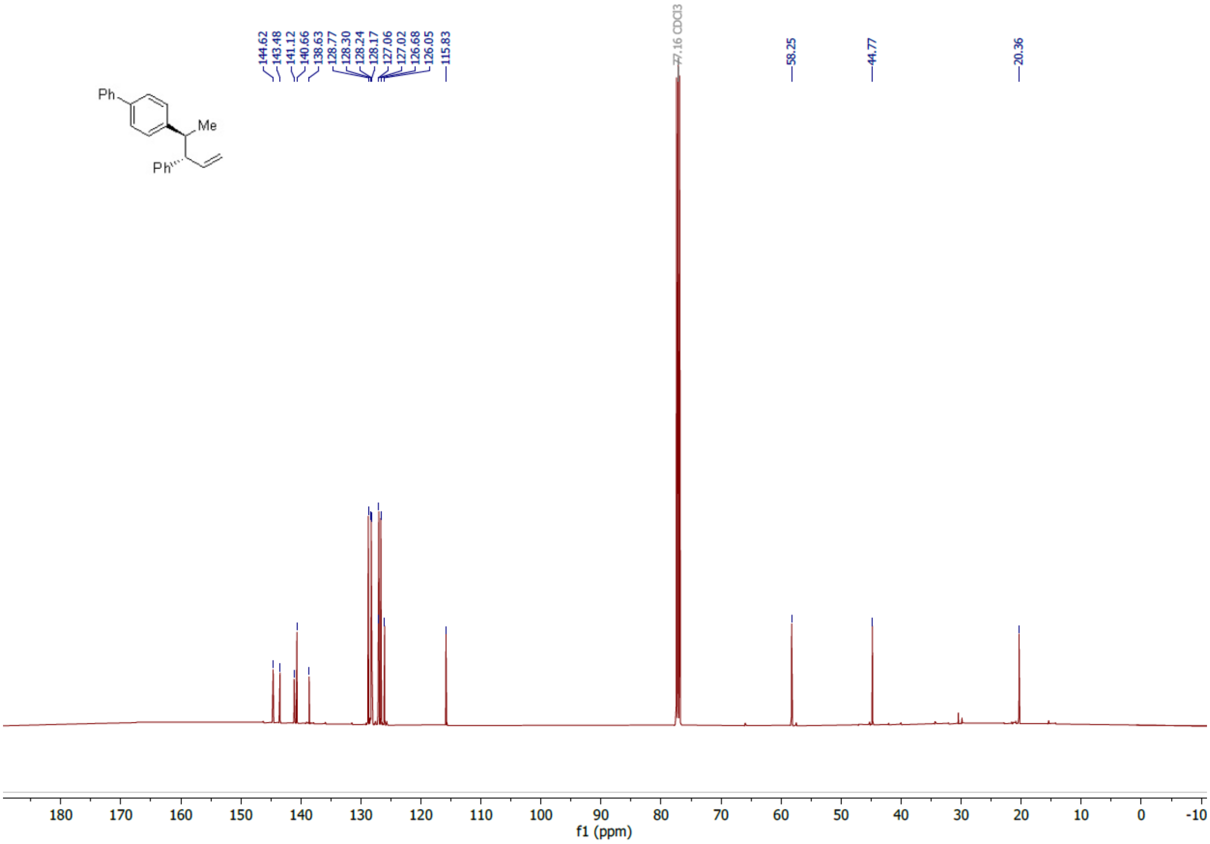

Compound **5'**

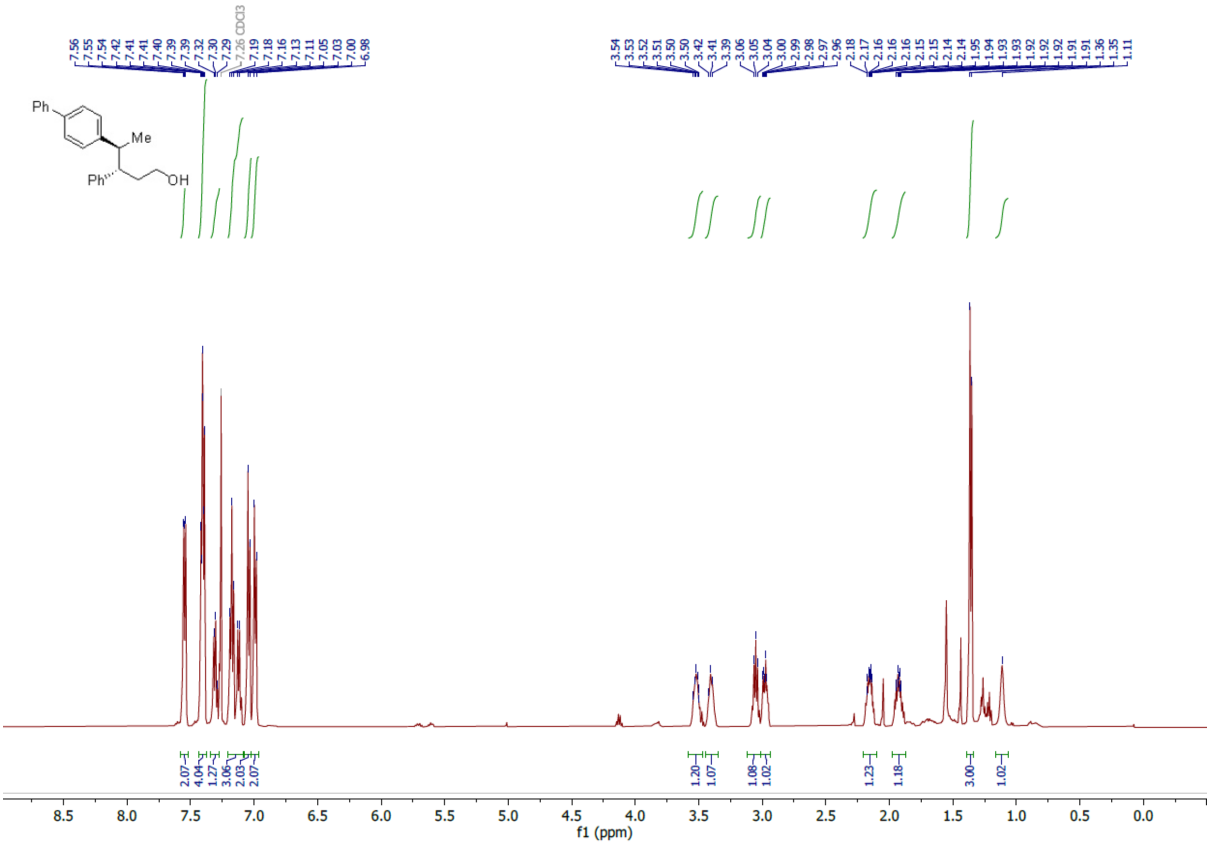
Ph
Me
Ph
OH
7.56 7.55 7.54 7.42 7.41 7.41 7.40 7.39 7.39 7.32 7.30 7.29 7.26 CDCl3 7.19 7.18 7.16 7.13 7.11 7.05 7.03 7.00 6.98
3.54 3.53 3.52 3.51 3.50 3.50 3.42 3.41 3.39 3.06 3.05 3.04 3.00 2.99 2.98 2.97 2.96 2.18 2.17 2.16 2.16 2.16 2.15 2.15 2.14 1.95 1.94 1.93 1.93 1.92 1.92 1.92 1.91 1.91 1.36 1.35 1.11
2.07 4.04 1.27 3.06 2.03 2.07 1.20 1.07 1.08 1.02 1.23 1.18 3.00 1.02
f1 (ppm)

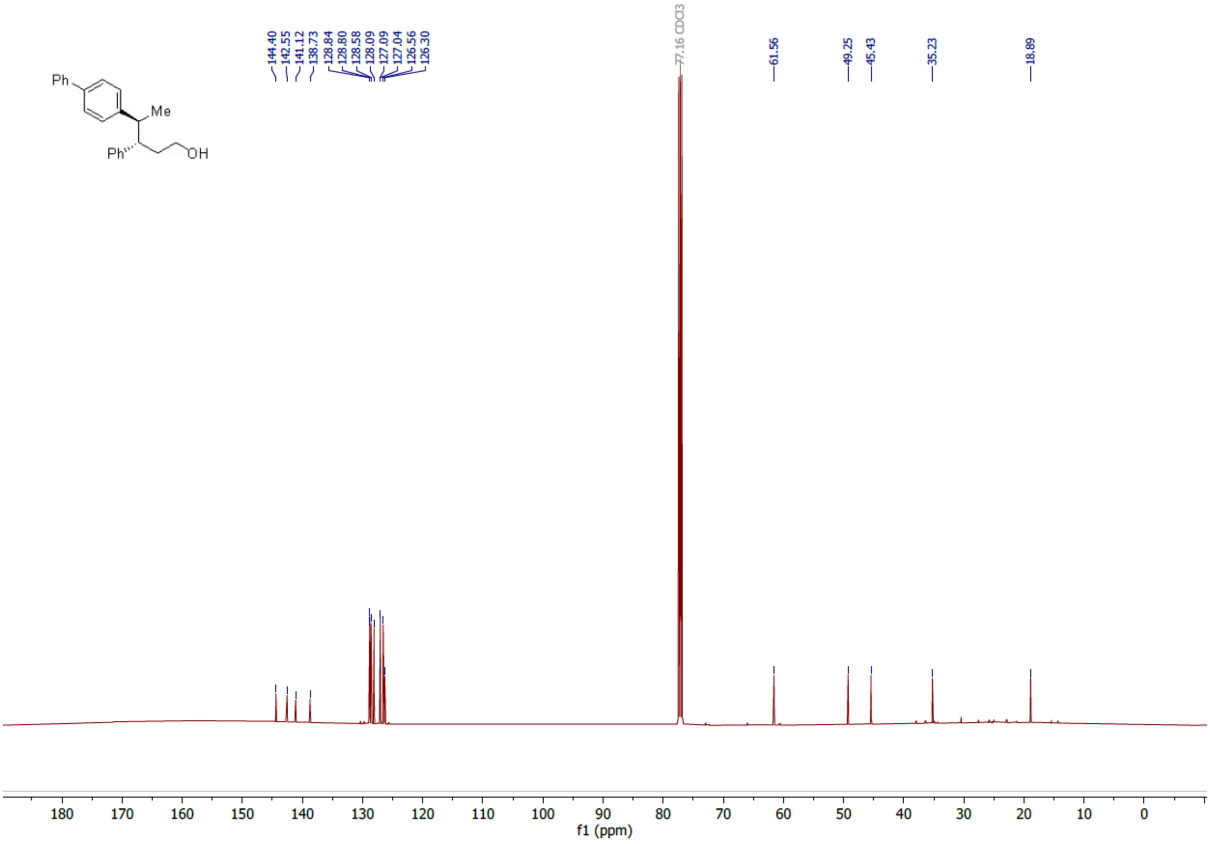
Ph
Me
Ph
OH
144.40 142.55 141.12 138.73 128.84 128.80 128.58 128.09 127.09 127.04 126.56 126.30
77.16 CDCl3
61.56
49.25
45.43
35.23
18.89
f1 (ppm)

Compound **6**

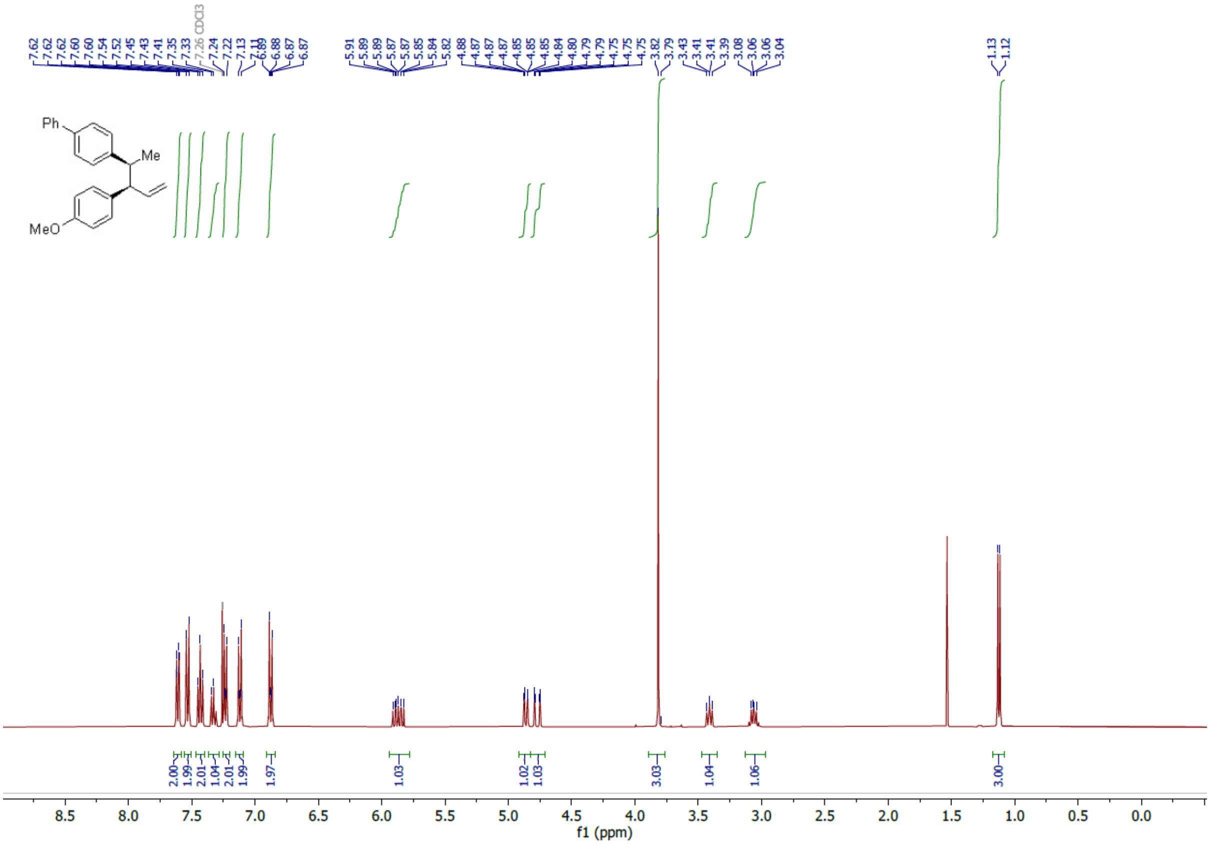

Ph
Me
MeO
f1 (ppm)

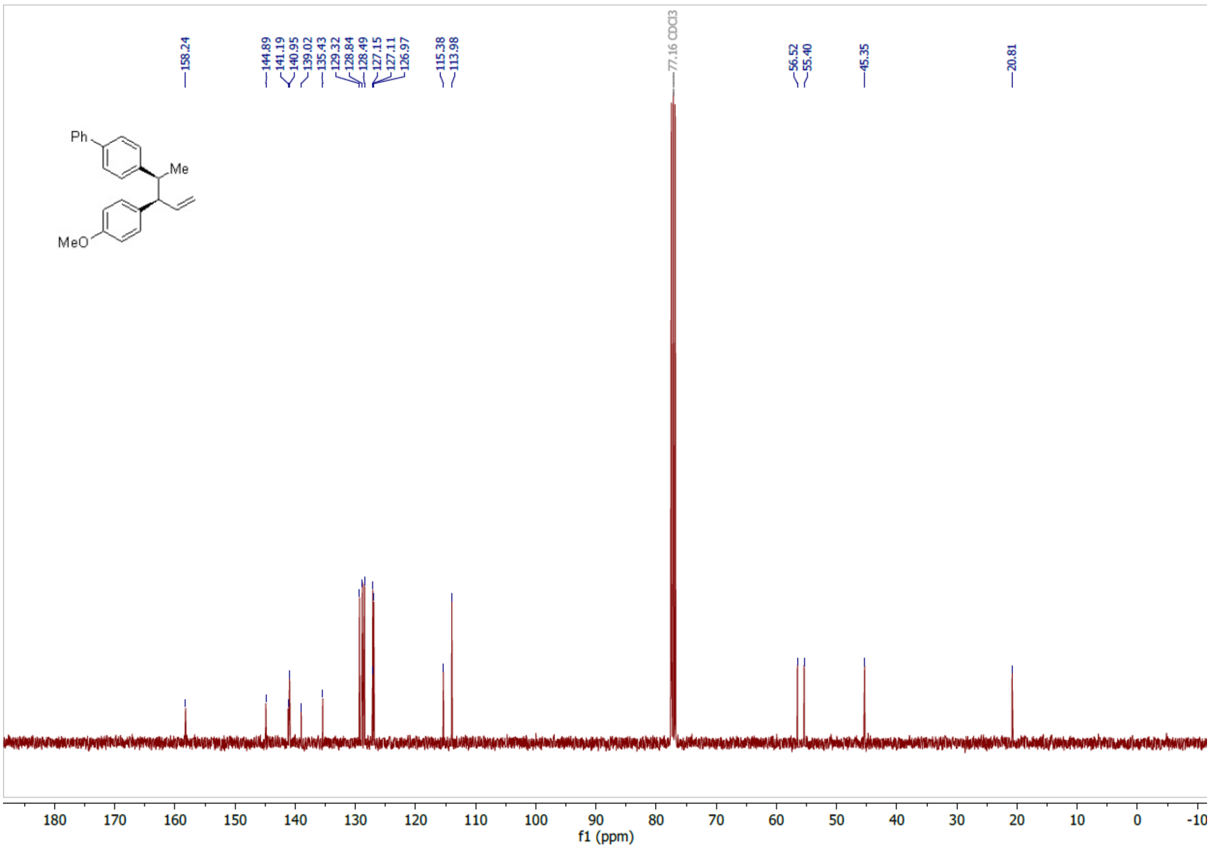

158.24
144.89
141.19
140.95
139.02
135.43
129.32
128.84
128.49
127.15
127.11
126.97
115.38
113.98
77.16 CDCl3
56.52
55.40
45.35
20.81
Ph
Me
MeO
f1 (ppm)

Compound **7**

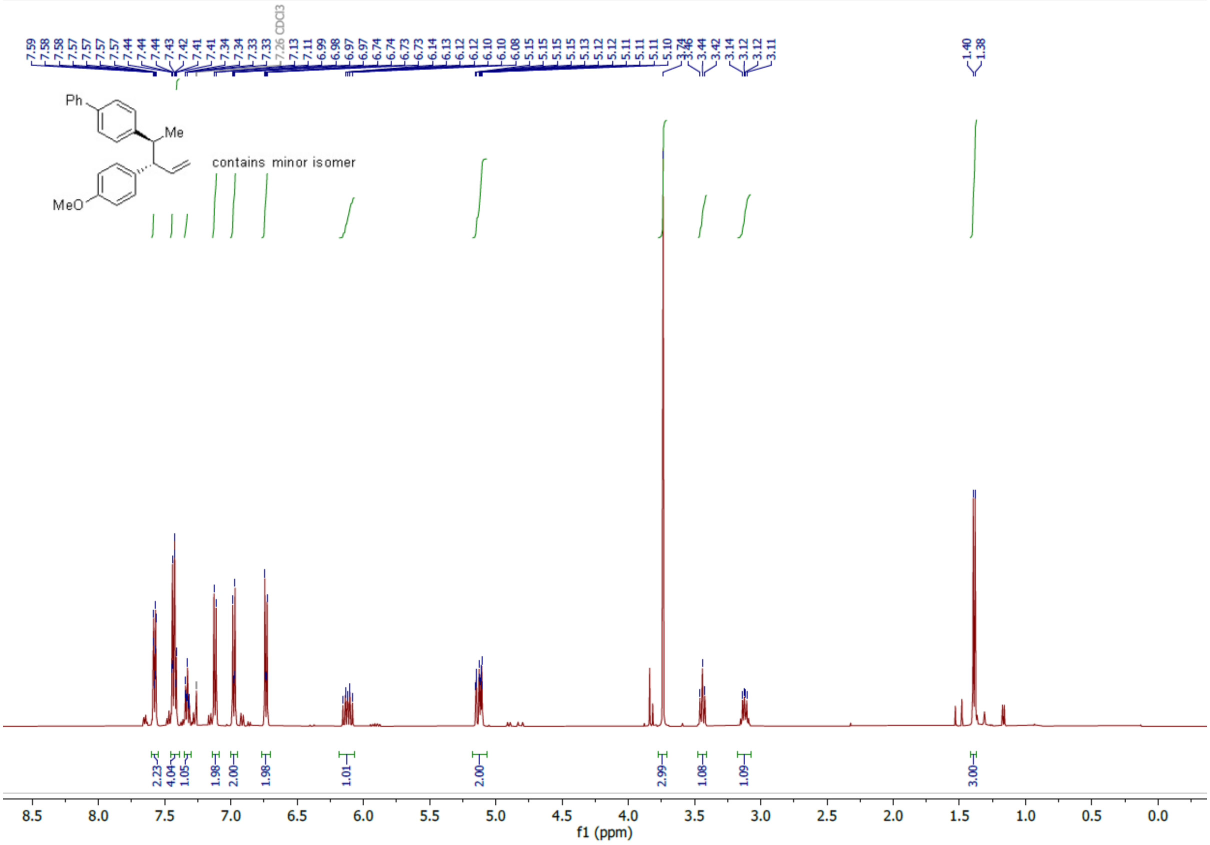

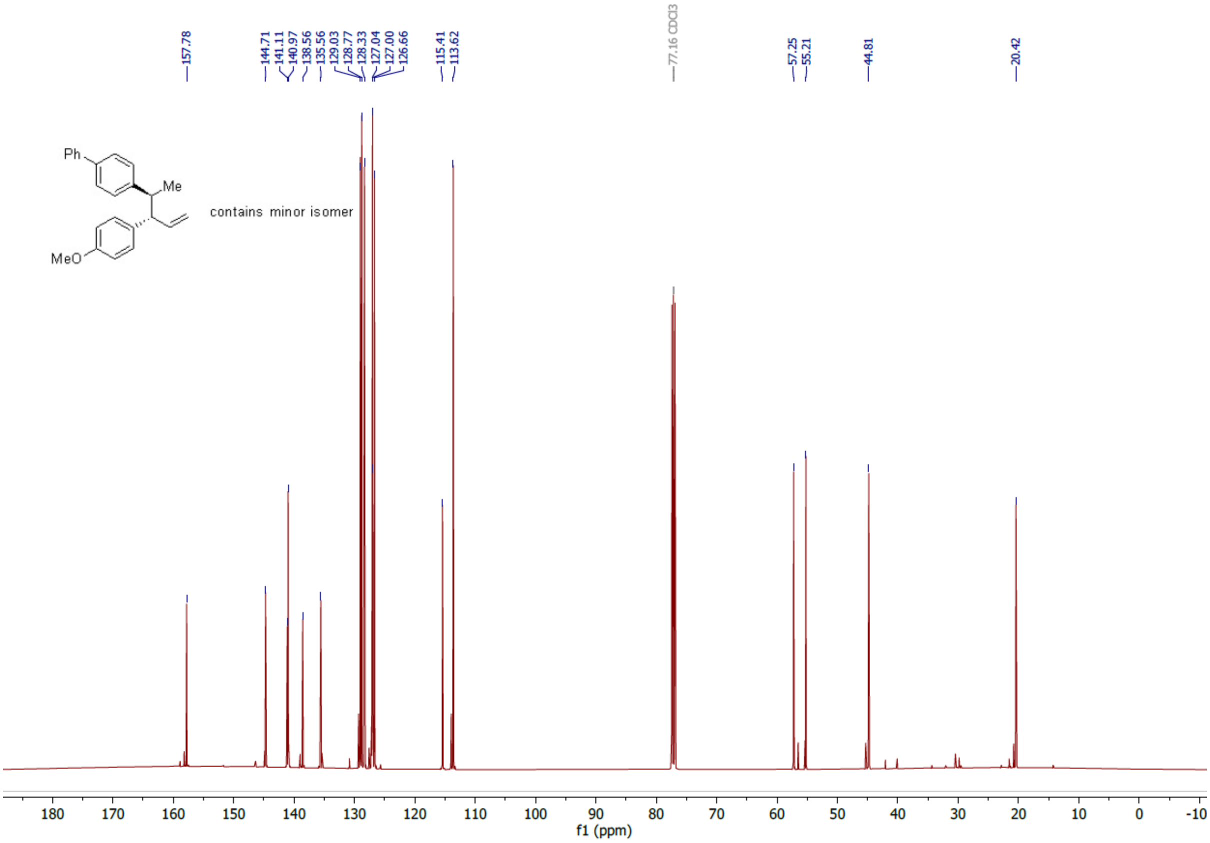

Compound **8**

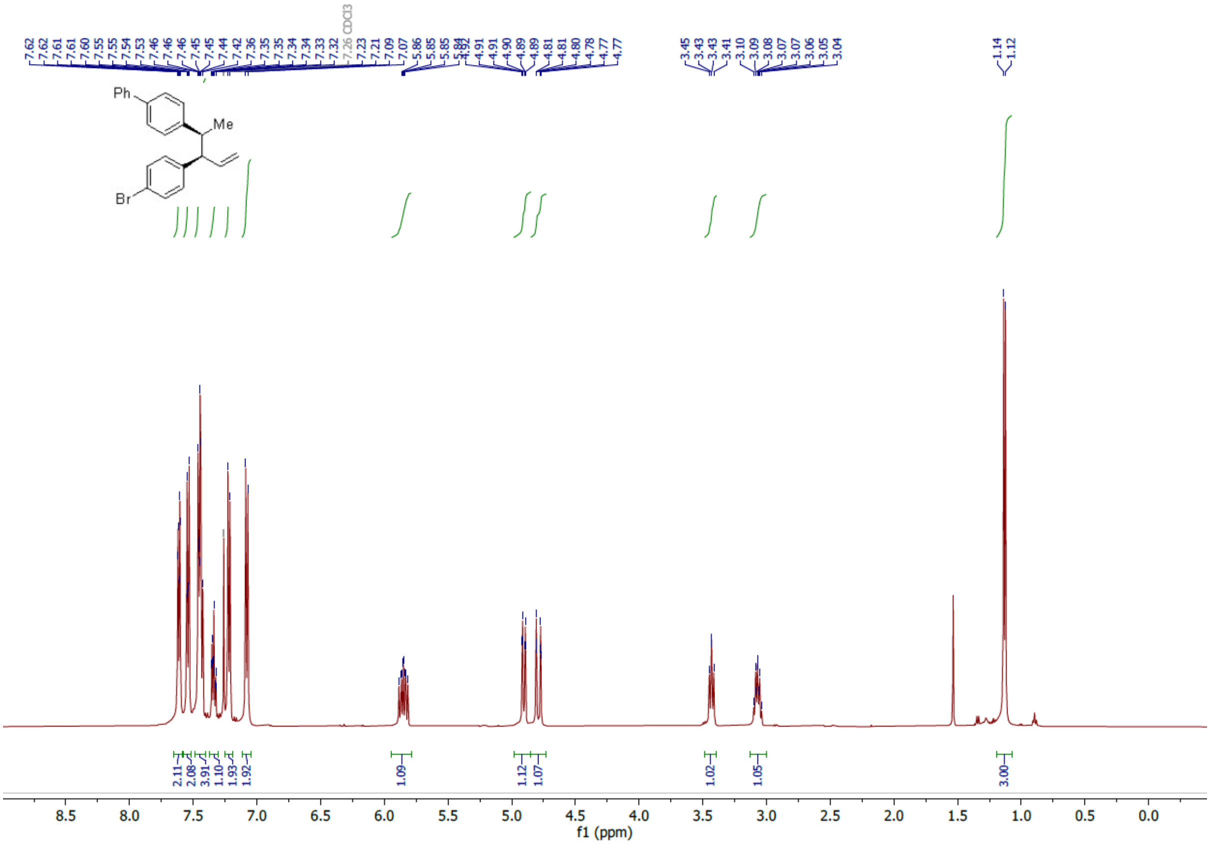

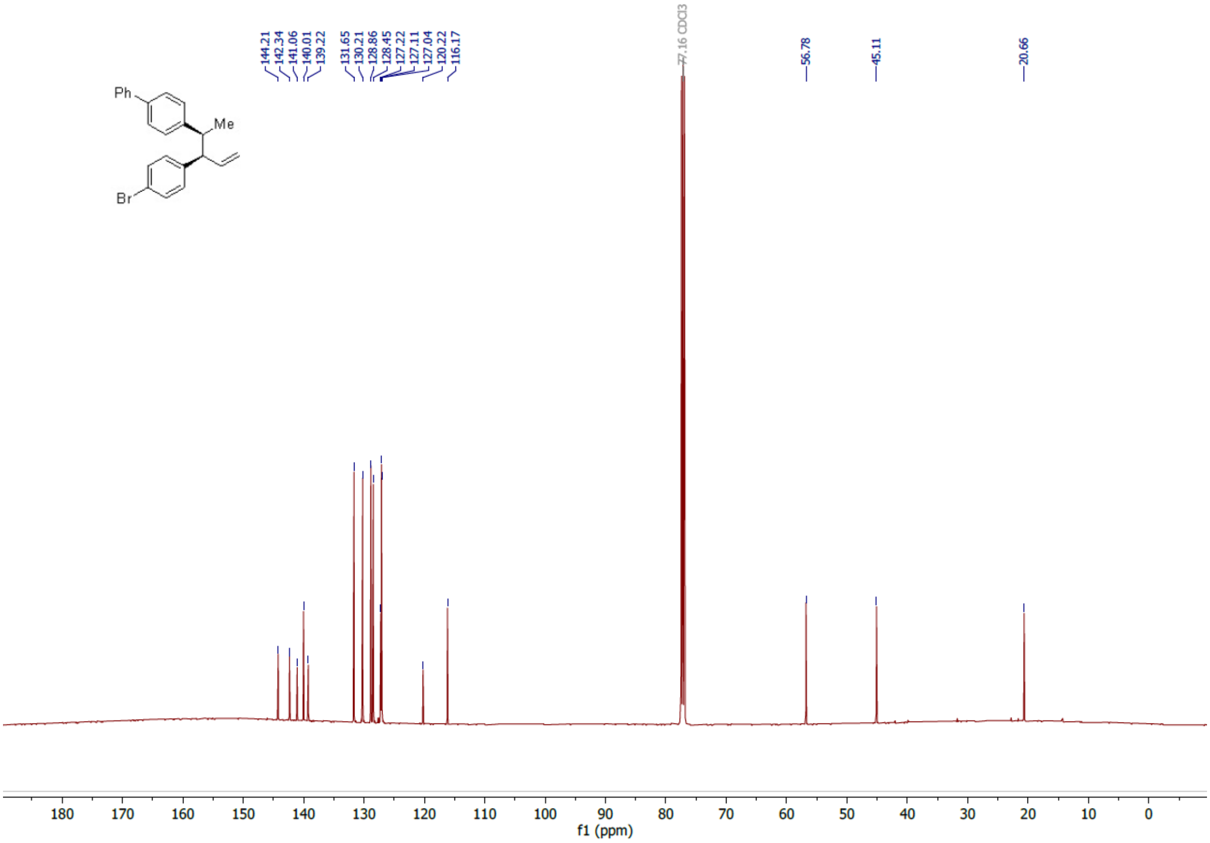

Compound **8'**

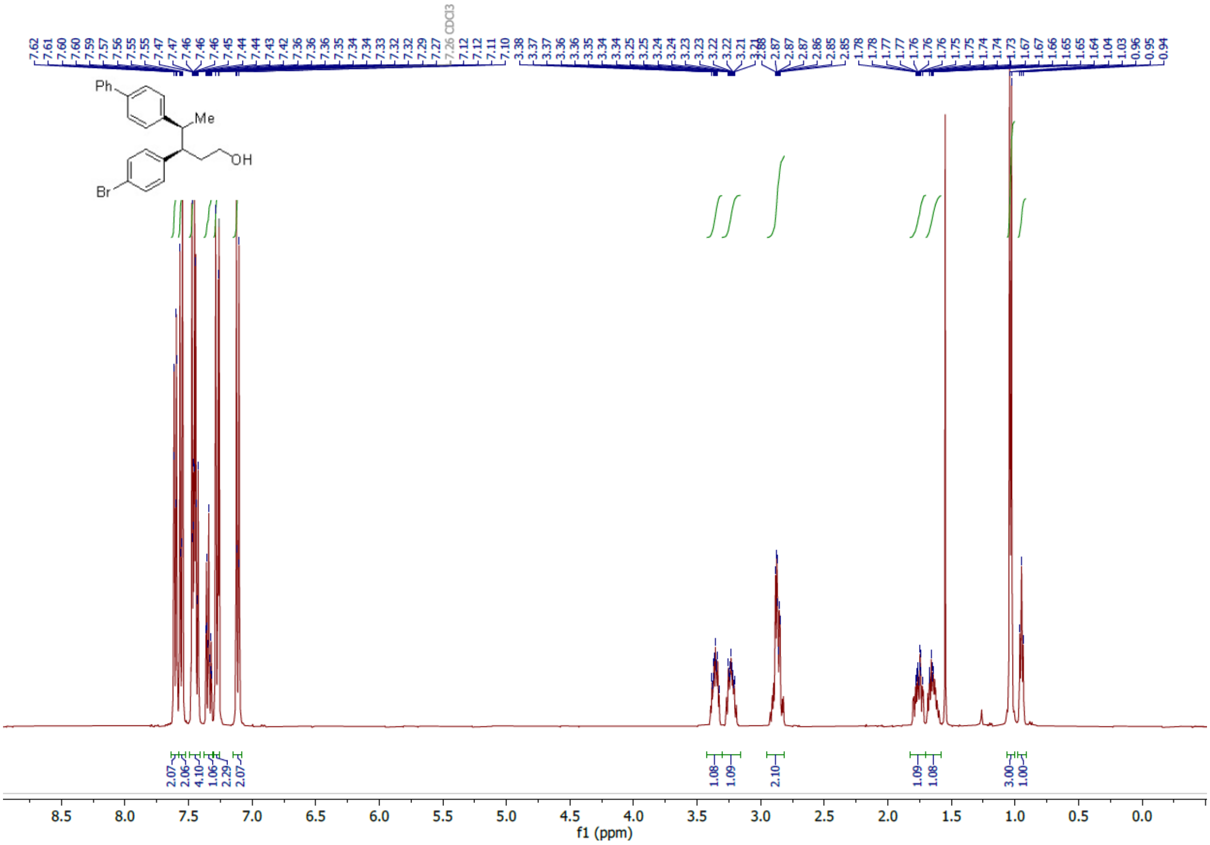

Ph
Me
OH
Br
f1 (ppm)

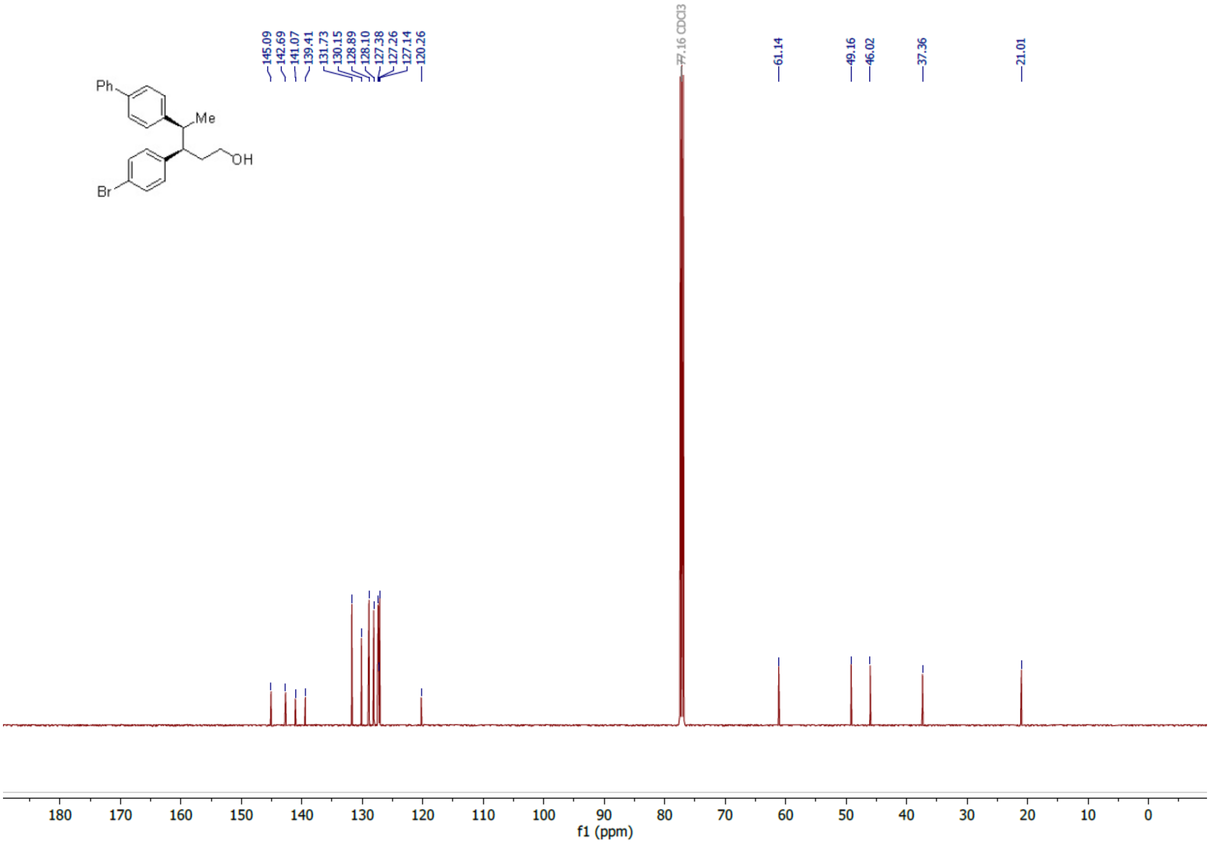

Ph
Me
OH
Br
145.09
142.69
141.07
139.41
131.73
130.15
128.89
128.10
127.38
127.26
127.14
120.26
77.16 CDCl3
61.14
49.16
46.02
37.36
21.01
f1 (ppm)

Compound **9**

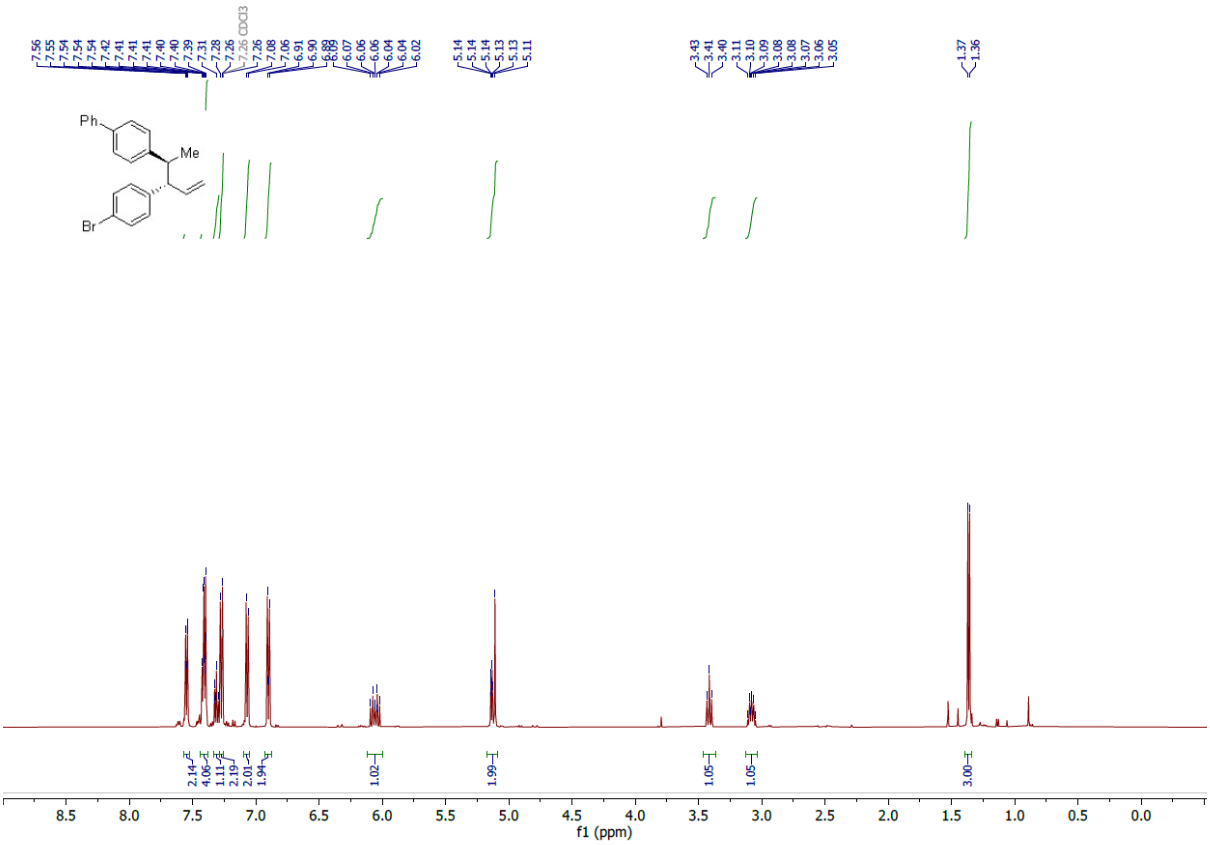

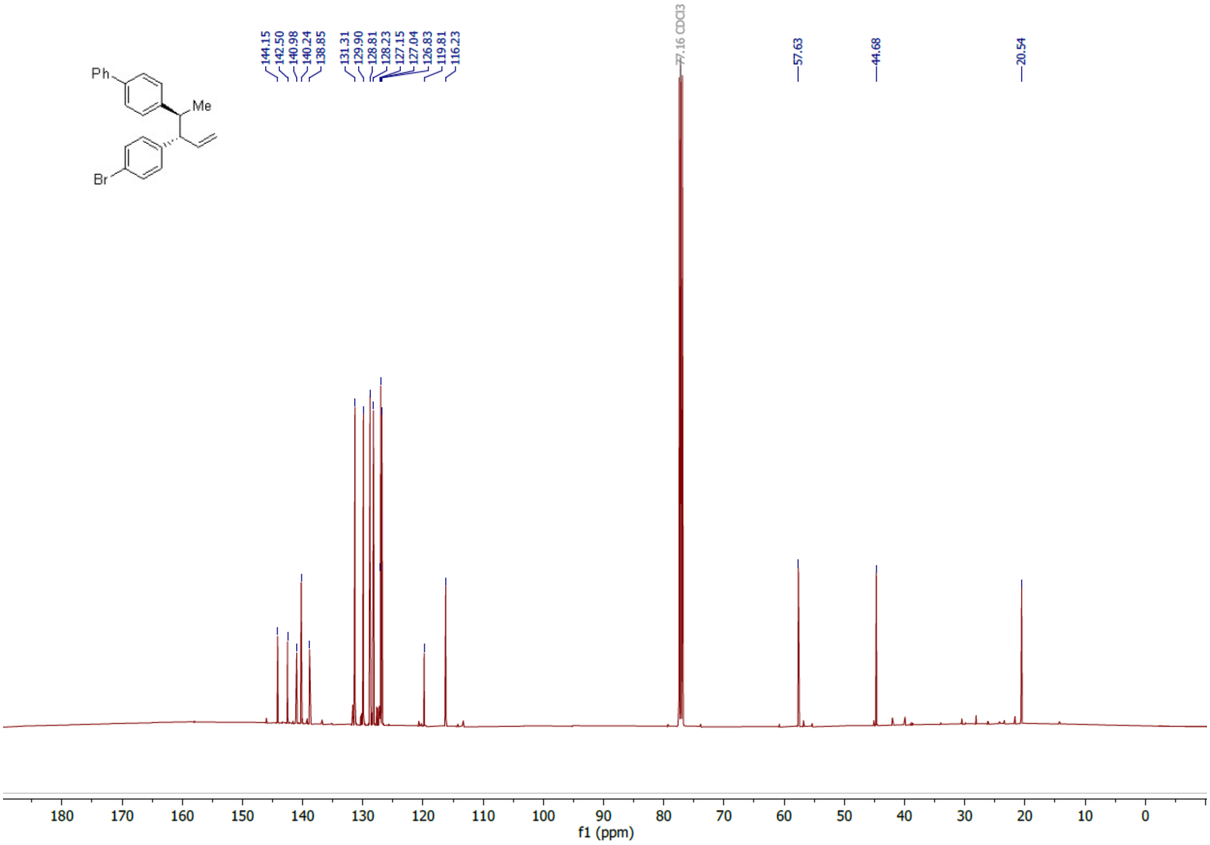

Compound **9'**

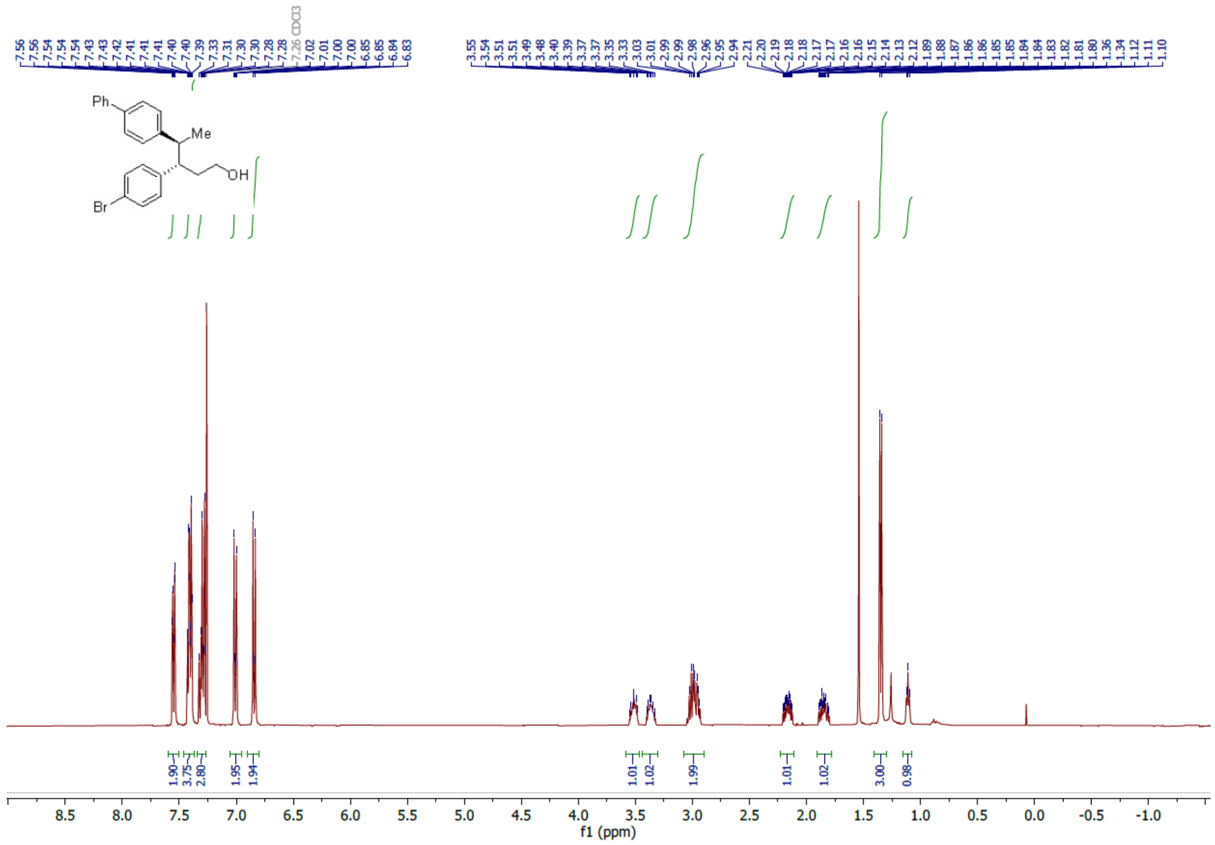

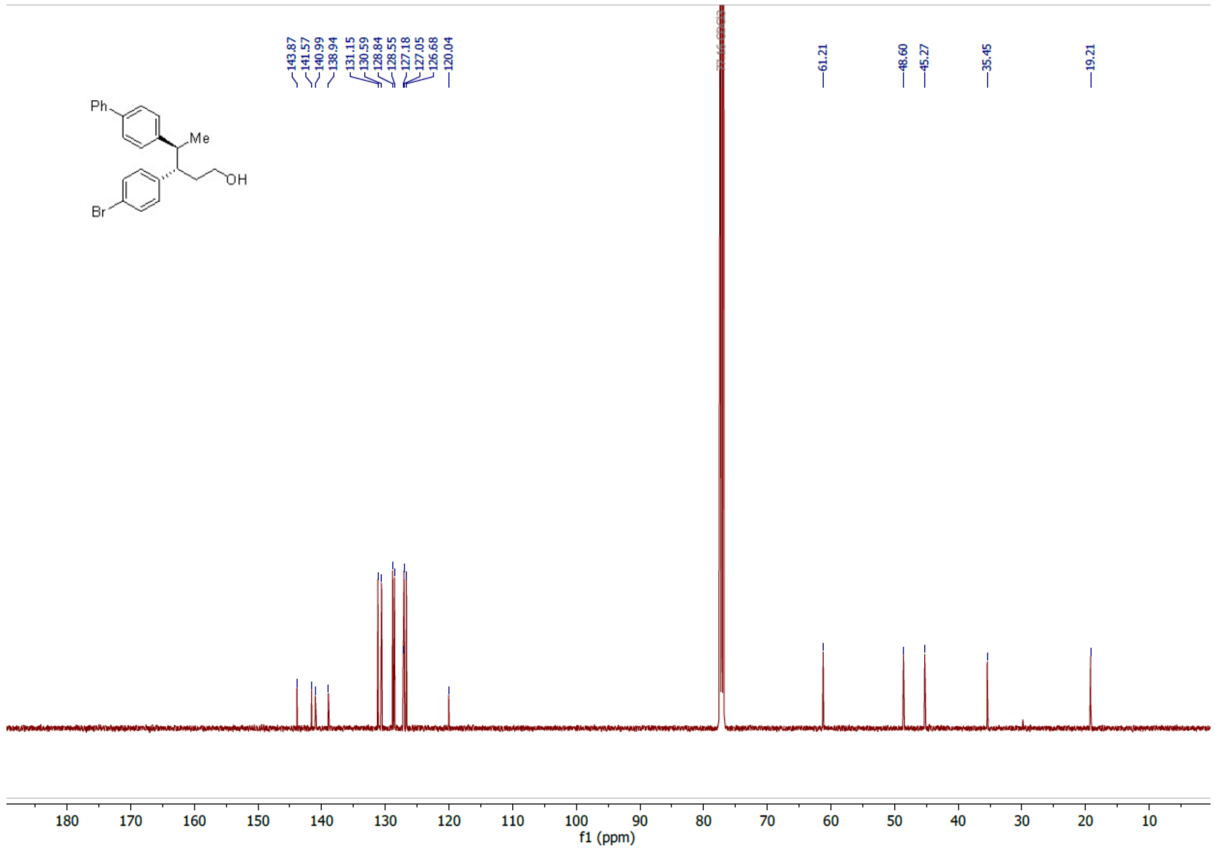

Compound **10**

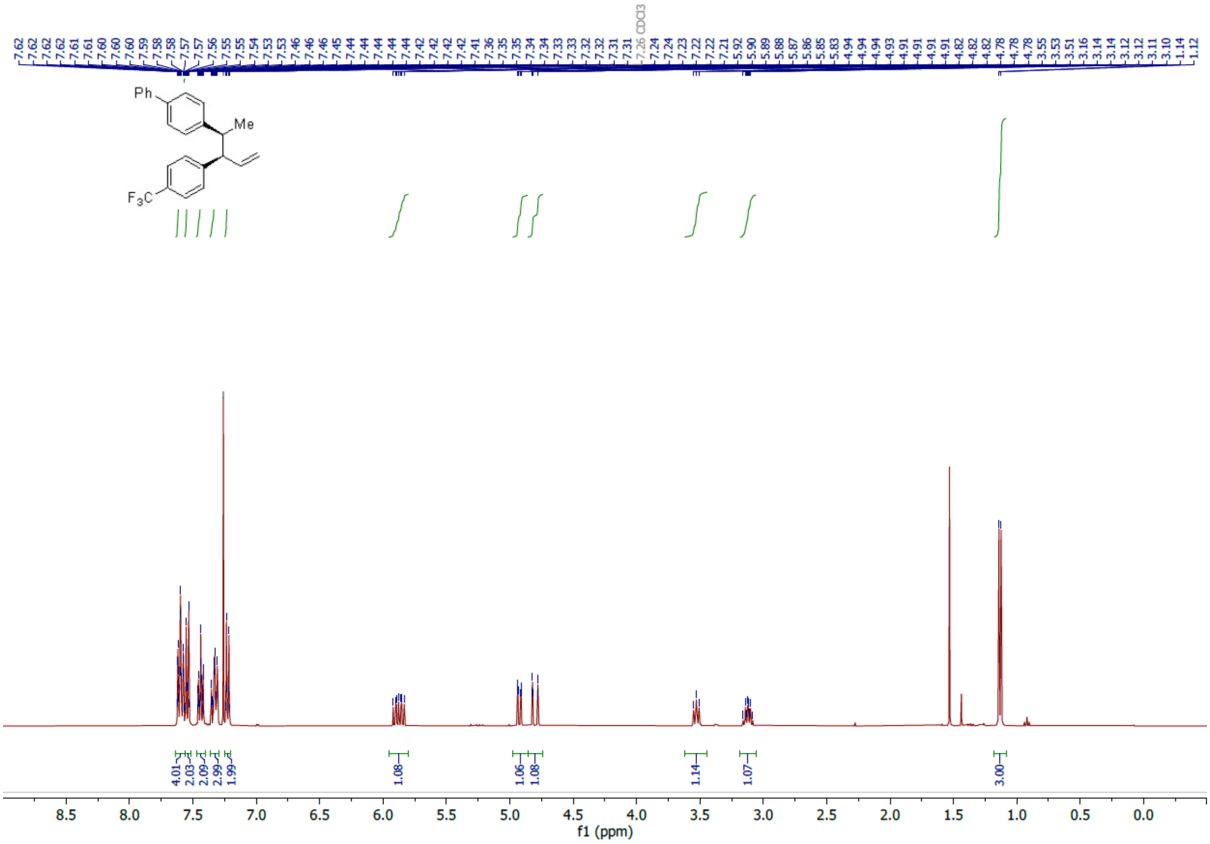

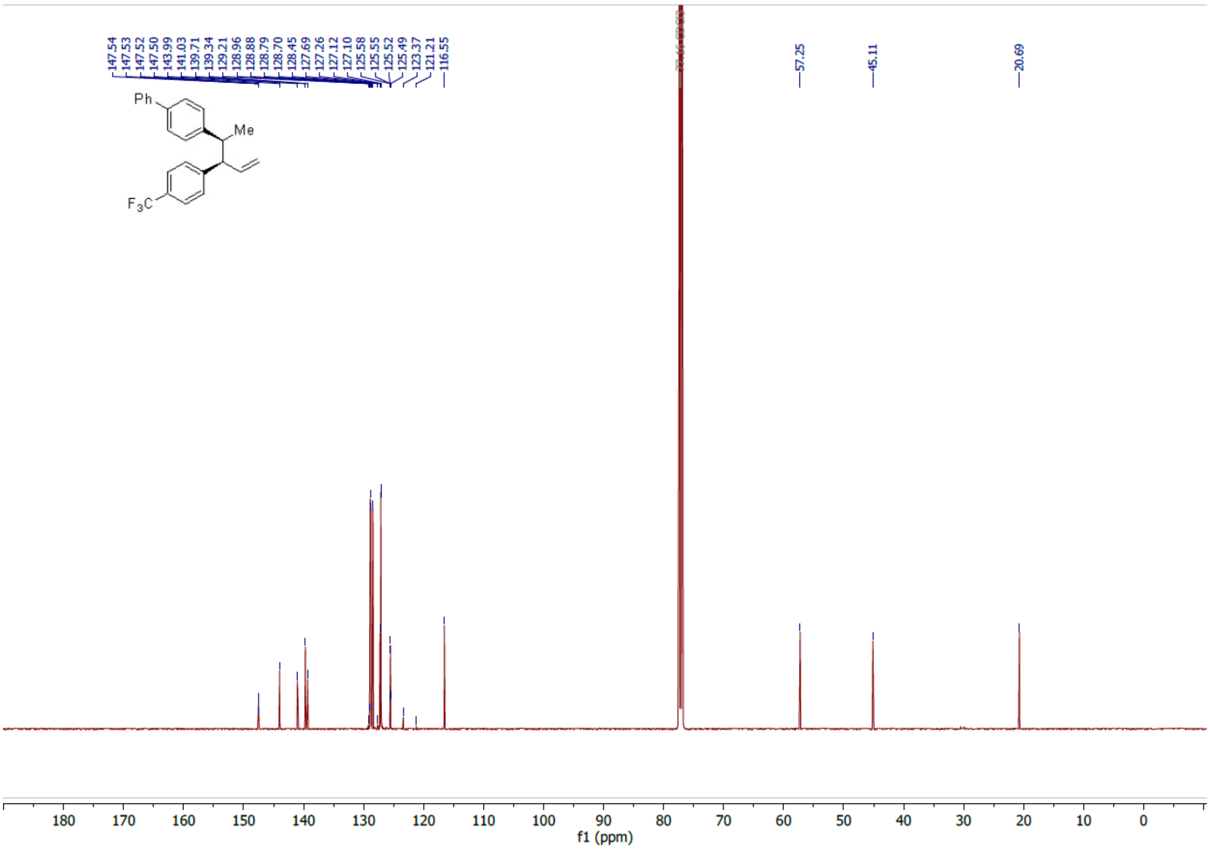

Compound **10'**

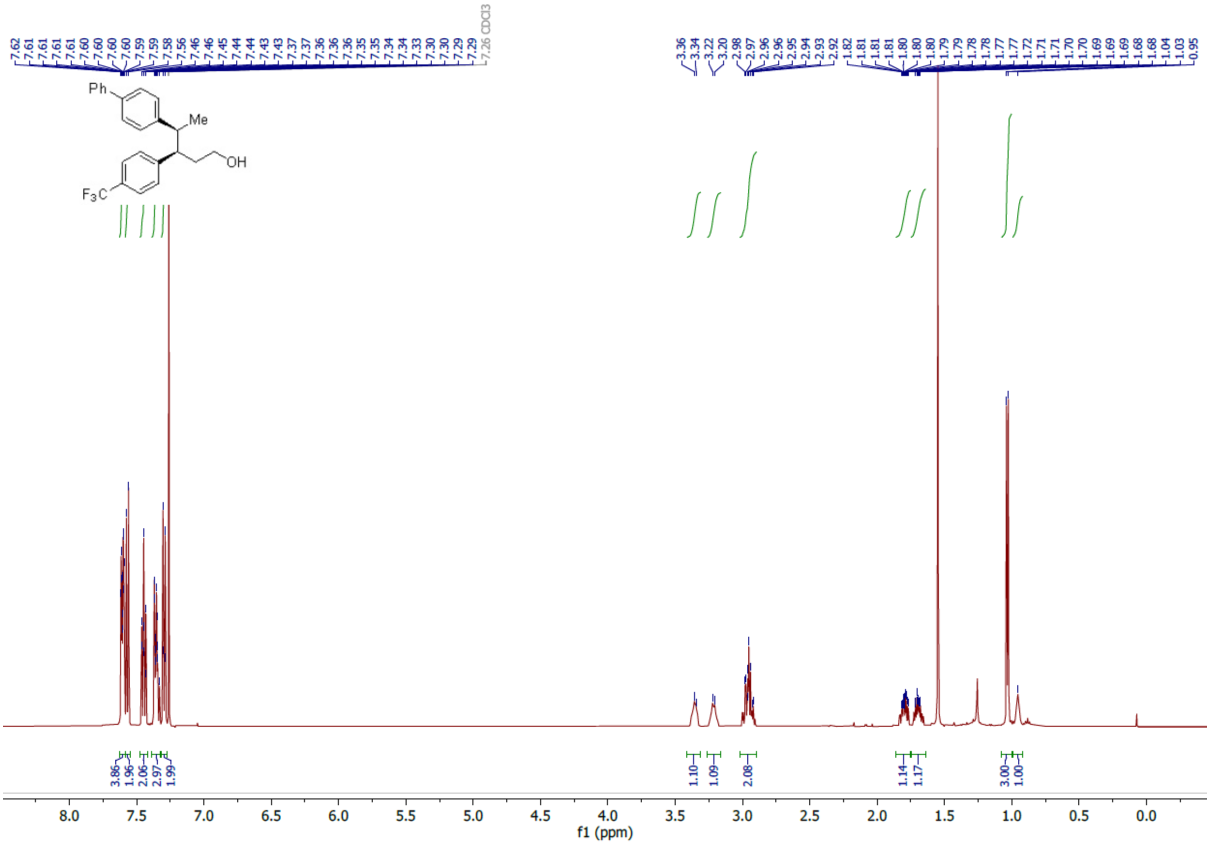

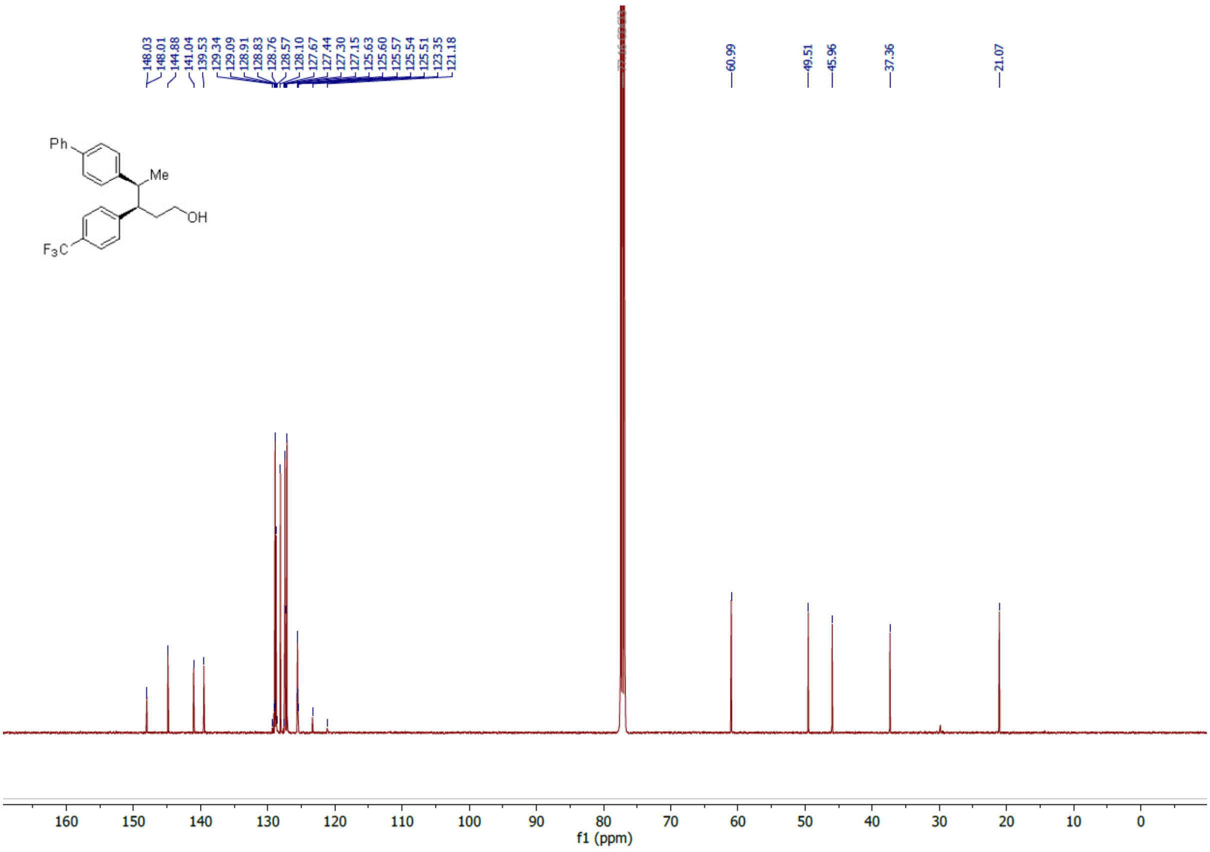

Compound **11**

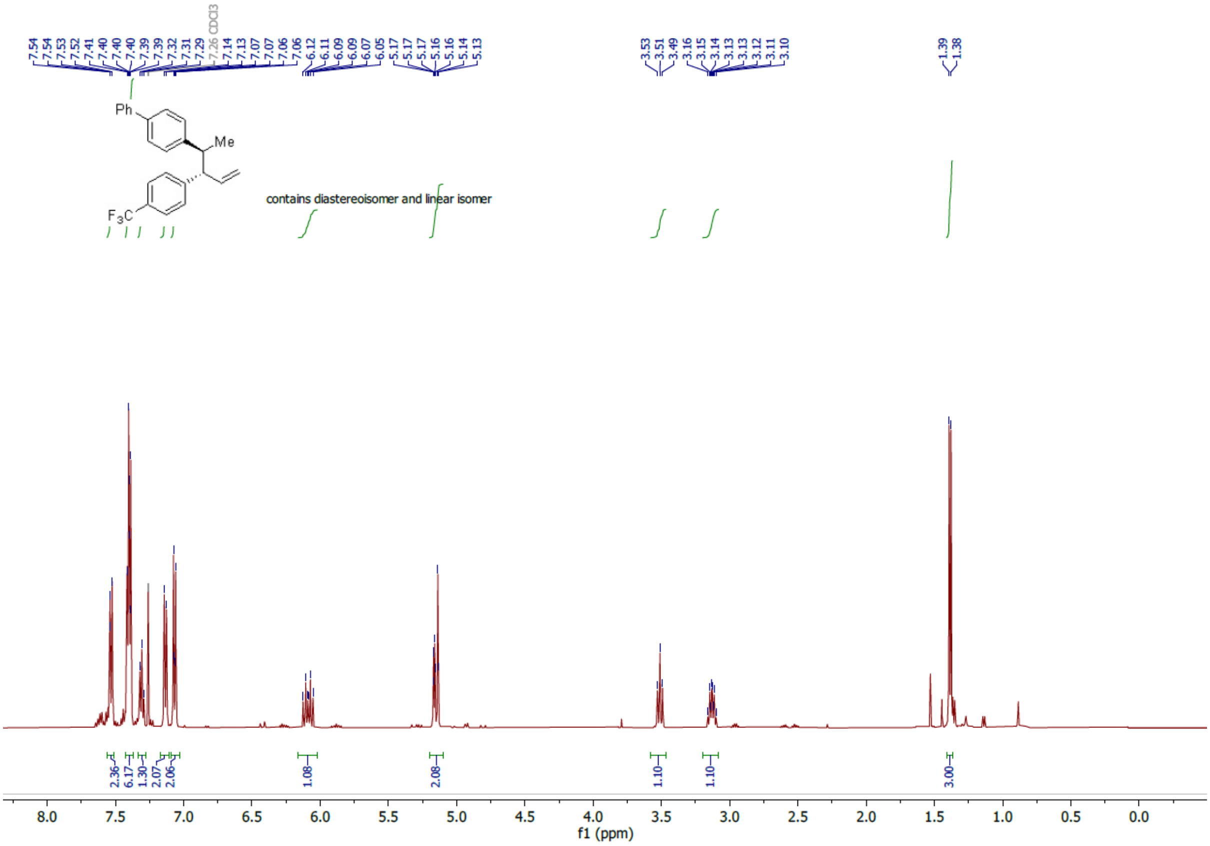

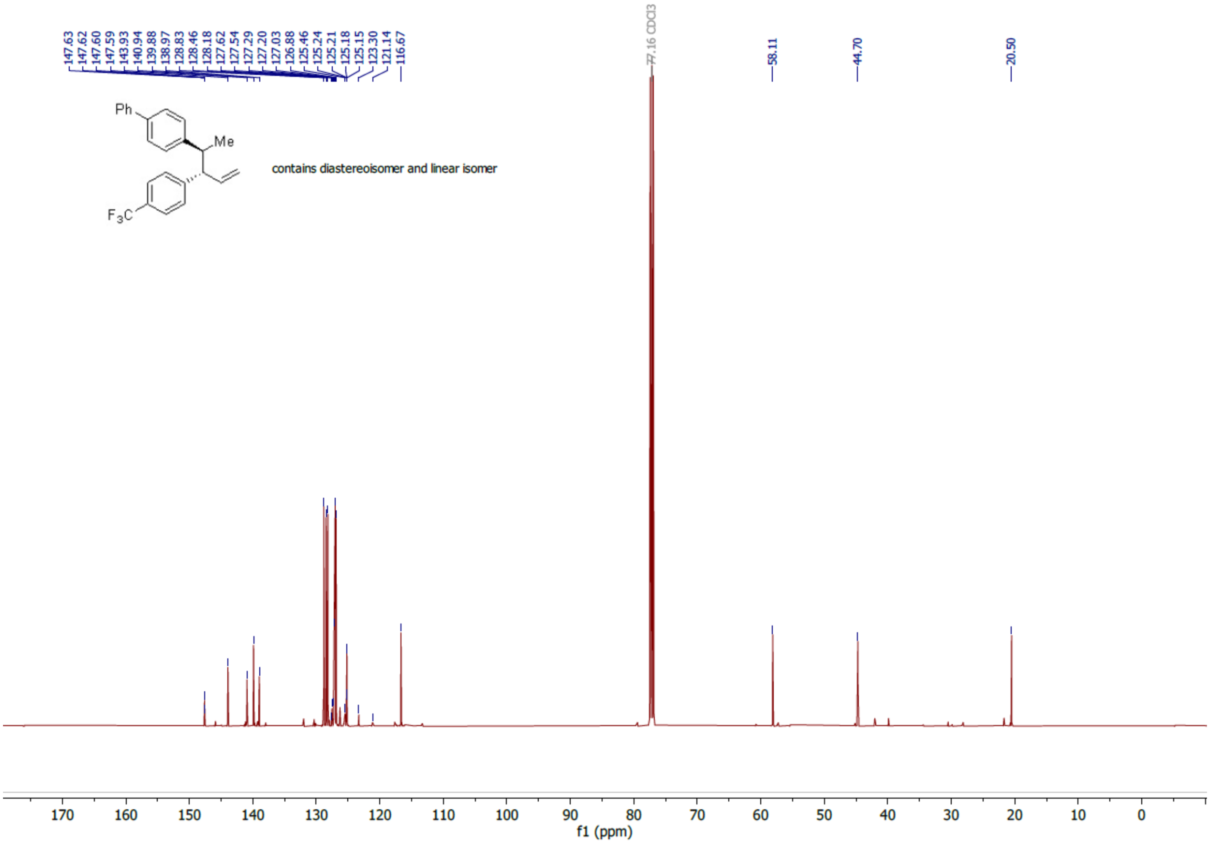

Compound **11'**

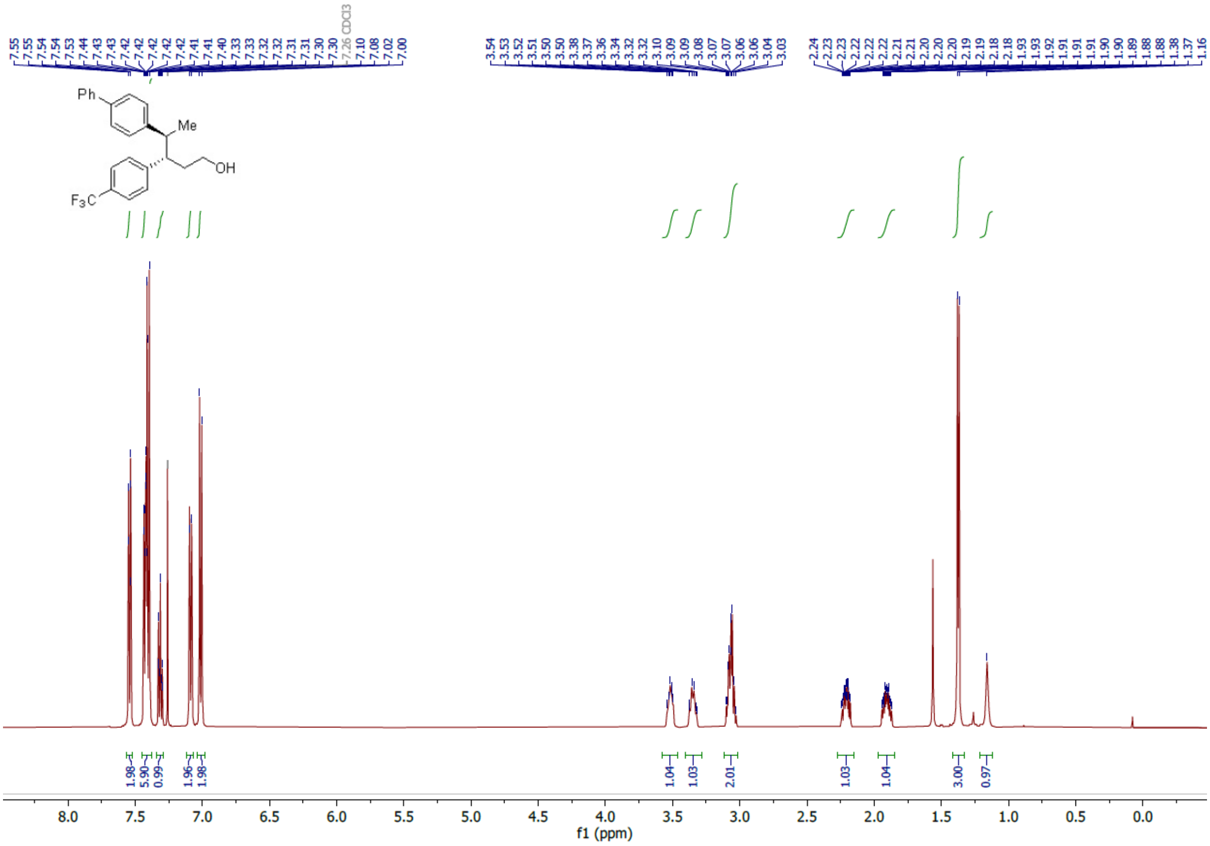

7.55
7.55
7.54
7.54
7.53
7.44
7.43
7.43
7.42
7.42
7.42
7.42
7.42
7.41
7.41
7.40
7.33
7.33
7.32
7.32
7.31
7.31
7.30
7.26 CDCl3
7.10
7.08
7.02
7.00
3.54
3.53
3.52
3.51
3.50
3.50
3.38
3.37
3.36
3.34
3.32
3.32
3.10
3.09
3.09
3.08
3.07
3.07
3.06
3.06
3.04
3.03
2.24
2.23
2.23
2.22
2.22
2.22
2.21
2.21
2.20
2.20
2.20
2.19
2.19
2.18
1.93
1.93
1.92
1.91
1.91
1.91
1.90
1.89
1.88
1.88
1.38
1.37
1.16
Ph
Me
OH
F3C
1.98
5.90
0.99
1.96
1.98
1.04
1.03
2.01
1.03
1.04
3.00
0.97
8.0
7.5
7.0
6.5
6.0
5.5
5.0
4.5
4.0
3.5
3.0
2.5
2.0
1.5
1.0
0.5
0.0
f1 (ppm)

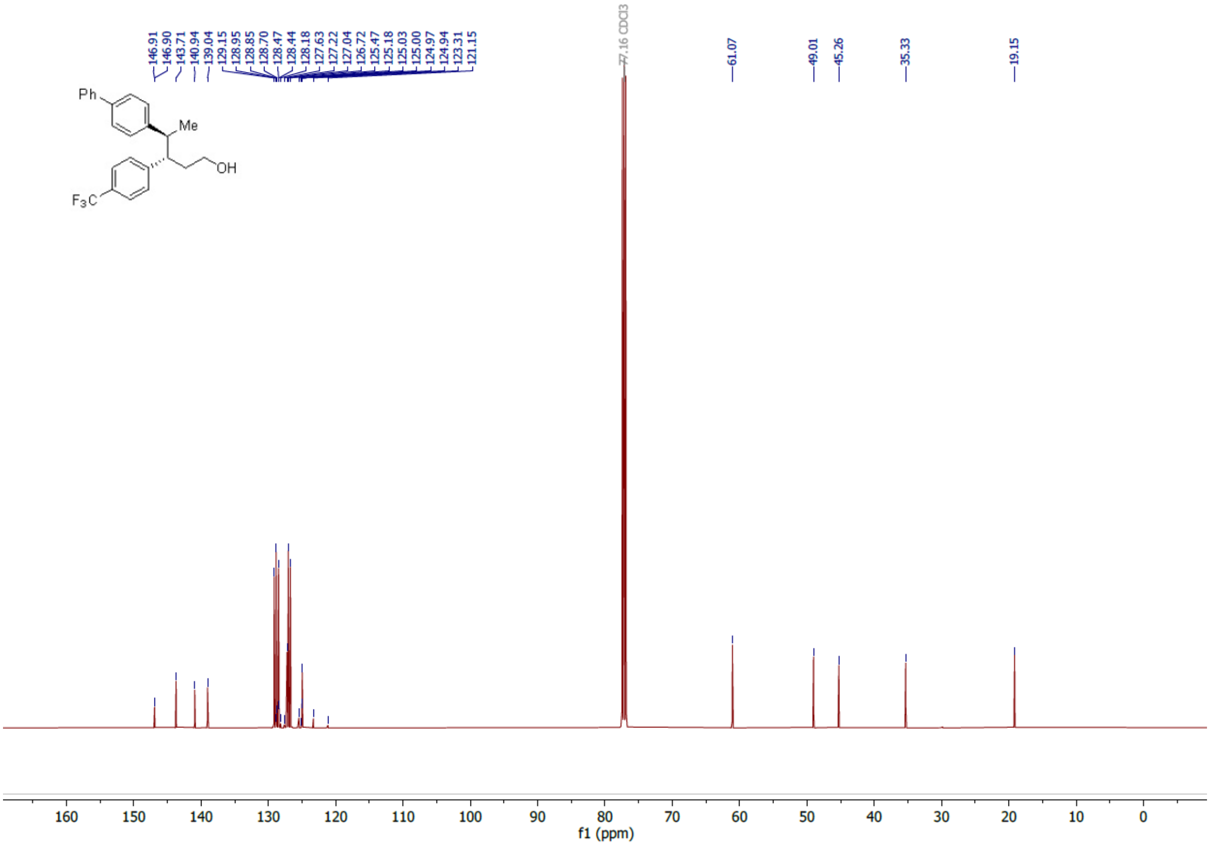

146.91
146.90
143.71
140.94
139.04
129.15
128.95
128.85
128.70
128.47
128.44
128.18
127.63
127.22
127.04
126.72
125.47
125.18
125.03
125.00
124.97
124.94
123.31
121.15
77.16 CDCl3
61.07
49.01
45.26
35.33
19.15
Ph
Me
OH
F3C
160
150
140
130
120
110
100
90
80
70
60
50
40
30
20
10
0
f1 (ppm)

Compound **12**

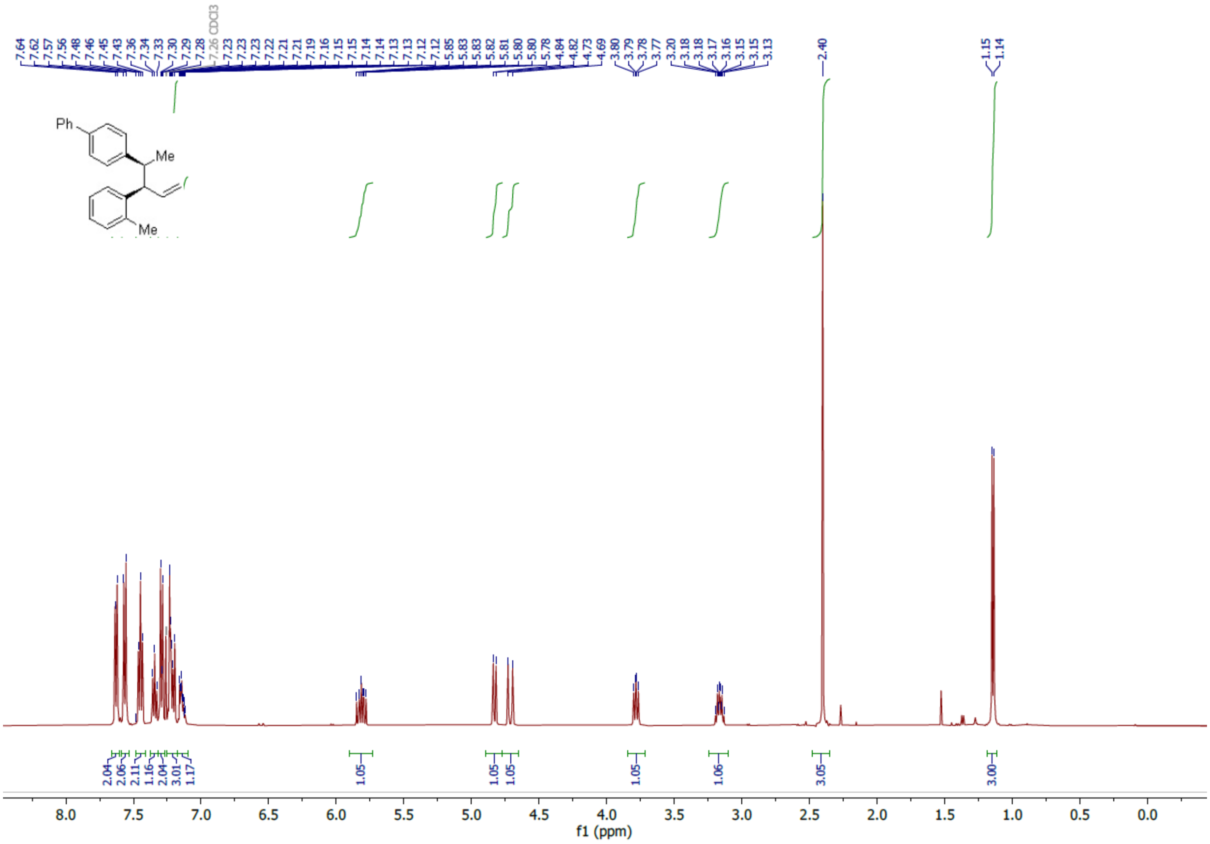

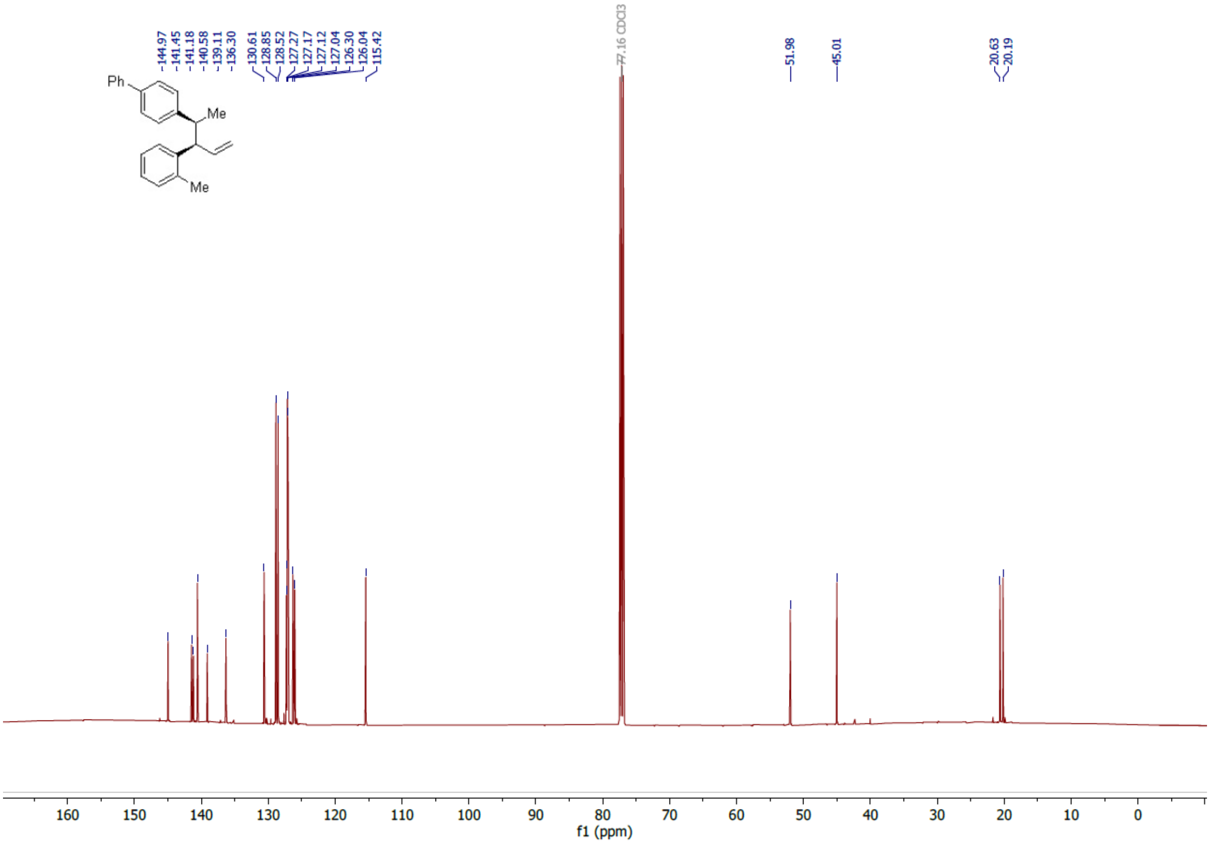

Compound **12'**

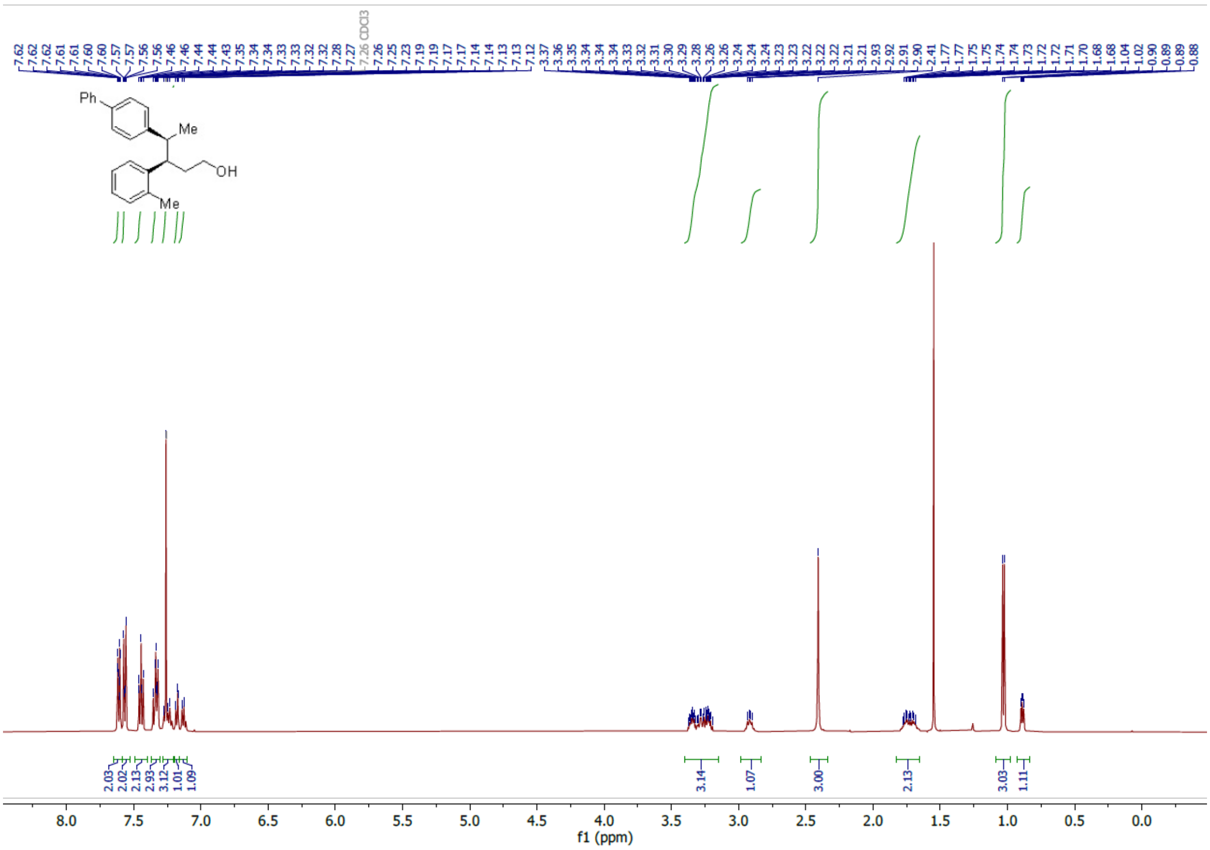

7.62 7.62 7.62 7.61 7.61 7.60 7.57 7.57 7.56 7.56 7.46 7.46 7.44 7.44 7.43 7.35 7.34 7.34 7.33 7.33 7.32 7.32 7.28 7.27 7.26 CDCl3 7.26 7.25 7.23 7.19 7.19 7.17 7.17 7.14 7.14 7.13 7.13 7.12
3.37 3.36 3.35 3.34 3.34 3.34 3.33 3.32 3.31 3.30 3.29 3.28 3.26 3.24 3.24 3.24 3.23 3.23 3.22 3.22 3.22 3.21 3.21 2.93 2.92 2.91 2.90 2.41 1.77 1.77 1.75 1.75 1.74 1.74 1.73 1.72 1.72 1.71 1.70 1.68 1.04 1.02 0.90 0.89 0.89 0.88
Ph
Me
OH
Me
2.03 2.02 2.13 2.93 3.12 1.01 1.09 3.14 1.07 3.00 2.13 3.03 1.11
8.0 7.5 7.0 6.5 6.0 5.5 5.0 4.5 4.0 3.5 3.0 2.5 2.0 1.5 1.0 0.5 0.0
f1 (ppm)

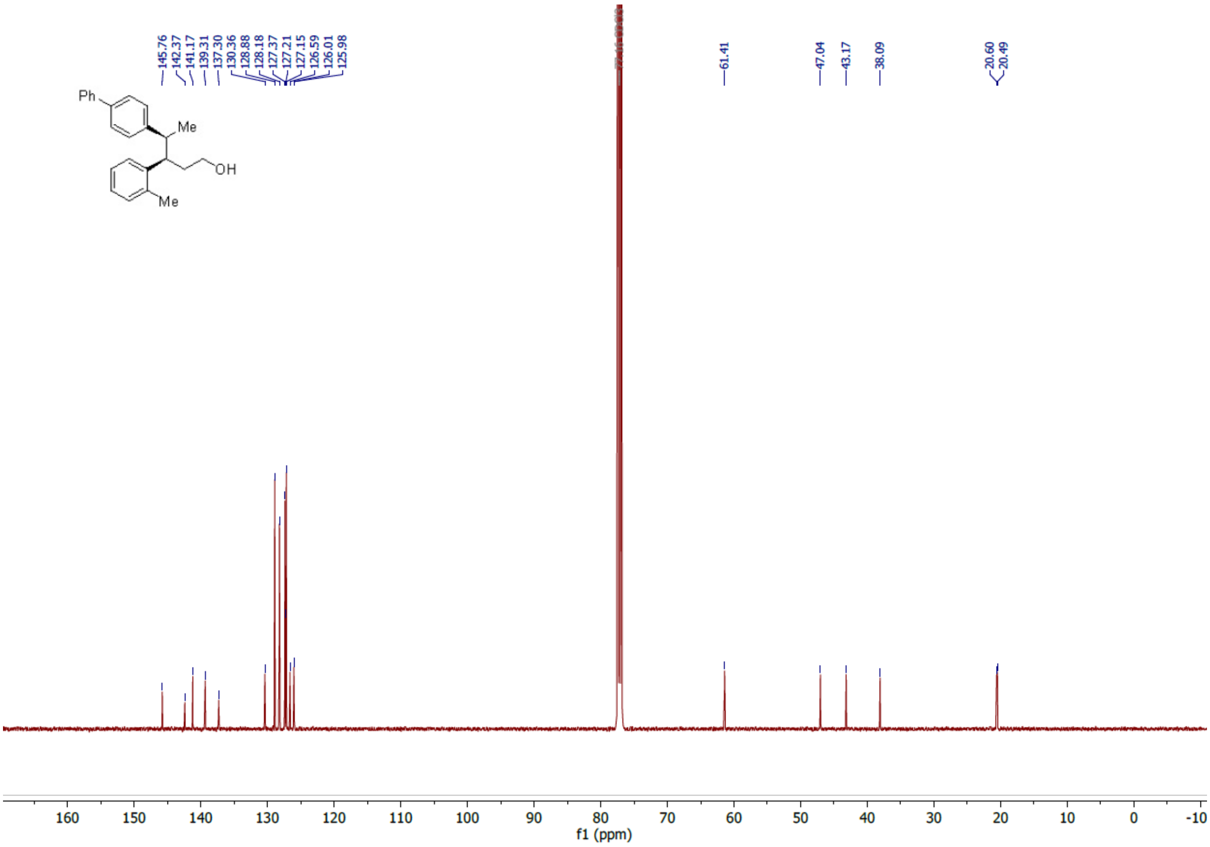

145.76 142.37 141.17 139.31 137.30 130.36 128.88 128.18 127.37 127.21 127.15 126.59 126.01 125.98
61.41
47.04
43.17
38.09
20.60 20.49
Ph
Me
OH
Me
160 150 140 130 120 110 100 90 80 70 60 50 40 30 20 10 0 -10
f1 (ppm)

Compound **13**

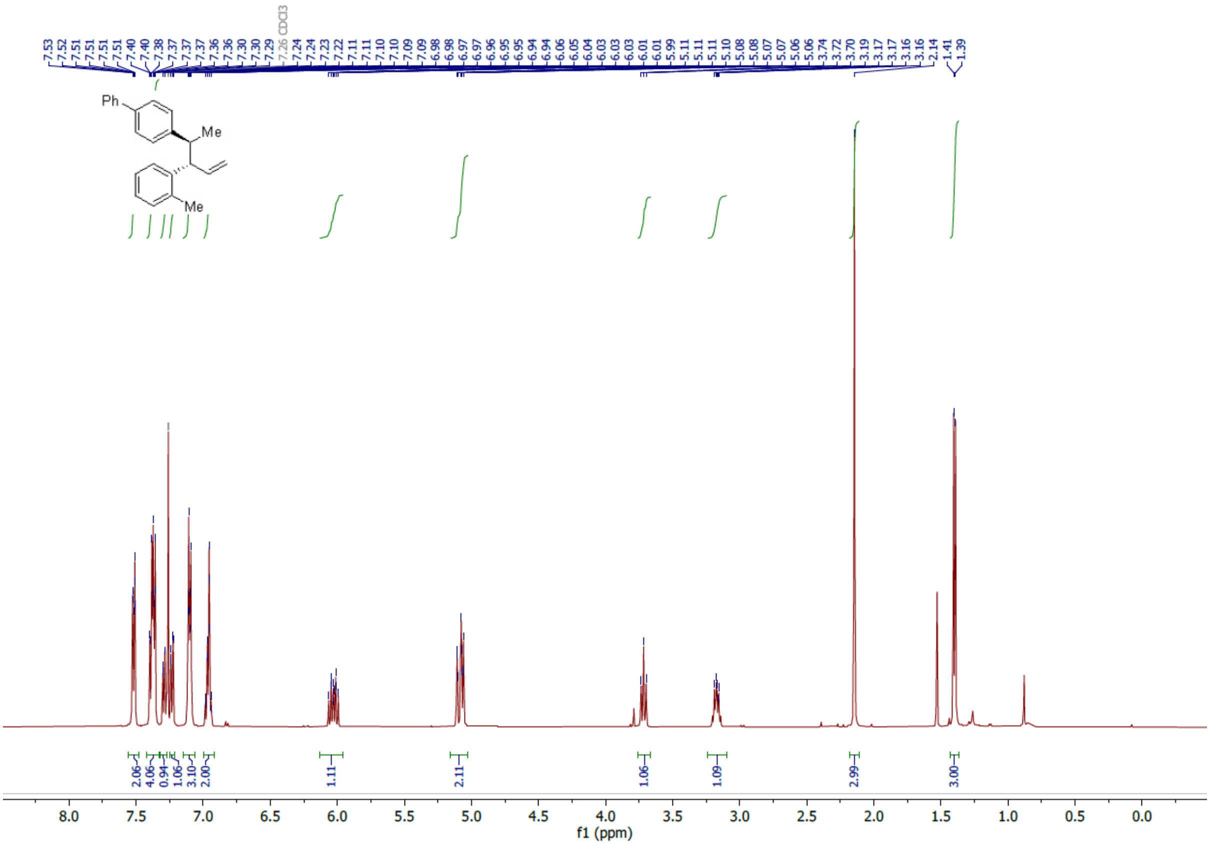

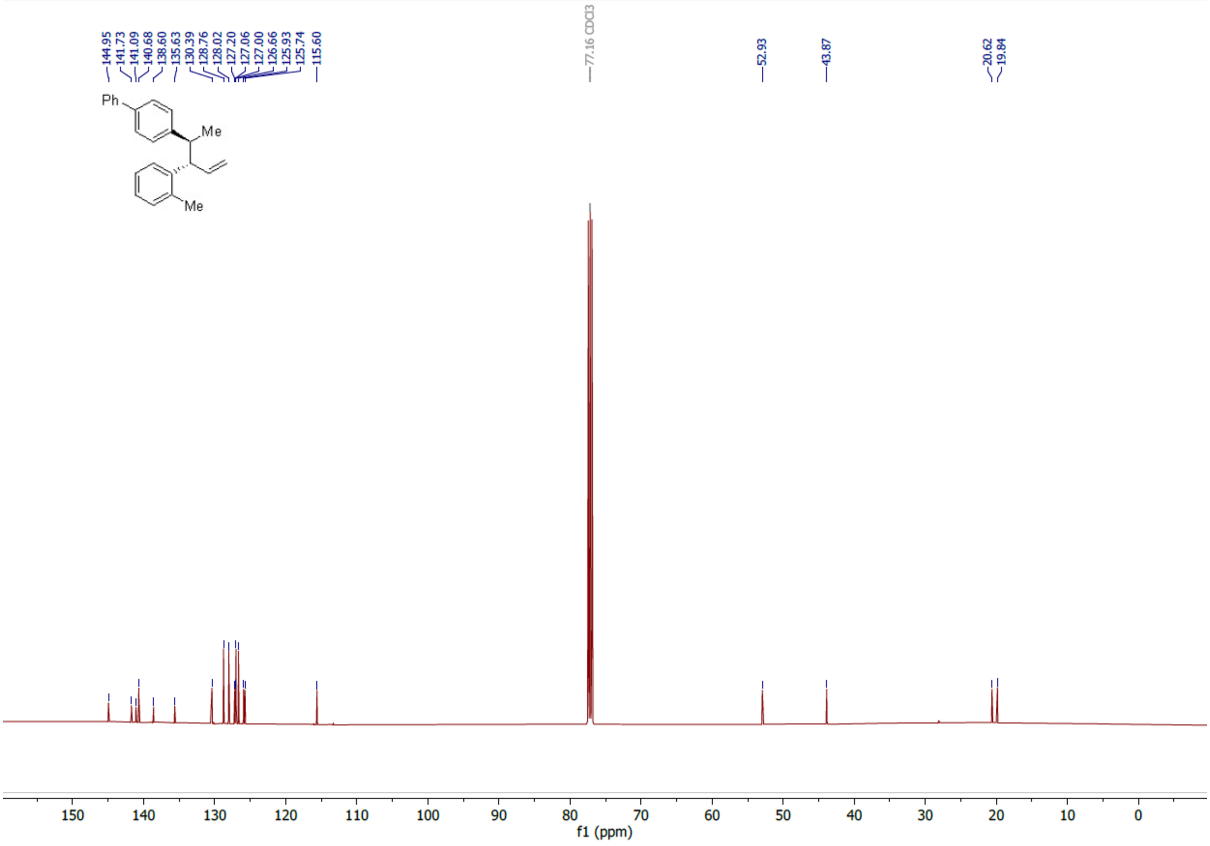

Compound **13'**

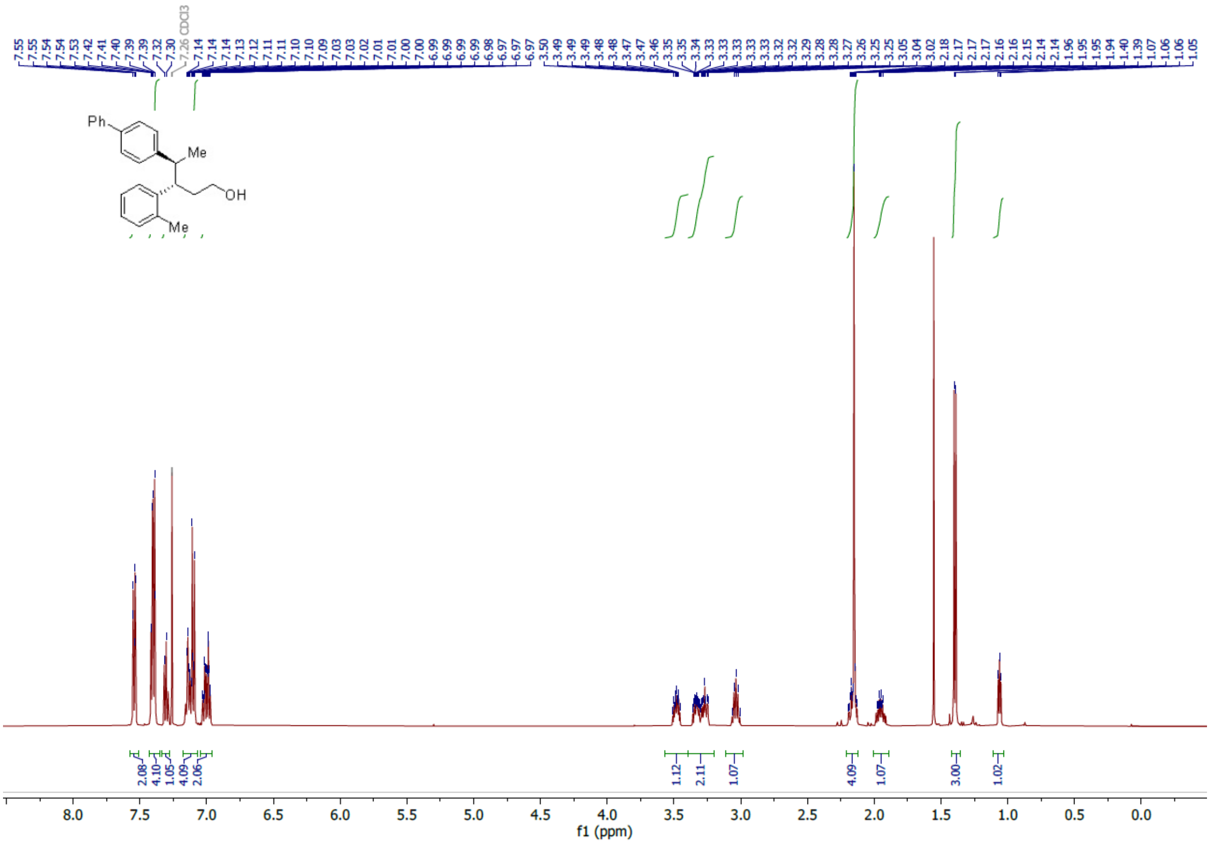

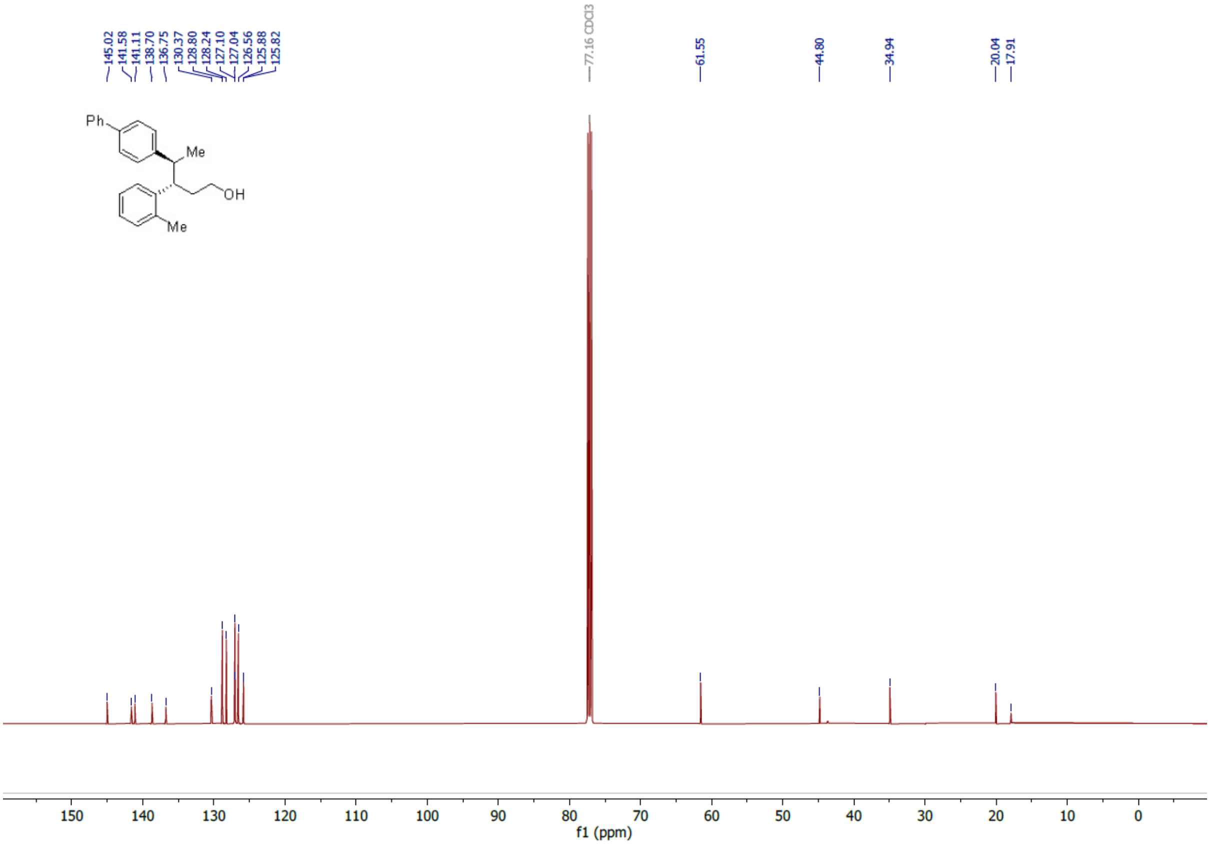

Compound **14**

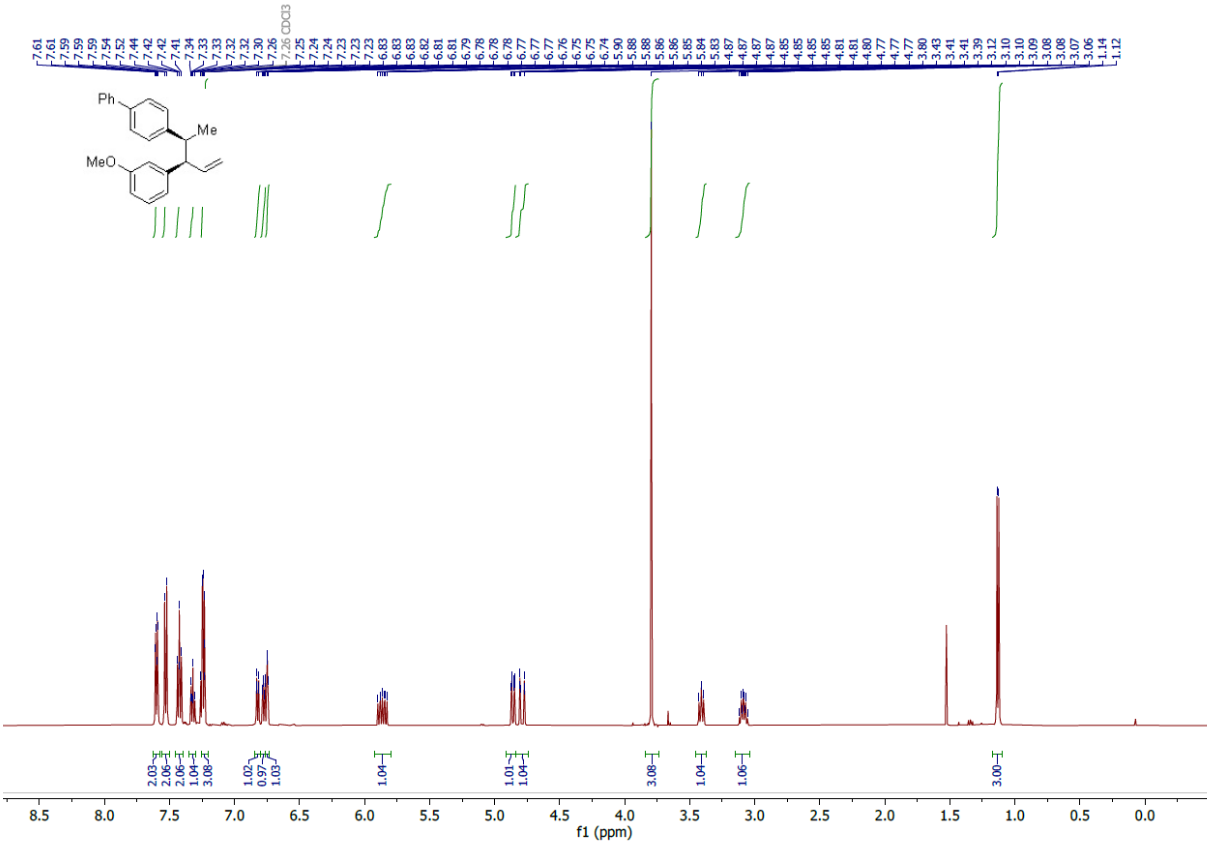
Ph
Me
MeO
CDCl3
7.61 7.61 7.59 7.59 7.59 7.54 7.52 7.44 7.42 7.42 7.41 7.34 7.33 7.33 7.32 7.32 7.30 7.26 7.26 7.25 7.24 7.24 7.23 7.23 7.23 6.83 6.83 6.83 6.82 6.81 6.81 6.79 6.78 6.78 6.78 6.77 6.77 6.77 6.76 6.75 6.75 6.74 5.90 5.88 5.88 5.86 5.86 5.85 5.84 5.83 4.87 4.87 4.87 4.85 4.85 4.85 4.85 4.81 4.81 4.80 4.77 4.77 4.77 3.80 3.43 3.41 3.41 3.39 3.12 3.10 3.10 3.09 3.08 3.08 3.07 3.06 1.14 1.12
2.03 2.06 2.06 1.04 3.08 1.02 0.97 1.03 1.04 1.01 1.04 3.08 1.04 1.06 3.00
8.5 8.0 7.5 7.0 6.5 6.0 5.5 5.0 4.5 4.0 3.5 3.0 2.5 2.0 1.5 1.0 0.5 0.0
f1 (ppm)

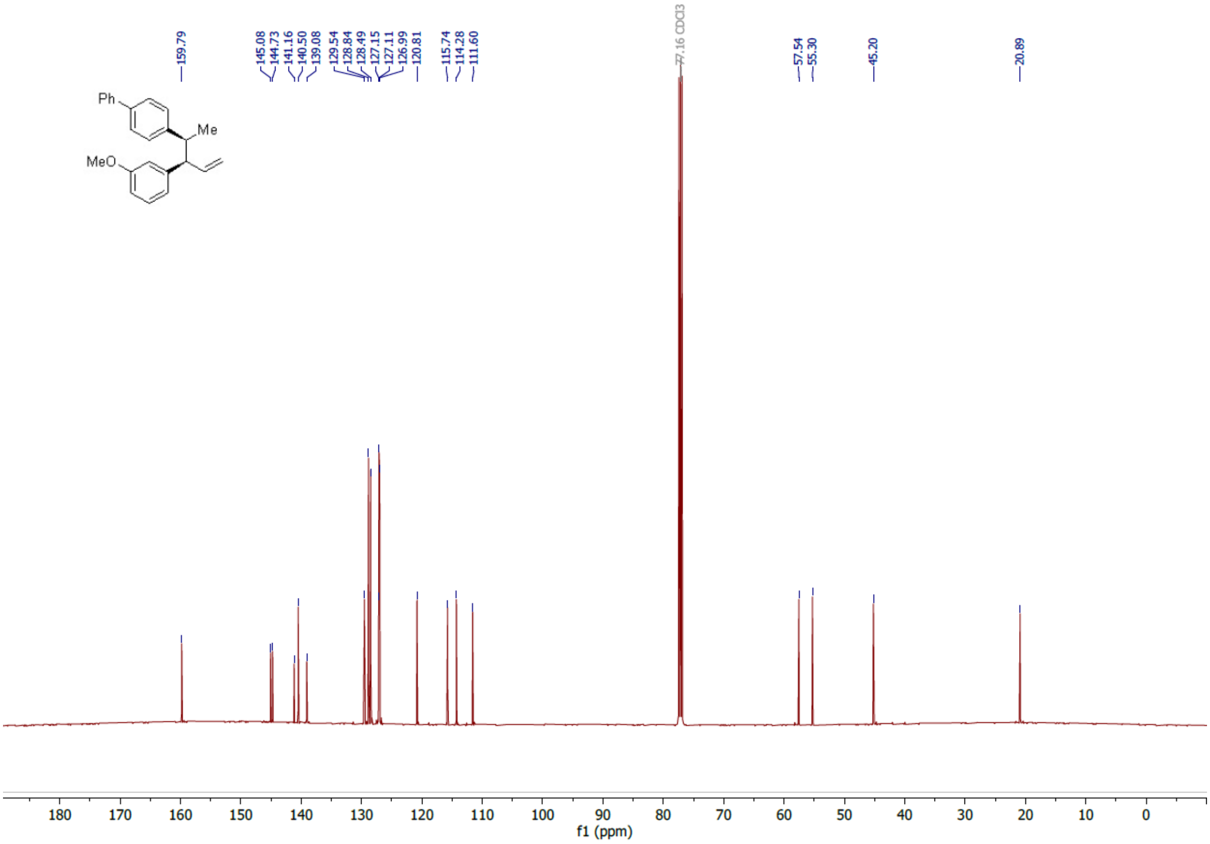
Ph
Me
MeO
159.79
145.08 144.73 141.16 140.50 139.08
129.54 128.84 128.49 127.15 127.11 126.99 120.81
115.74 114.28 111.60
77.16 CDCl3
57.54 55.30
45.20
20.89
180 170 160 150 140 130 120 110 100 90 80 70 60 50 40 30 20 10 0
f1 (ppm)

Compound **14'**

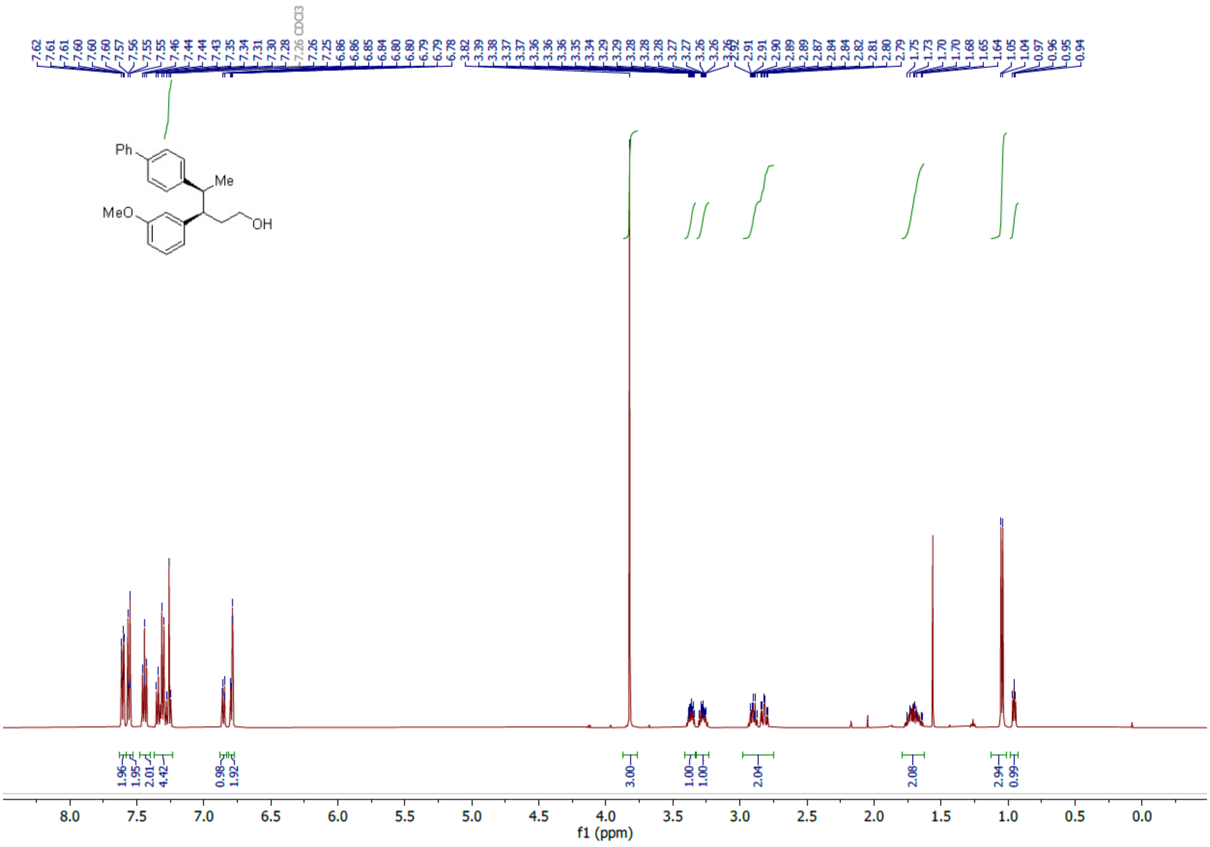

Ph
Me
MeO
OH
f1 (ppm)

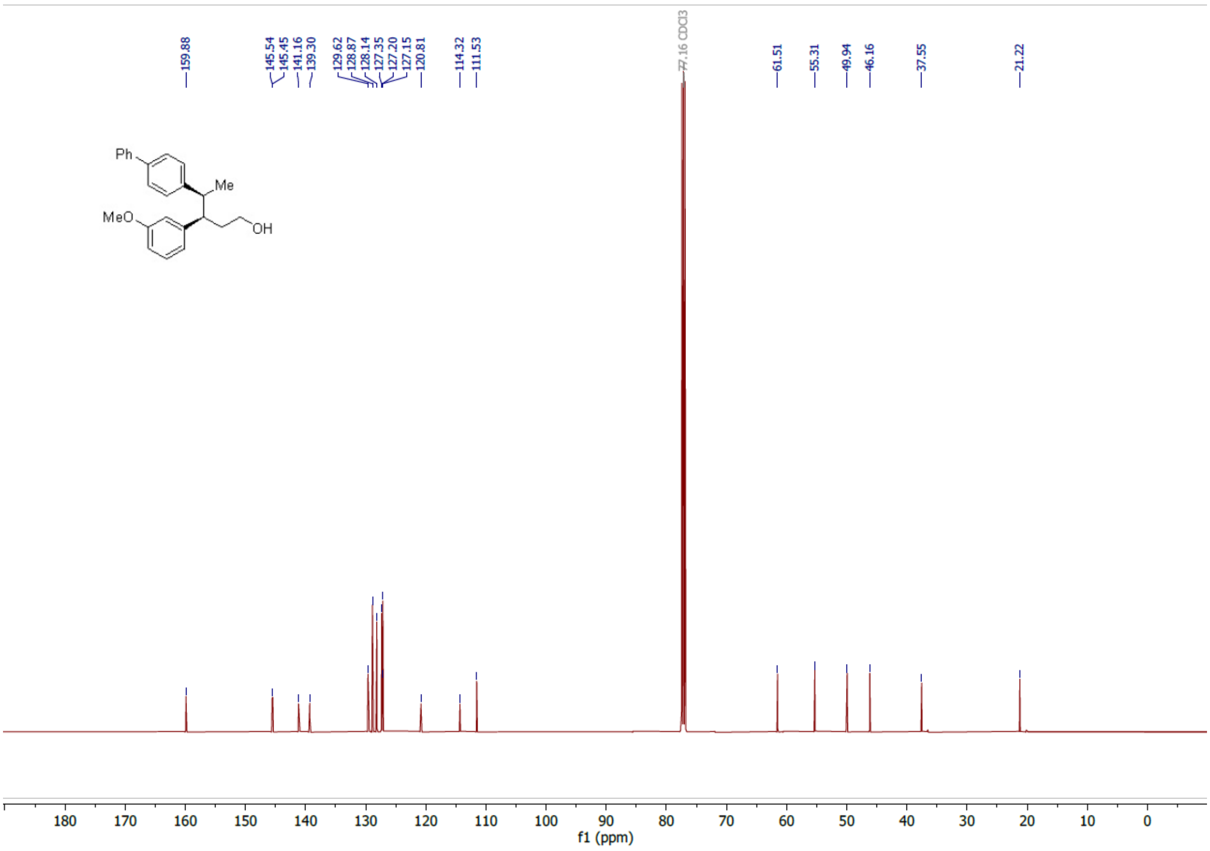

159.88
145.54
145.45
141.16
139.30
129.62
128.87
128.14
127.35
127.20
127.15
120.81
114.32
111.53
77.16 CDCl3
61.51
55.31
49.94
46.16
37.55
21.22
Ph
Me
MeO
OH
f1 (ppm)

Compound **15**

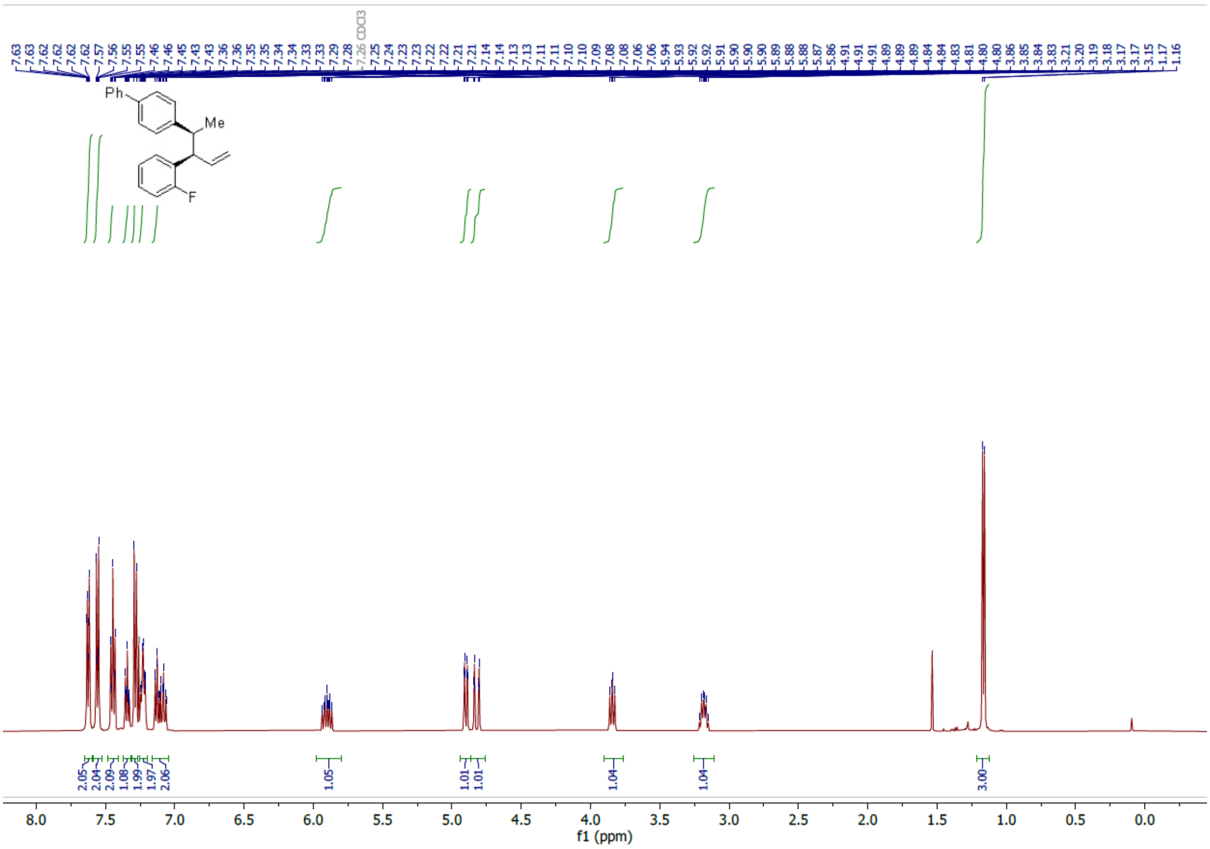

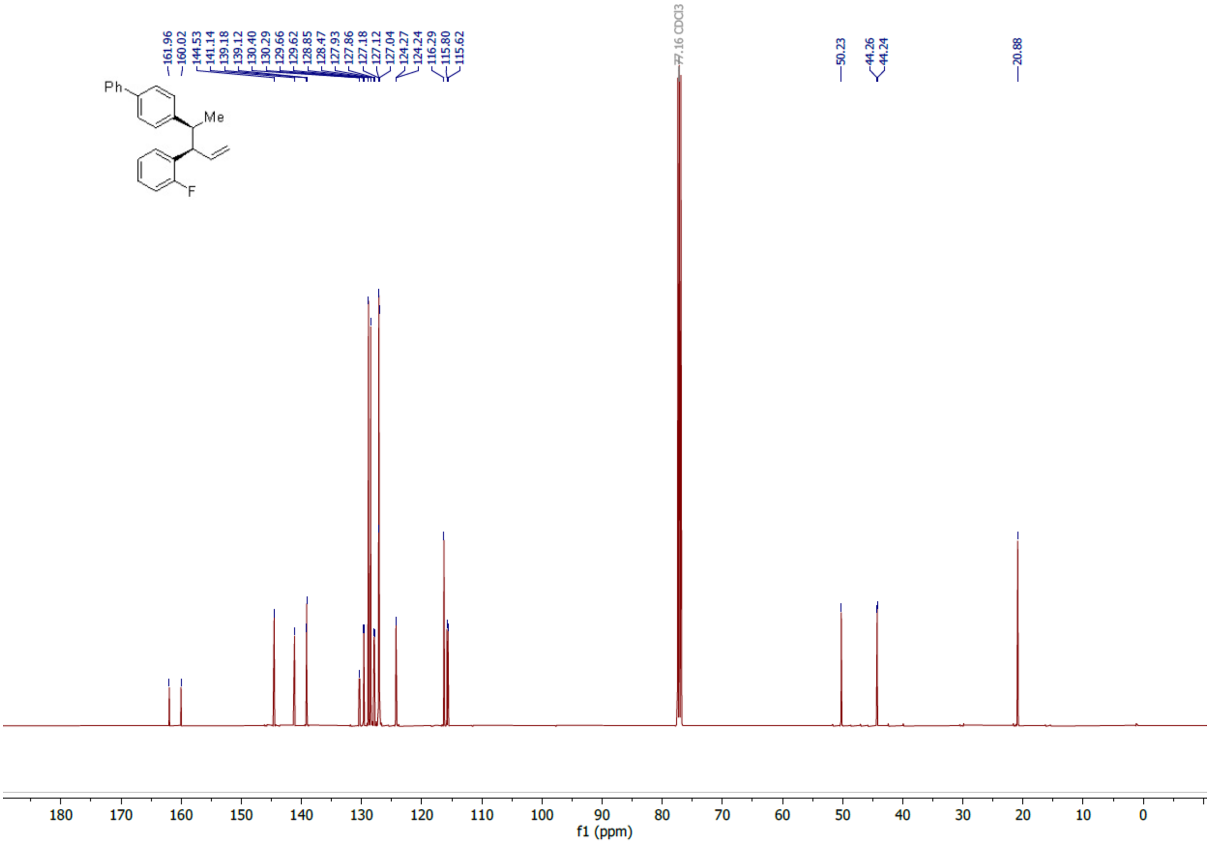

Compound **15'**

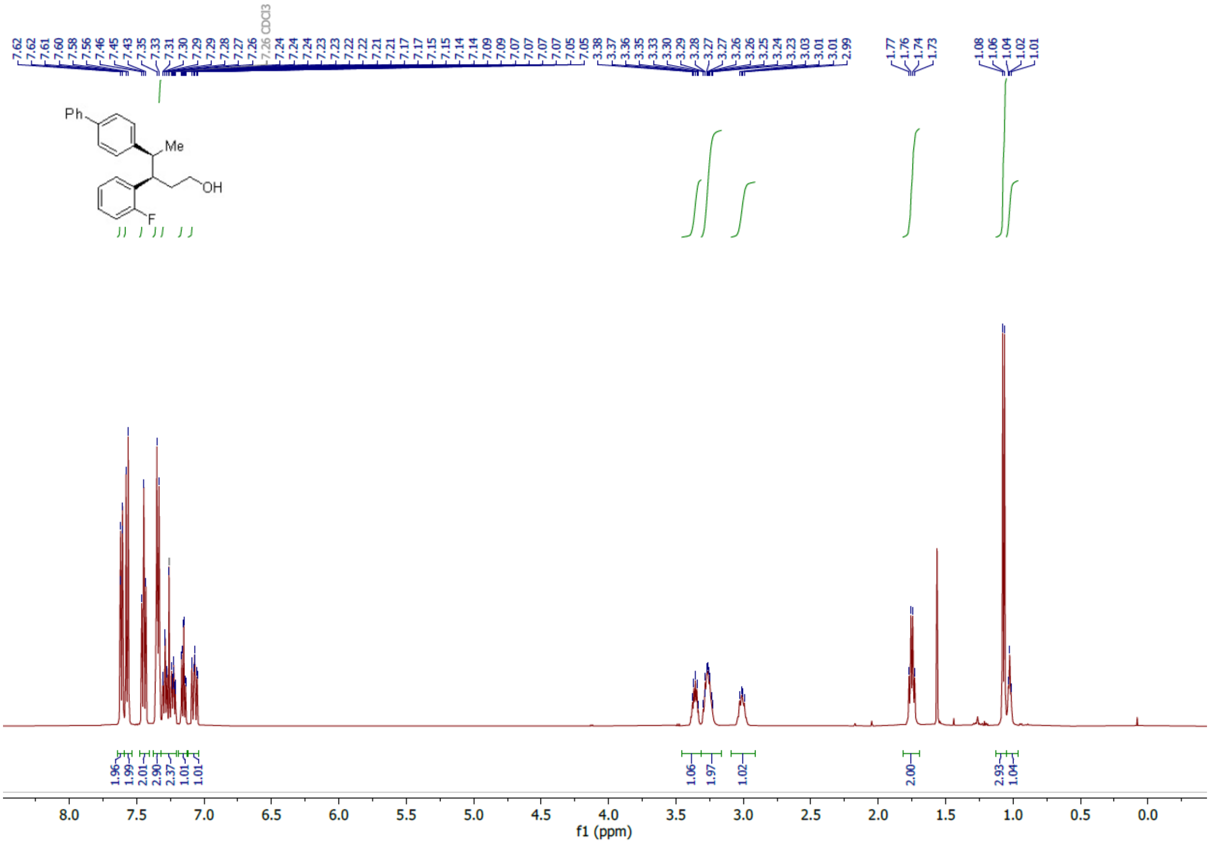
7.62 7.62 7.61 7.60 7.58 7.56 7.46 7.45 7.43 7.35 7.33 7.31 7.30 7.29 7.29 7.28 7.27 7.26 CDCl3 7.24 7.24 7.24 7.23 7.23 7.22 7.22 7.21 7.21 7.17 7.17 7.15 7.15 7.14 7.14 7.09 7.09 7.07 7.07 7.07 7.07 7.05 7.05 3.38 3.37 3.36 3.35 3.33 3.30 3.29 3.28 3.27 3.27 3.26 3.26 3.25 3.24 3.23 3.03 3.01 3.01 2.99 1.77 1.76 1.74 1.73 1.08 1.06 1.04 1.02 1.01
Ph
Me
OH
F
1.96 1.99 2.01 2.90 2.37 1.01 1.01 1.06 1.97 1.02 2.00 2.93 1.04
f1 (ppm)

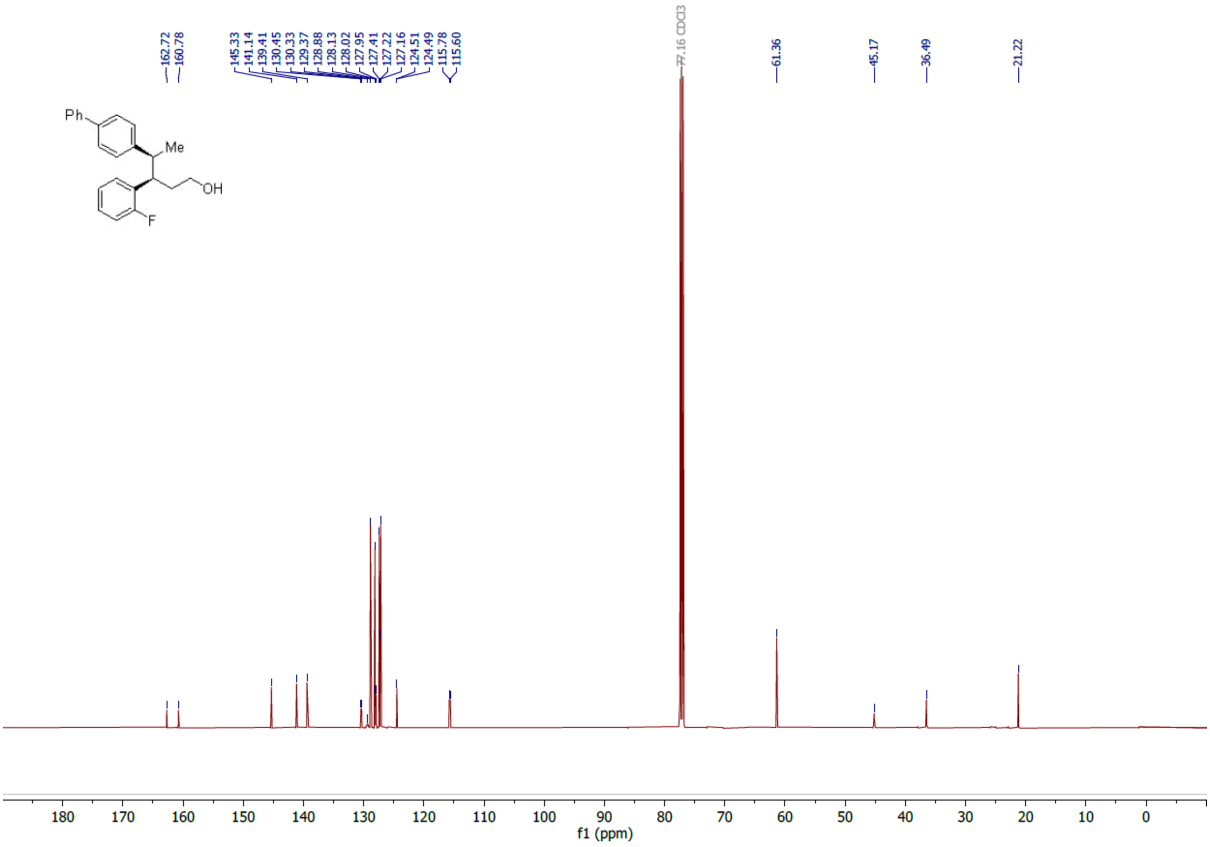
162.72 160.78 145.33 141.14 139.41 130.45 130.33 129.37 128.88 128.13 128.02 127.95 127.41 127.22 127.16 124.51 124.49 115.78 115.60 77.16 CDCl3 61.36 45.17 36.49 21.22
Ph
Me
OH
F
f1 (ppm)

Compound **16**

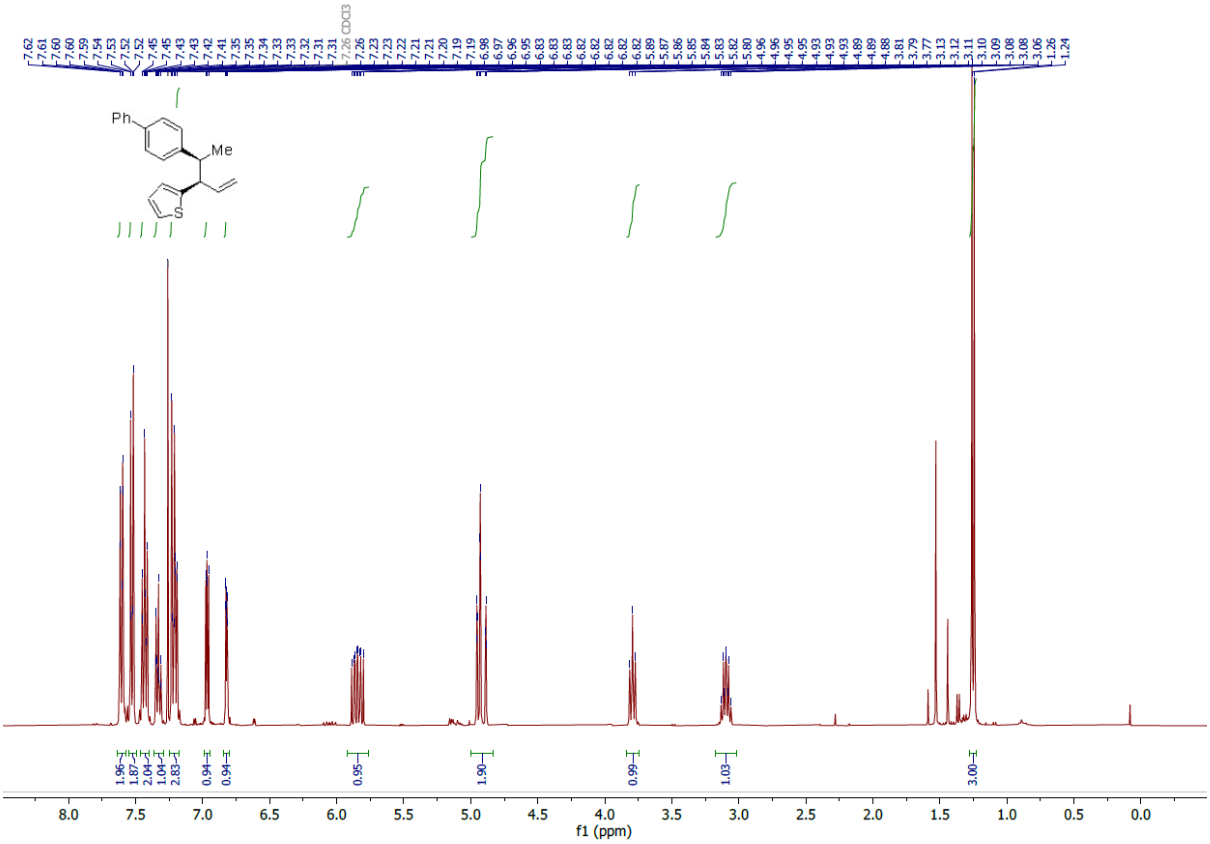

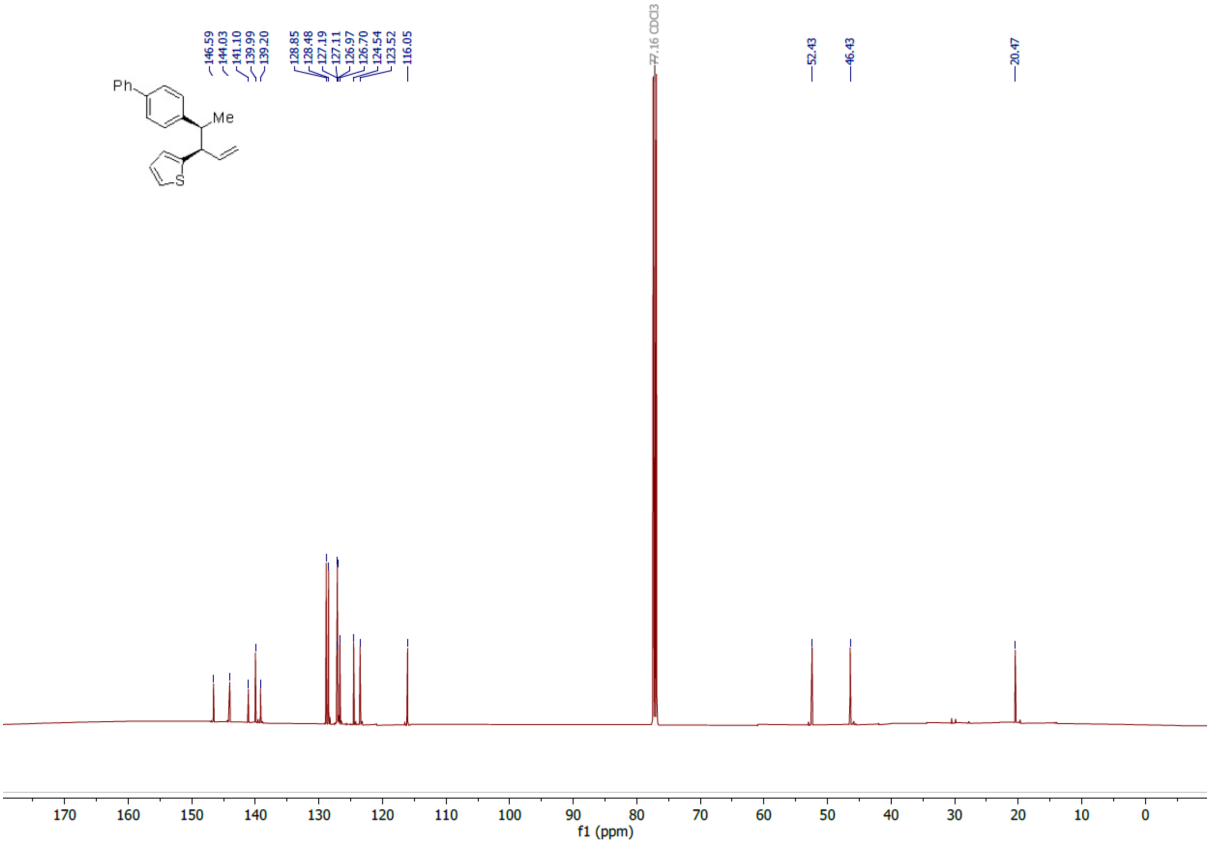

Compound **17**

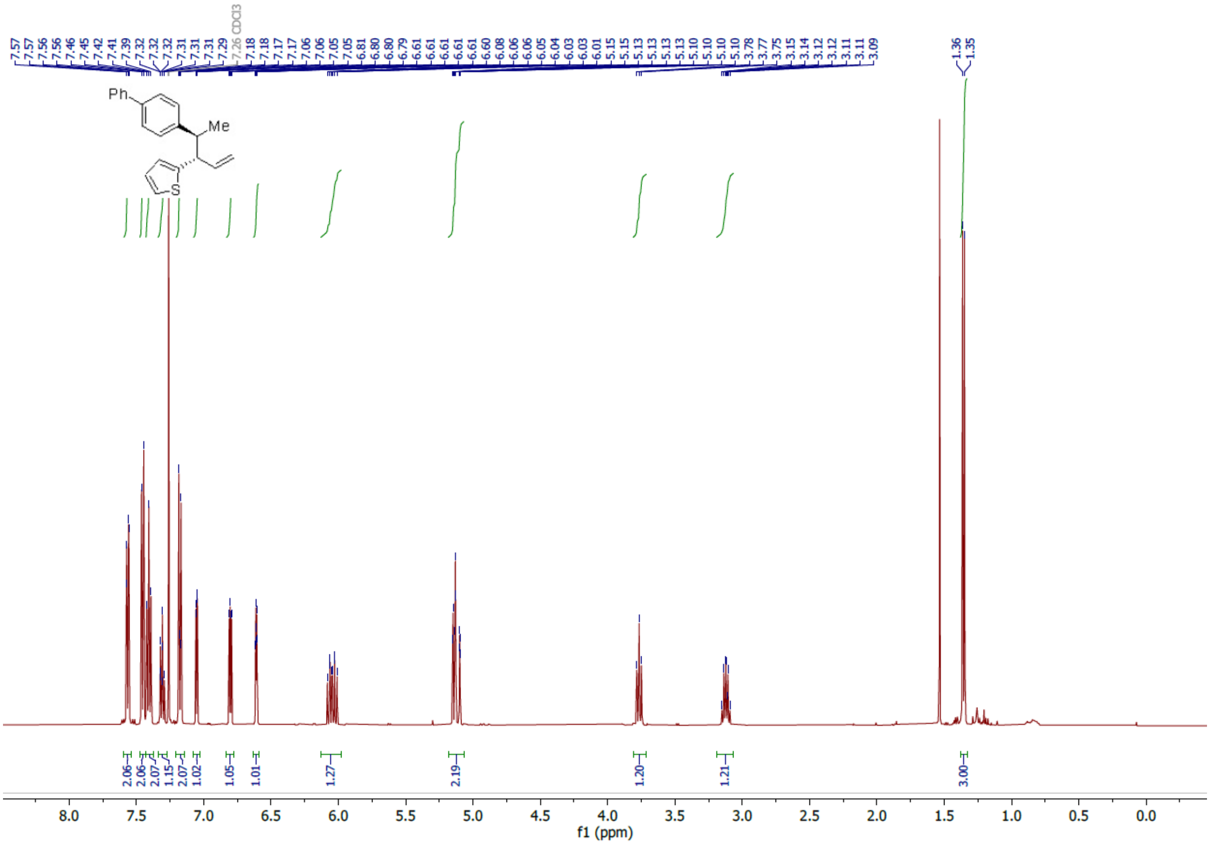

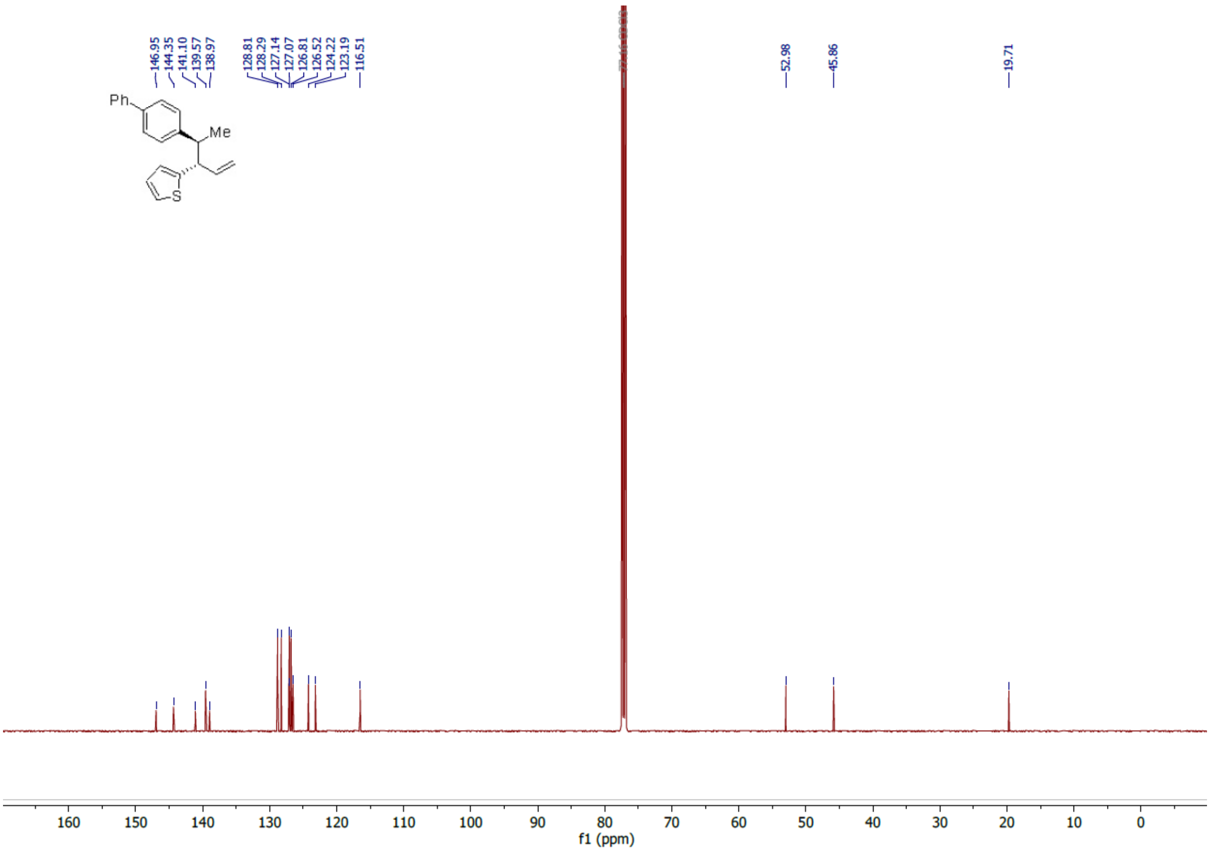

Compound **18**

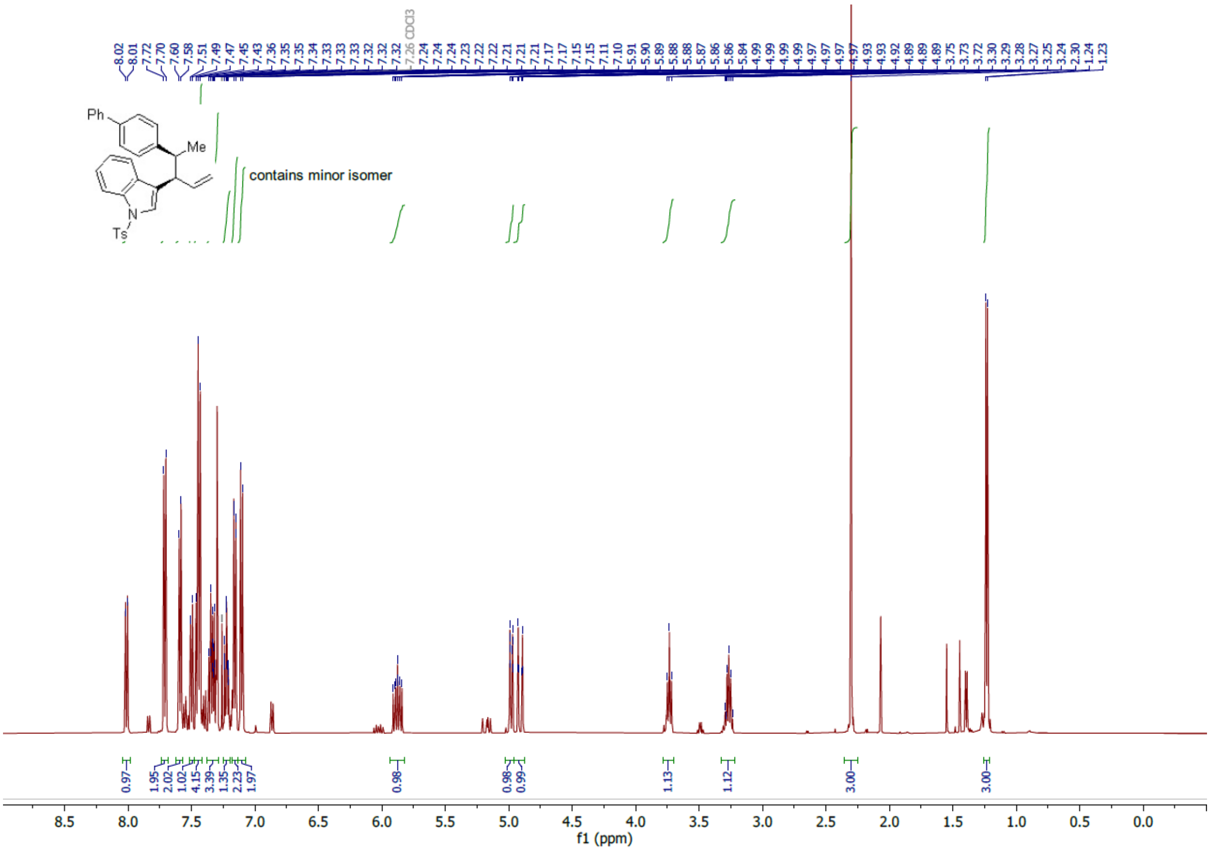
contains minor isomer
f1 (ppm)

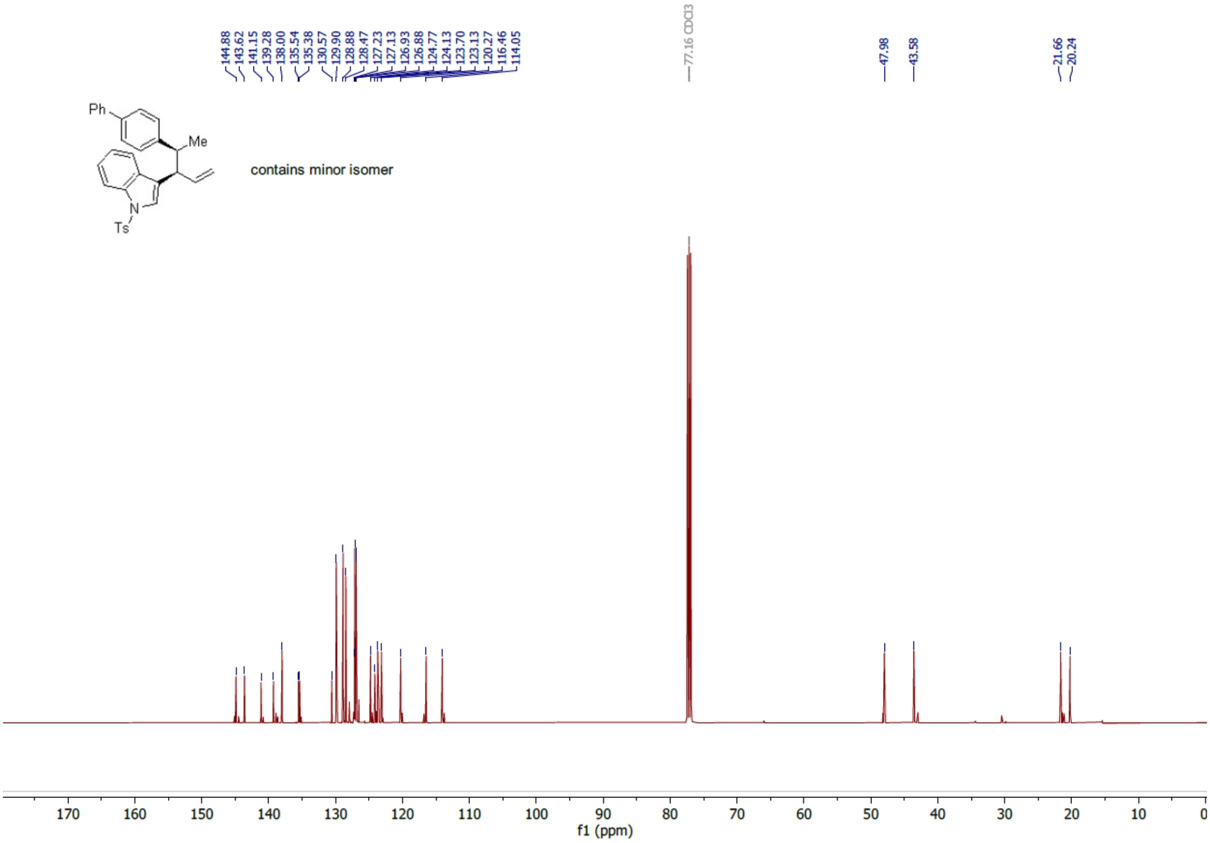
144.88
143.62
141.15
139.28
138.00
135.54
135.38
130.57
129.90
128.88
128.47
127.23
127.13
126.93
126.88
124.77
124.13
123.70
123.13
120.27
116.46
114.05
77.16 CDCl3
47.98
43.58
21.66
20.24
contains minor isomer
f1 (ppm)

Compound **18'**

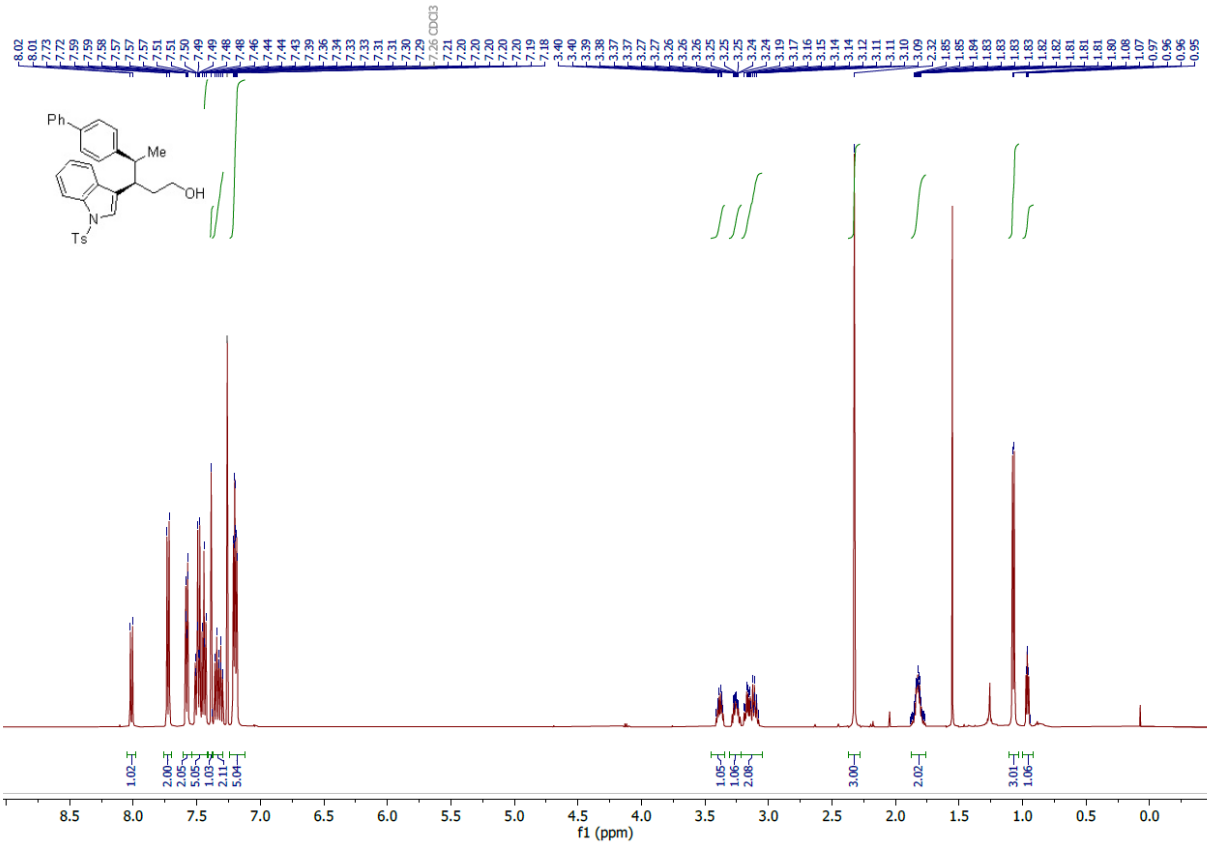

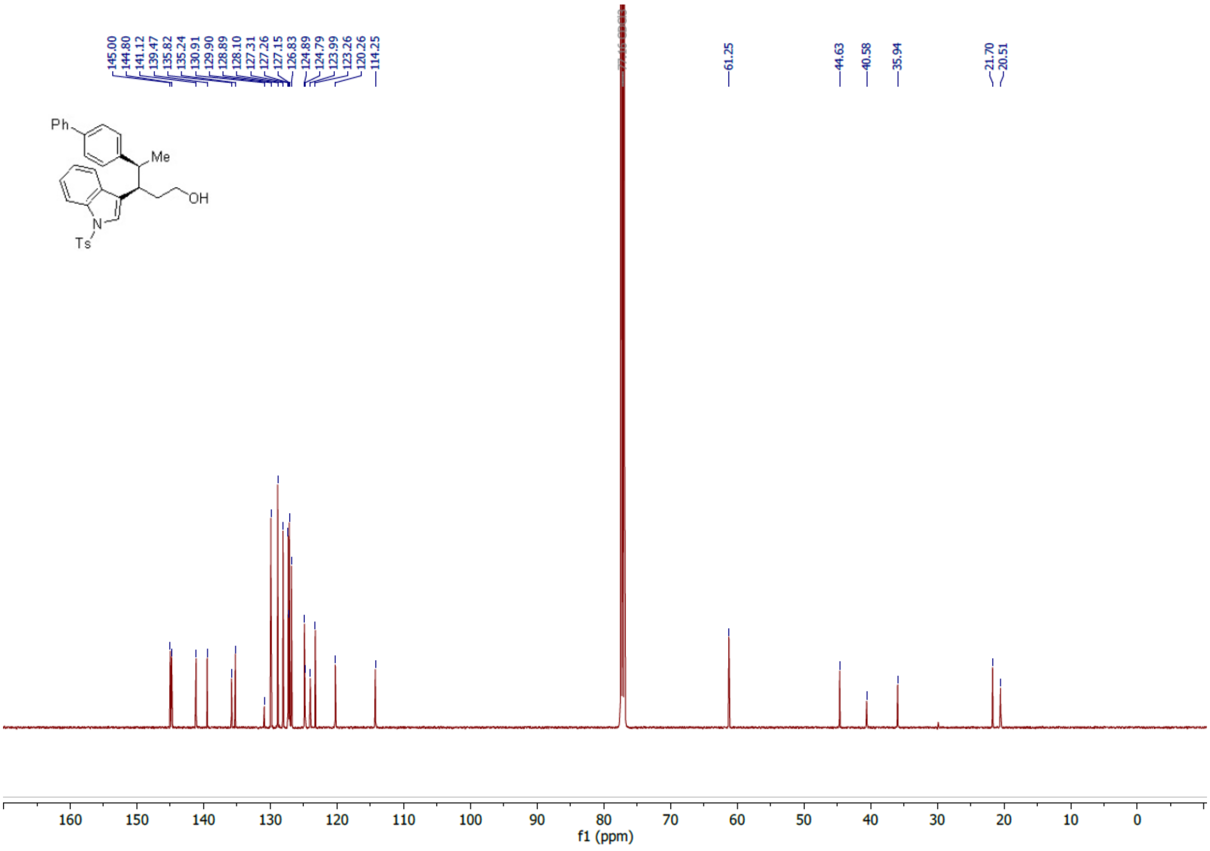

Compound **19**

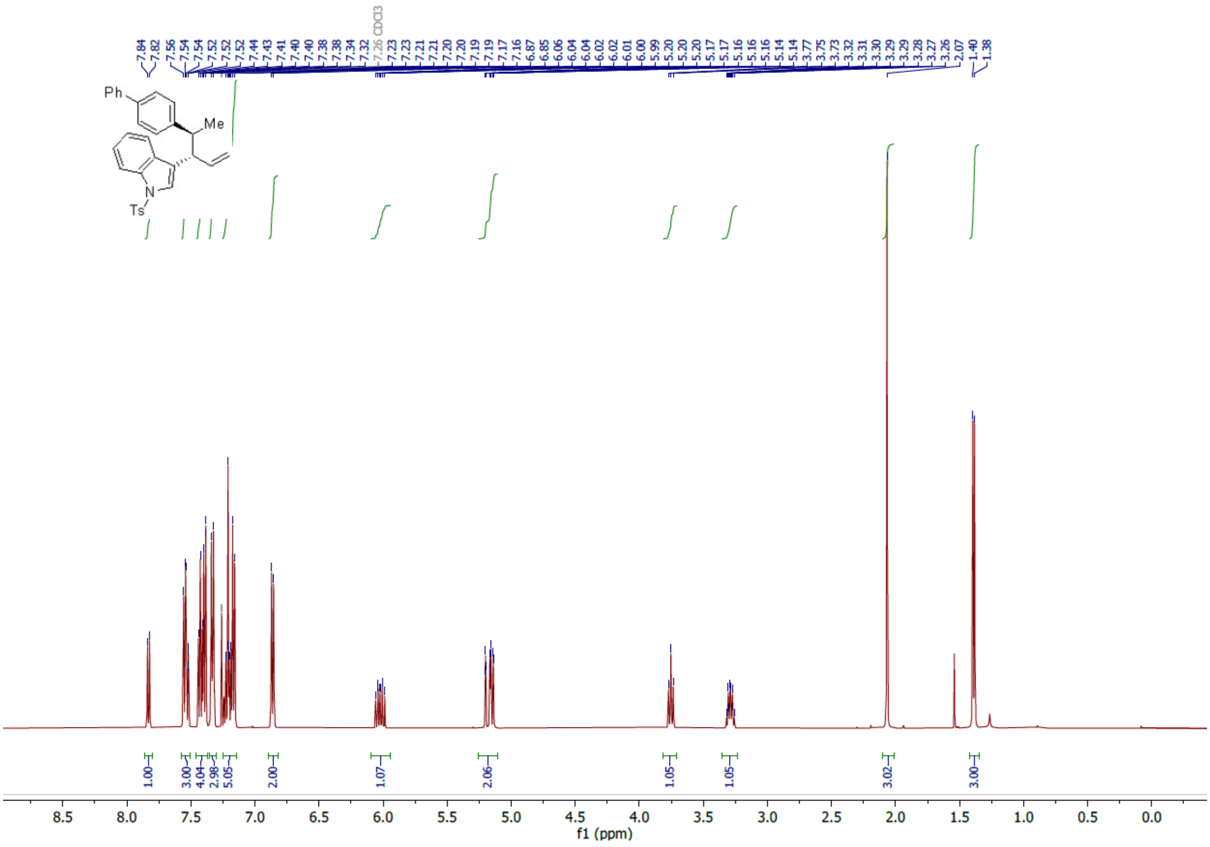

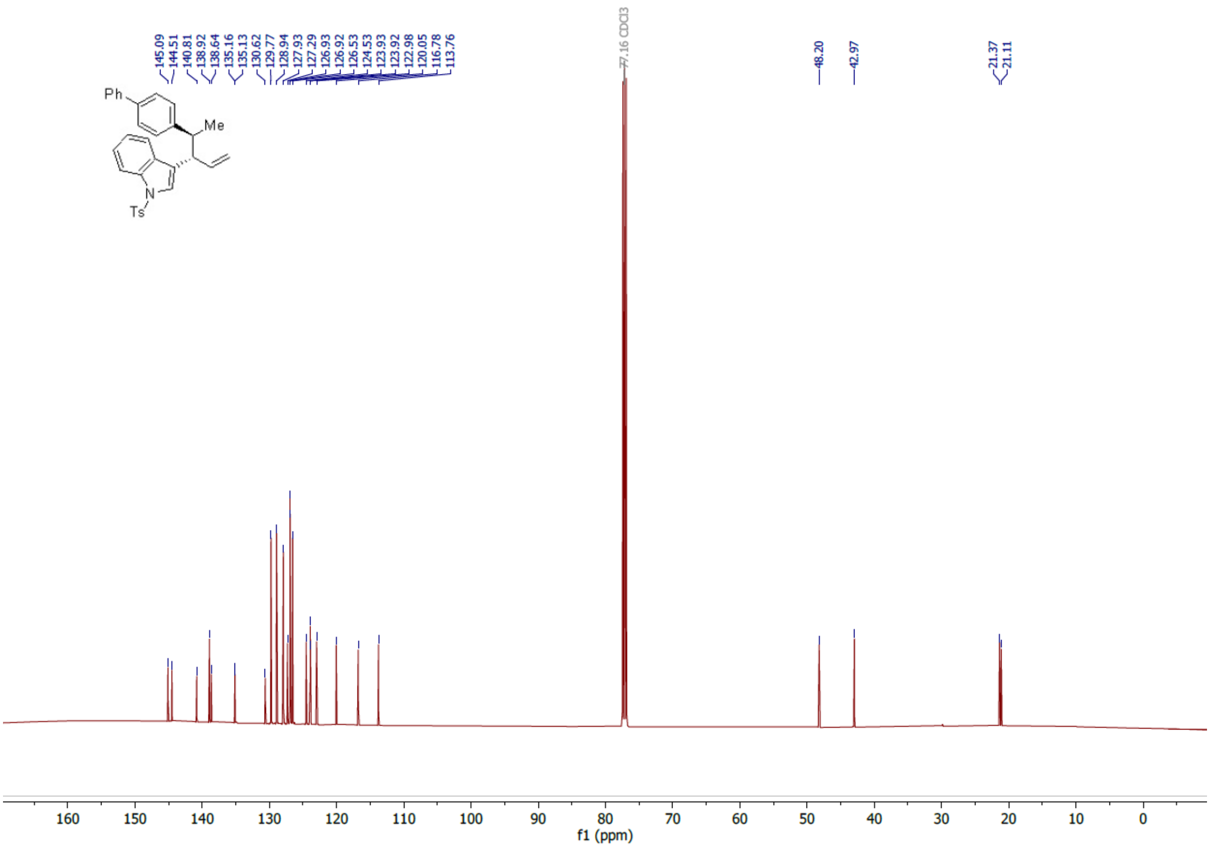

Compound **19'**

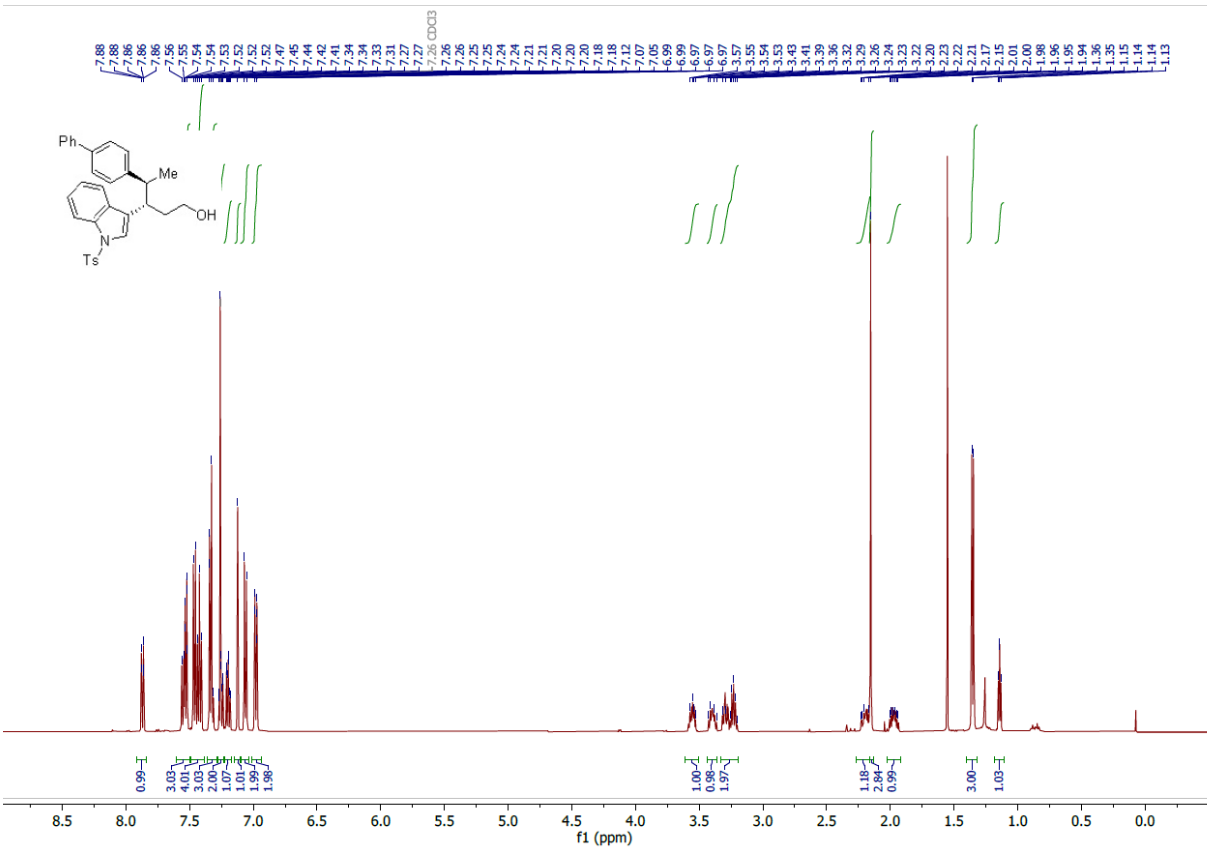

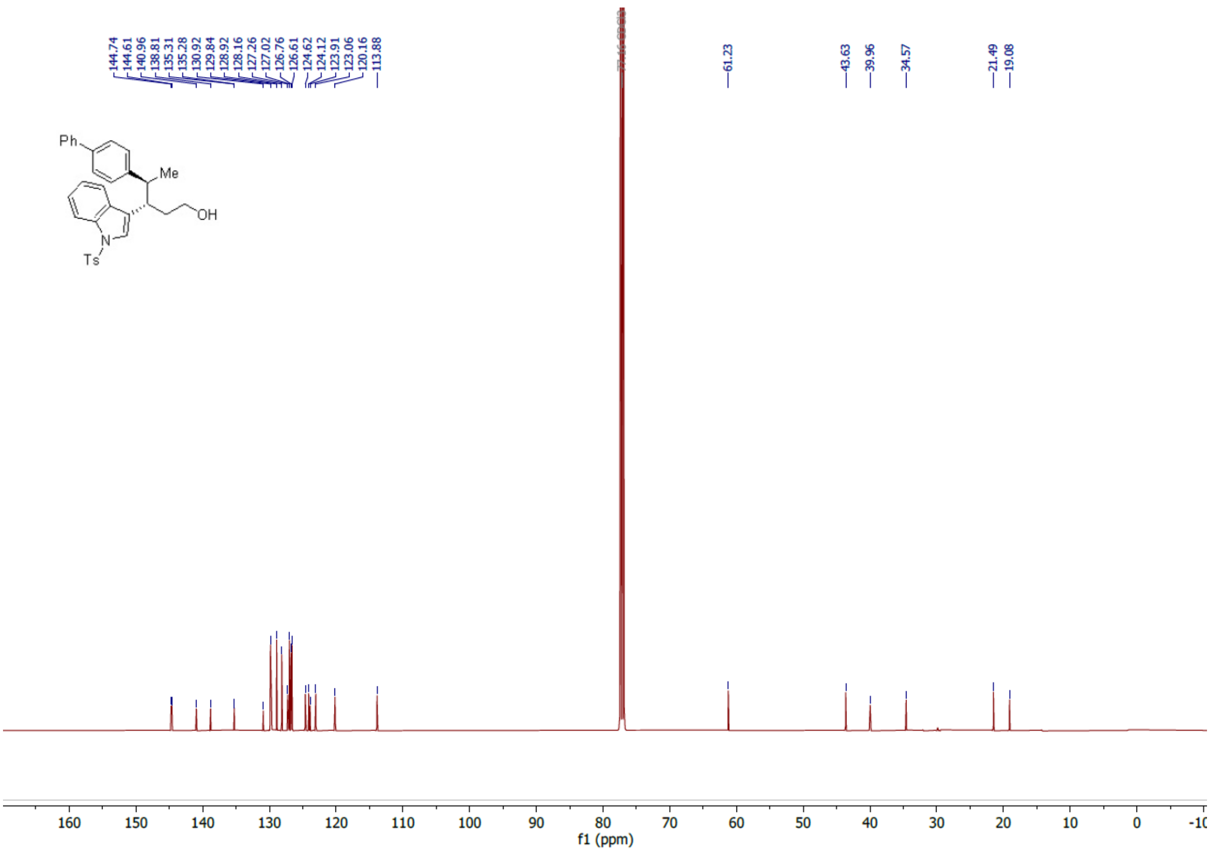

Compound **20**

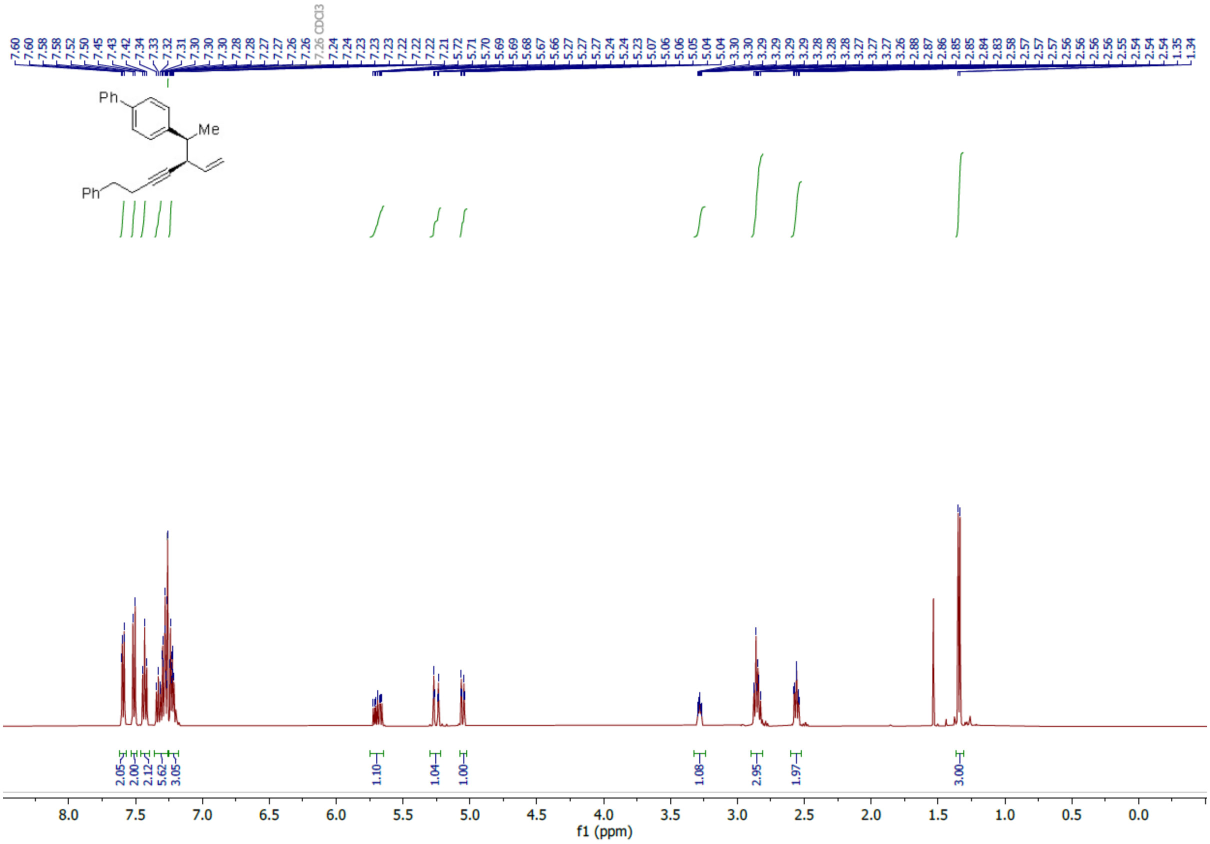

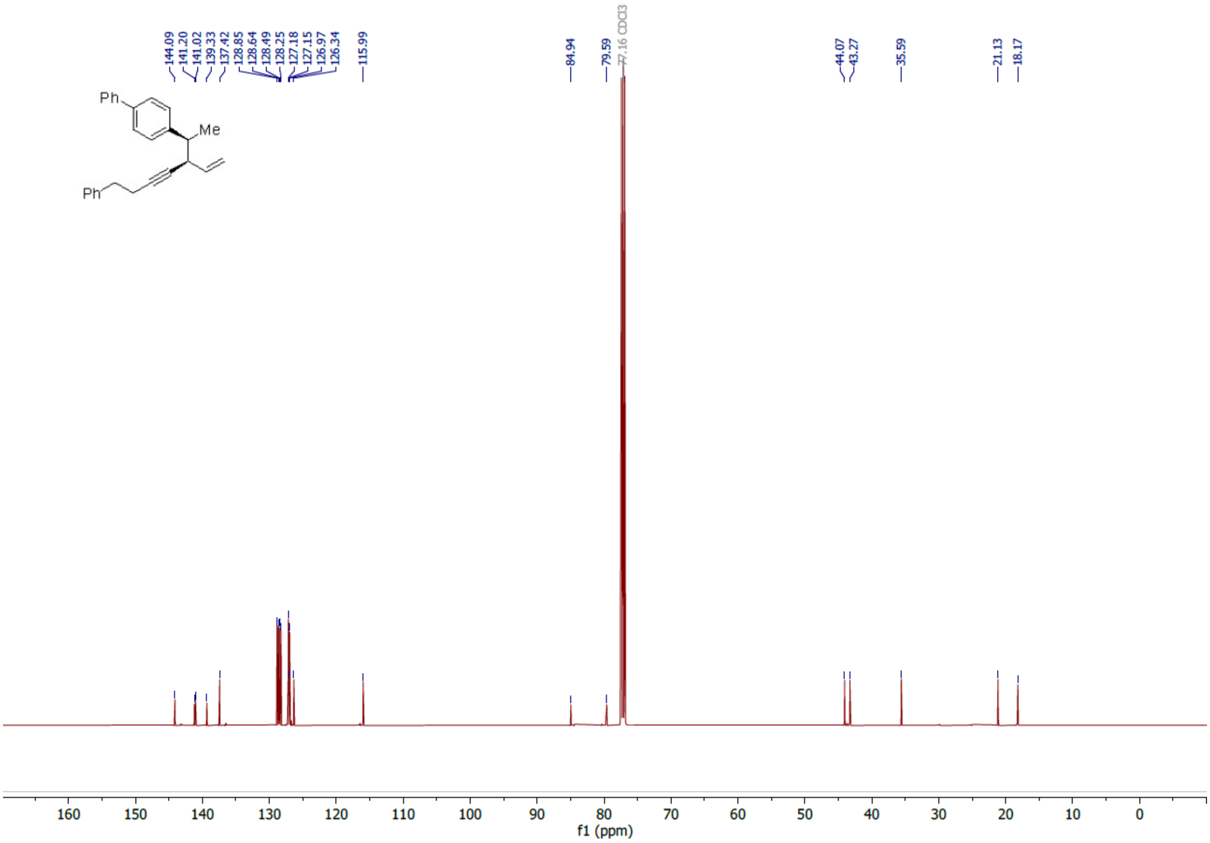

Compound **20'**

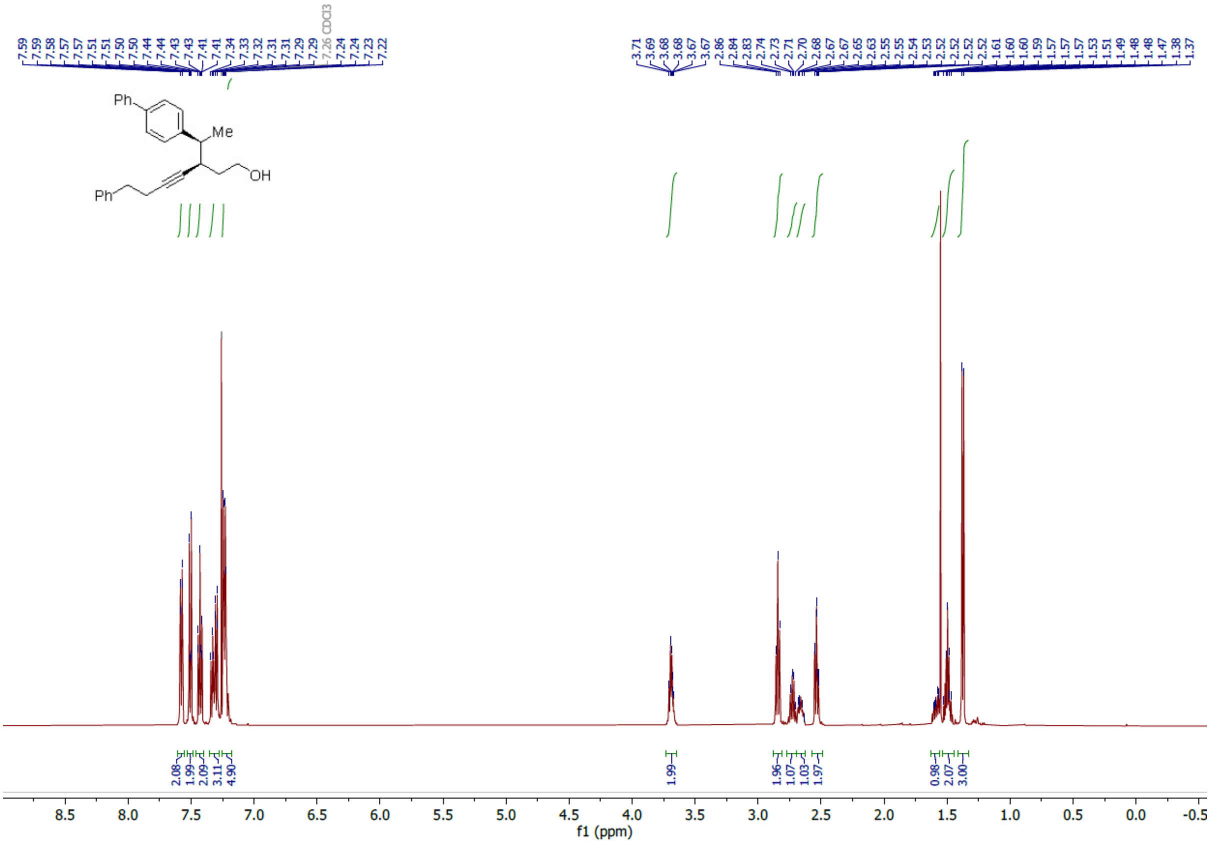
7.59 7.59 7.58 7.57 7.57 7.51 7.51 7.50 7.50 7.44 7.44 7.43 7.43 7.41 7.41 7.34 7.33 7.32 7.31 7.31 7.29 7.29 7.26 CDCl3 7.24 7.24 7.23 7.22
3.71 3.69 3.68 3.68 3.67 3.67 2.86 2.84 2.83 2.74 2.73 2.71 2.70 2.68 2.67 2.65 2.63 2.55 2.55 2.54 2.53 2.52 2.52 2.52 2.52 1.61 1.60 1.60 1.59 1.57 1.57 1.57 1.53 1.51 1.49 1.48 1.47 1.38 1.37
Ph
Me
OH
Ph
2.08 1.99 2.09 3.11 4.90
1.99
1.96 1.07 1.03 1.97
0.98 2.07 3.00
8.5 8.0 7.5 7.0 6.5 6.0 5.5 5.0 4.5 4.0 3.5 3.0 2.5 2.0 1.5 1.0 0.5 0.0 -0.5
f1 (ppm)

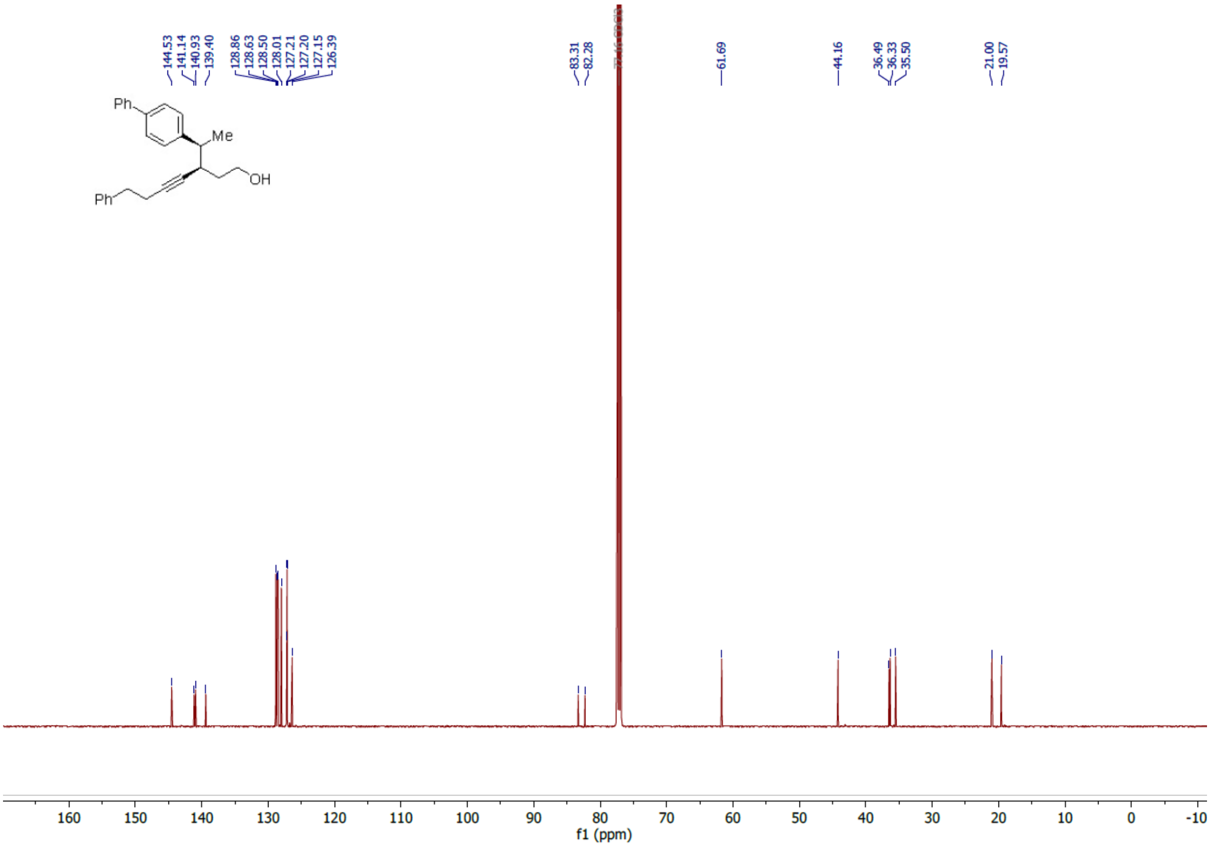
144.53 141.14 140.93 139.40 128.86 128.63 128.50 128.01 127.21 127.20 127.15 126.39
83.31 82.28
61.69
44.16
36.49 36.33 35.50
21.00 19.57
Ph
Me
OH
Ph
160 150 140 130 120 110 100 90 80 70 60 50 40 30 20 10 0 -10
f1 (ppm)

Compound **21**

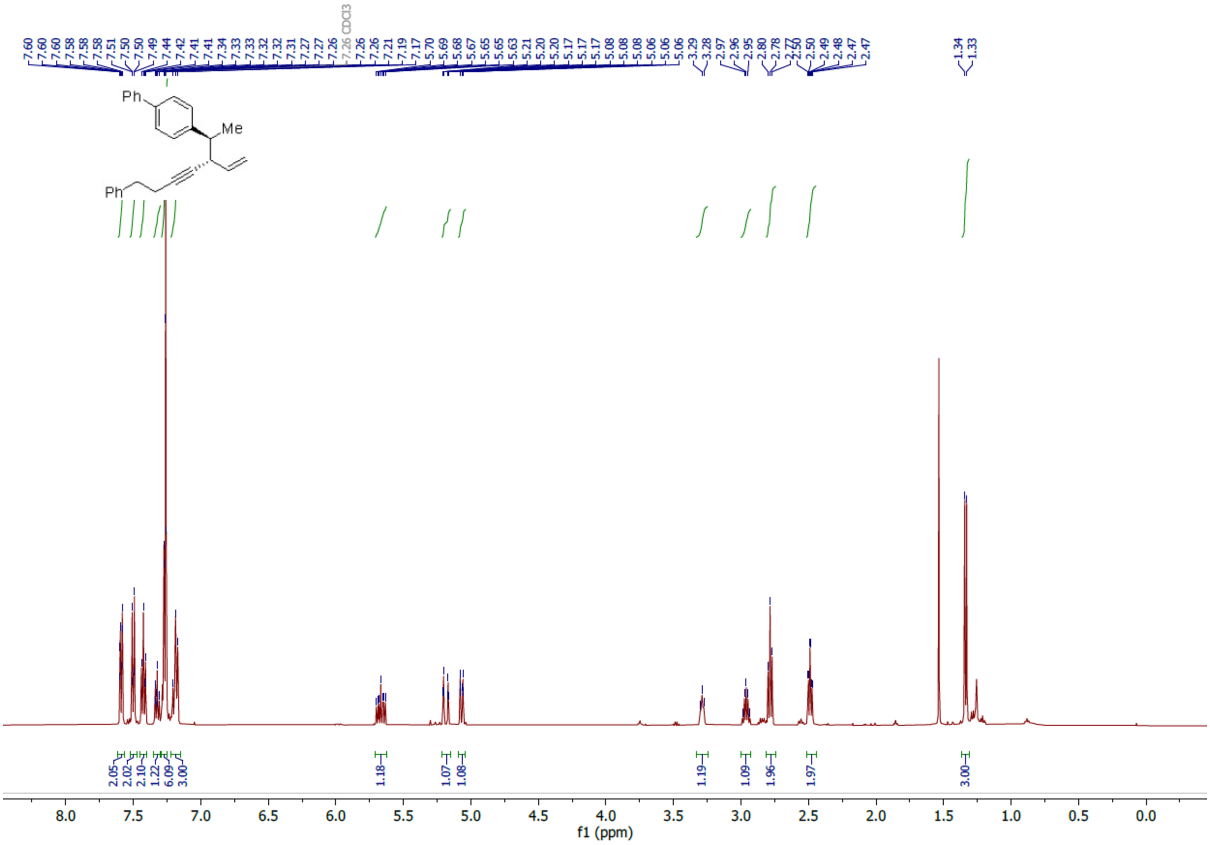

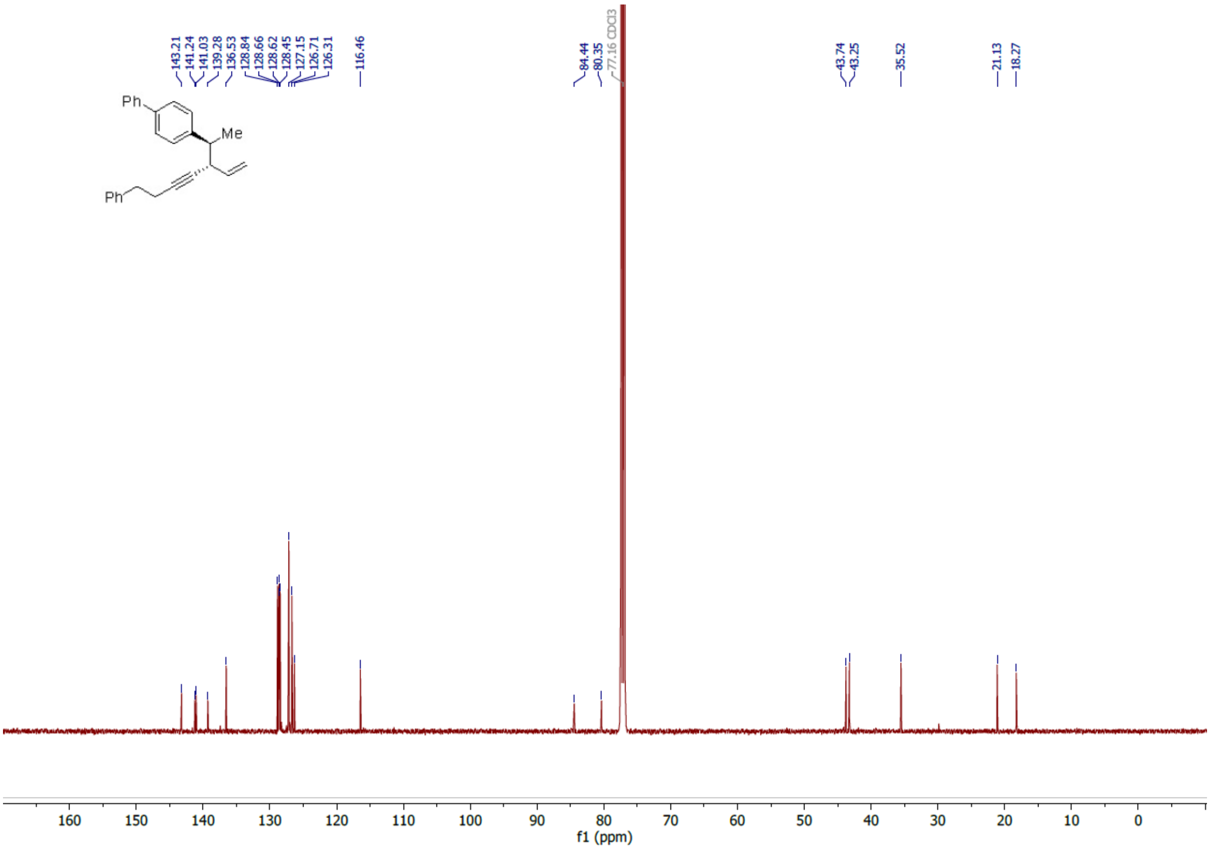

Compound **21'**

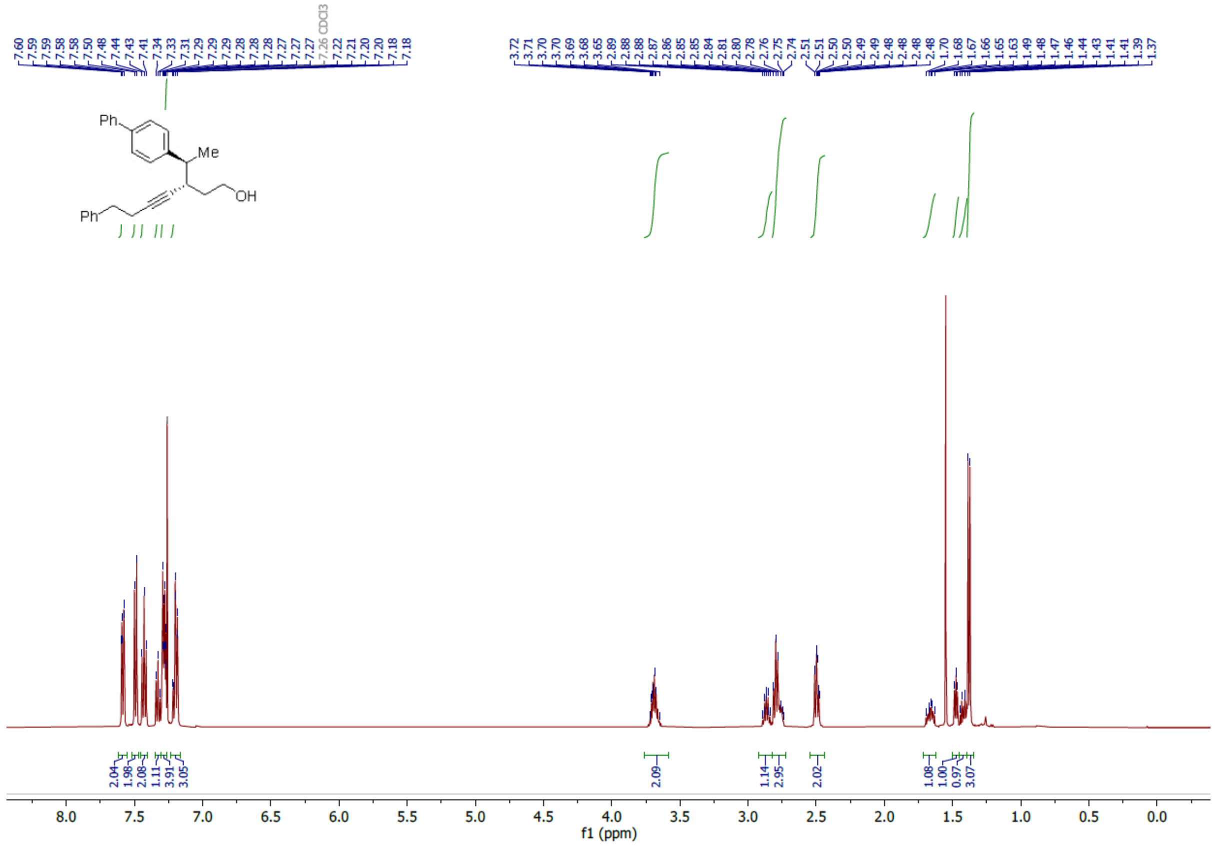

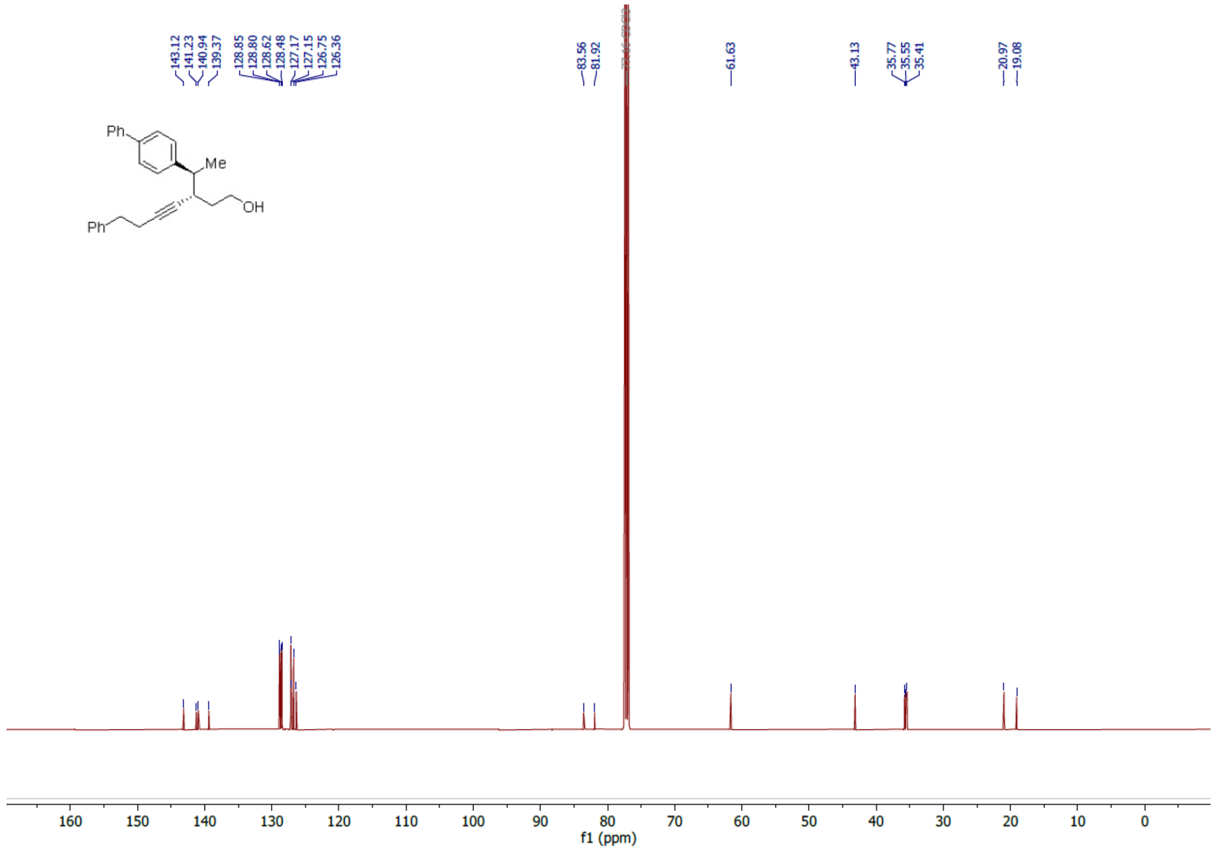

Compound **23**

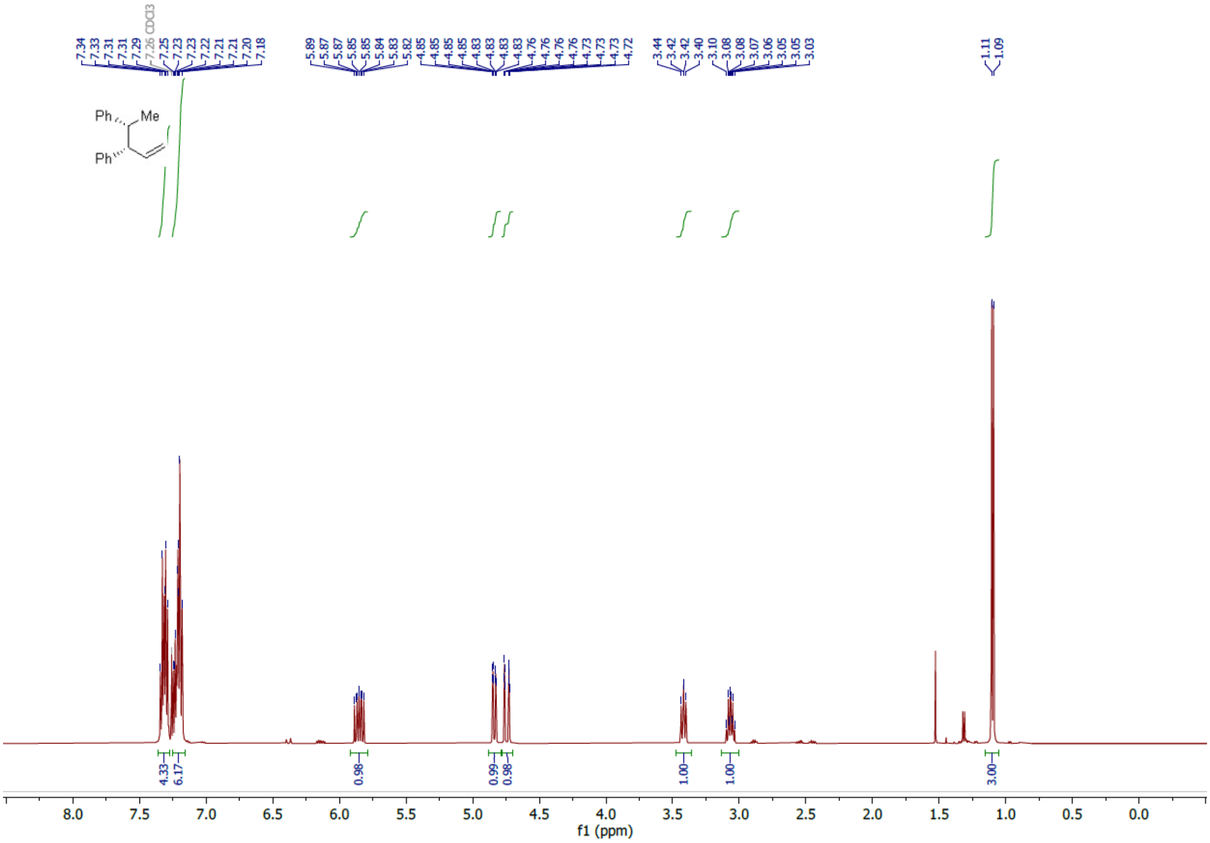

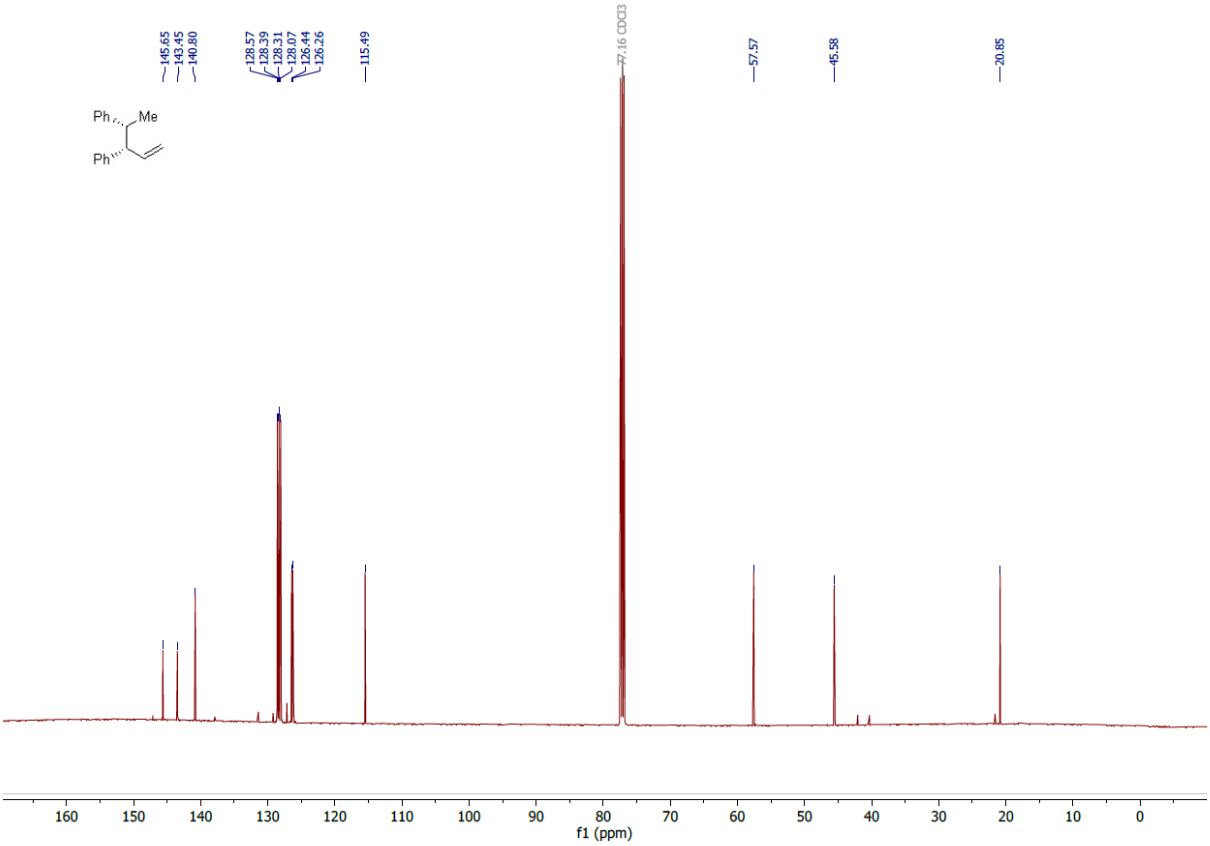

Compound **23'**

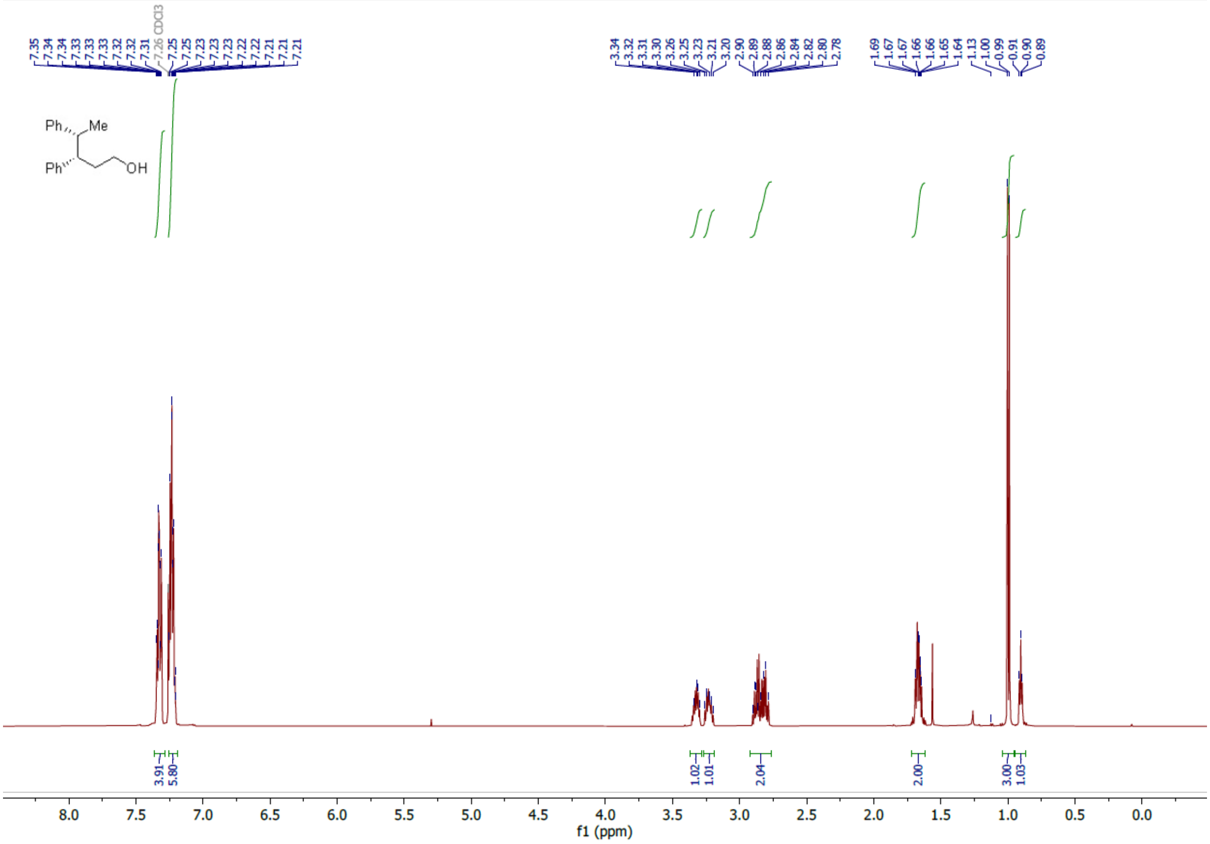

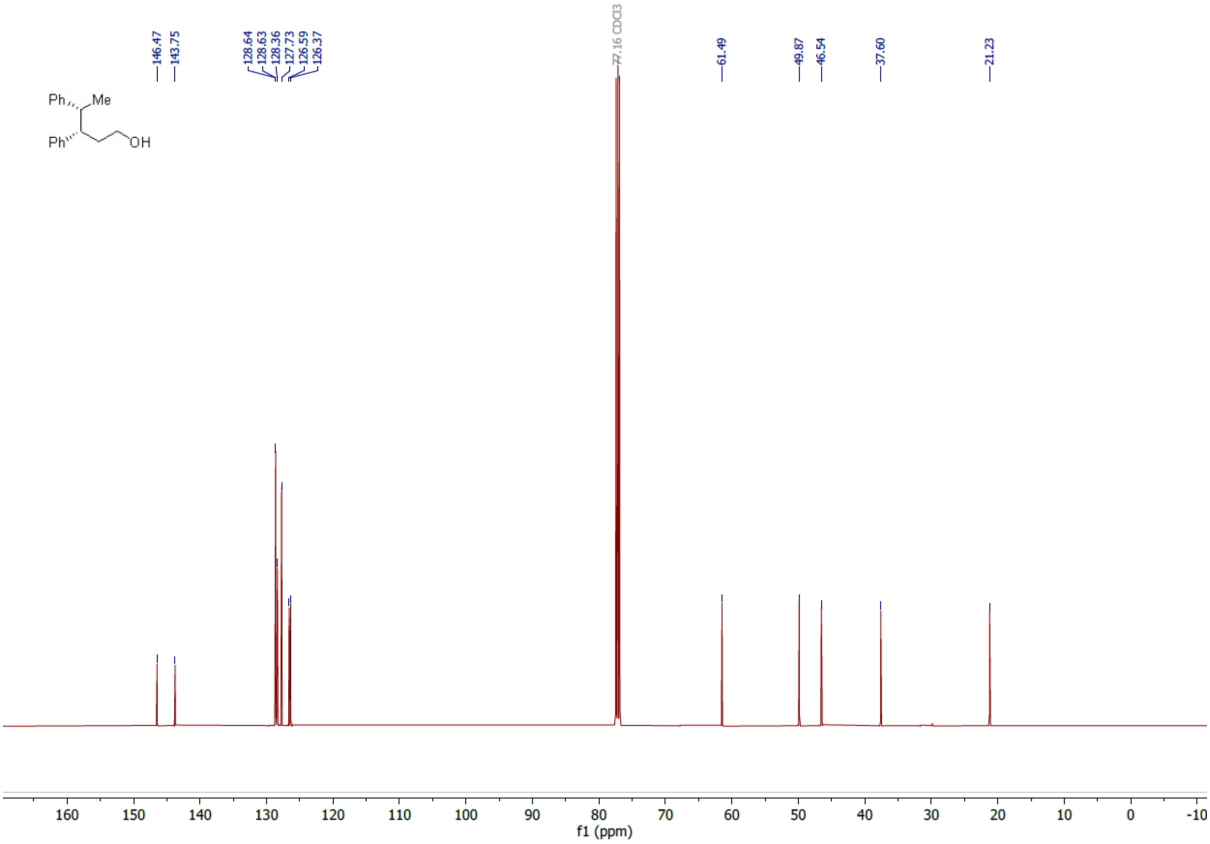

Compound **24**

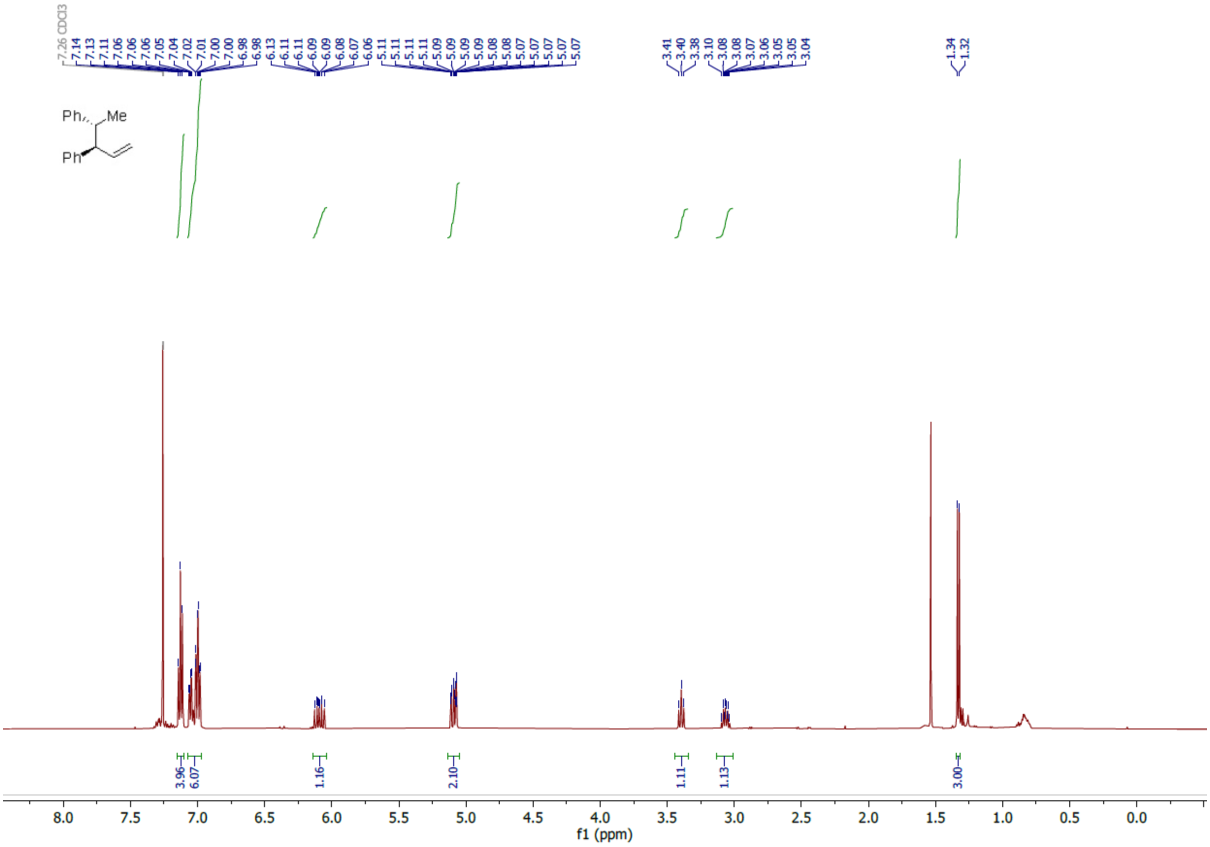

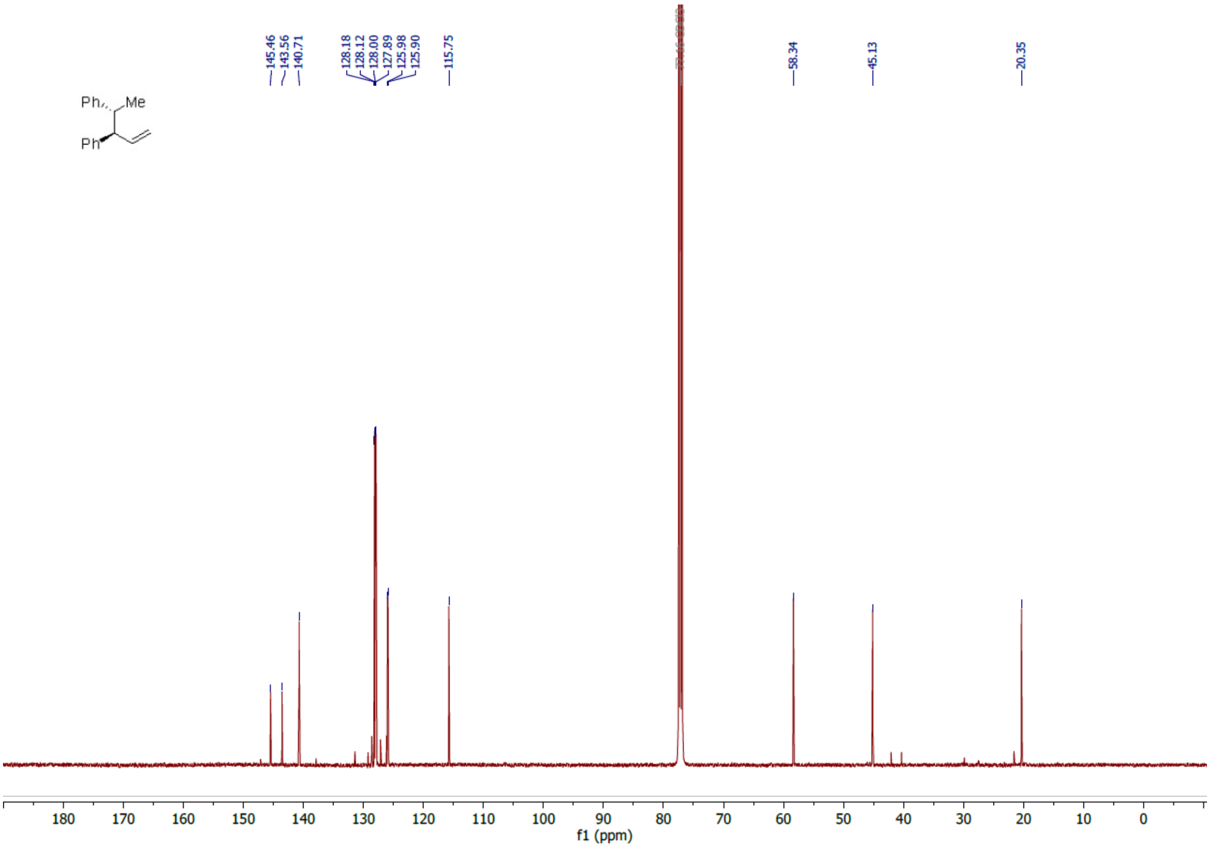

Compound **24'**

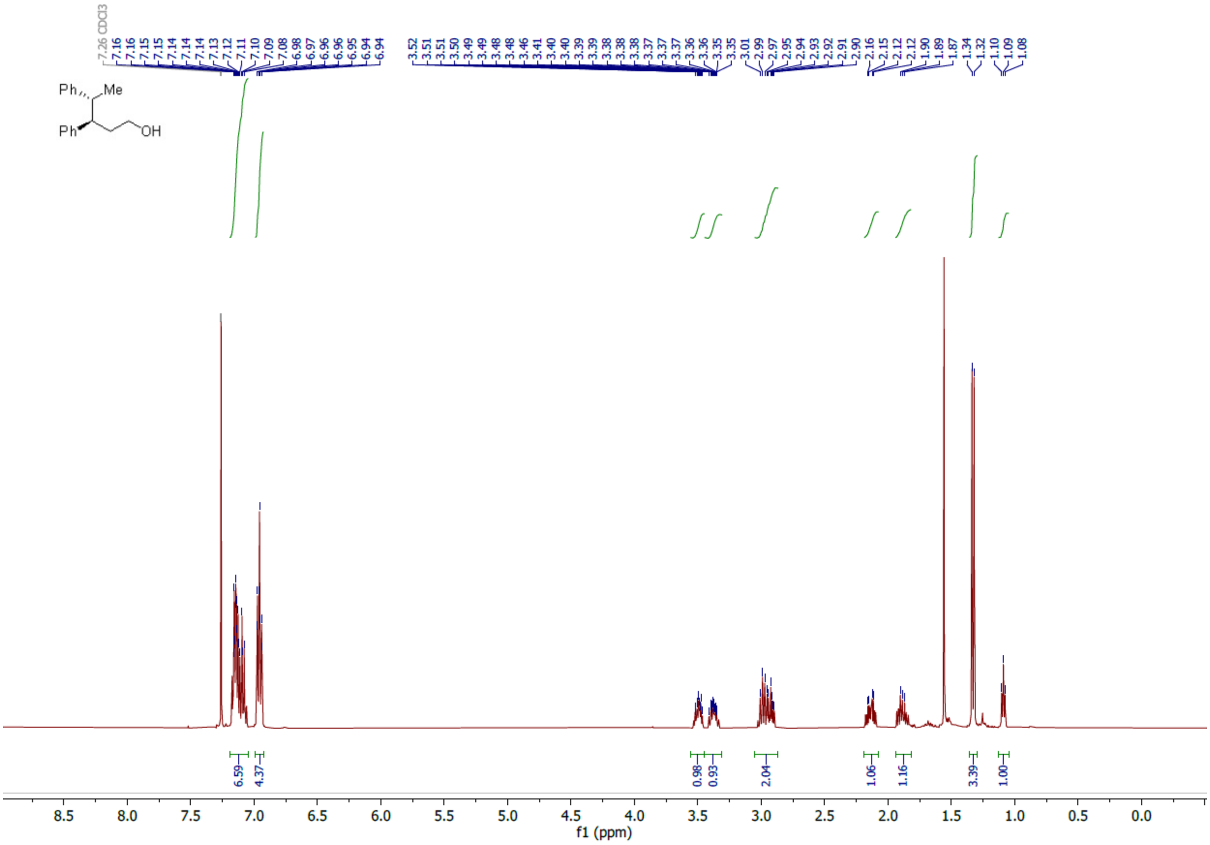
Ph
Me
Ph
OH
f1 (ppm)

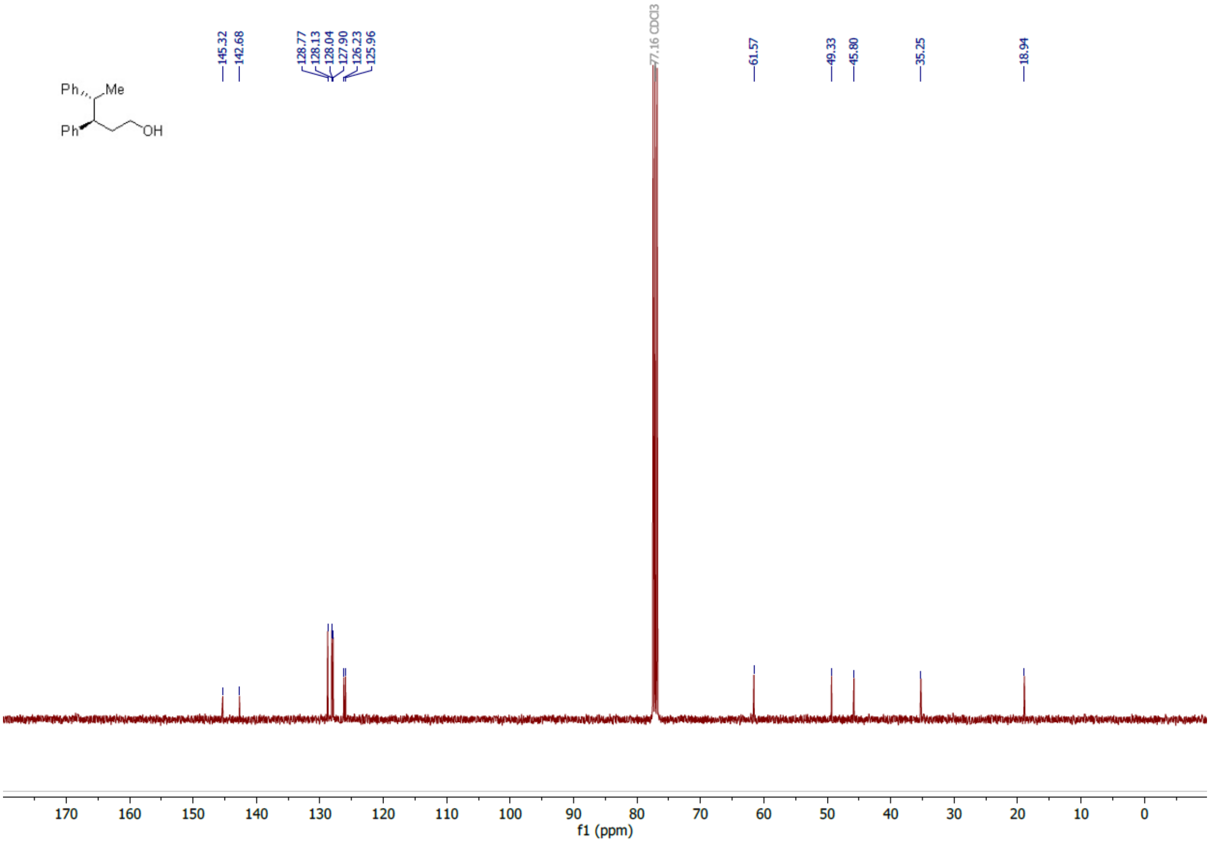
145.32
142.68
128.77
128.13
128.04
127.90
126.23
125.96
77.16 CDCl3
61.57
49.33
45.80
35.25
18.94
Ph
Me
Ph
OH
f1 (ppm)

Compound **26**

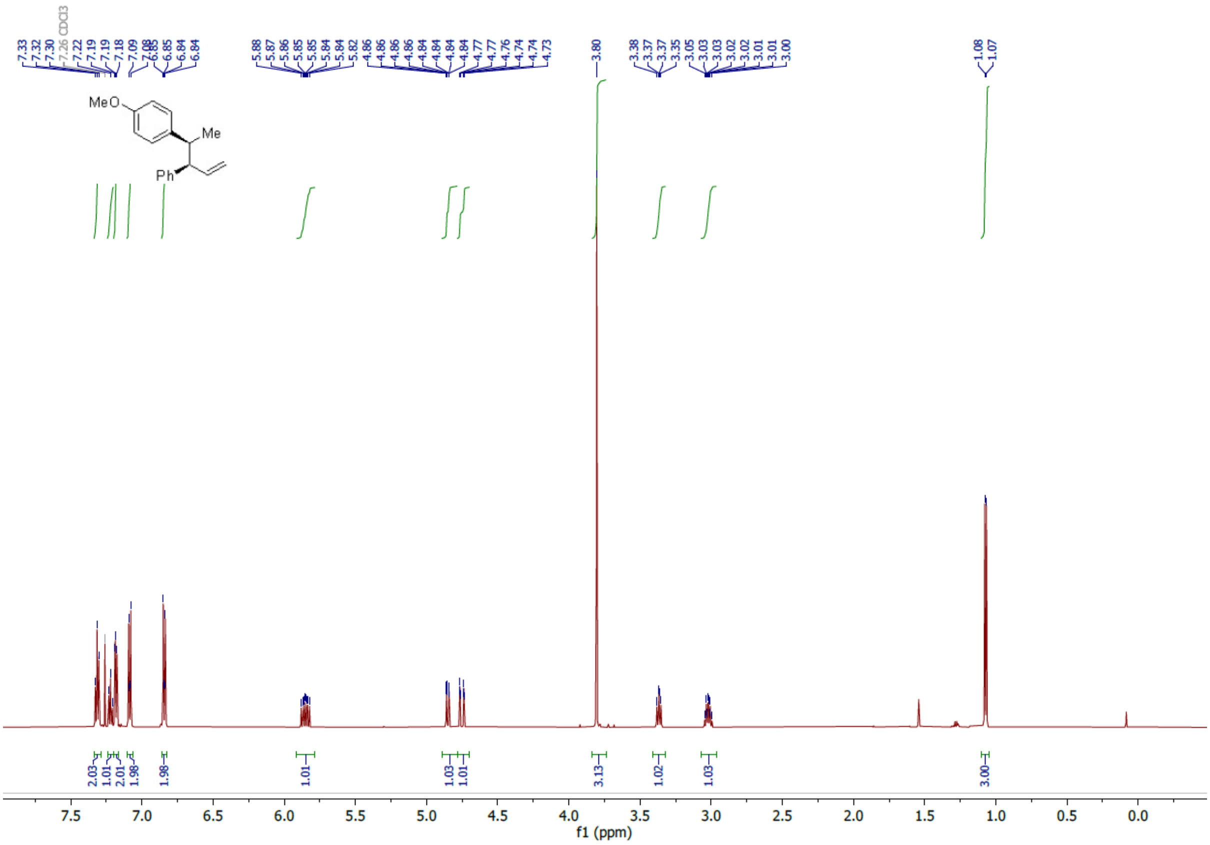

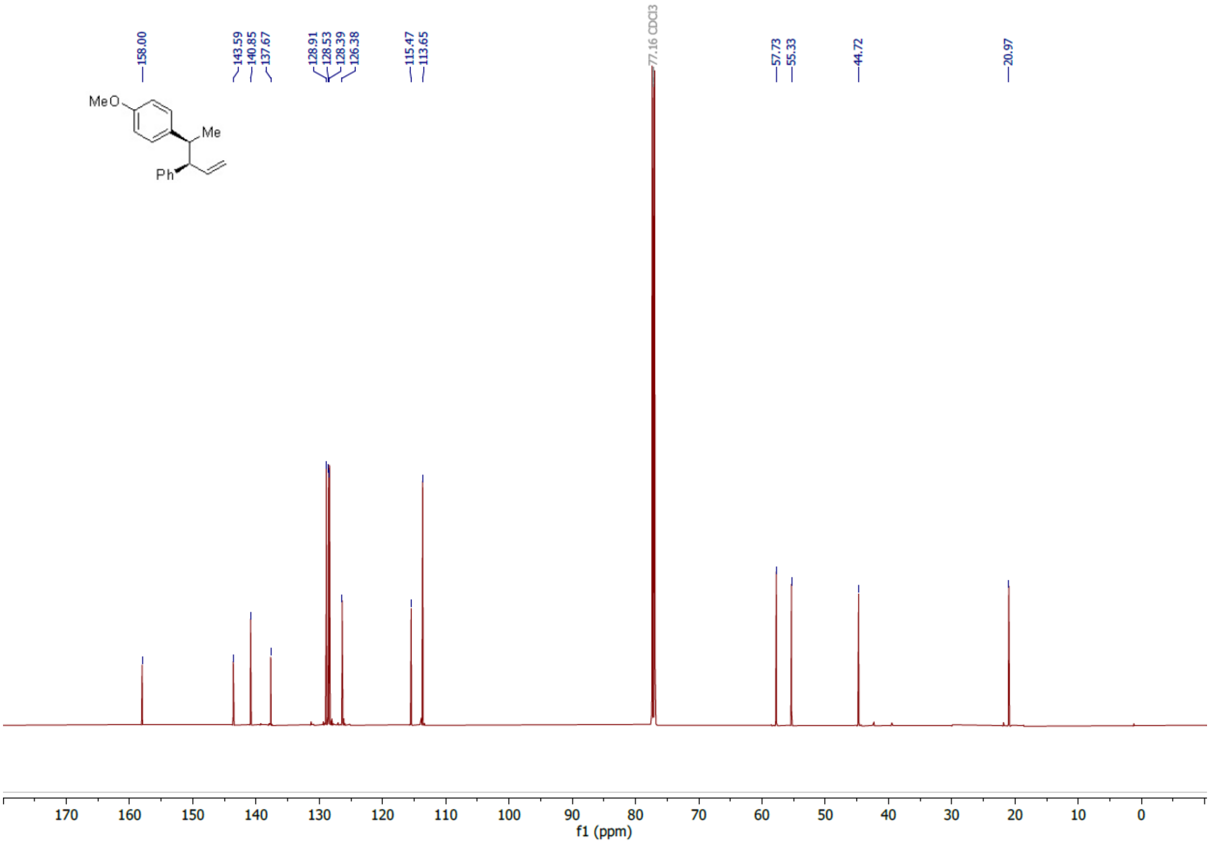

Compound **26'**

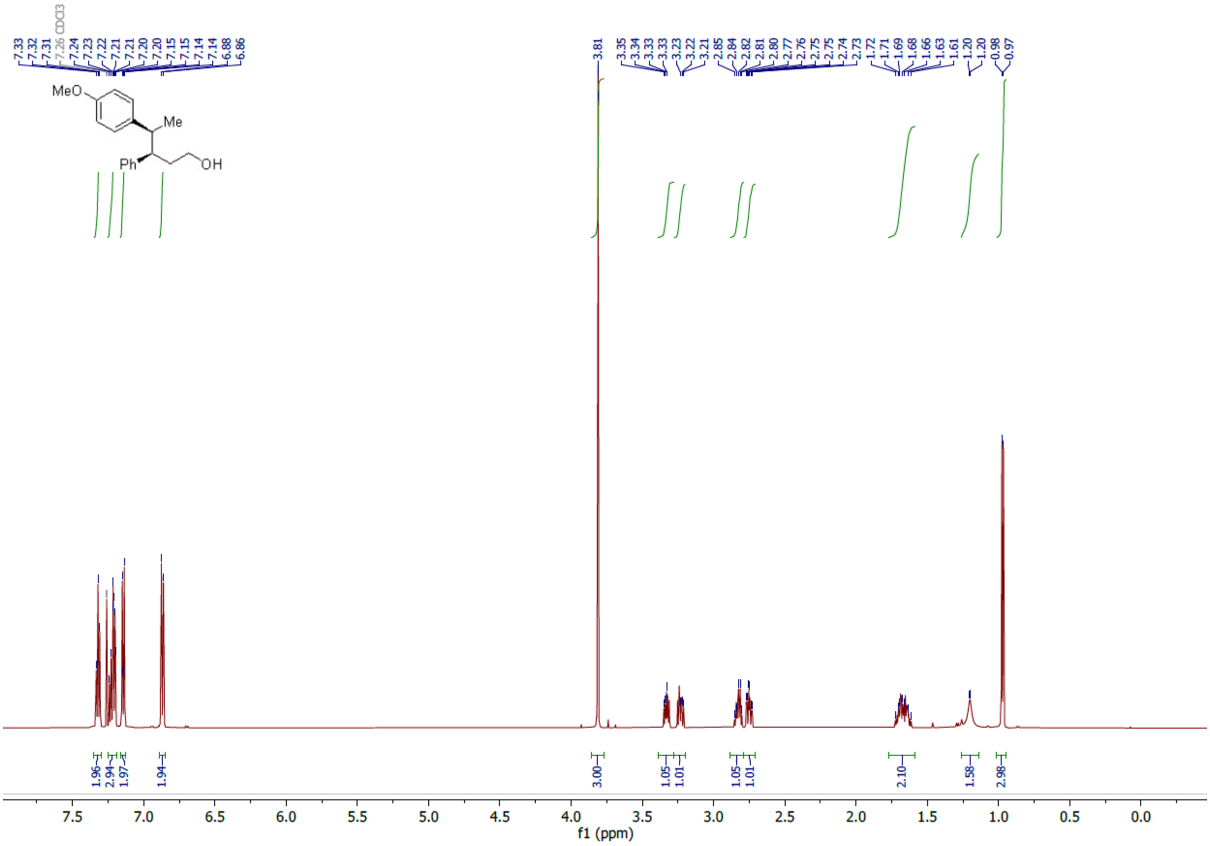
7.33
7.32
7.31
7.26 CDCl3
7.24
7.23
7.22
7.21
7.21
7.20
7.15
7.15
7.14
6.88
6.86
3.81
3.35
3.34
3.33
3.33
3.23
3.22
3.21
2.85
2.84
2.82
2.81
2.80
2.77
2.76
2.75
2.75
2.74
2.73
1.72
1.71
1.69
1.68
1.66
1.63
1.61
1.20
1.20
0.98
0.97
MeO
Me
Ph
OH
1.96
2.94
1.97
1.94
3.00
1.05
1.01
1.05
1.01
2.10
1.58
2.98
7.5 7.0 6.5 6.0 5.5 5.0 4.5 4.0 3.5 3.0 2.5 2.0 1.5 1.0 0.5 0.0
f1 (ppm)

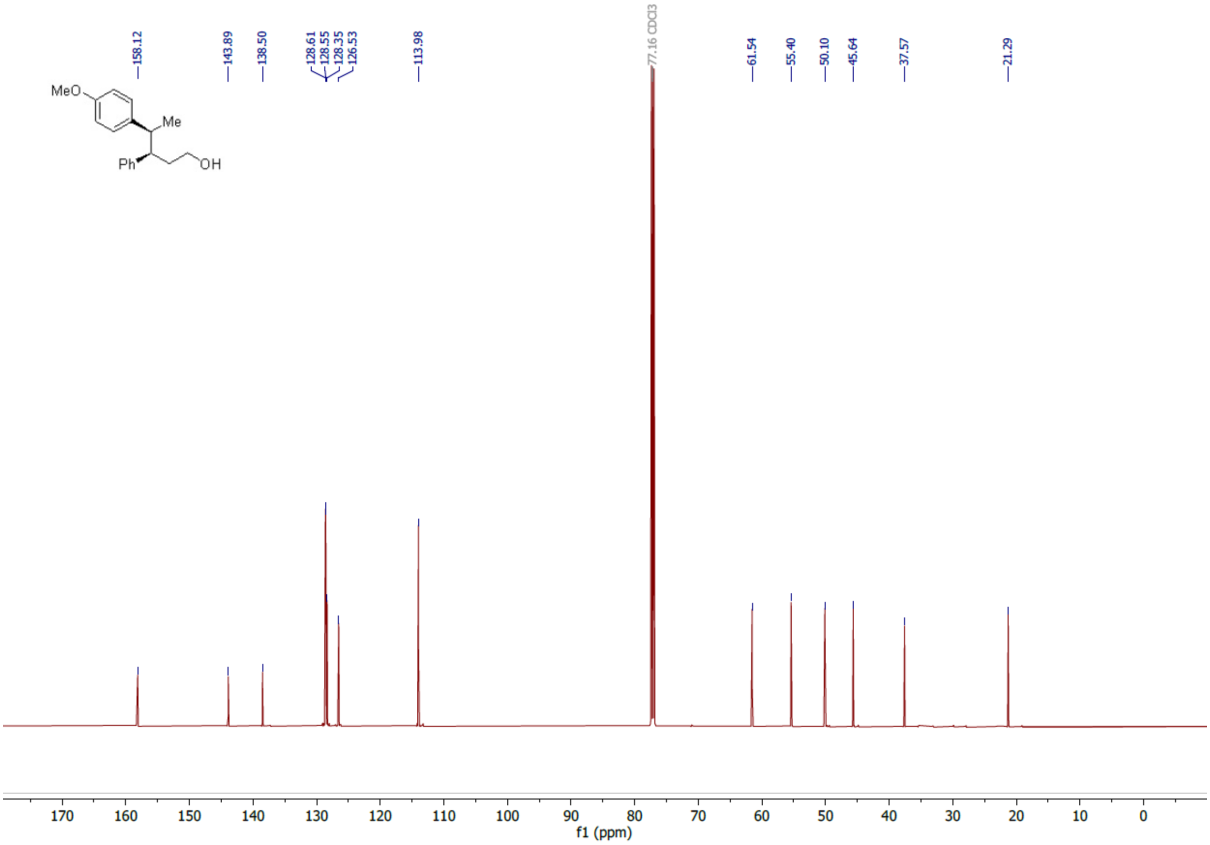
158.12
143.89
138.50
128.61
128.55
128.35
126.53
113.98
77.16 CDCl3
61.54
55.40
50.10
45.64
37.57
21.29
MeO
Me
Ph
OH
170 160 150 140 130 120 110 100 90 80 70 60 50 40 30 20 10 0
f1 (ppm)

Compound **27**

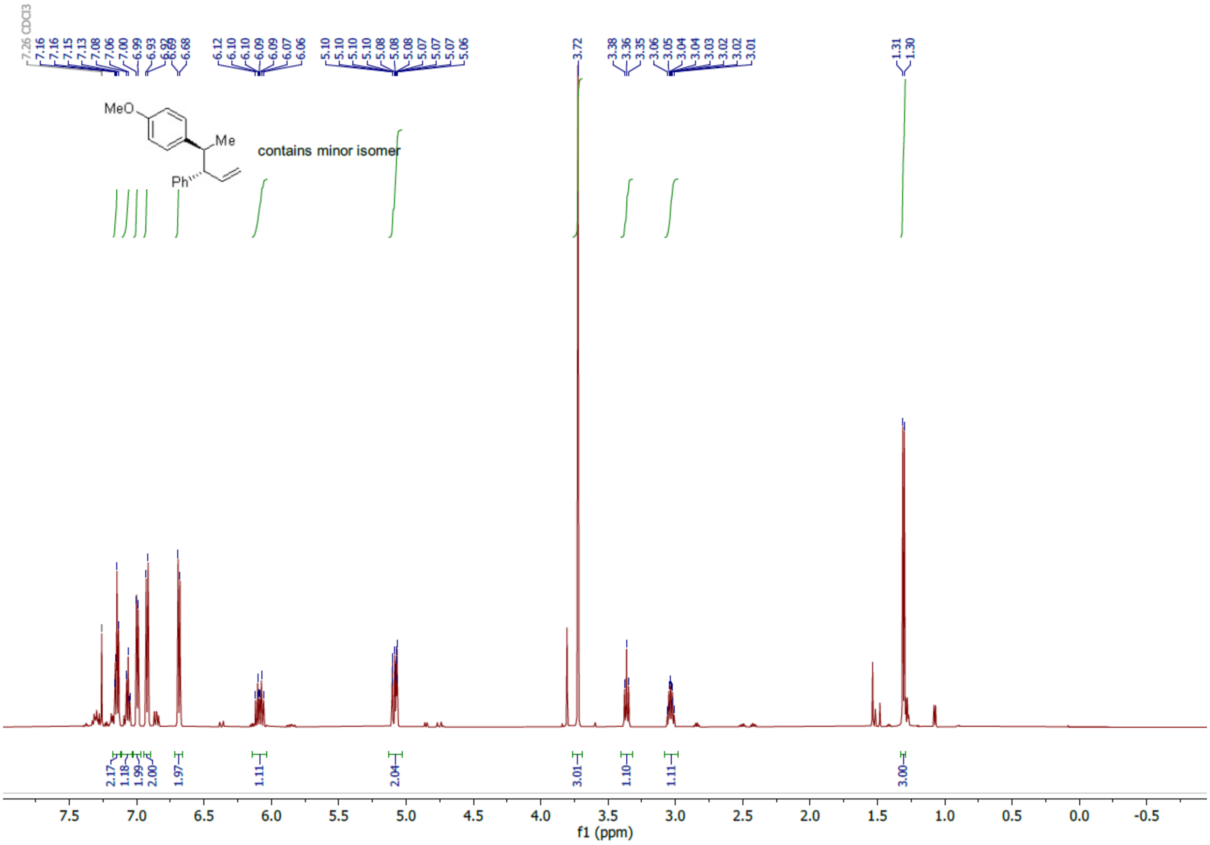
MeO
Me
Ph
contains minor isomer
f1 (ppm)

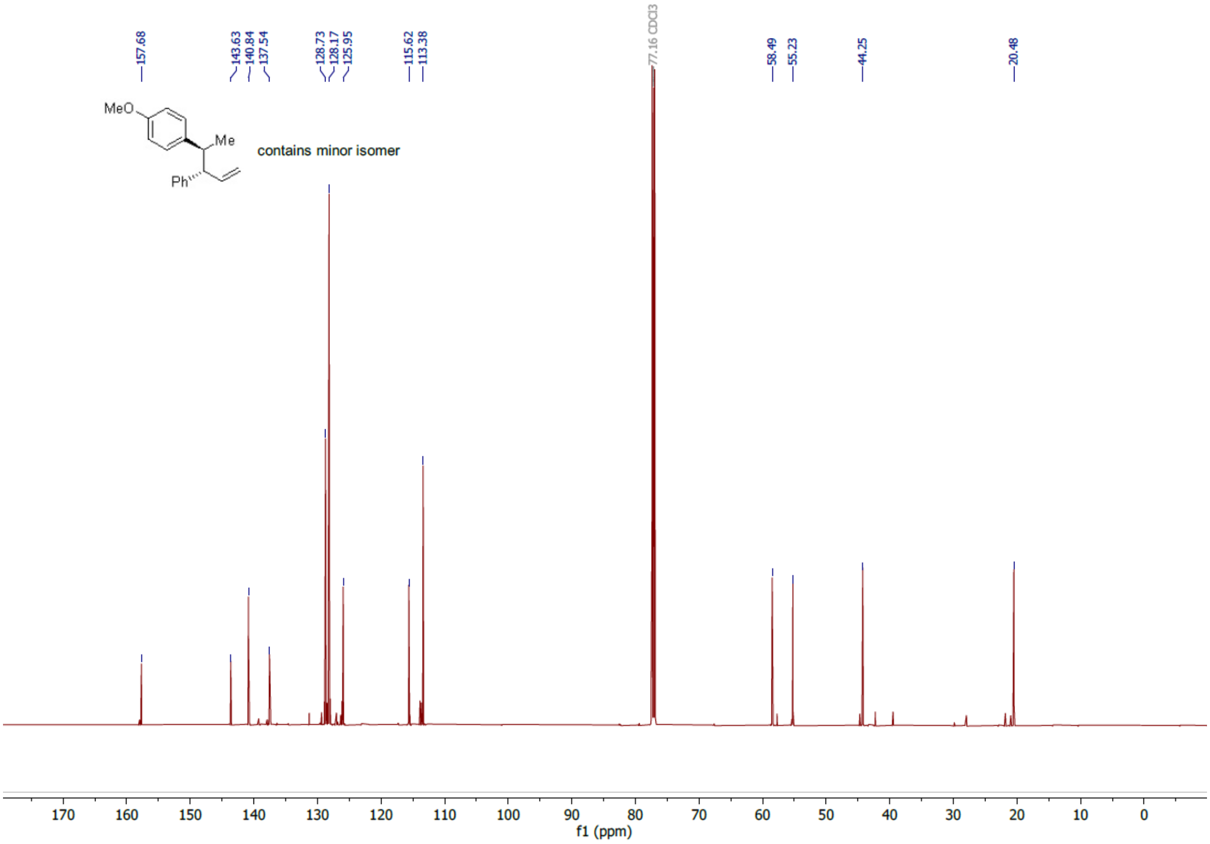
MeO
Me
Ph
contains minor isomer
f1 (ppm)

Compound **27'**

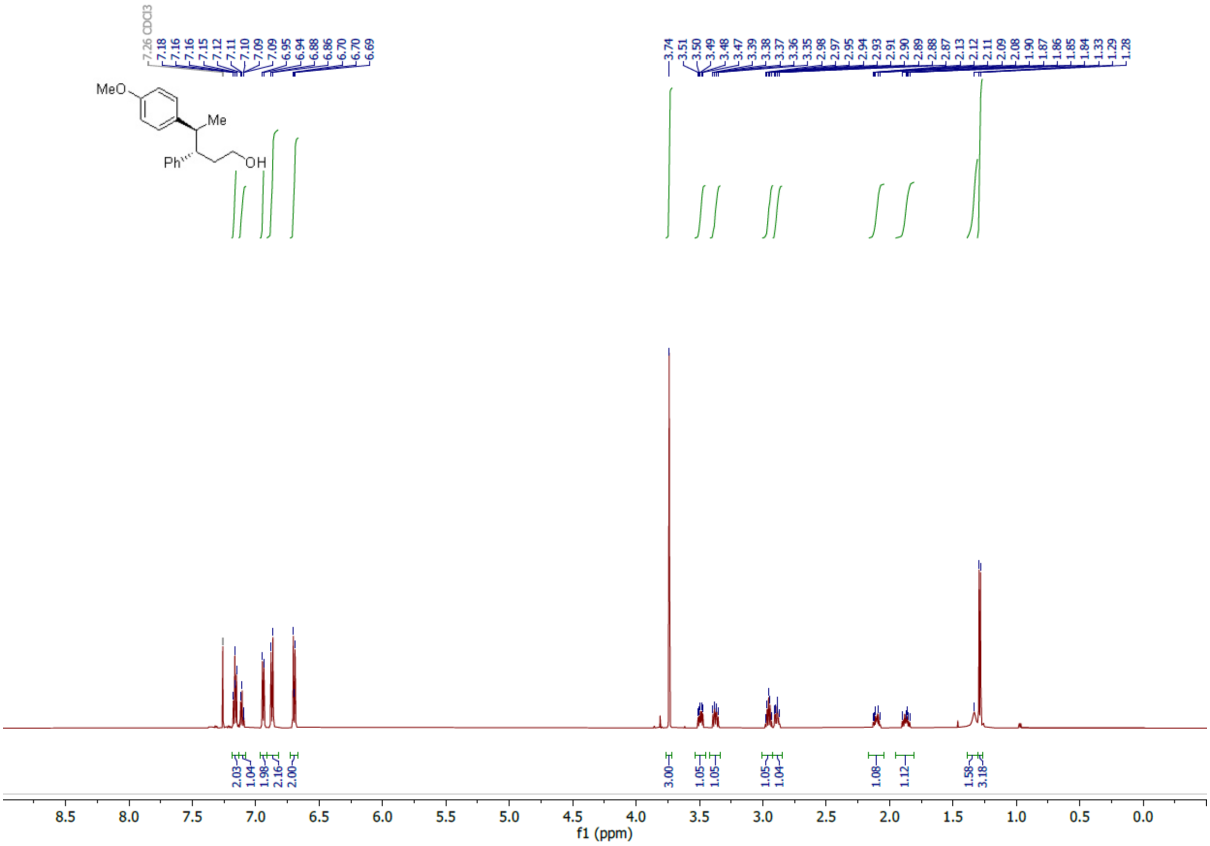

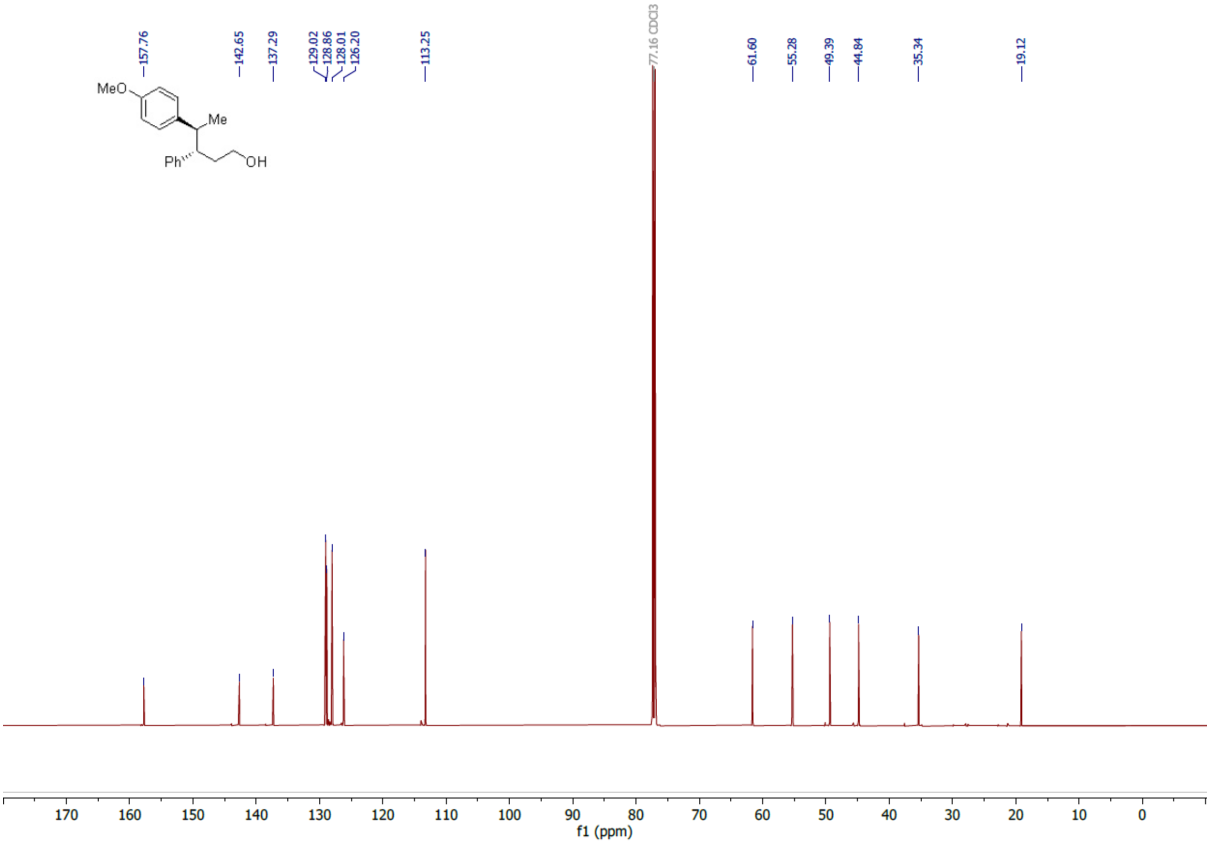

Compound **29**

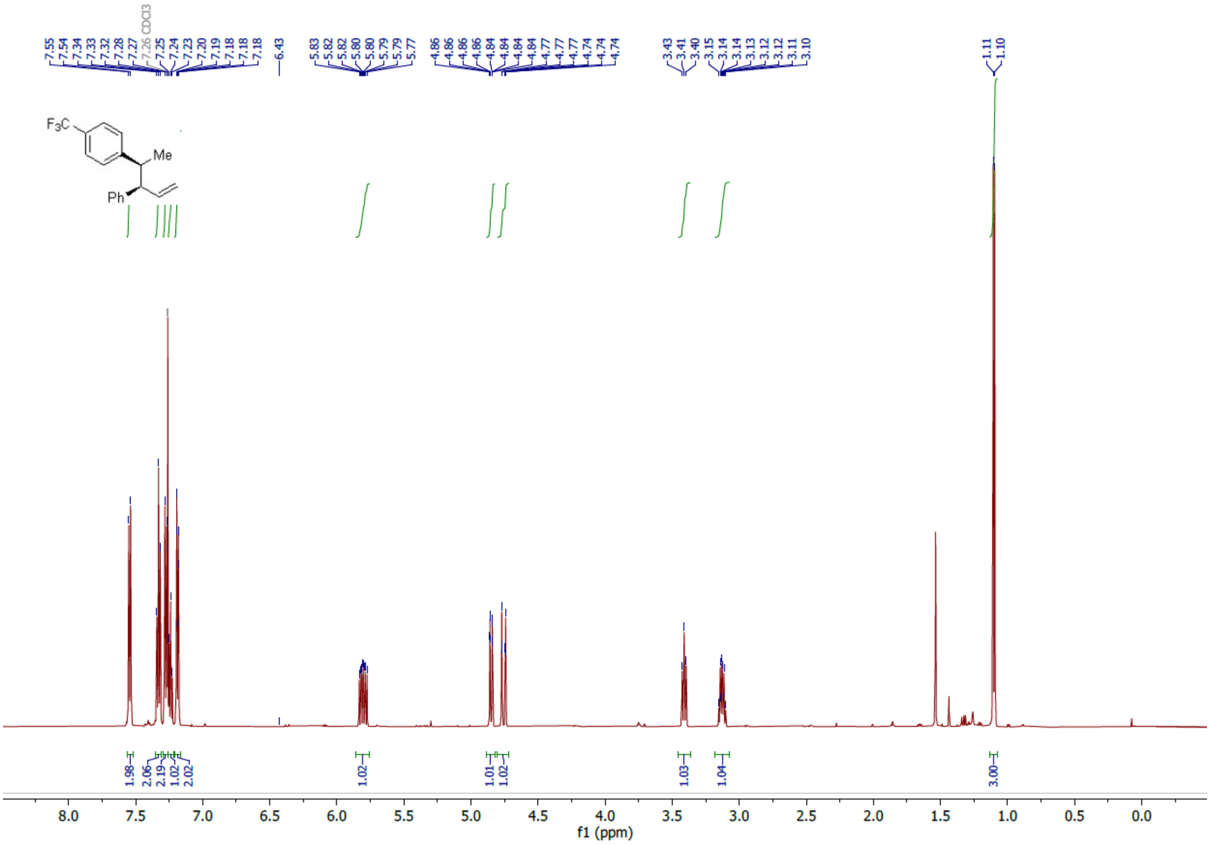

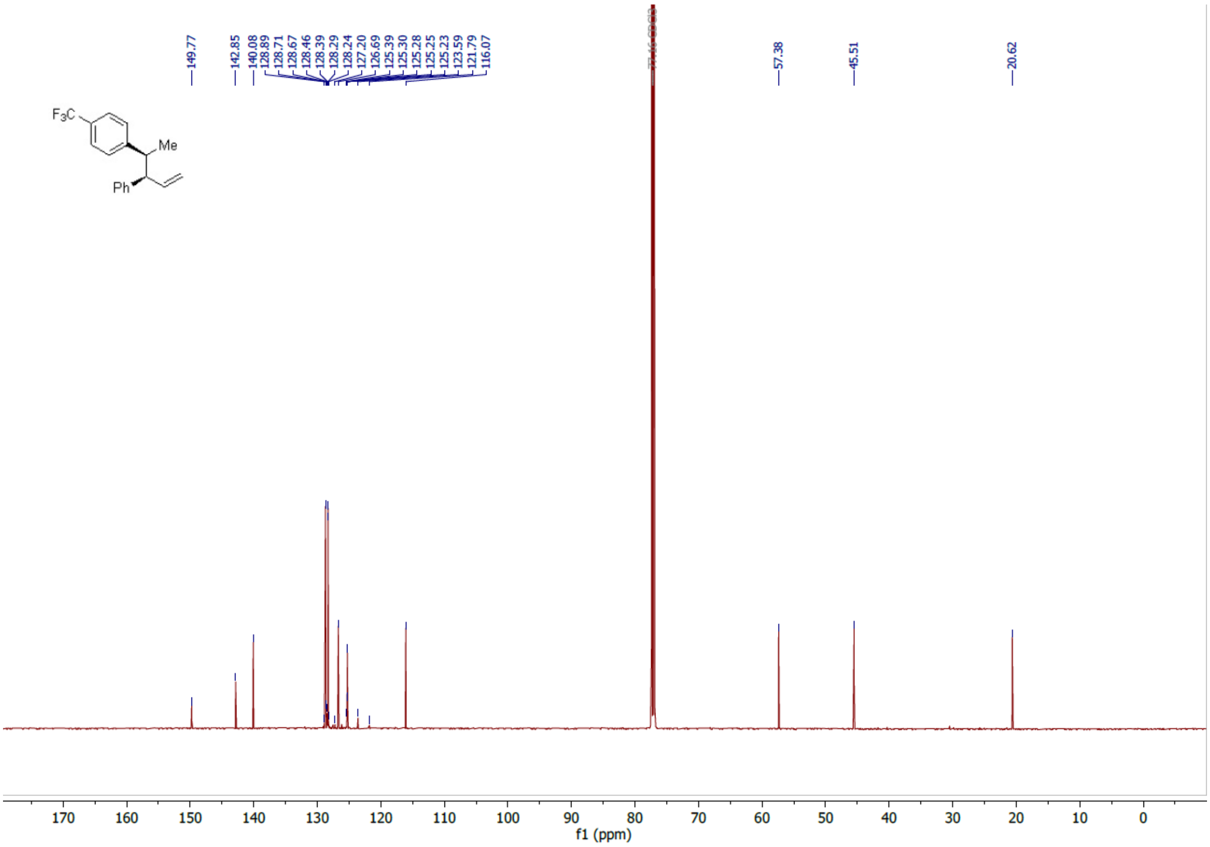

Compound **29'**

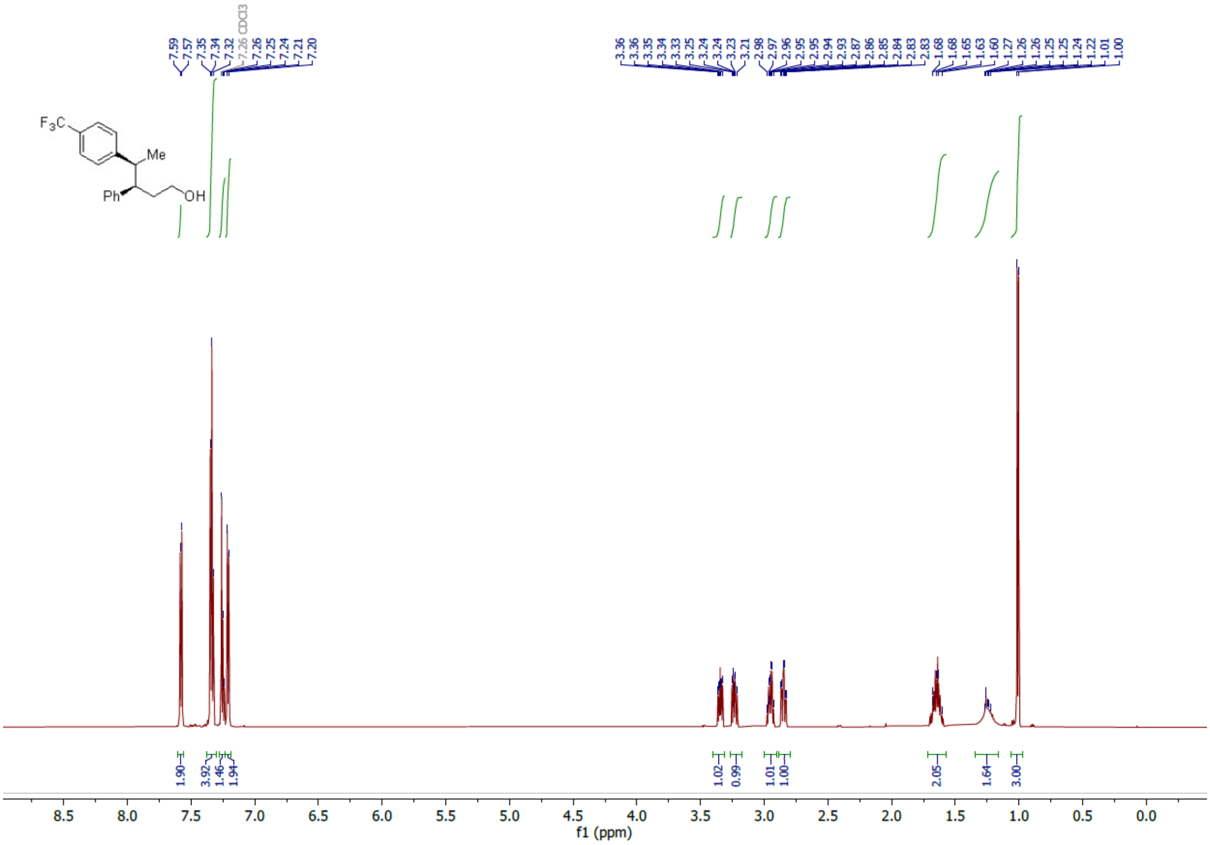
7.59
7.57
7.35
7.34
7.32
7.26 CDCl3
7.26
7.25
7.24
7.21
7.20
3.36
3.36
3.35
3.34
3.33
3.25
3.24
3.24
3.23
3.21
2.98
2.97
2.96
2.95
2.95
2.94
2.93
2.87
2.86
2.85
2.84
2.83
2.83
1.68
1.68
1.65
1.63
1.60
1.27
1.26
1.26
1.25
1.25
1.24
1.22
1.01
1.00
F3C
Me
Ph
OH
1.90
3.92
1.46
1.94
1.02
0.99
1.01
1.00
2.05
1.64
3.00
f1 (ppm)

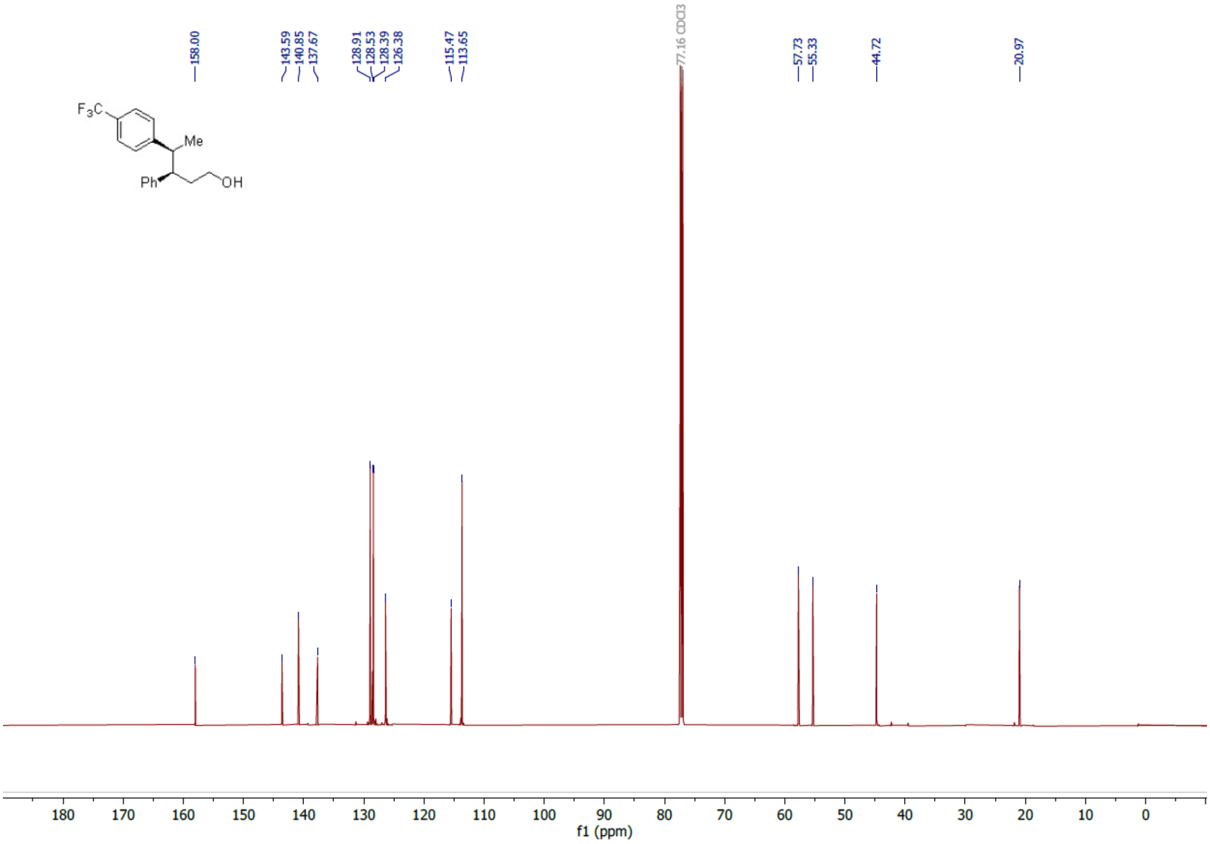
158.00
143.59
140.85
137.67
128.91
128.53
128.39
126.38
115.47
113.65
77.16 CDCl3
57.73
55.33
44.72
20.97
F3C
Me
Ph
OH
f1 (ppm)

Compound **30**

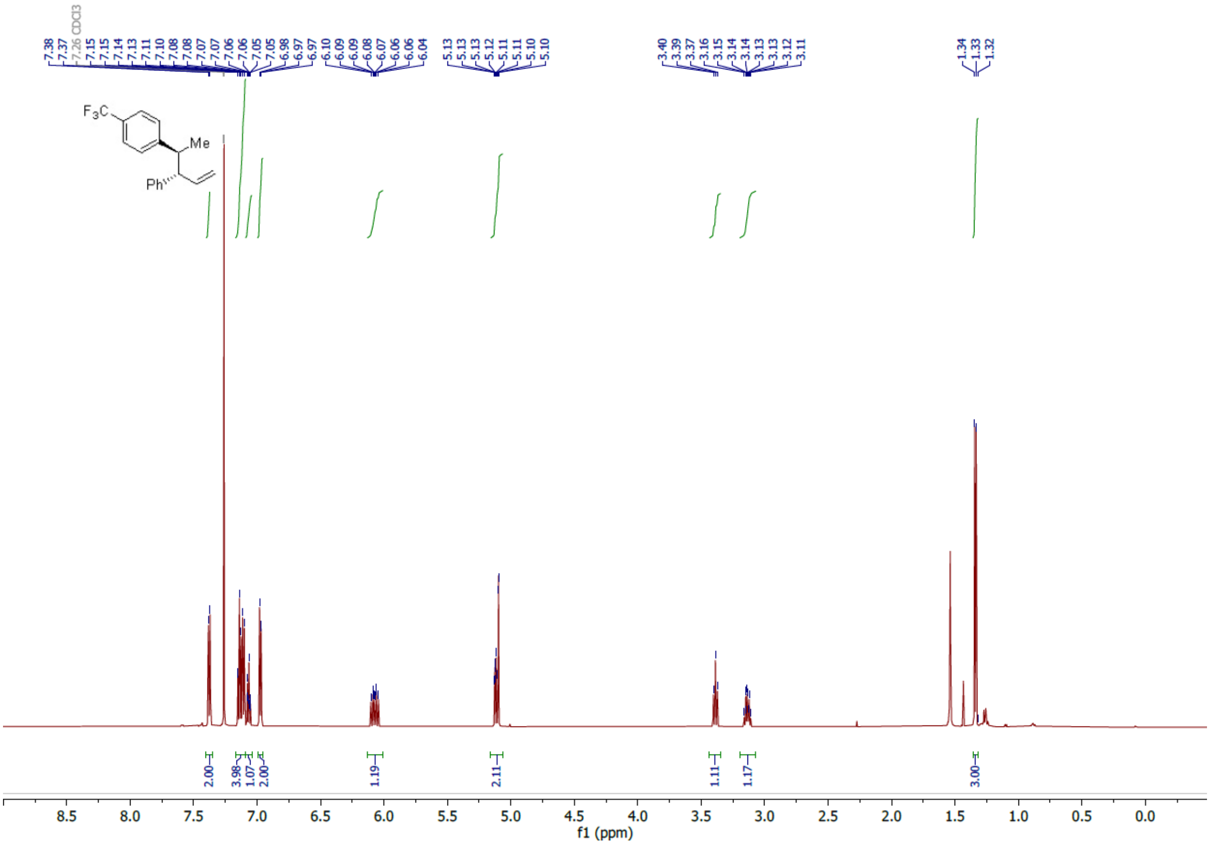
7.38
7.37
7.26 CDCl3
7.15
7.15
7.14
7.13
7.11
7.10
7.08
7.08
7.07
7.07
7.06
7.06
7.05
7.05
6.98
6.97
6.97
6.10
6.09
6.09
6.08
6.08
6.07
6.06
6.06
6.04
5.13
5.13
5.13
5.12
5.11
5.11
5.11
5.10
5.10
3.40
3.39
3.37
3.16
3.15
3.14
3.14
3.13
3.13
3.12
3.11
1.34
1.33
1.32
F_3C
Me
Ph
2.00
3.98
1.07
2.00
1.19
2.11
1.11
1.17
3.00
8.5
8.0
7.5
7.0
6.5
6.0
5.5
5.0
4.5
4.0
3.5
3.0
2.5
2.0
1.5
1.0
0.5
0.0
f1 (ppm)

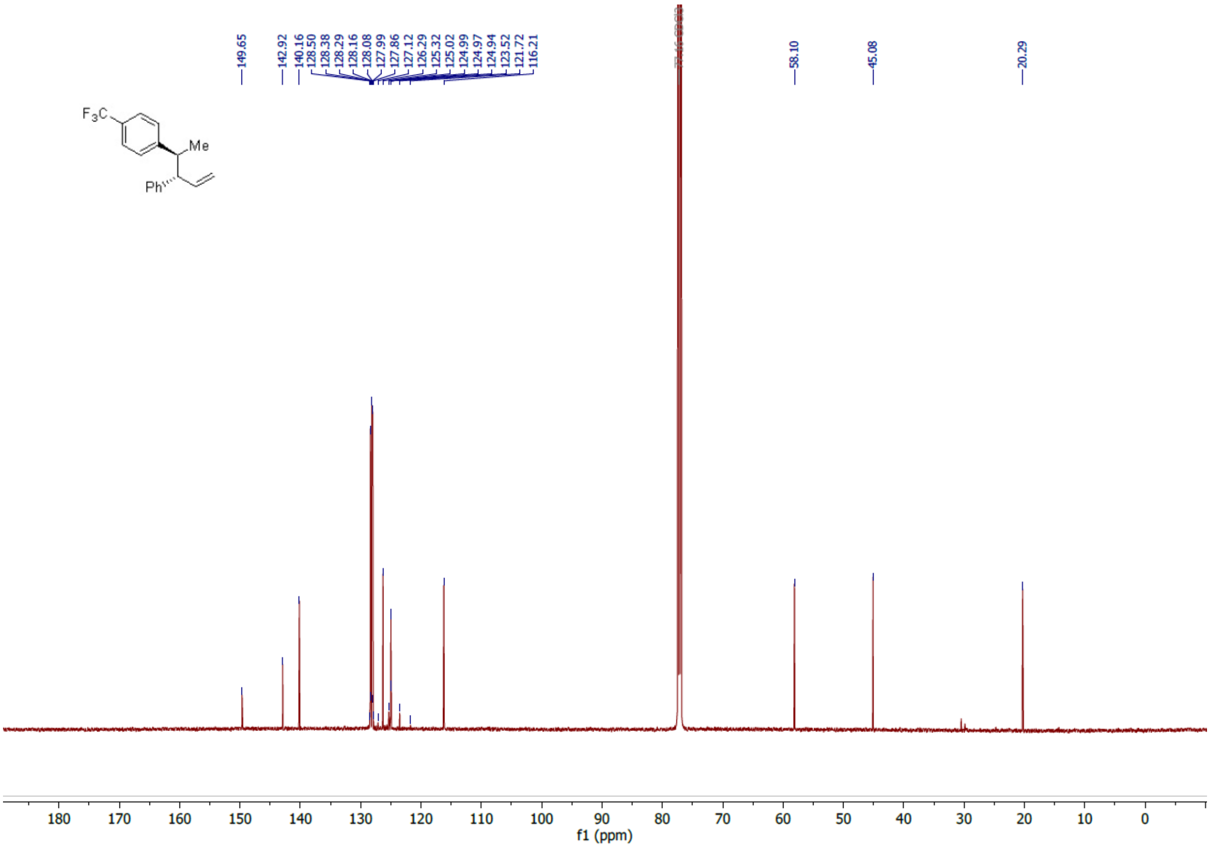
149.65
142.92
140.16
128.50
128.38
128.29
128.16
128.08
127.99
127.86
127.12
126.29
125.32
125.02
124.99
124.97
124.94
123.52
121.72
116.21
58.10
45.08
20.29
F_3C
Me
Ph
180
170
160
150
140
130
120
110
100
90
80
70
60
50
40
30
20
10
0
f1 (ppm)

Compound **30'**

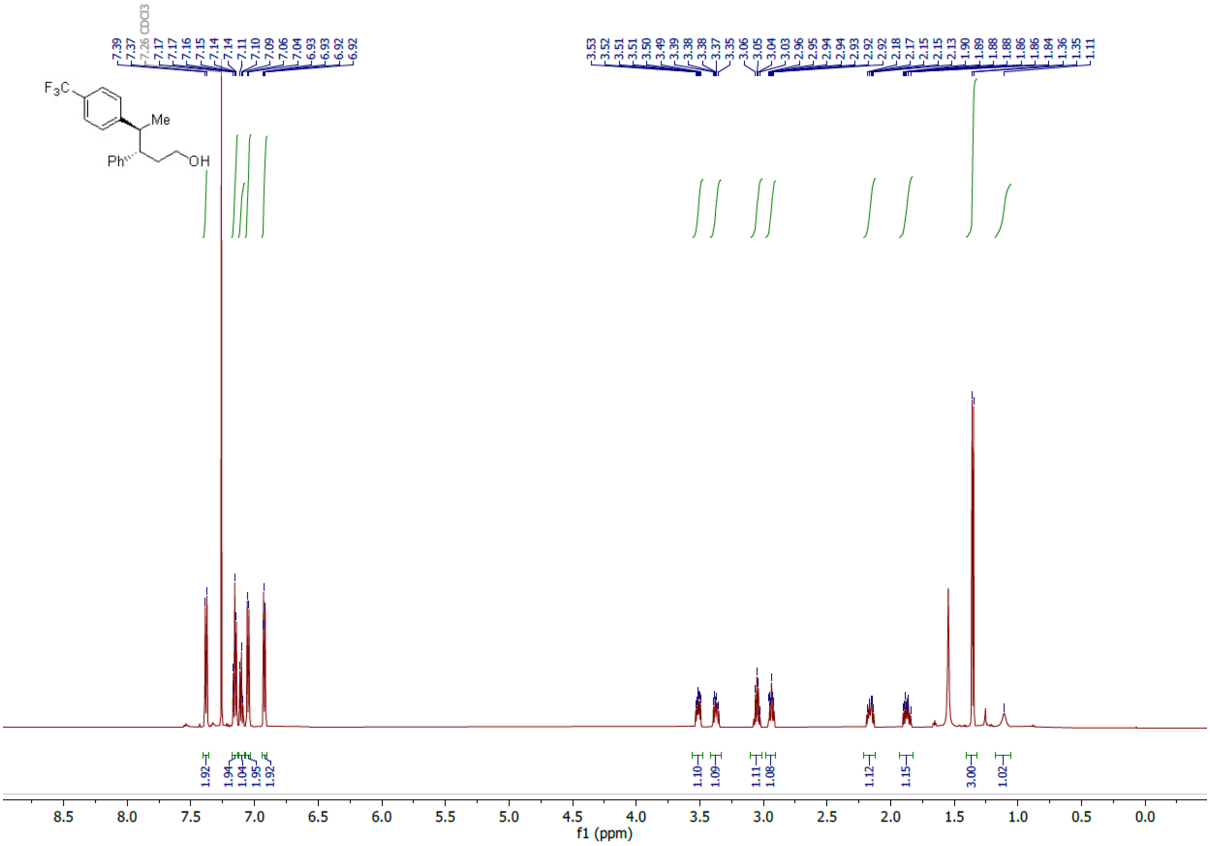

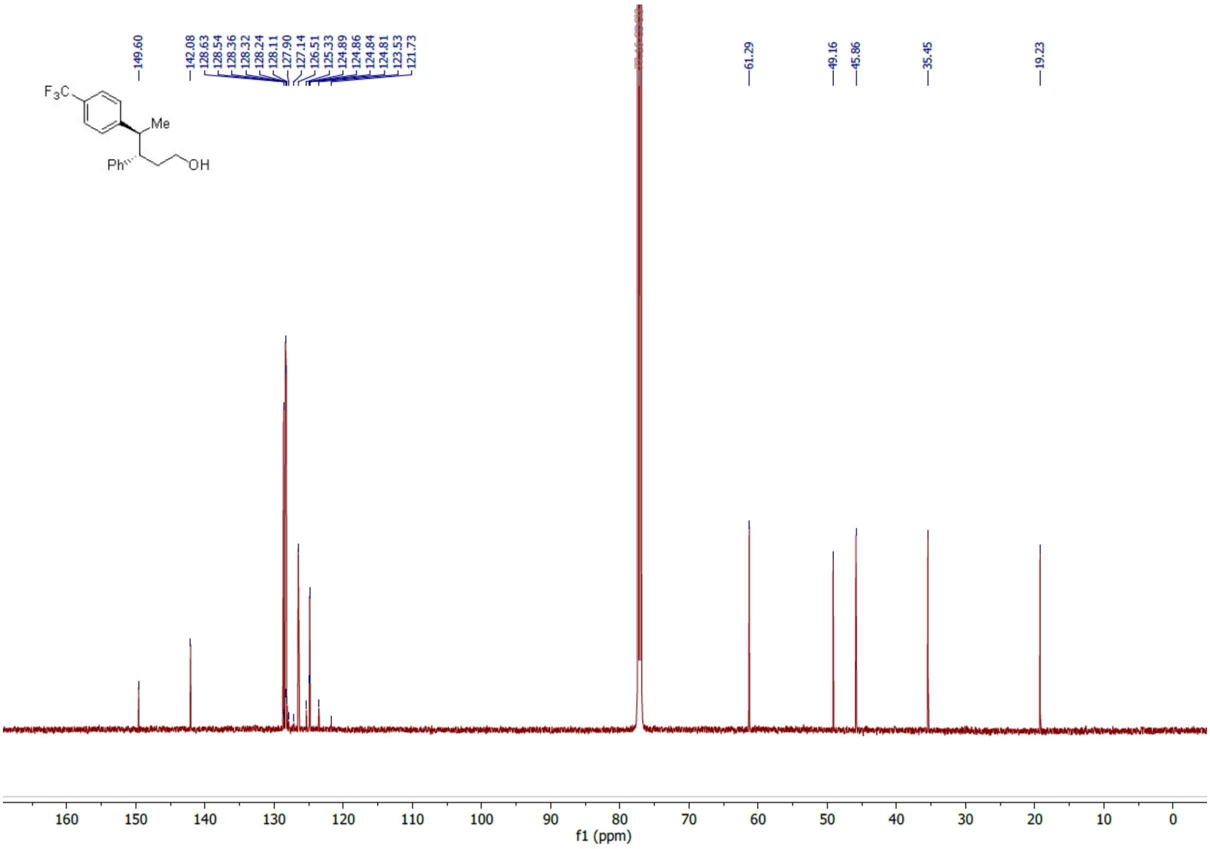

Compound **31**

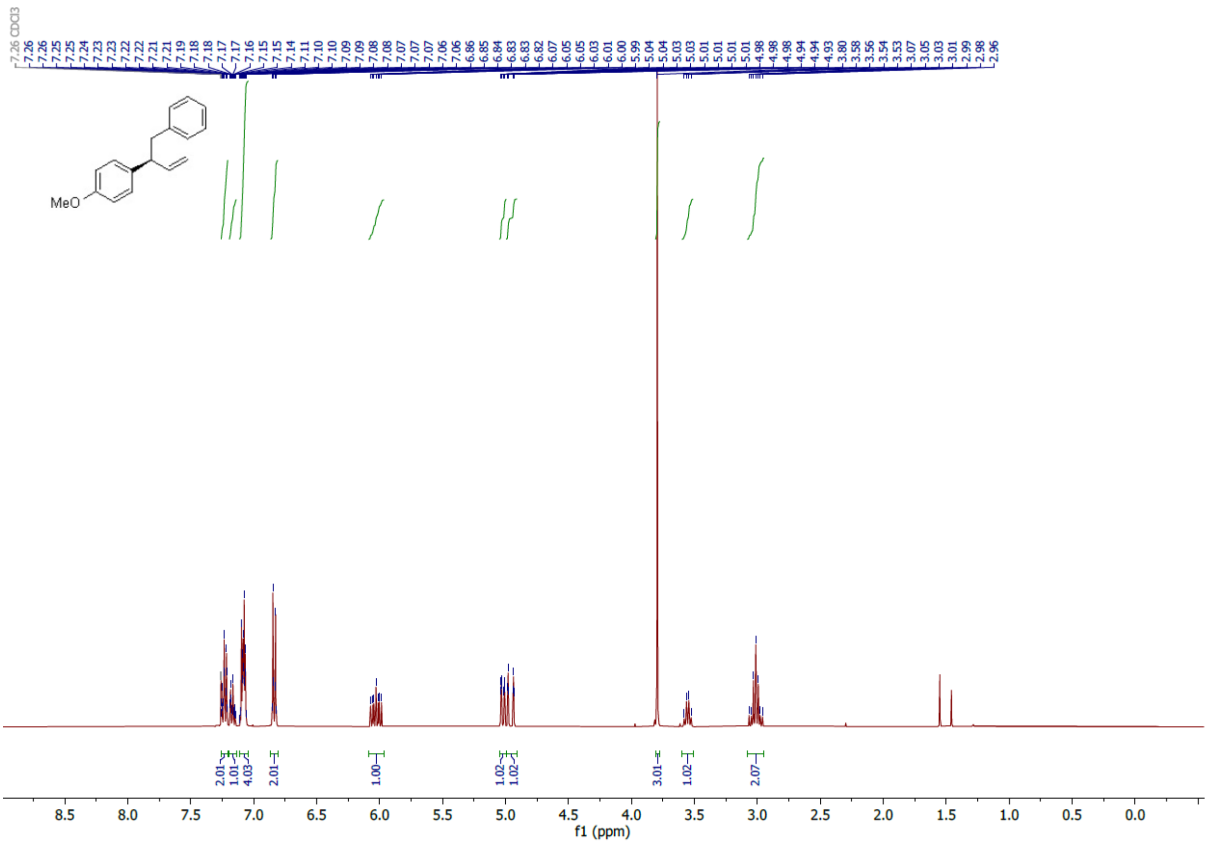

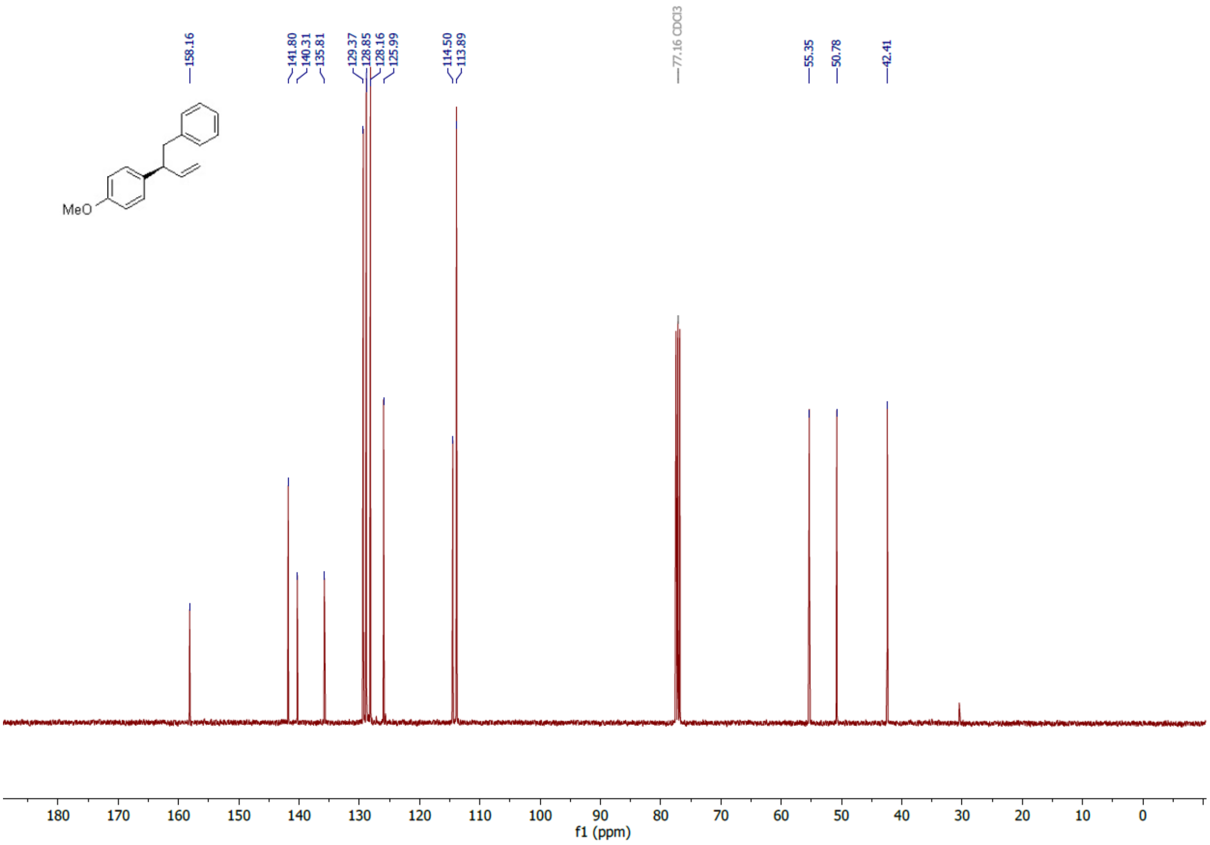

Compound **32**

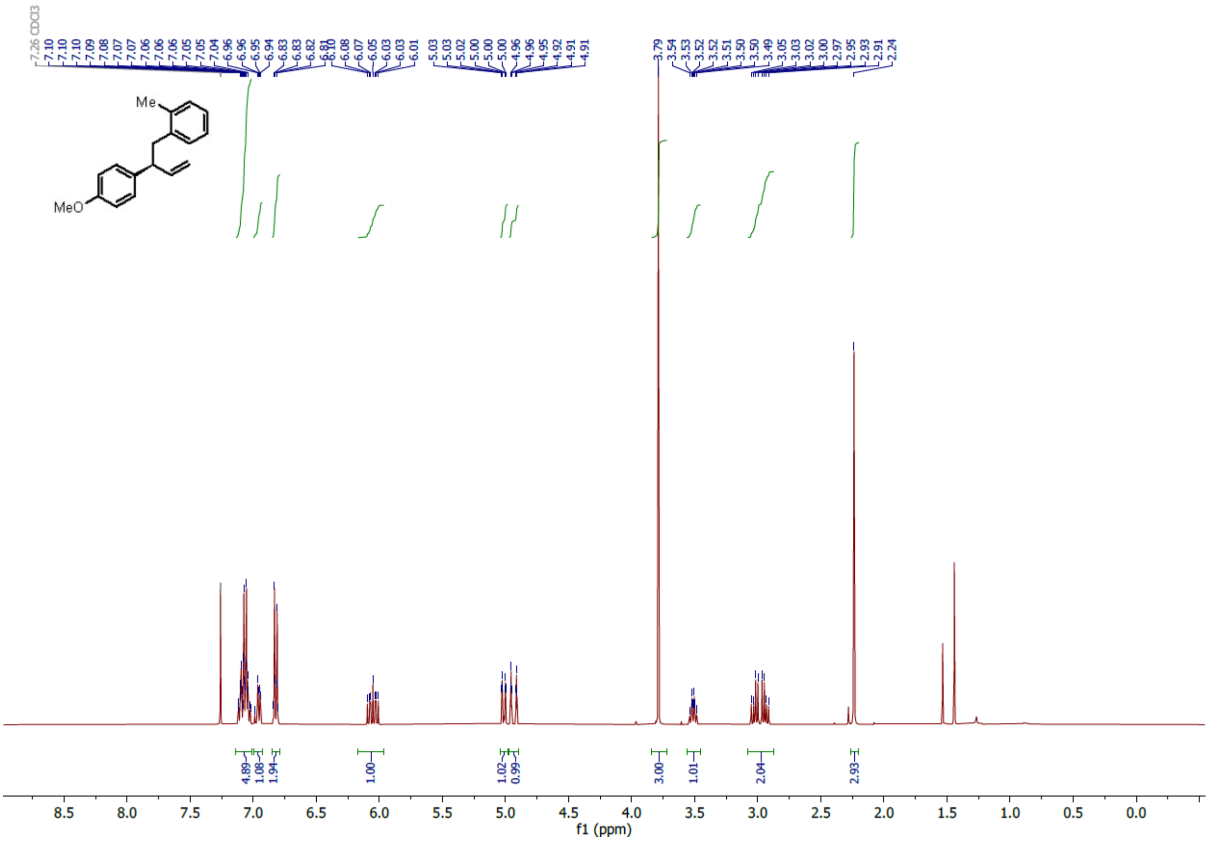

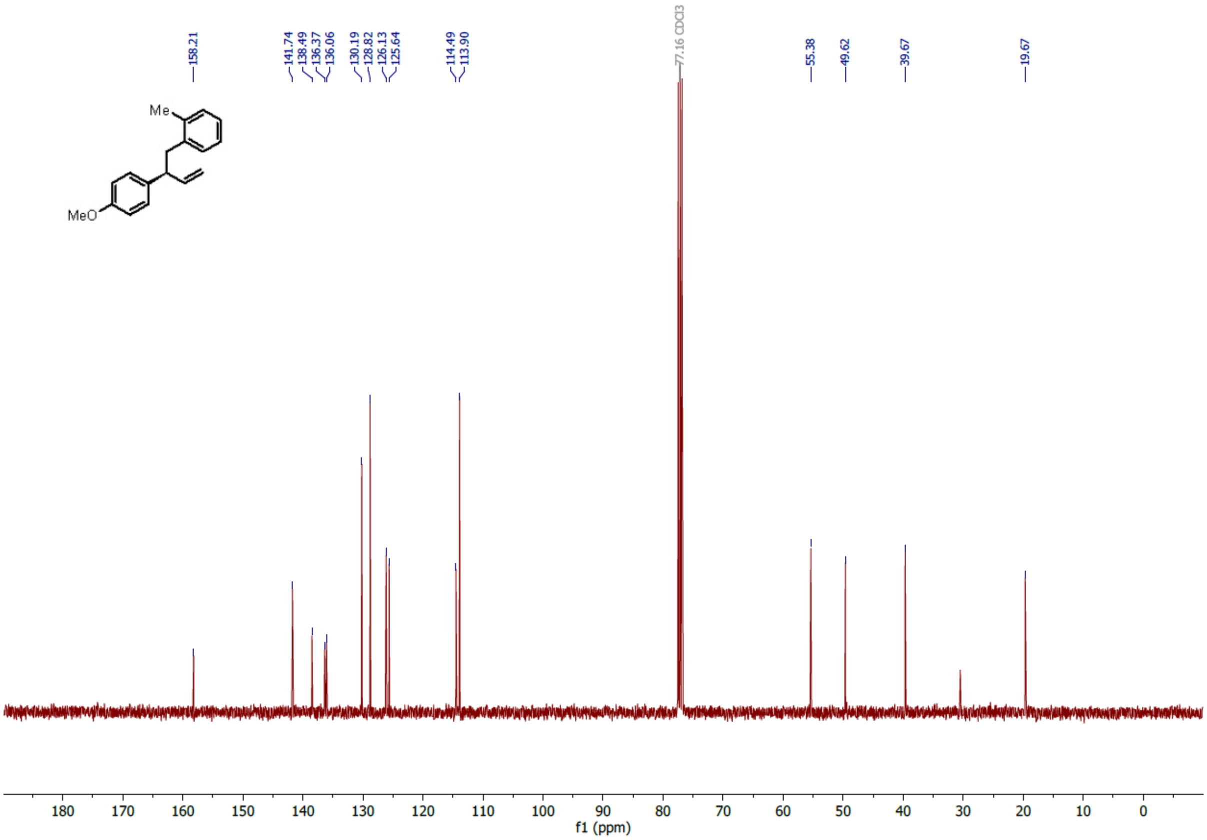

Compound **32'**

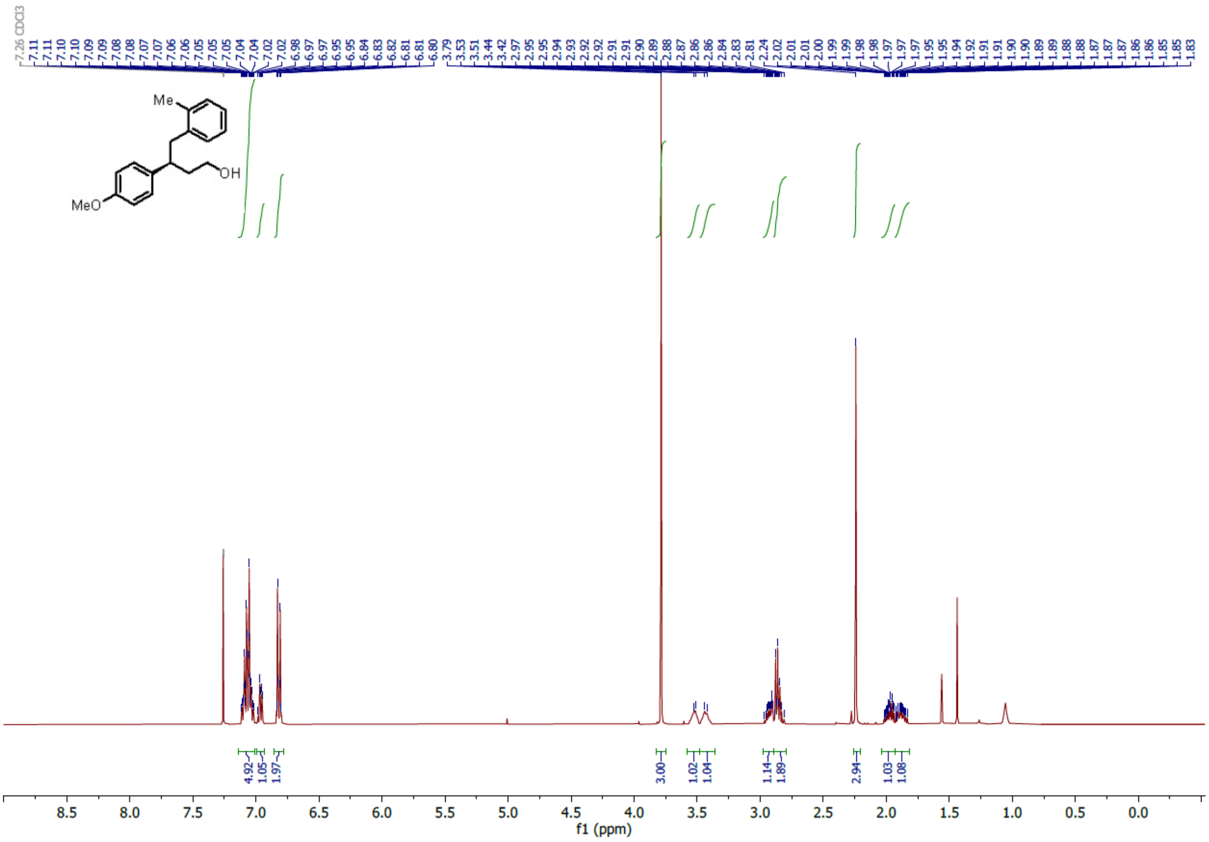

7.26 CDCl3
7.11
7.11
7.10
7.10
7.09
7.09
7.08
7.08
7.07
7.07
7.06
7.06
7.05
7.05
7.05
7.04
7.04
7.02
7.02
6.98
6.97
6.97
6.95
6.95
6.84
6.83
6.82
6.81
6.81
6.80
3.79
3.53
3.51
3.44
3.42
2.97
2.95
2.95
2.94
2.93
2.92
2.92
2.91
2.91
2.90
2.89
2.88
2.87
2.86
2.86
2.84
2.83
2.81
2.24
2.02
2.01
2.01
2.00
1.99
1.99
1.98
1.98
1.97
1.97
1.97
1.95
1.95
1.94
1.92
1.91
1.91
1.90
1.90
1.89
1.89
1.88
1.88
1.87
1.87
1.86
1.86
1.85
1.85
1.83
Me
OH
MeO
4.92
1.05
1.97
3.00
1.02
1.04
1.14
1.89
2.94
1.03
1.08
8.5
8.0
7.5
7.0
6.5
6.0
5.5
5.0
4.5
4.0
3.5
3.0
2.5
2.0
1.5
1.0
0.5
0.0
f1 (ppm)

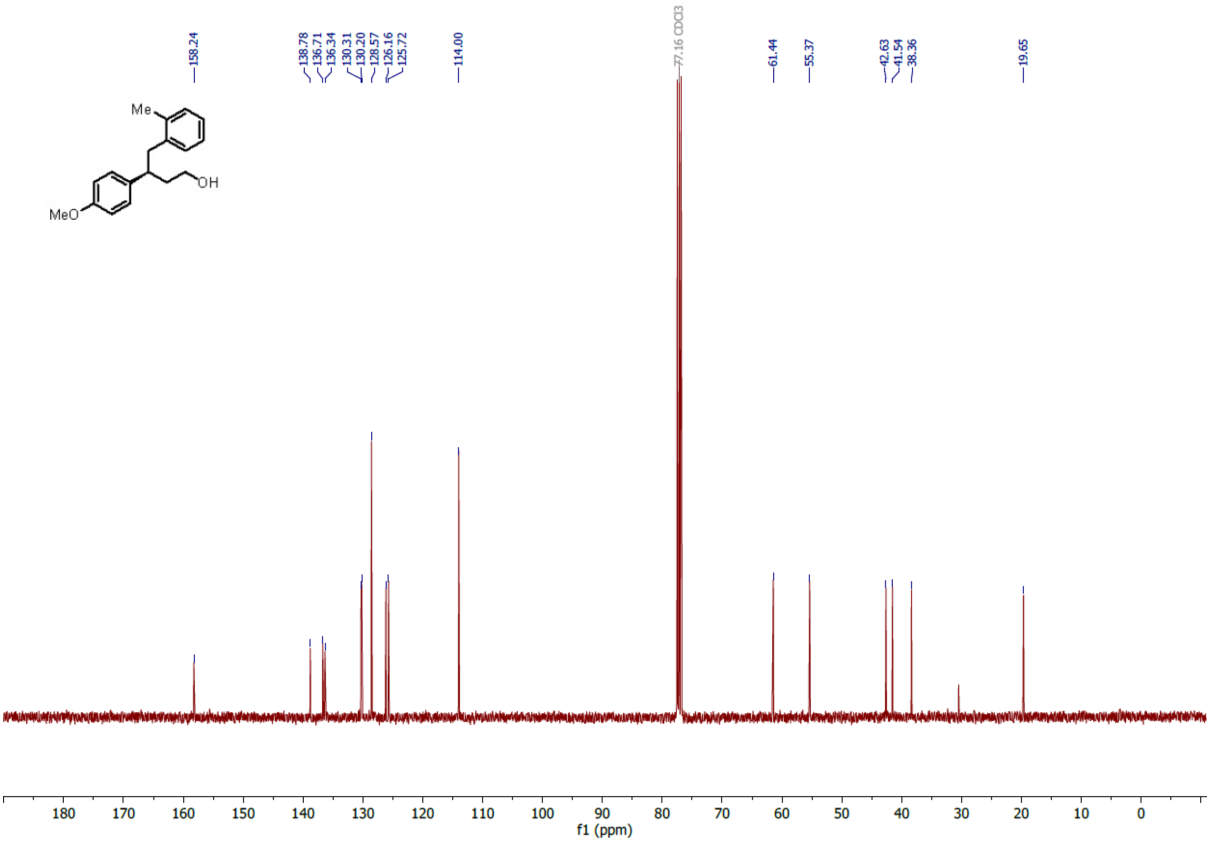

158.24
138.78
136.71
136.34
130.31
130.20
128.57
126.16
125.72
114.00
77.16 CDCl3
61.44
55.37
42.63
41.54
38.36
19.65
Me
OH
MeO
180
170
160
150
140
130
120
110
100
90
80
70
60
50
40
30
20
10
0
f1 (ppm)

Compound **33**

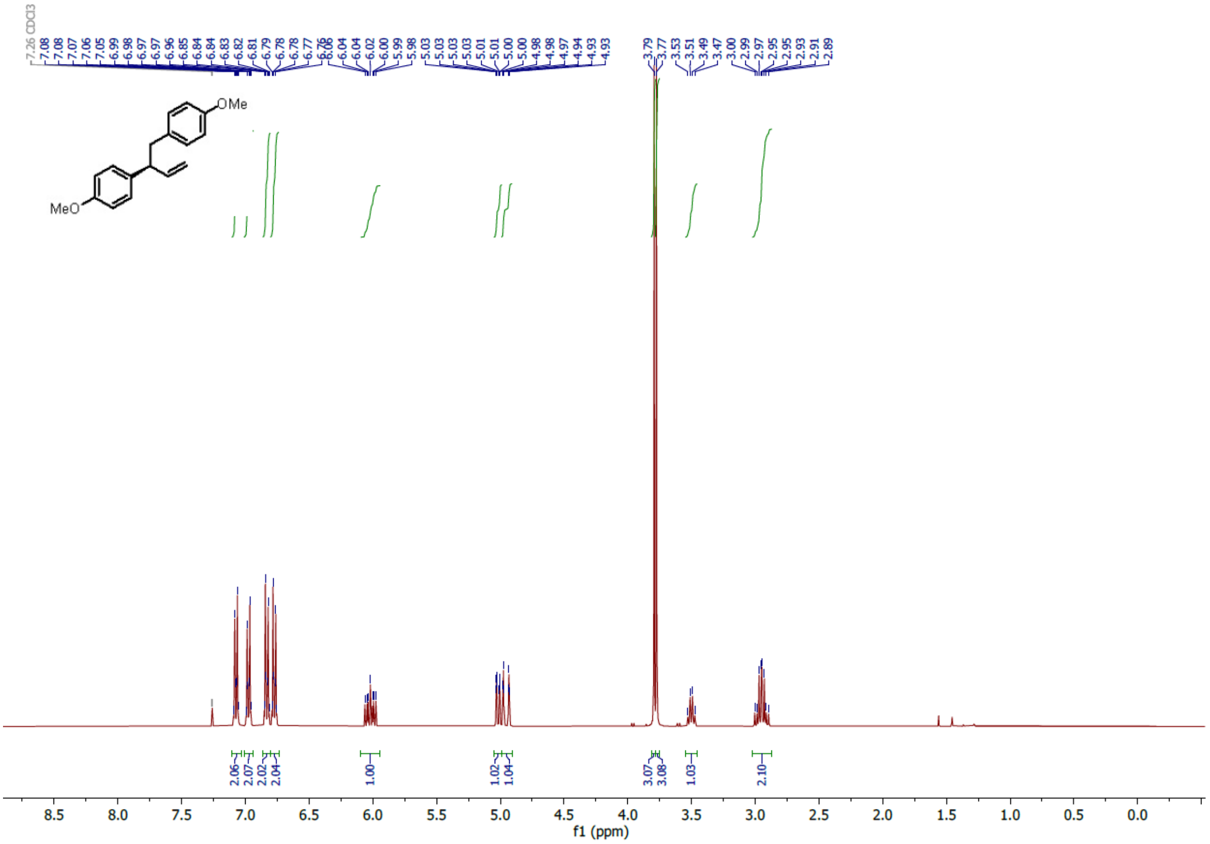

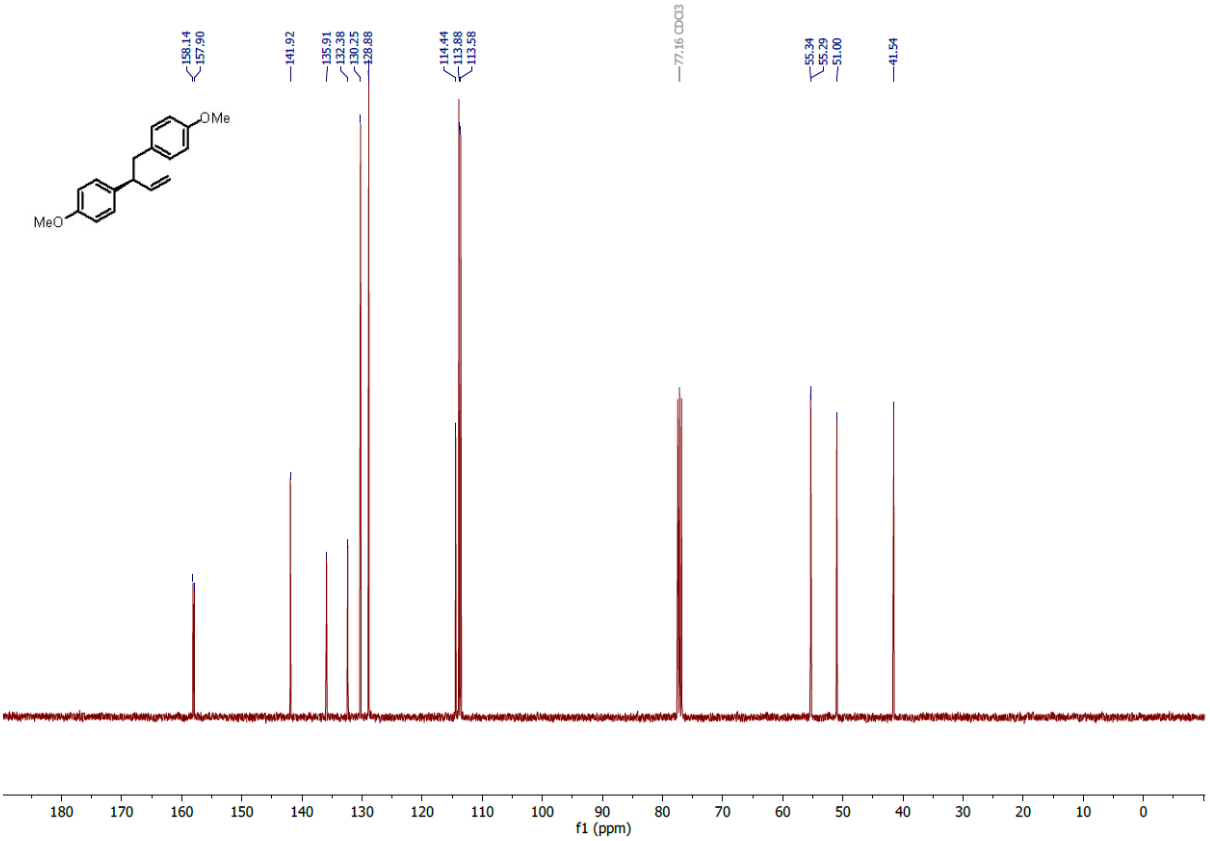

Compound **34**

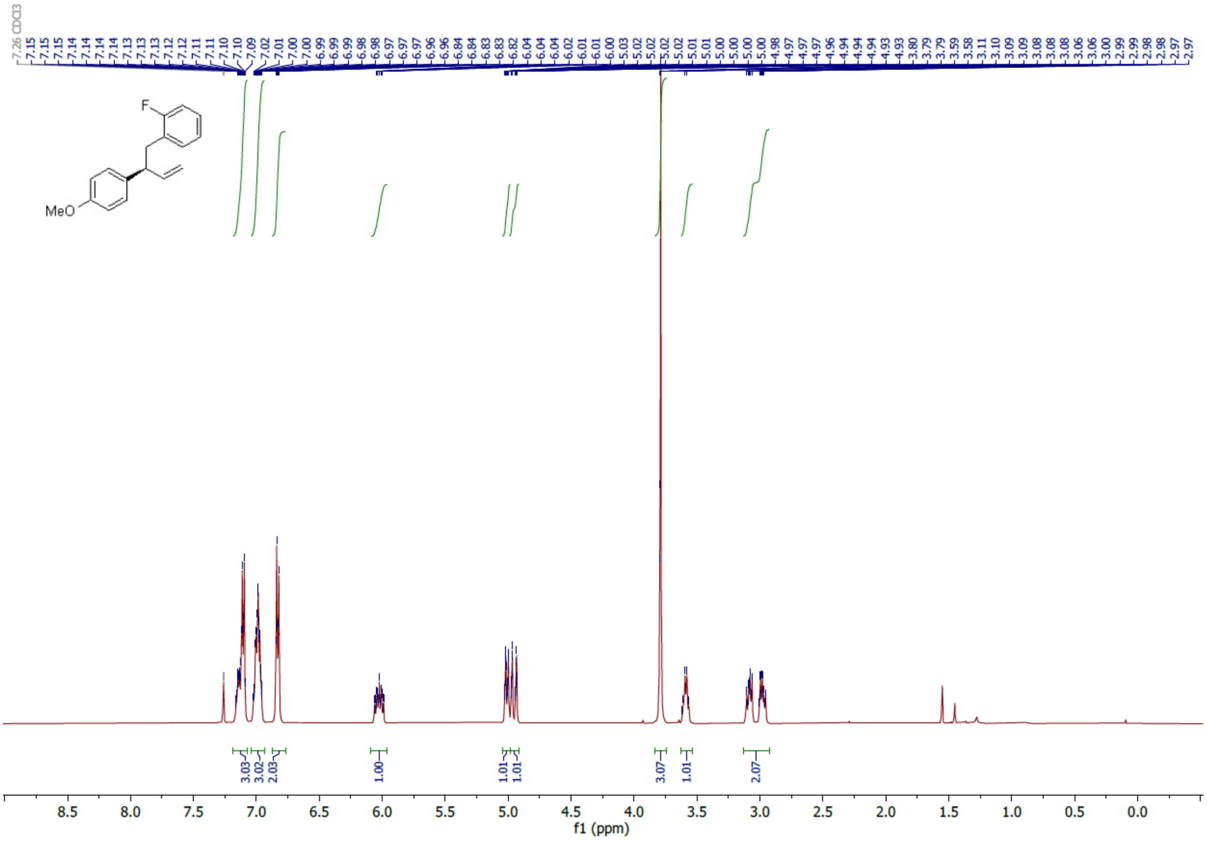
F
MeO
f1 (ppm)
3.03
3.02
2.03
1.00
1.01
1.01
3.07
1.01
2.07

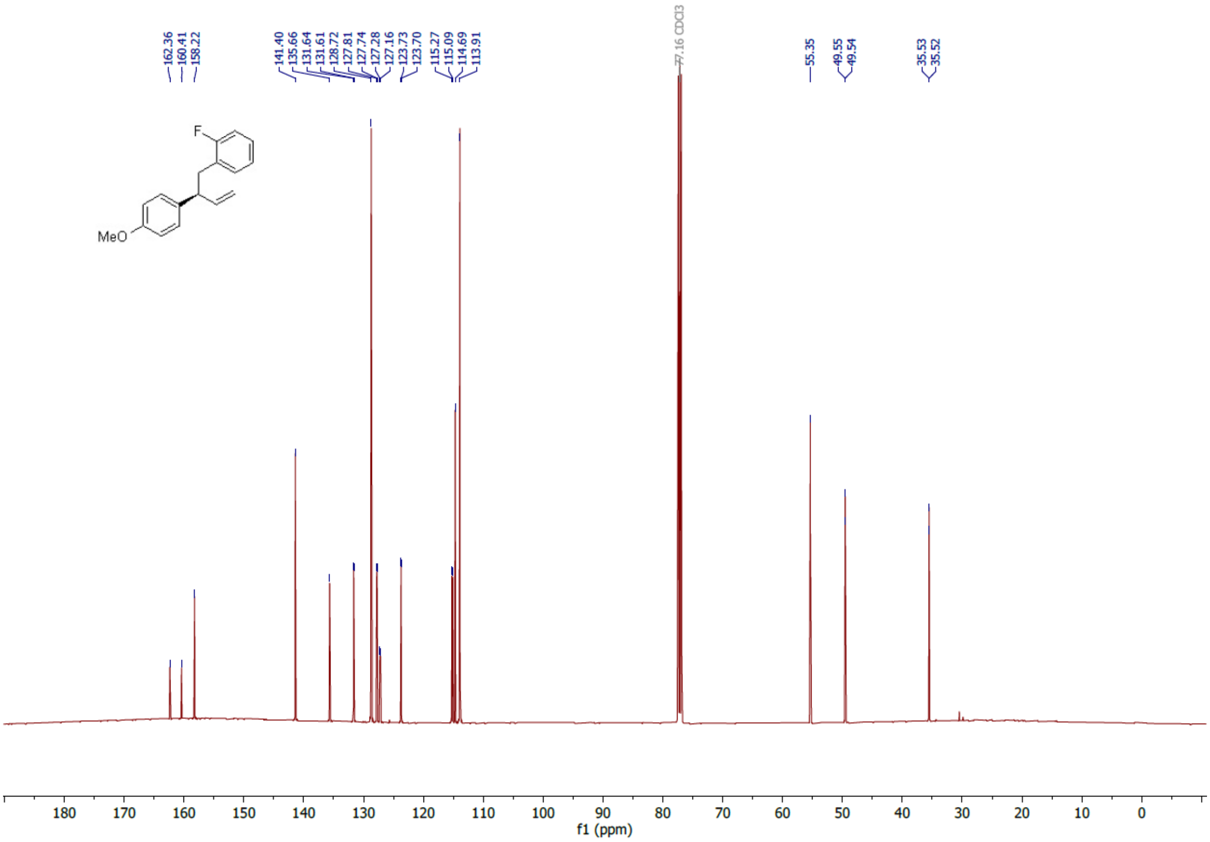
162.36
160.41
158.22
141.40
135.66
131.64
131.61
128.72
127.81
127.74
127.28
127.16
123.73
123.70
115.27
115.09
114.69
113.91
77.16 CDCl3
55.35
49.55
49.54
35.53
35.52
F
MeO
f1 (ppm)

Compound **34'**

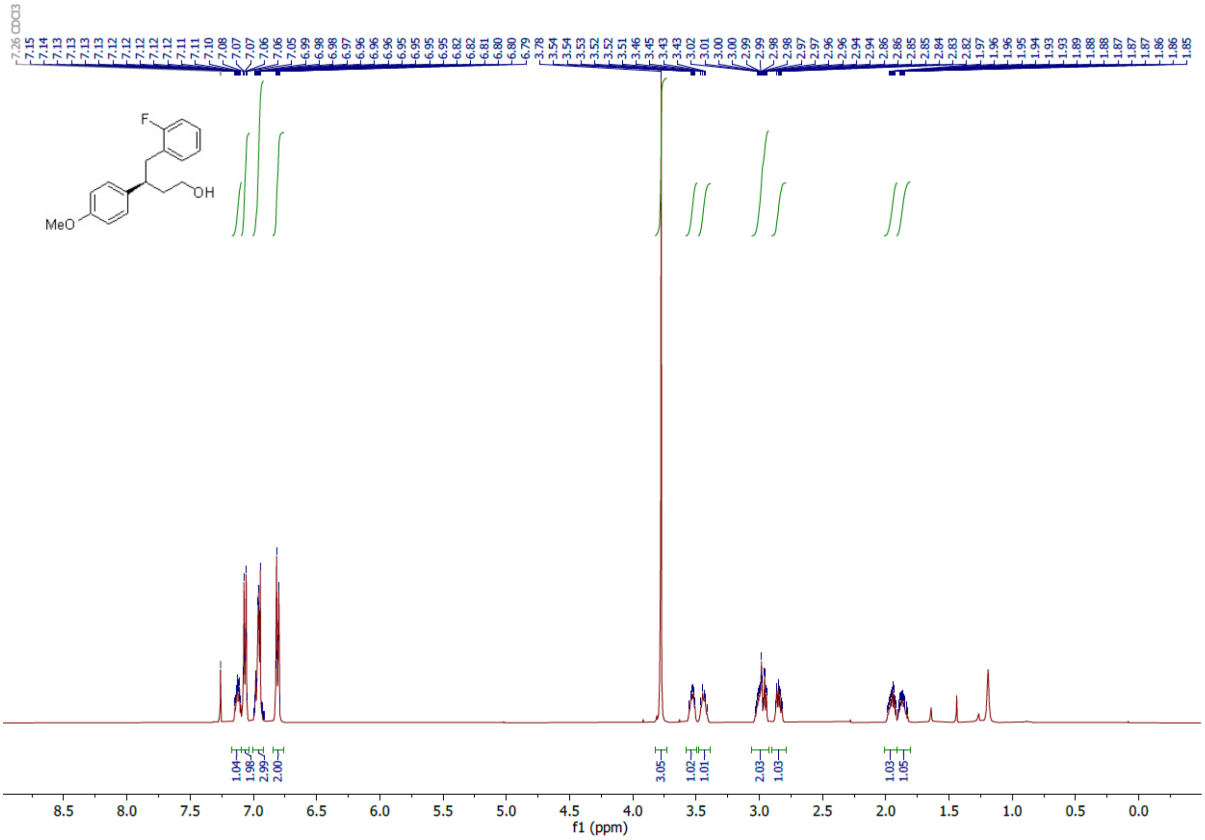

7.26 CDCl3
7.15 7.14 7.13 7.13 7.13 7.13 7.12 7.12 7.12 7.12 7.11 7.11 7.10 7.08 7.07 7.07 7.06 7.06 7.05 6.99 6.98 6.98 6.97 6.96 6.96 6.96 6.95 6.95 6.95 6.95 6.82 6.81 6.80 6.80 6.79 3.78 3.54 3.54 3.53 3.52 3.52 3.51 3.46 3.45 3.45 3.43 3.43 3.02 3.01 3.00 3.00 2.99 2.99 2.98 2.98 2.97 2.97 2.96 2.96 2.94 2.94 2.86 2.86 2.85 2.85 2.84 2.83 2.82 1.97 1.96 1.96 1.95 1.94 1.93 1.93 1.89 1.88 1.88 1.87 1.87 1.87 1.86 1.86 1.85
F
OH
MeO
1.04 1.98 2.99 2.00
3.05
1.02 1.01
2.03 1.03
1.03 1.05
8.5 8.0 7.5 7.0 6.5 6.0 5.5 5.0 4.5 4.0 3.5 3.0 2.5 2.0 1.5 1.0 0.5 0.0
f1 (ppm)

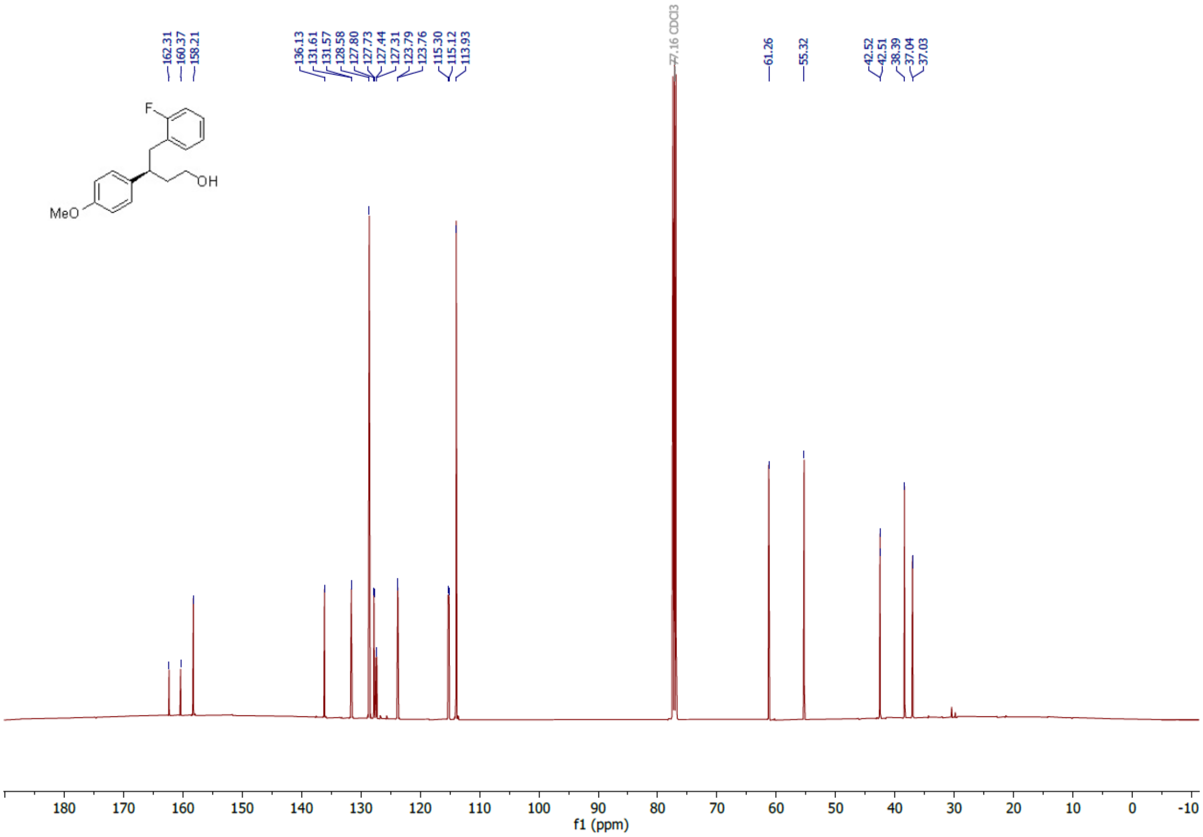

162.31 160.37 158.21
136.13 131.61 131.57 128.58 127.80 127.73 127.44 127.31 123.79 123.76 115.30 115.12 113.93
77.16 CDCl3
61.26
55.32
42.52 42.51 38.39 37.04 37.03
F
OH
MeO
180 170 160 150 140 130 120 110 100 90 80 70 60 50 40 30 20 10 0 -10
f1 (ppm)

Compound **S3**

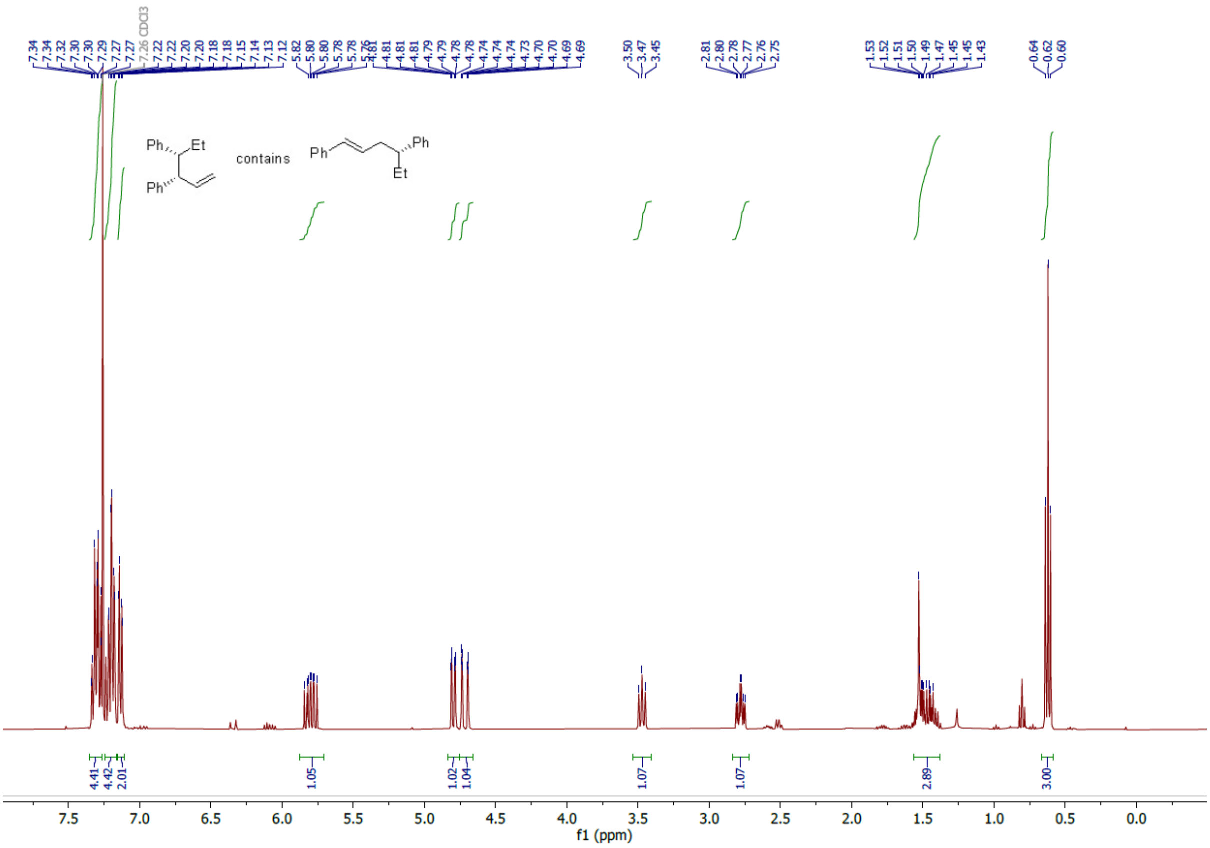

Ph Et Ph
contains
Ph Ph Et
7.34 7.34 7.32 7.30 7.30 7.29 7.27 7.27 7.26 CDCl3 7.22 7.22 7.20 7.20 7.18 7.18 7.15 7.14 7.13 7.12 5.82 5.80 5.80 5.78 5.78 5.76 4.81 4.81 4.81 4.79 4.79 4.78 4.78 4.74 4.74 4.74 4.73 4.70 4.69 4.69 3.50 3.47 3.45 2.81 2.80 2.78 2.77 2.76 2.75 1.53 1.52 1.51 1.50 1.49 1.47 1.45 1.45 1.43 0.64 0.62 0.60
4.41 4.42 2.01 1.05 1.02 1.04 1.07 1.07 2.89 3.00
7.5 7.0 6.5 6.0 5.5 5.0 4.5 4.0 3.5 3.0 2.5 2.0 1.5 1.0 0.5 0.0
f1 (ppm)

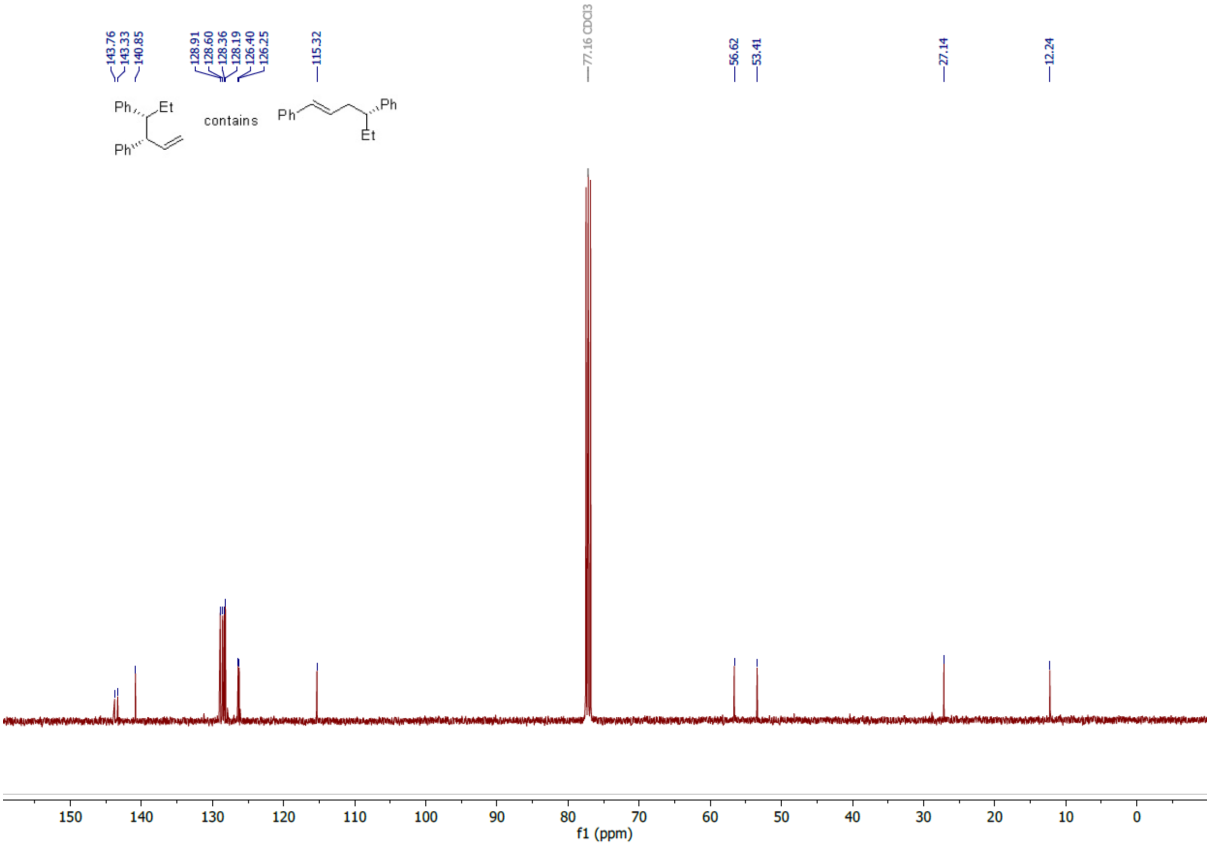

143.76 143.33 140.85 128.91 128.60 128.36 128.19 126.40 126.25 115.32 77.16 CDCl3 56.62 53.41 27.14 12.24
Ph Et Ph
contains
Ph Ph Et
150 140 130 120 110 100 90 80 70 60 50 40 30 20 10 0
f1 (ppm)

Compound **54'**

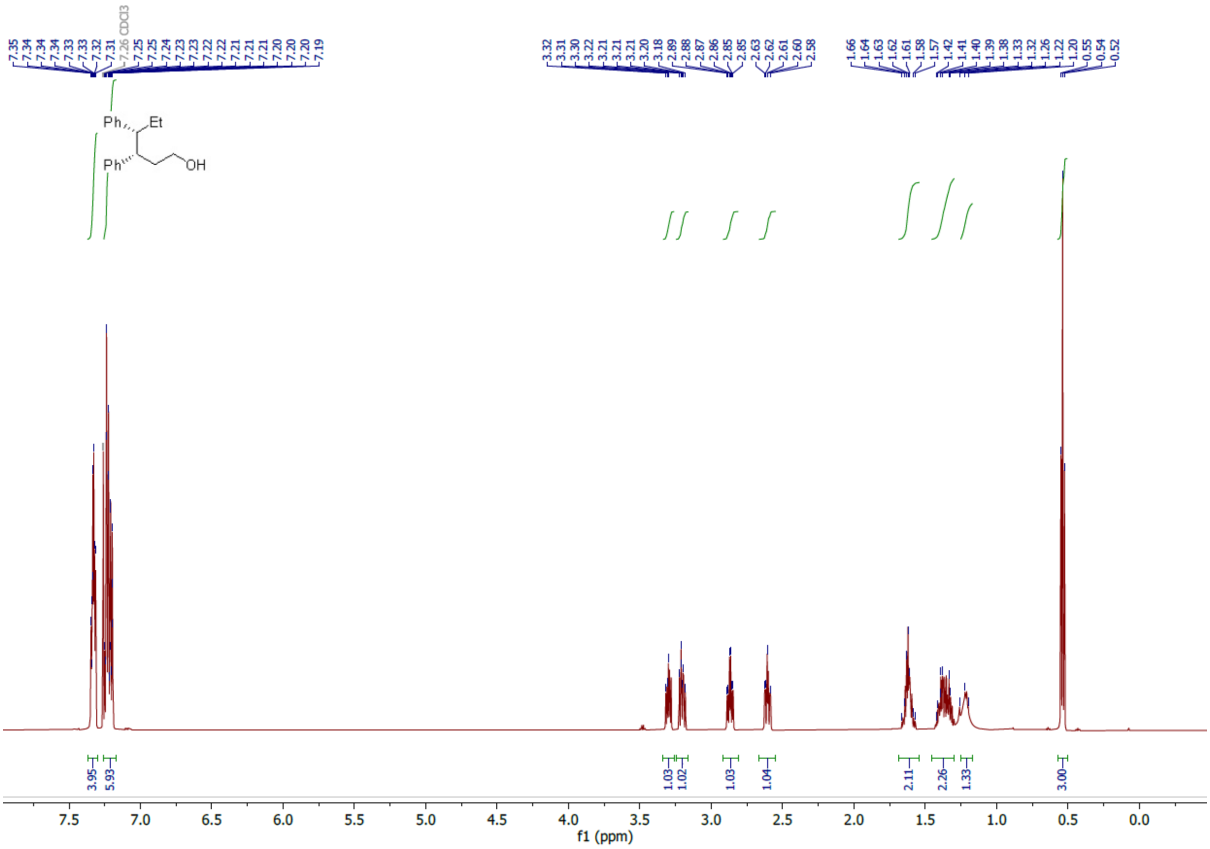

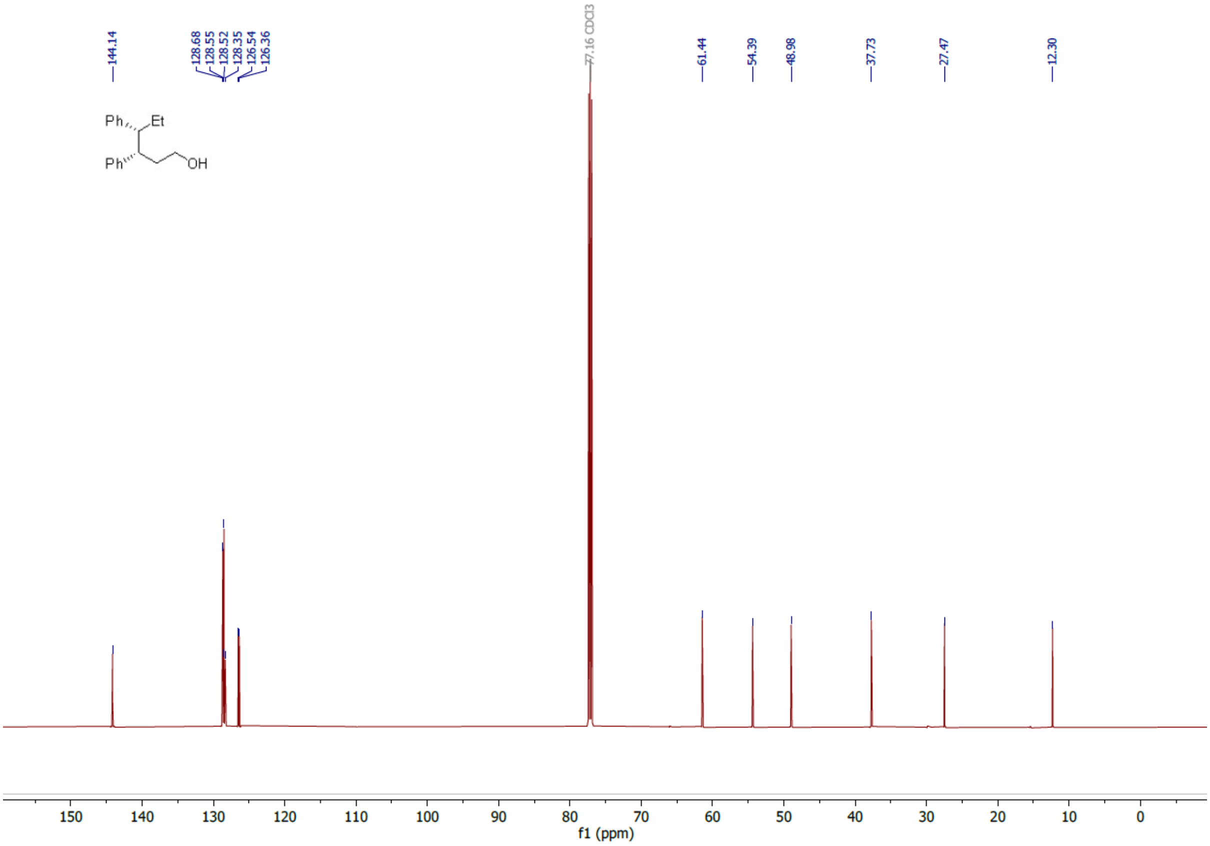

Compound **35**

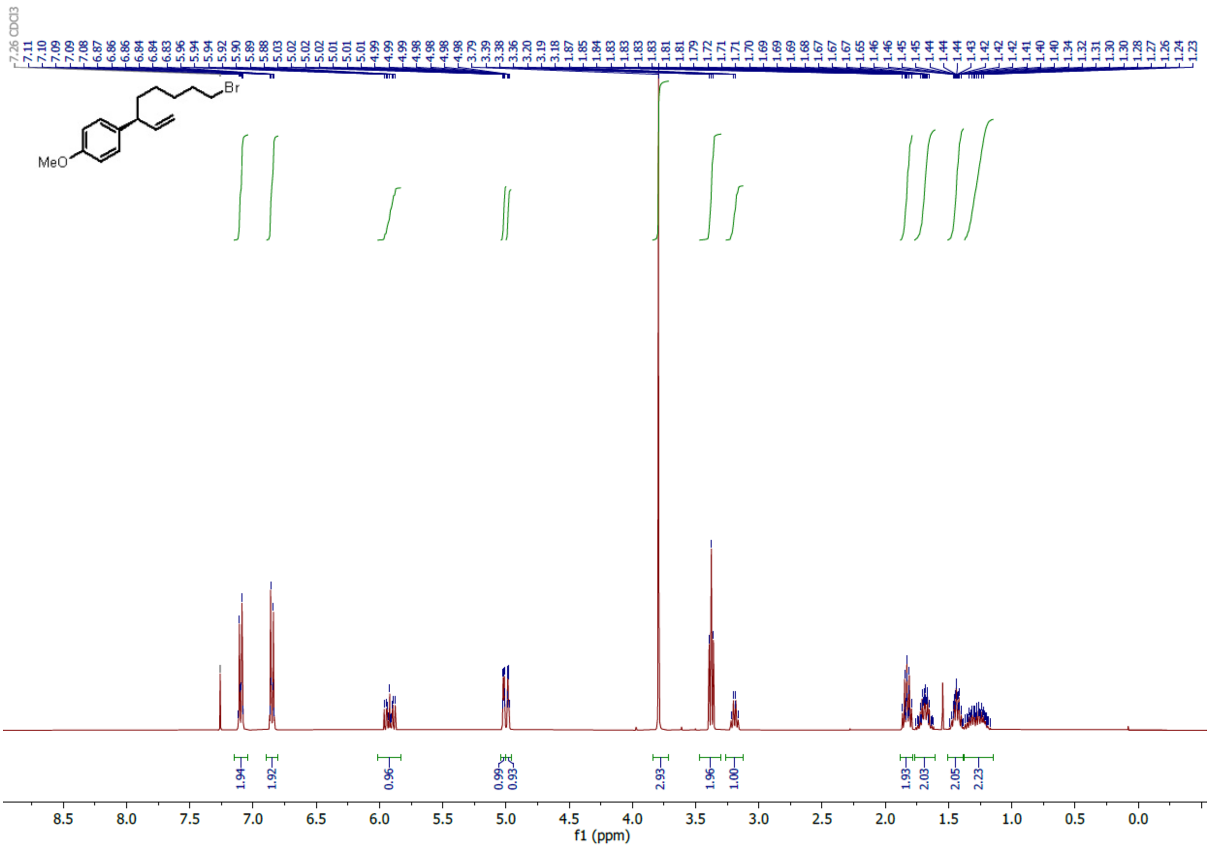

MeO
Br
f1 (ppm)

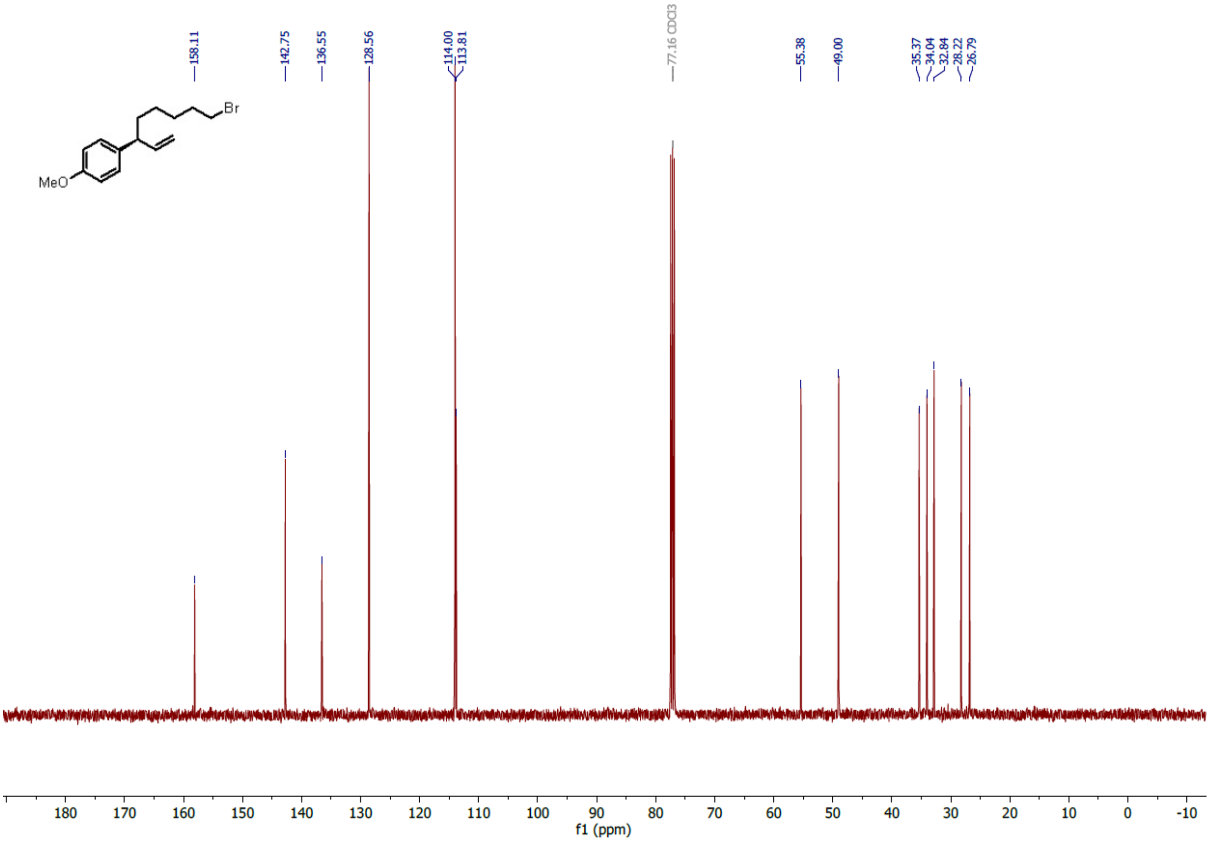

158.11
142.75
136.55
128.56
114.00
113.81
77.16 CDCl3
55.38
49.00
35.37
34.04
32.84
28.22
26.79
MeO
Br
f1 (ppm)

Compound **35'**

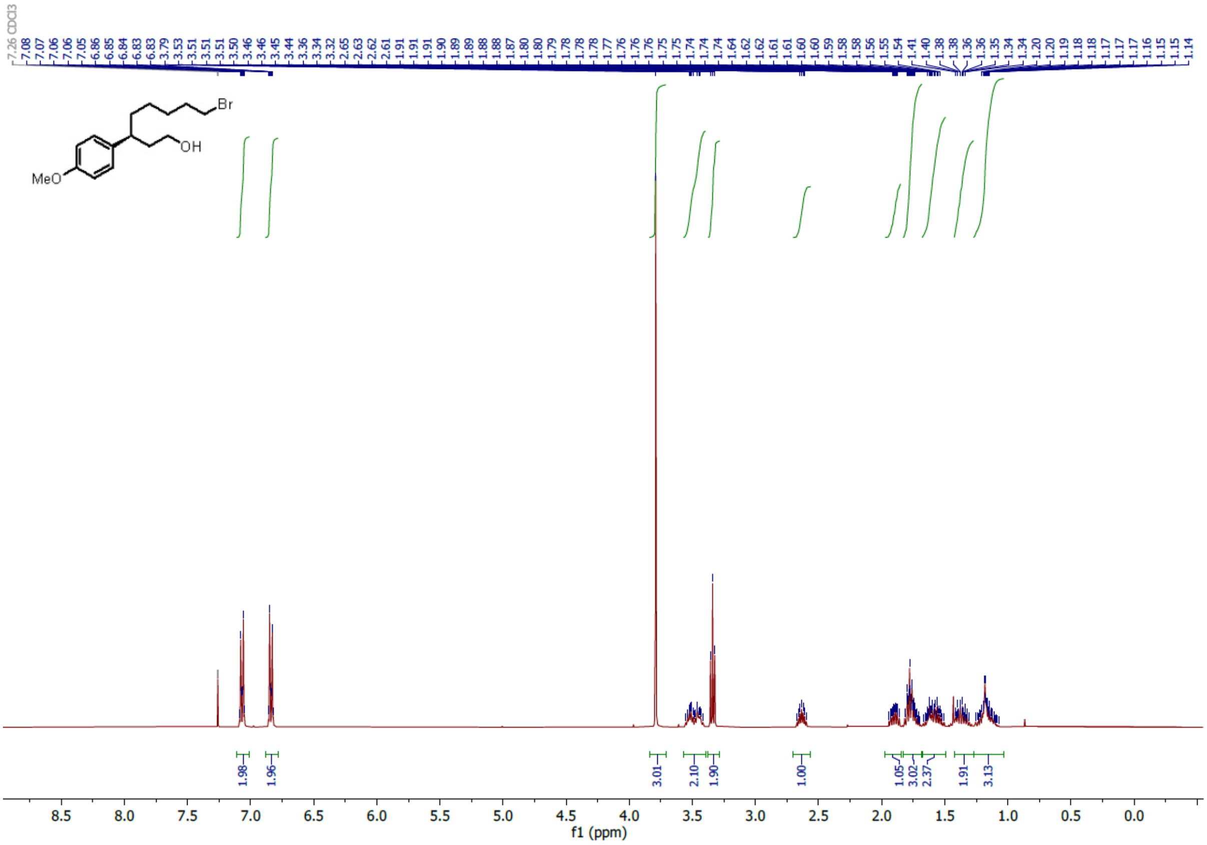

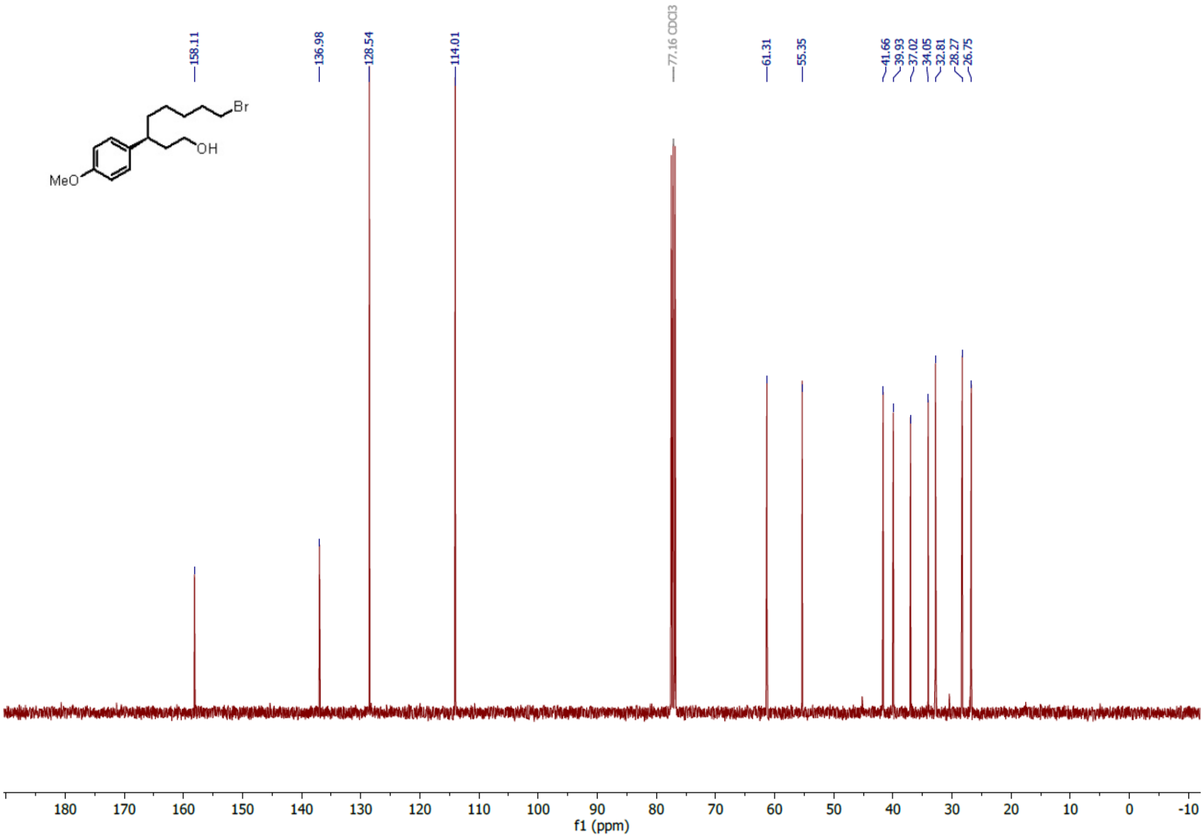

Compound **36**

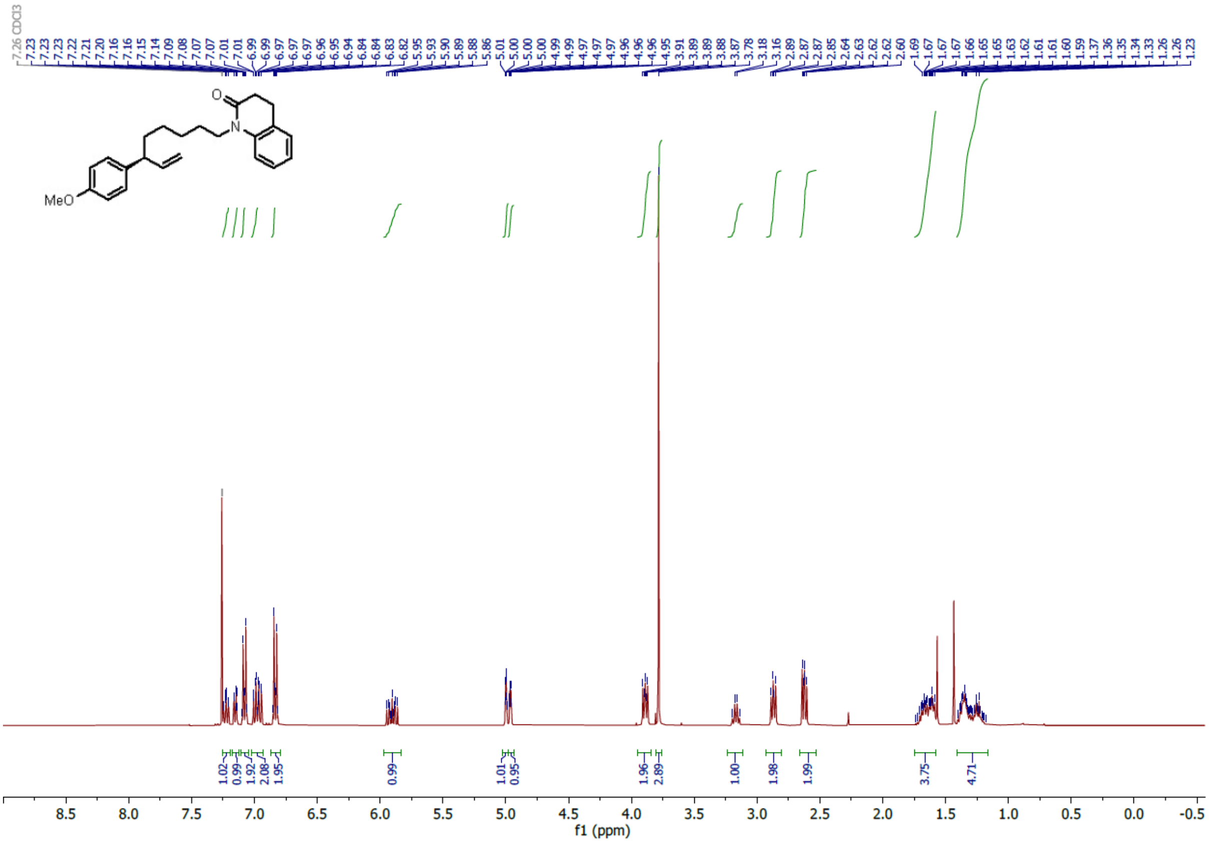
7.26 CDCl3
7.23 7.23 7.23 7.22 7.21 7.20 7.16 7.16 7.15 7.14 7.09 7.08 7.07 7.07 7.01 7.01 6.99 6.99 6.97 6.97 6.97 6.96 6.95 6.94 6.84 6.84 6.83 6.82 5.95 5.93 5.90 5.89 5.88 5.86 5.01 5.00 5.00 5.00 4.99 4.99 4.97 4.97 4.97 4.96 4.96 4.96 4.95 3.91 3.89 3.89 3.88 3.87 3.78 3.18 3.16 2.89 2.87 2.87 2.85 2.64 2.63 2.62 2.62 2.60 1.69 1.67 1.67 1.67 1.66 1.65 1.65 1.63 1.63 1.62 1.61 1.61 1.60 1.59 1.37 1.36 1.35 1.34 1.33 1.26 1.26 1.23
O
N
MeO
1.02 0.99 1.92 2.08 1.95 0.99 1.01 0.95 1.96 2.89 1.00 1.98 1.99 3.75 4.71
8.5 8.0 7.5 7.0 6.5 6.0 5.5 5.0 4.5 4.0 3.5 3.0 2.5 2.0 1.5 1.0 0.5 0.0 -0.5
f1 (ppm)

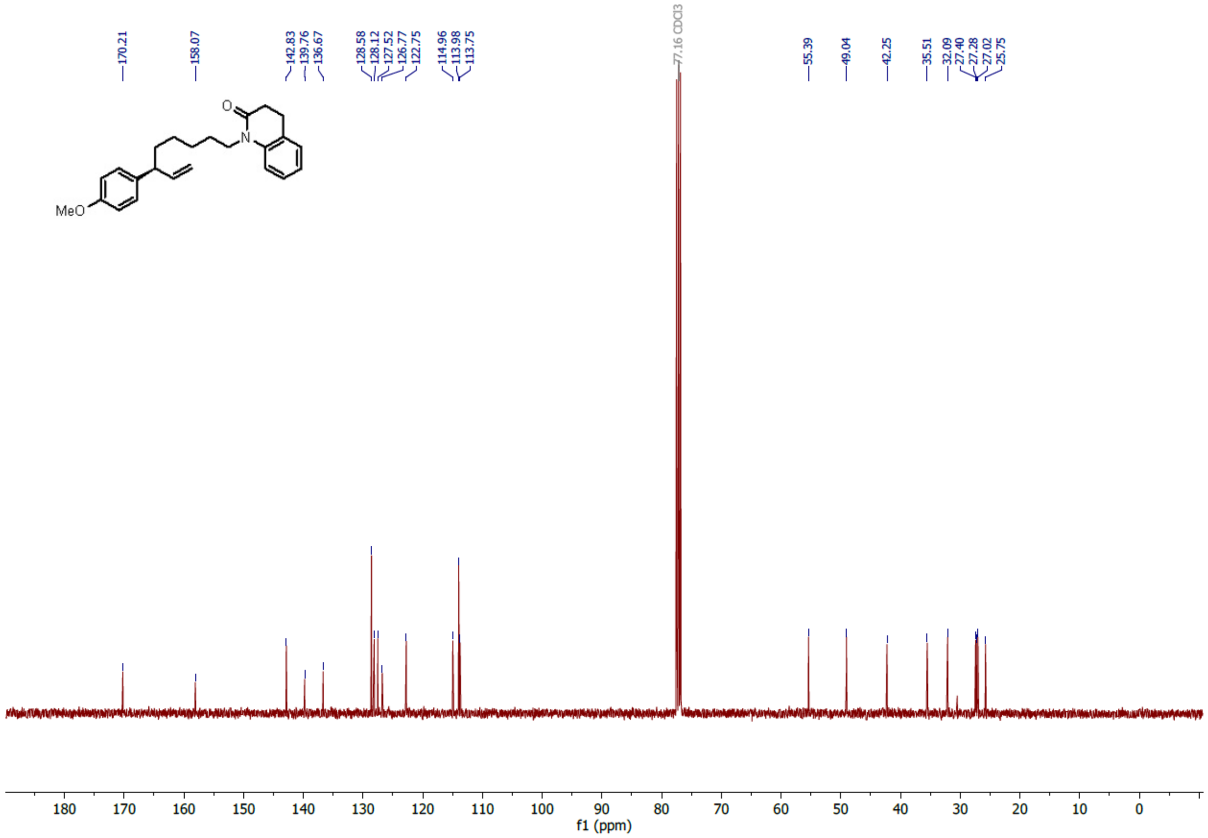
170.21
158.07
142.83 139.76 136.67
128.58 128.12 127.52 126.77 122.75
114.96 113.98 113.75
77.16 CDCl3
55.39
49.04
42.25
35.51 32.09 27.40 27.28 27.02 25.75
O
N
MeO
180 170 160 150 140 130 120 110 100 90 80 70 60 50 40 30 20 10 0
f1 (ppm)

Compound **37**

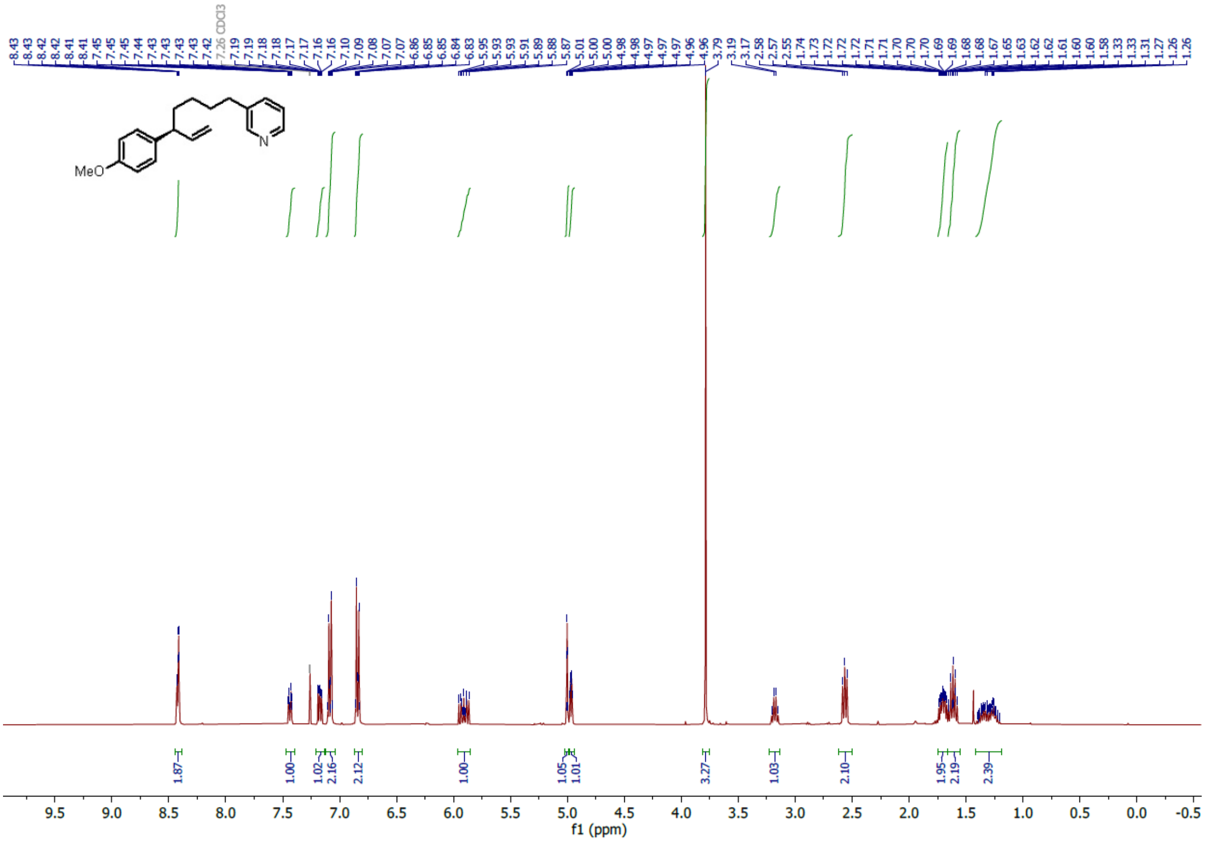

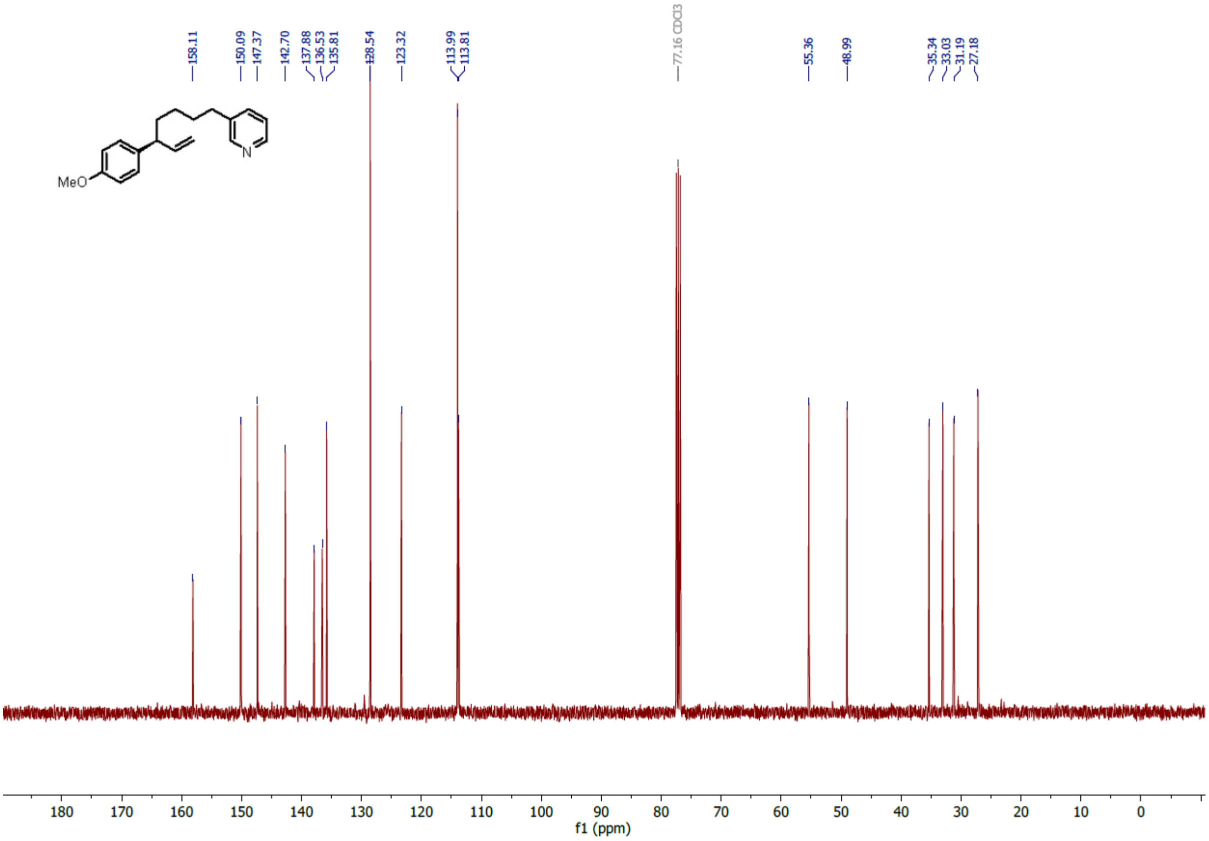

Compound **38**

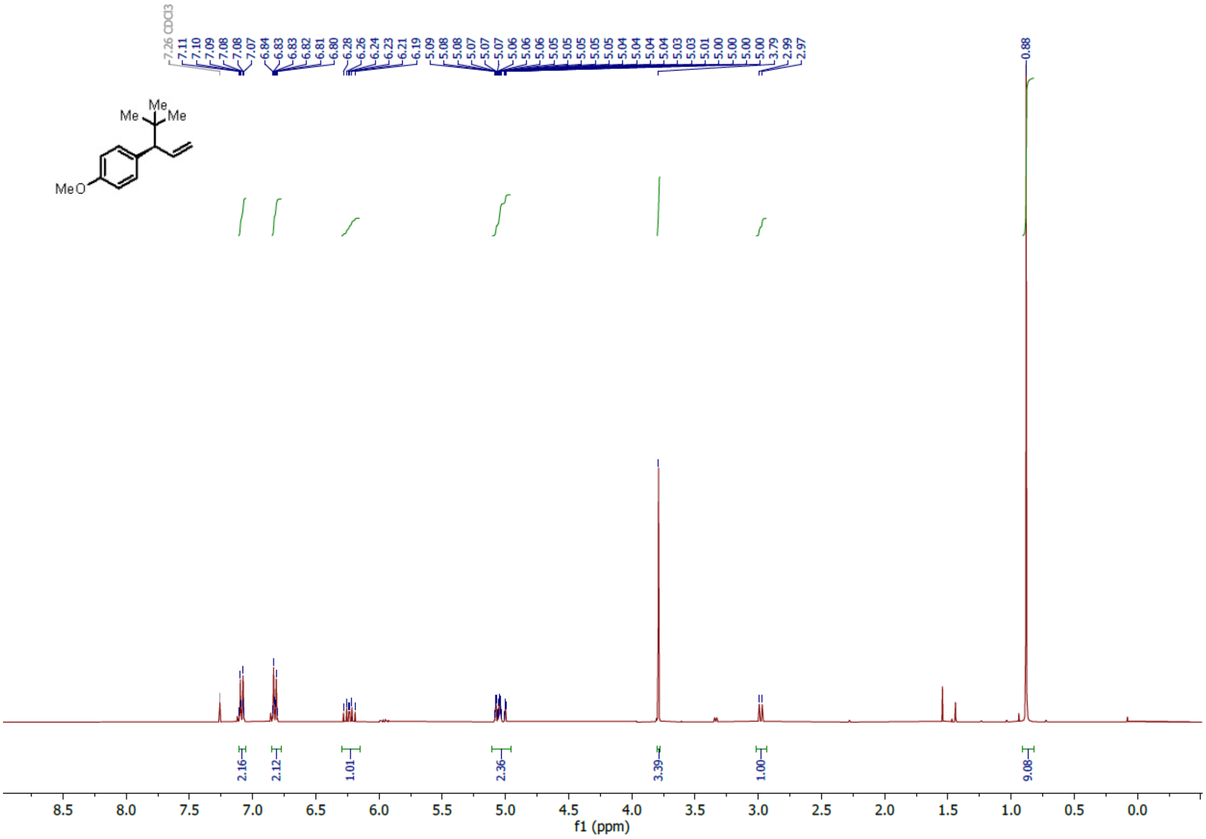

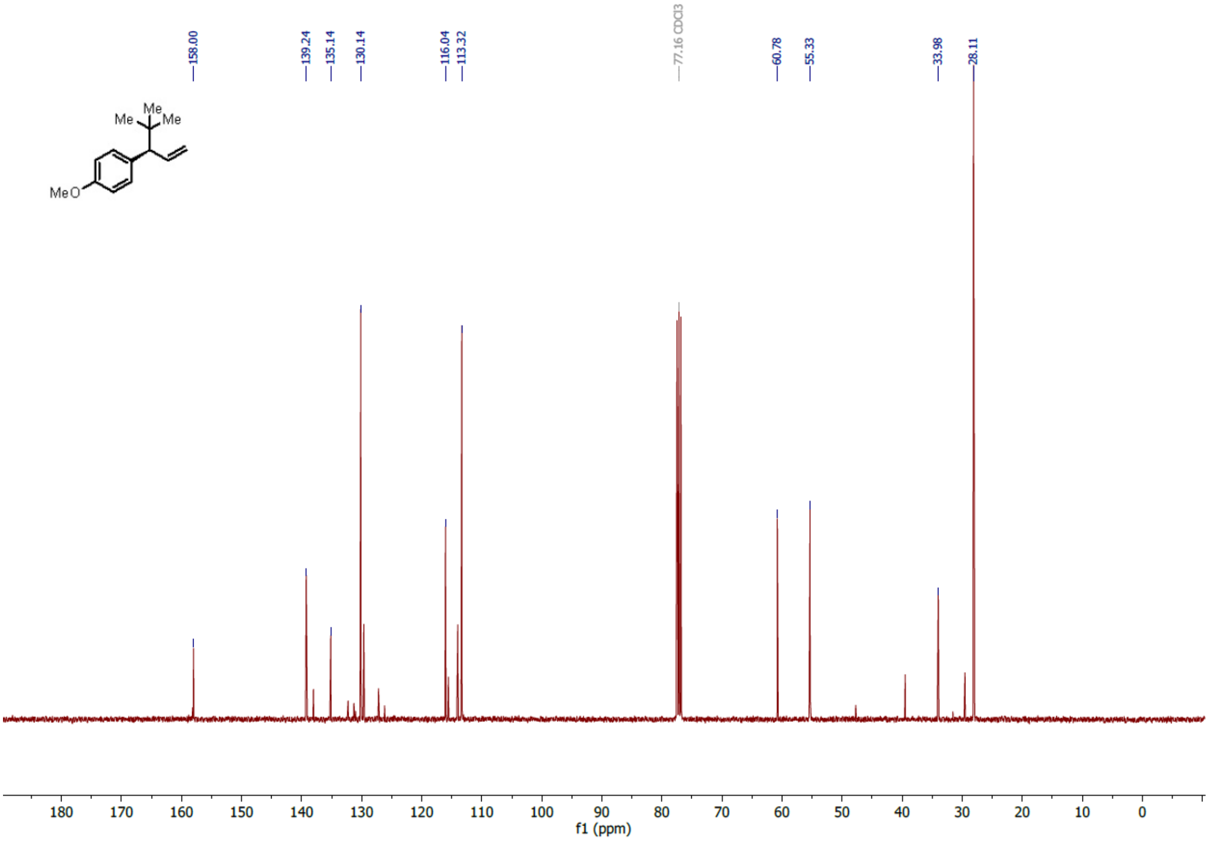

Compound **38'**

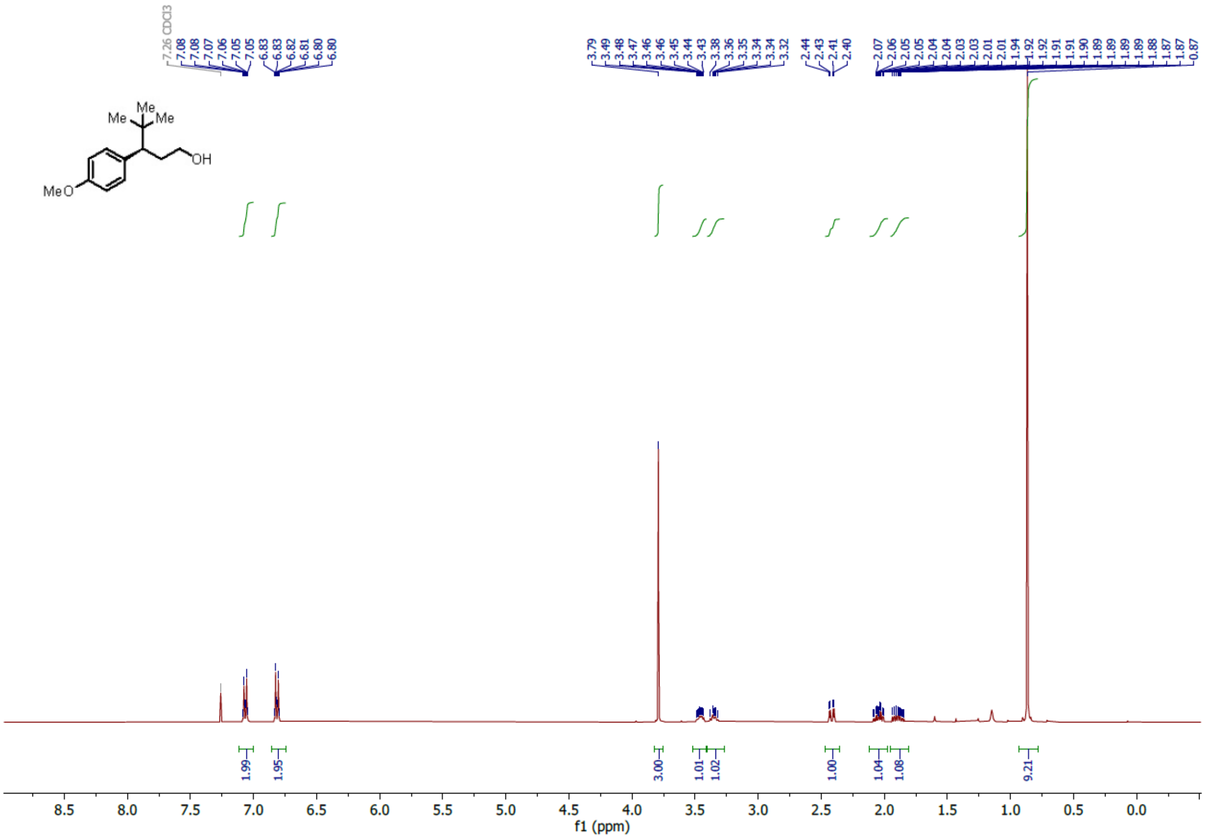

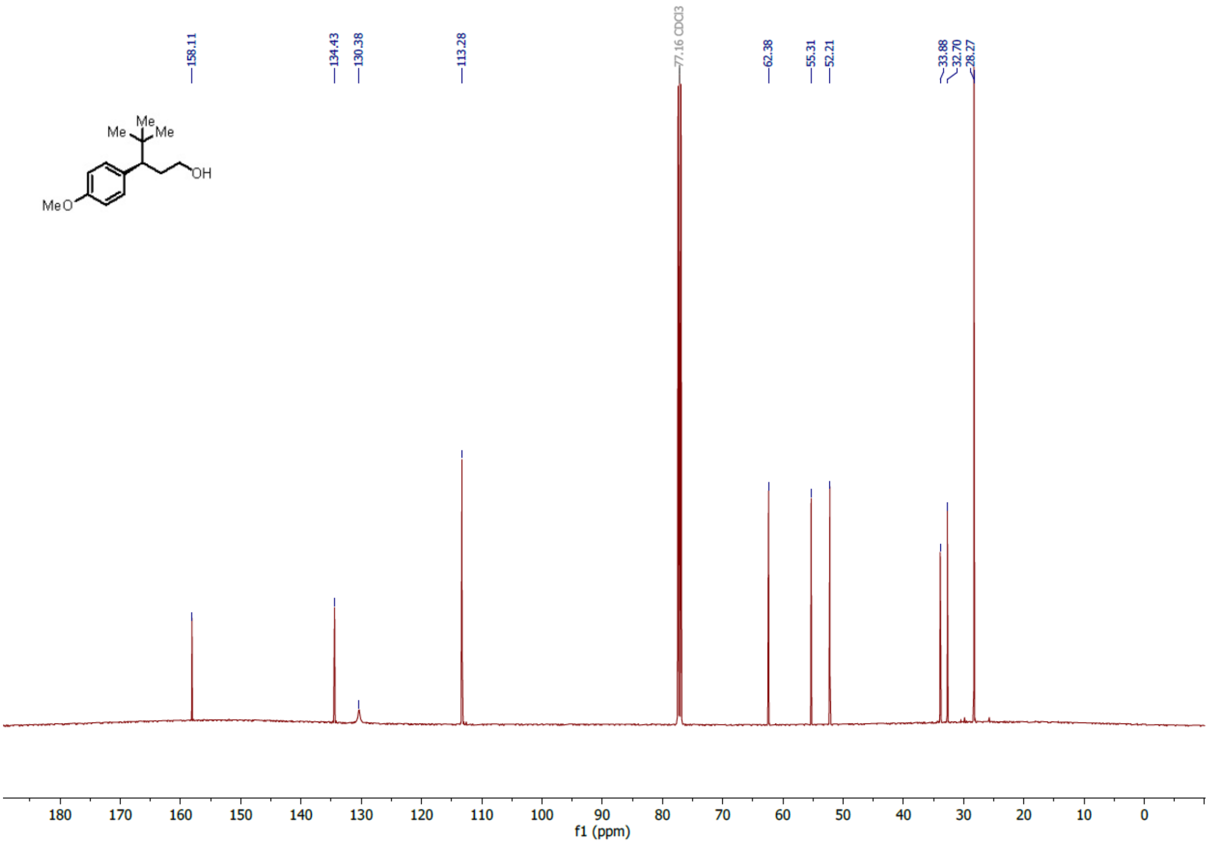

Compound **39**

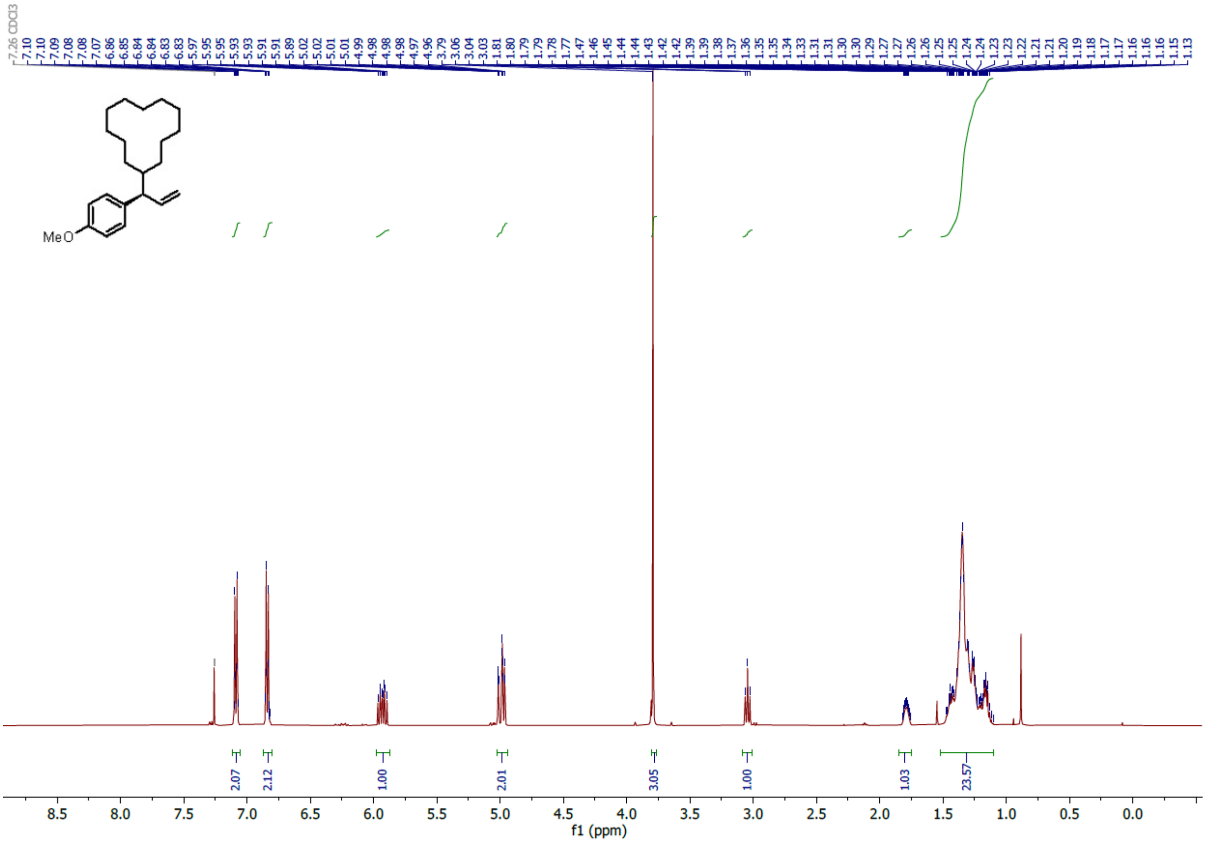

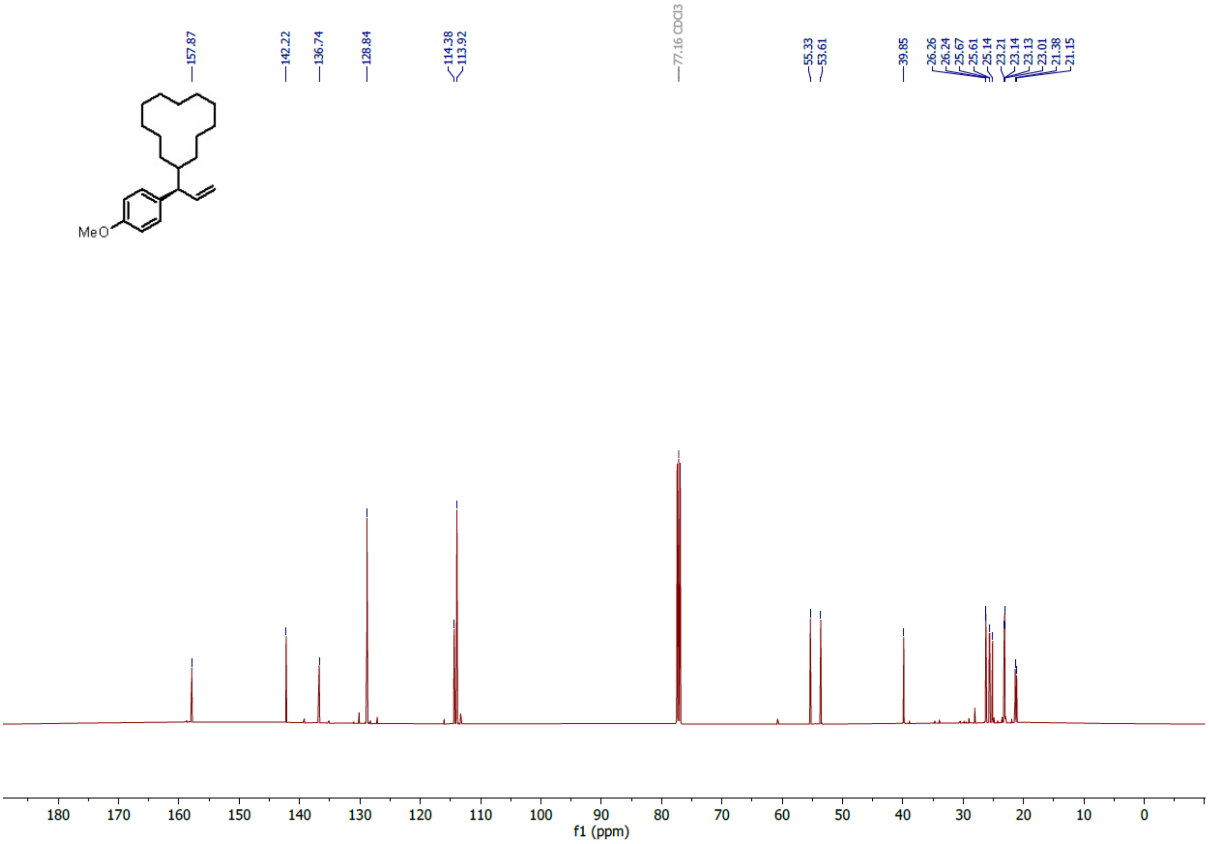

Compound **39'**

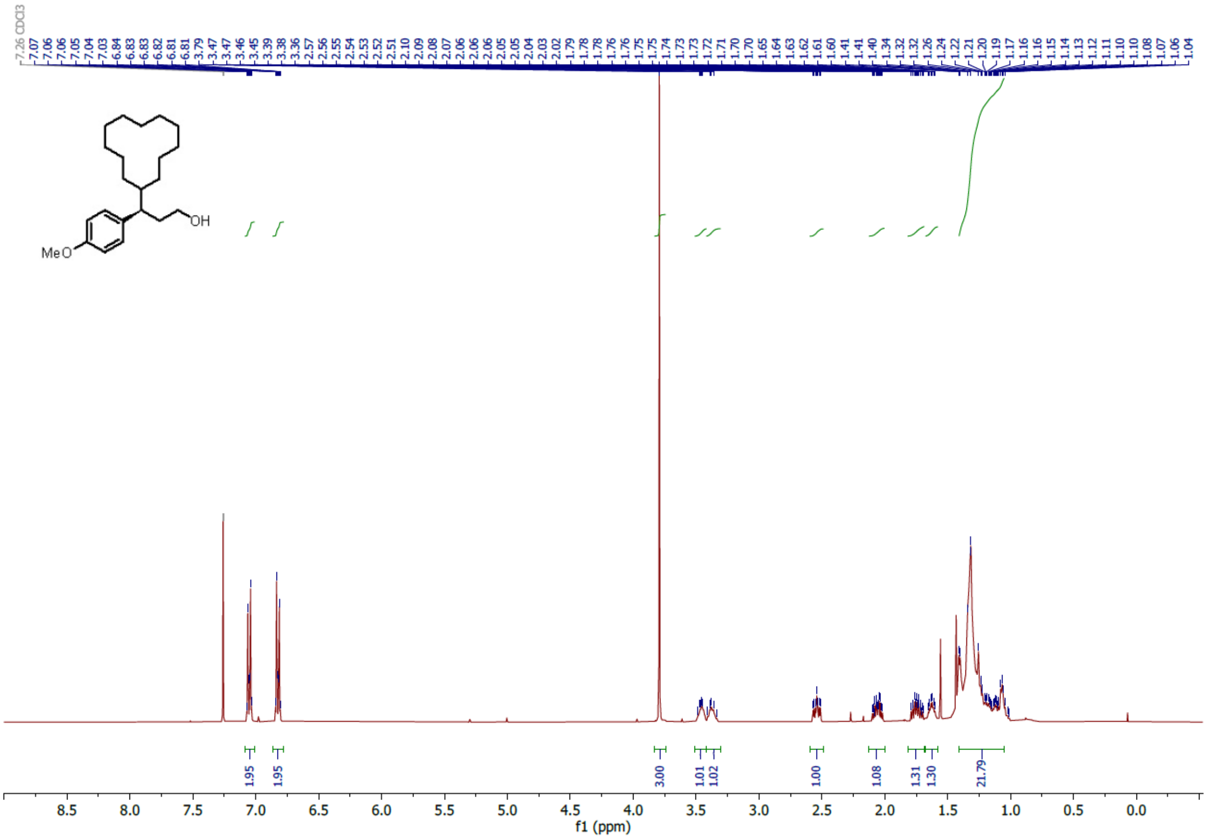

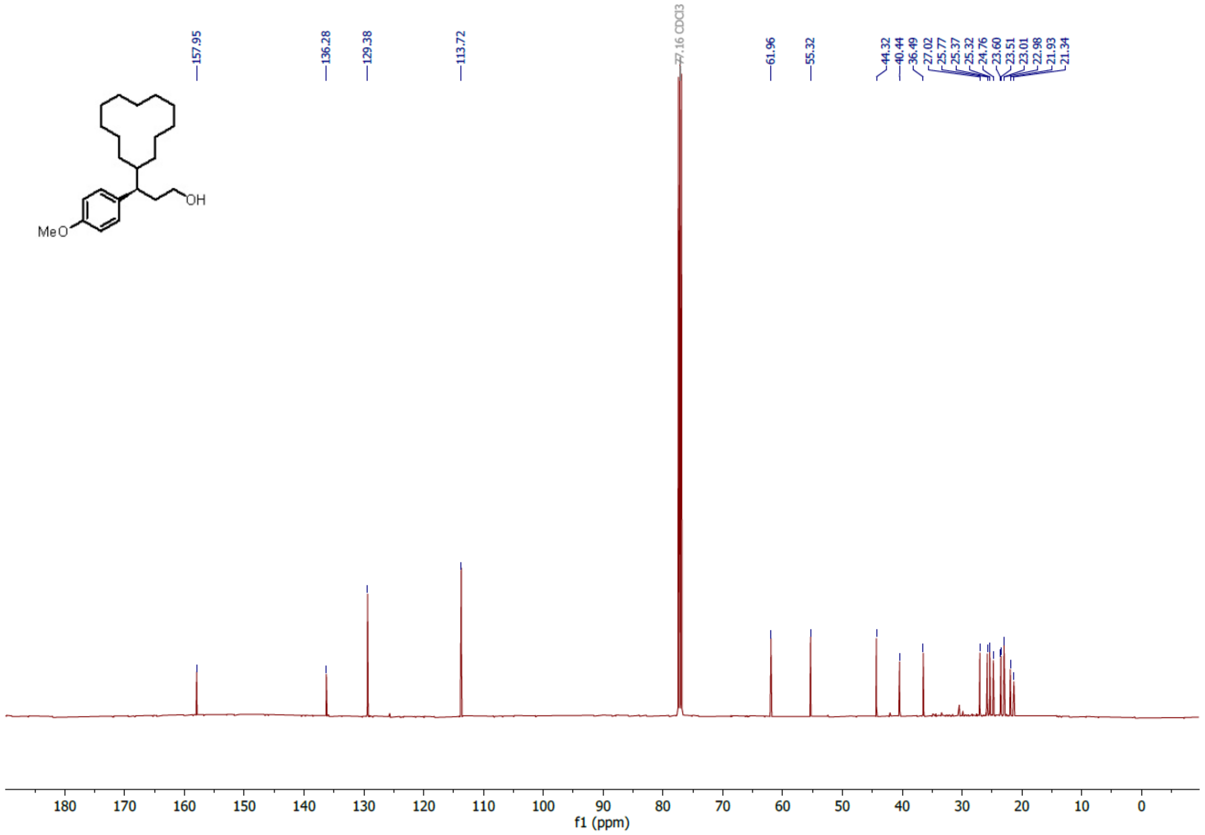

Compound **41**

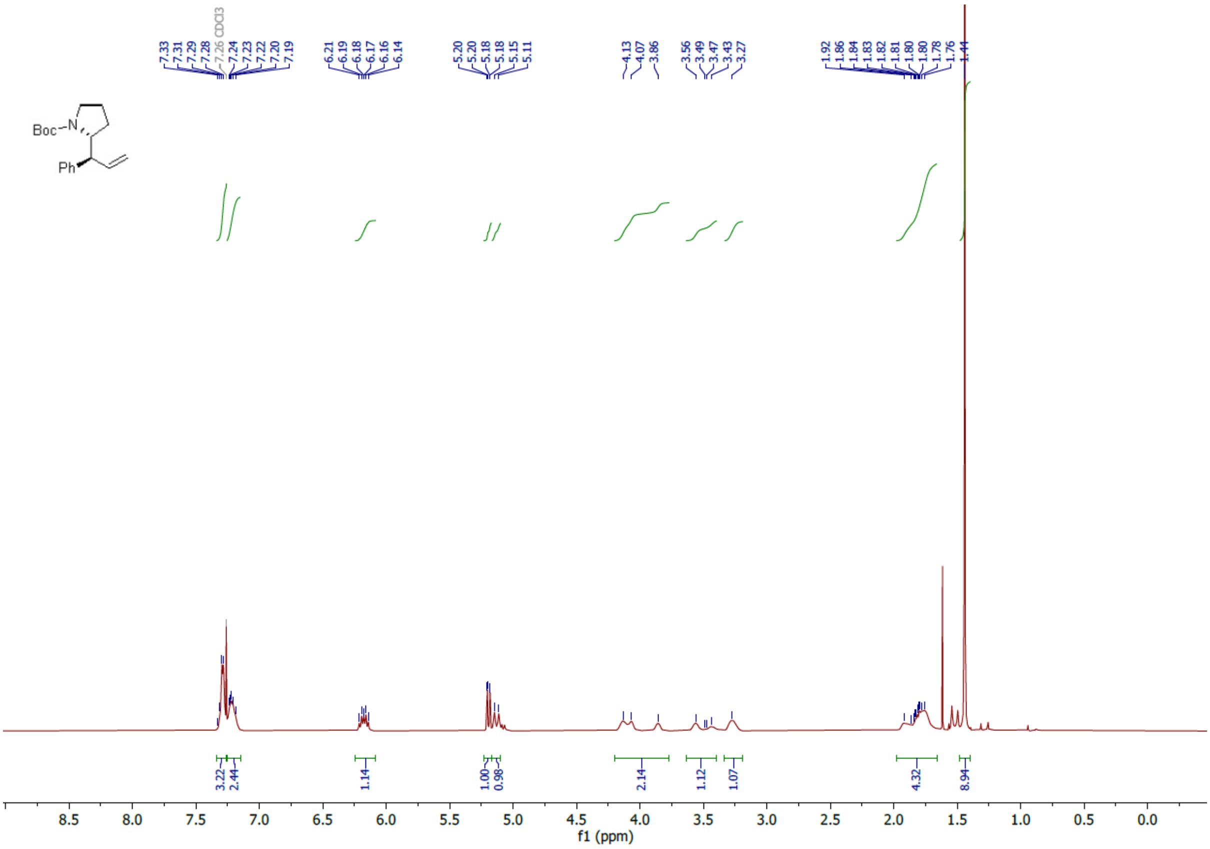

Boc–N
Ph
7.33
7.31
7.29
7.28
7.26 CDCl3
7.24
7.23
7.22
7.20
7.19
6.21
6.19
6.18
6.17
6.16
6.14
5.20
5.20
5.18
5.18
5.15
5.11
4.13
4.07
3.86
3.56
3.49
3.47
3.43
3.27
1.92
1.86
1.84
1.83
1.82
1.81
1.80
1.80
1.78
1.76
1.44
3.22
2.44
1.14
1.00
0.98
2.14
1.12
1.07
4.32
8.94
f1 (ppm)

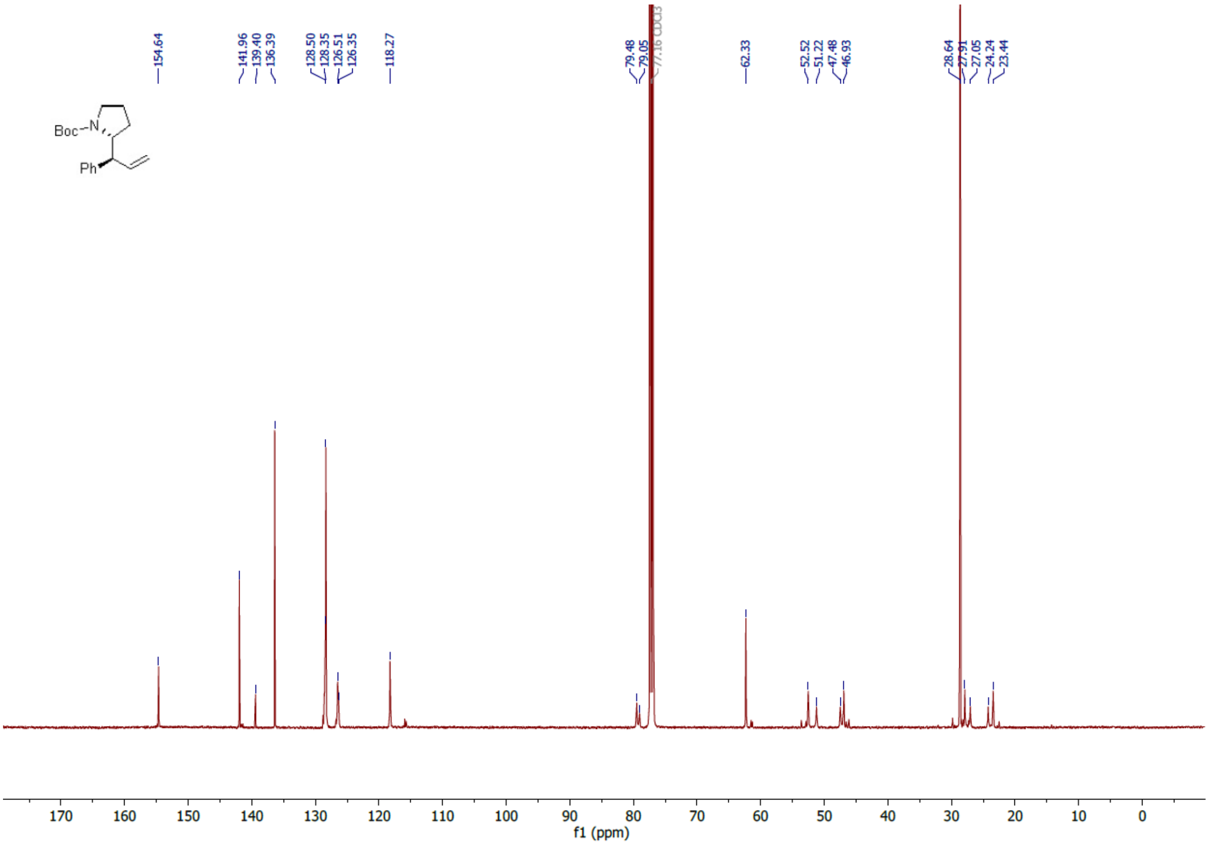

Boc–N
Ph
154.64
141.96
139.40
136.39
128.50
128.35
126.51
126.35
118.27
79.48
79.05
77.16 CDCl3
62.33
52.52
51.22
47.48
46.93
28.64
27.91
27.05
24.24
23.44
f1 (ppm)

Compound **42**

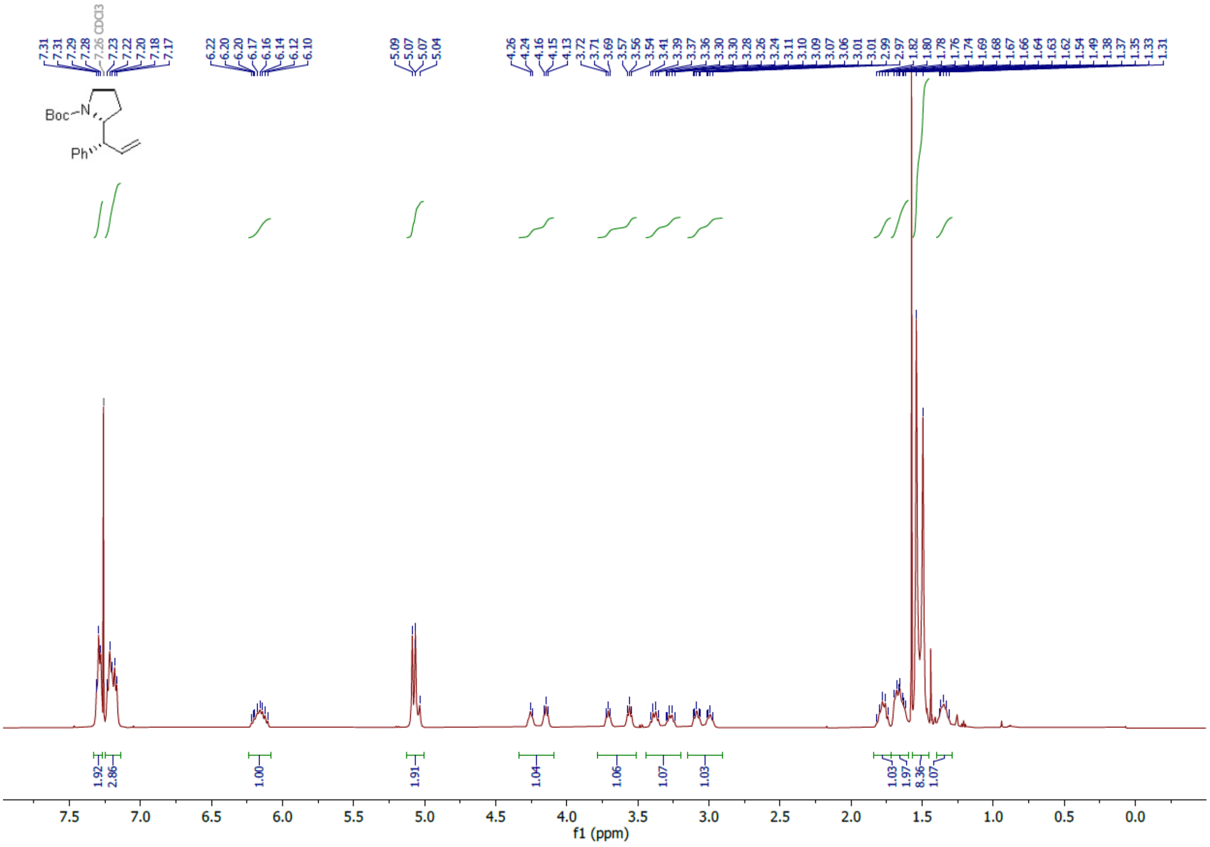

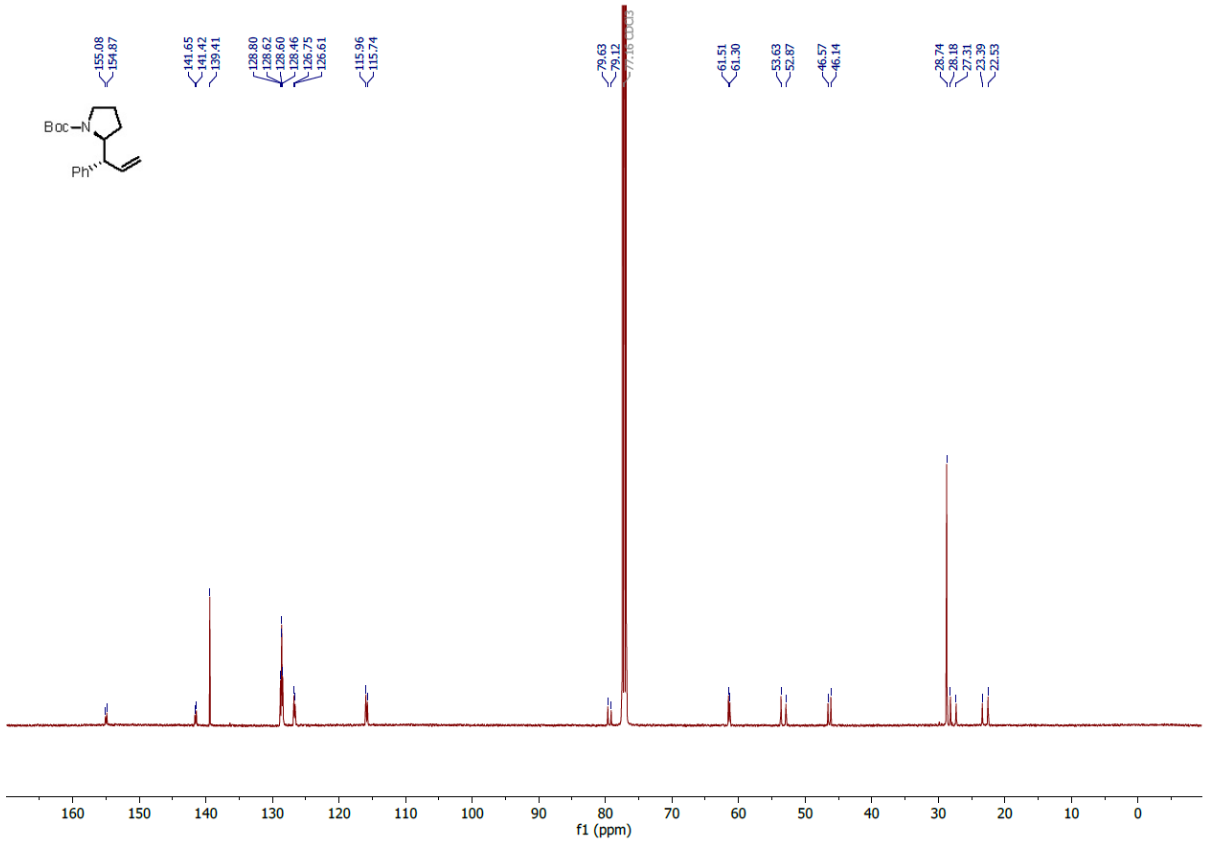

Compound **47**

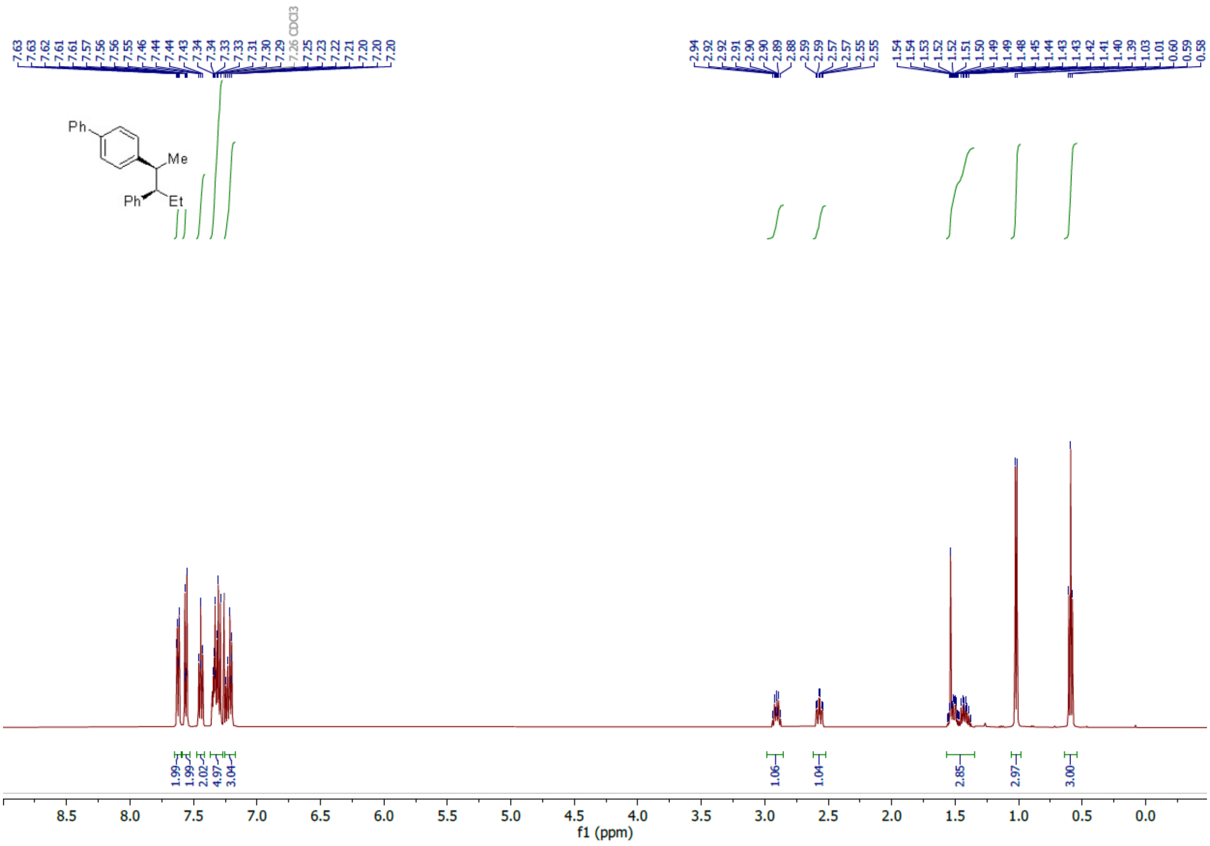

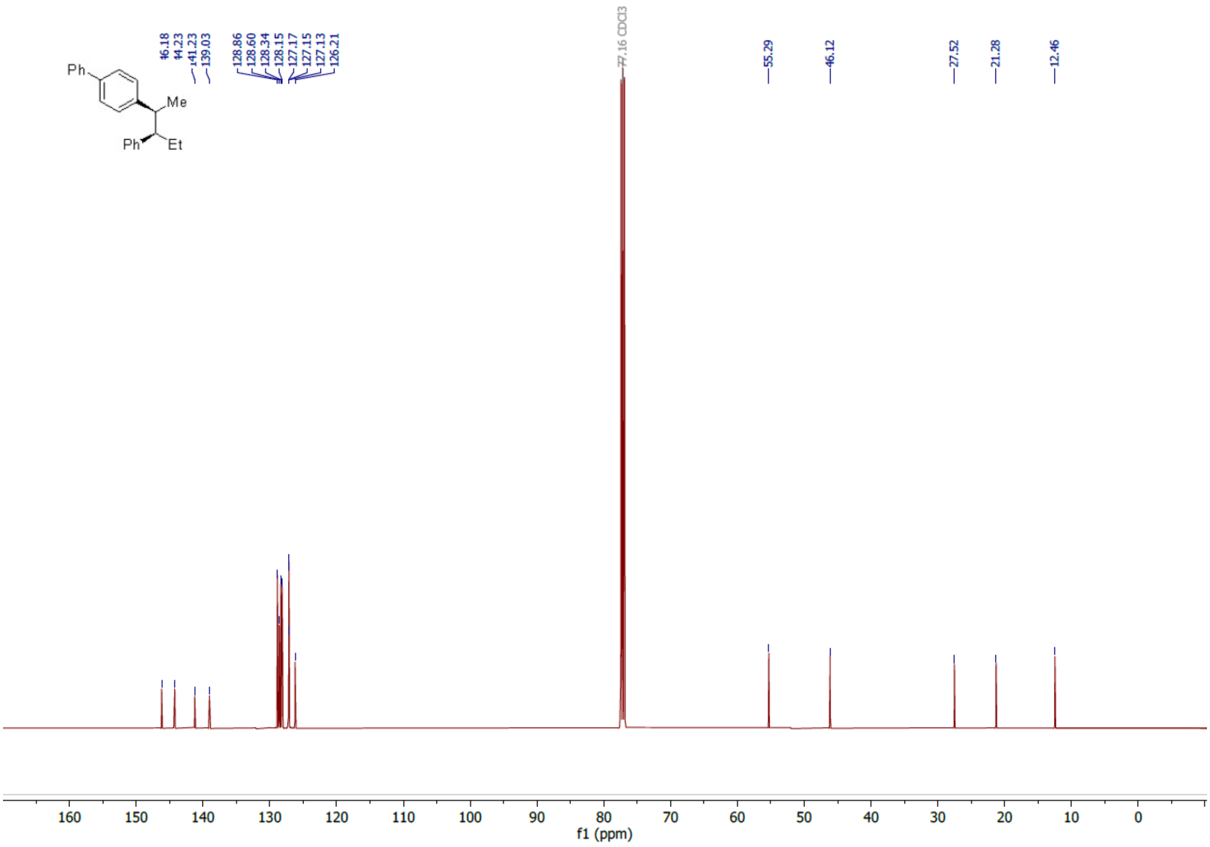

Compound **48**

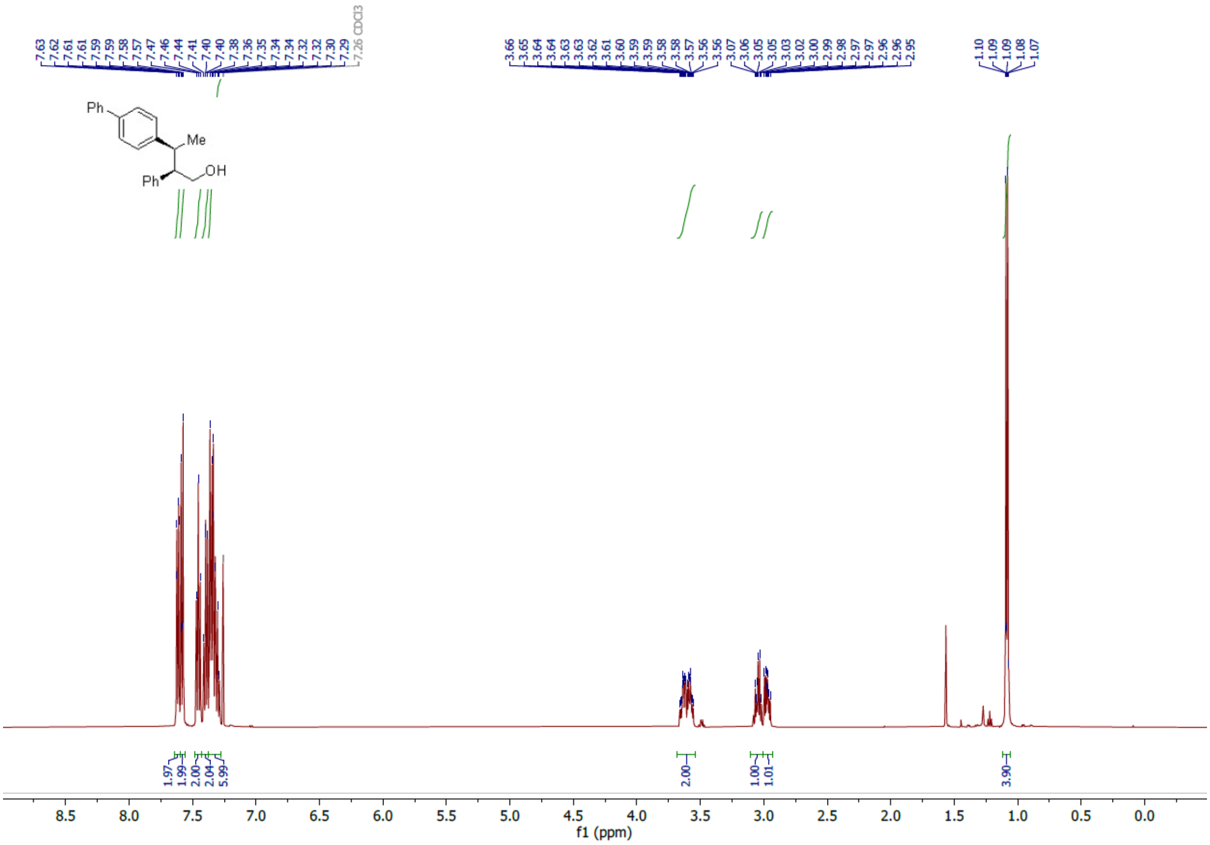
7.63 7.62 7.61 7.61 7.59 7.59 7.58 7.57 7.47 7.46 7.44 7.41 7.40 7.40 7.38 7.36 7.35 7.34 7.34 7.32 7.30 7.29 7.26 CDCl3
3.66 3.65 3.64 3.64 3.63 3.63 3.62 3.61 3.60 3.59 3.59 3.58 3.58 3.57 3.56 3.56 3.07 3.06 3.05 3.05 3.03 3.02 3.00 2.99 2.98 2.97 2.97 2.96 2.96 2.95
1.10 1.09 1.09 1.08 1.07
Ph
Me
Ph
OH
1.97 1.99 2.00 2.04 5.99
2.00
1.00 1.01
3.90
f1 (ppm)

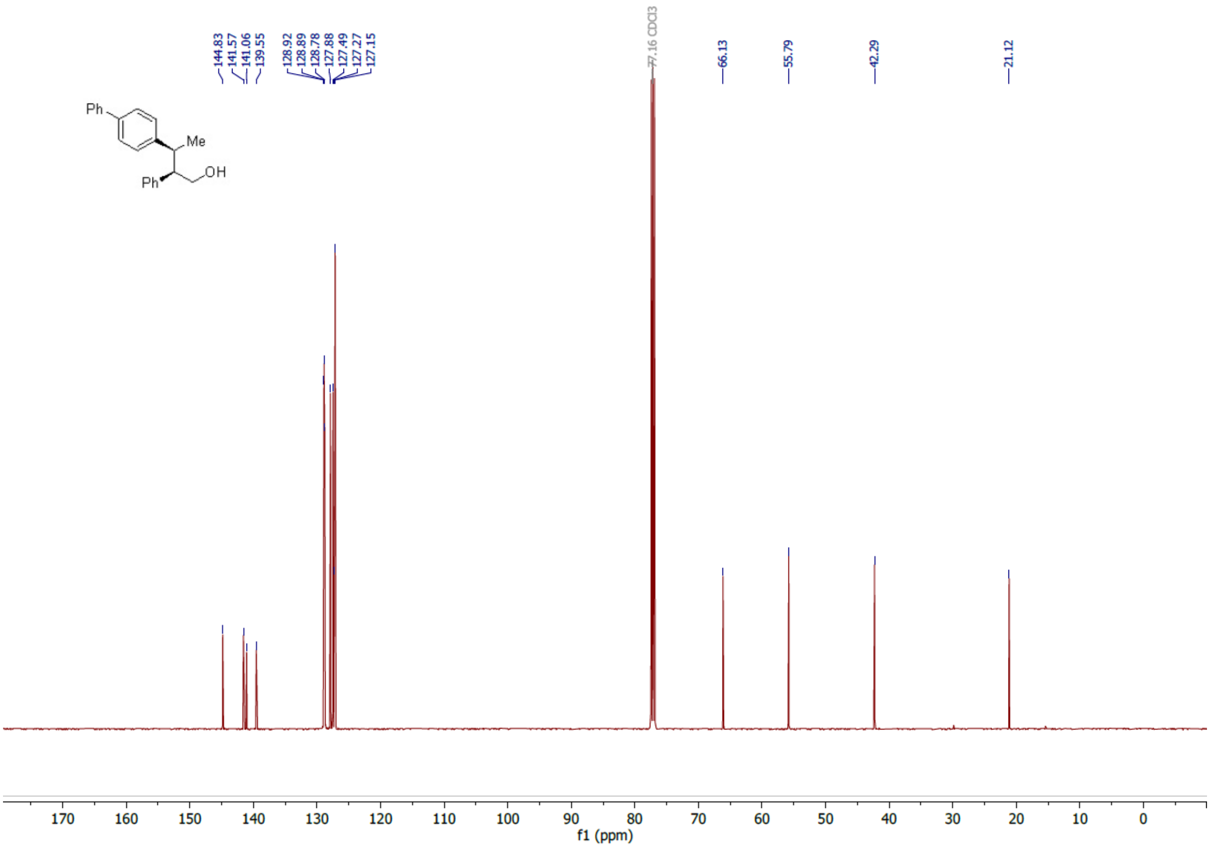
144.83 141.57 141.06 139.55
128.92 128.89 128.78 127.88 127.49 127.27 127.15
77.16 CDCl3
66.13
55.79
42.29
21.12
Ph
Me
Ph
OH
f1 (ppm)

Compound **49**

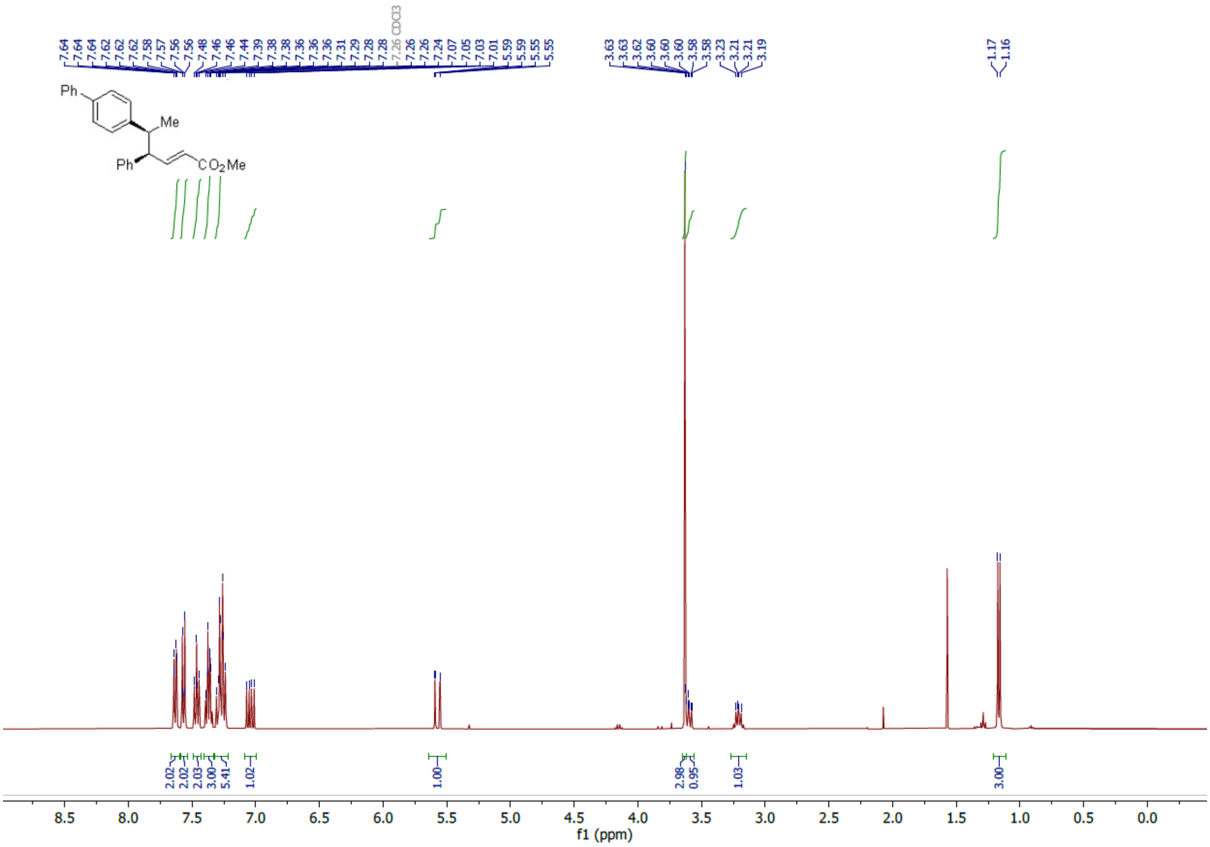

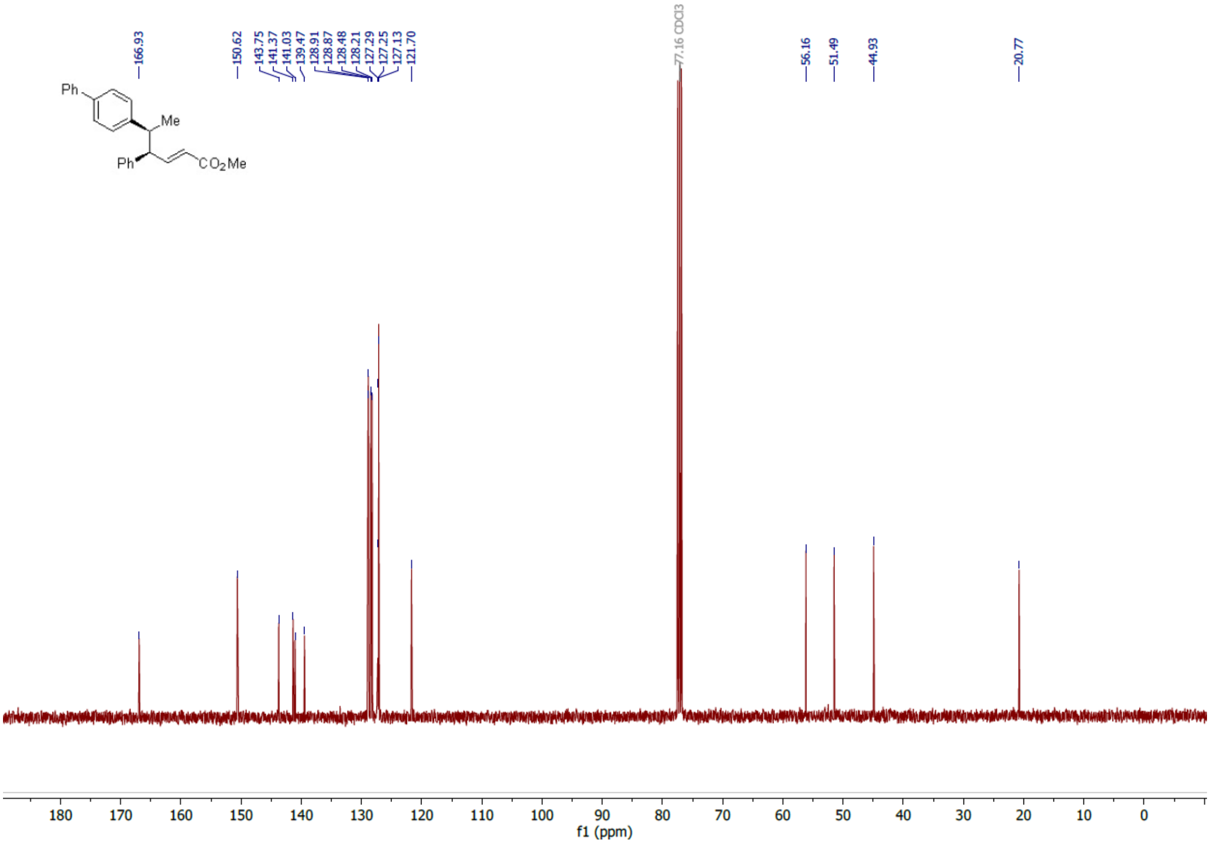

Compound **50**

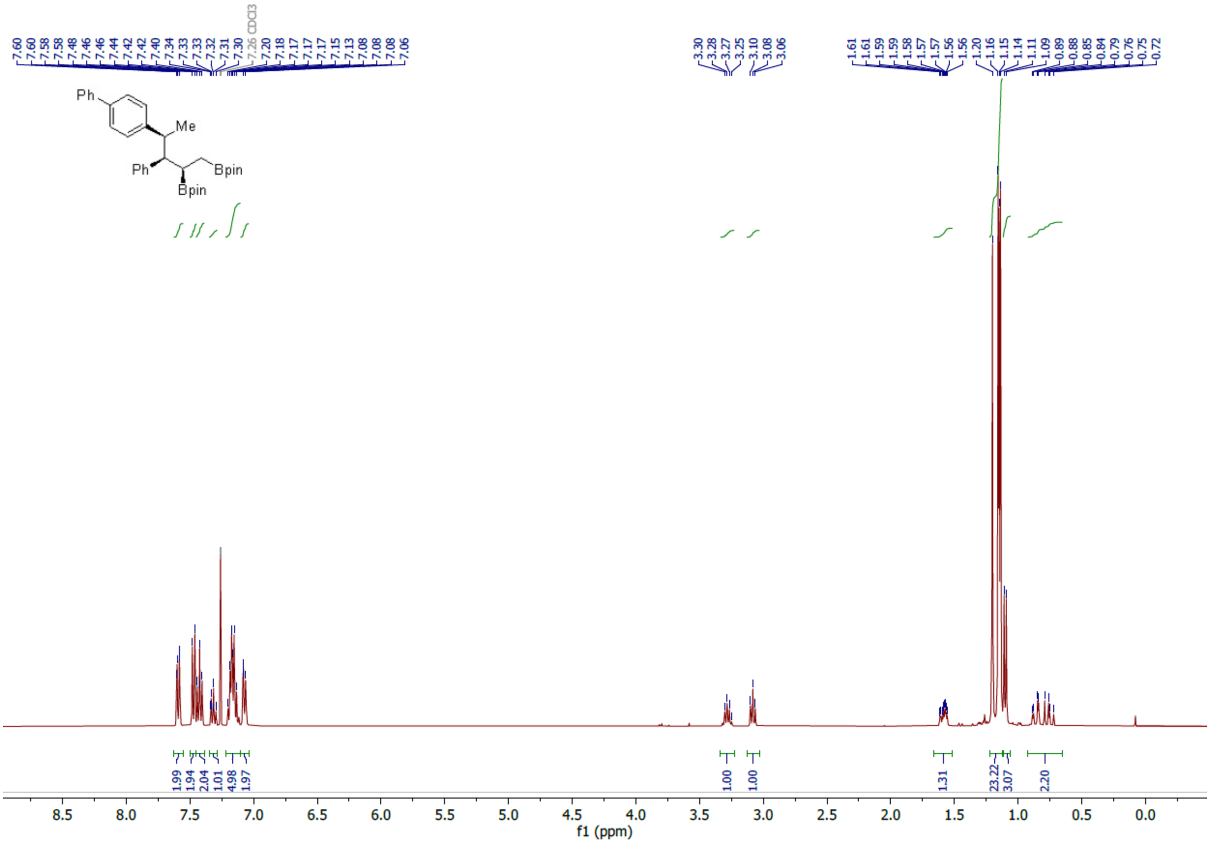

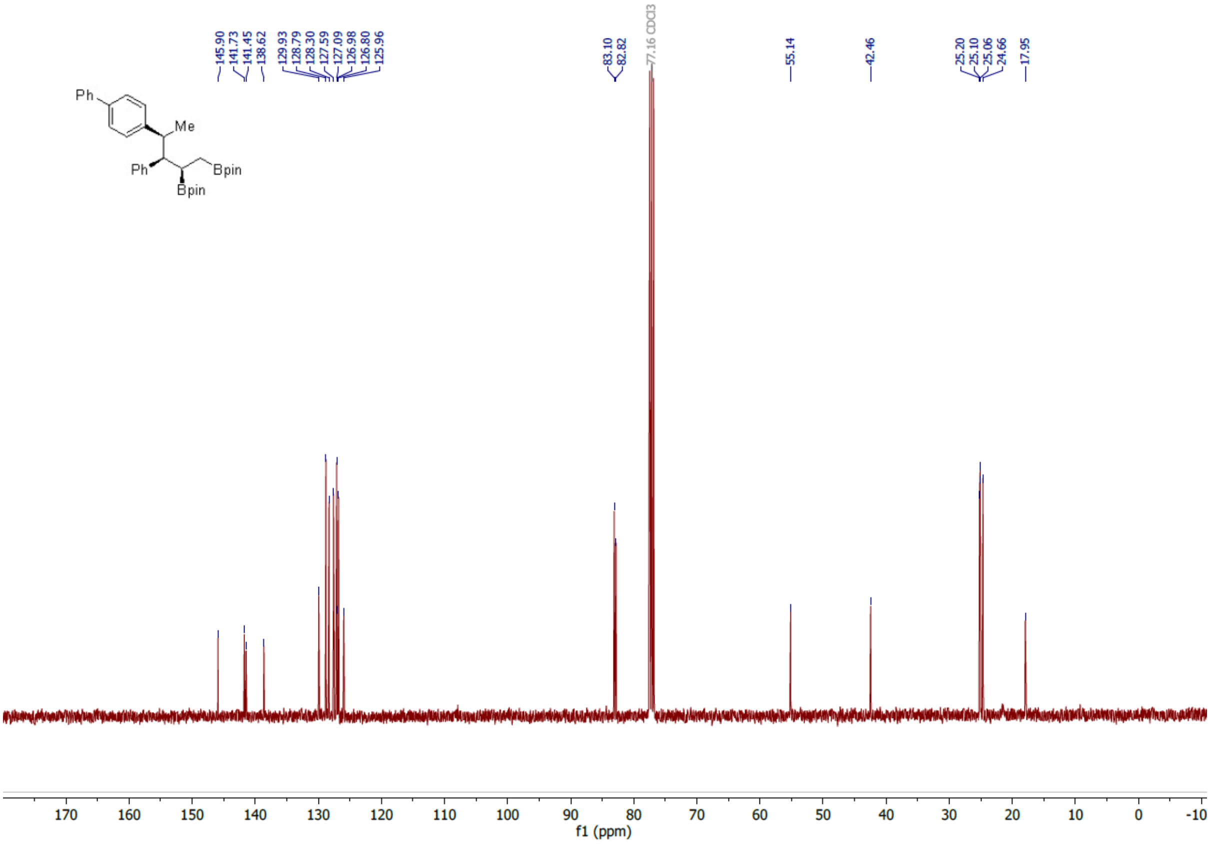

Compound **51**

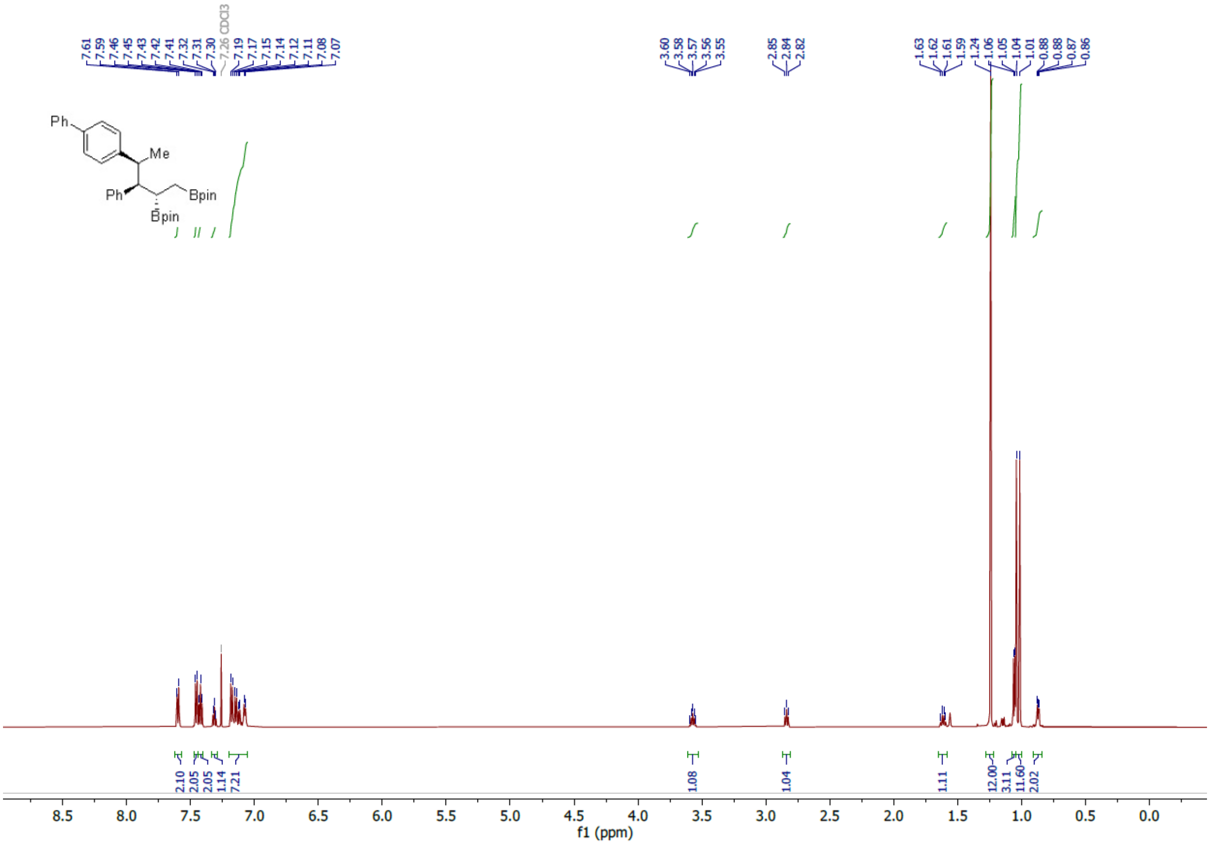

7.61
7.59
7.46
7.45
7.43
7.42
7.41
7.32
7.31
7.30
7.26 CDCl3
7.19
7.17
7.15
7.14
7.12
7.11
7.08
7.07
3.60
3.58
3.57
3.56
3.55
2.85
2.84
2.82
1.63
1.62
1.61
1.59
1.24
1.06
1.05
1.04
1.01
0.88
0.88
0.87
0.86
Ph
Me
Ph
Bpin
Bpin
2.10
2.05
2.05
1.14
7.21
1.08
1.04
1.11
12.00
3.11
11.60
2.02
f1 (ppm)

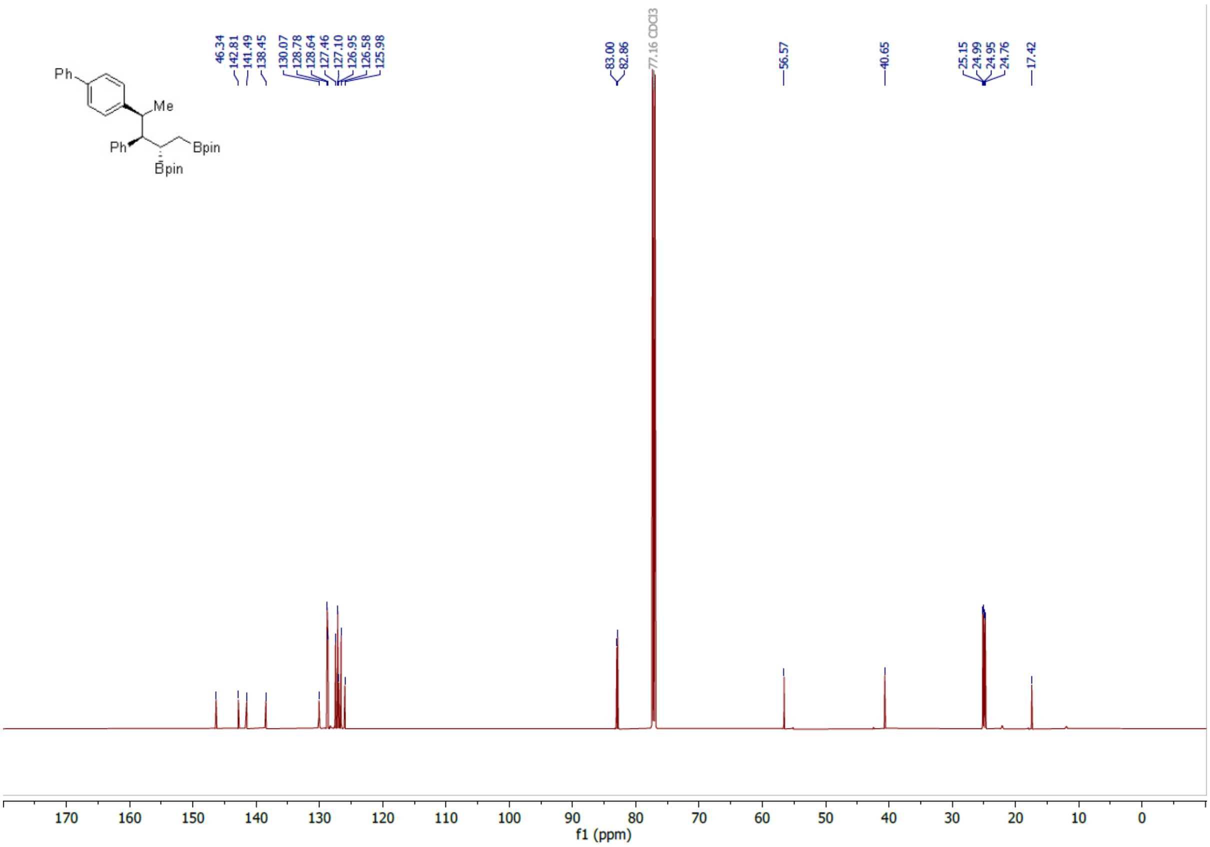

146.34
142.81
141.49
138.45
130.07
128.78
128.64
127.46
127.10
126.95
126.58
125.98
83.00
82.86
77.16 CDCl3
56.57
40.65
25.15
24.99
24.95
24.76
17.42
Ph
Me
Ph
Bpin
Bpin
f1 (ppm)

Compound **53**

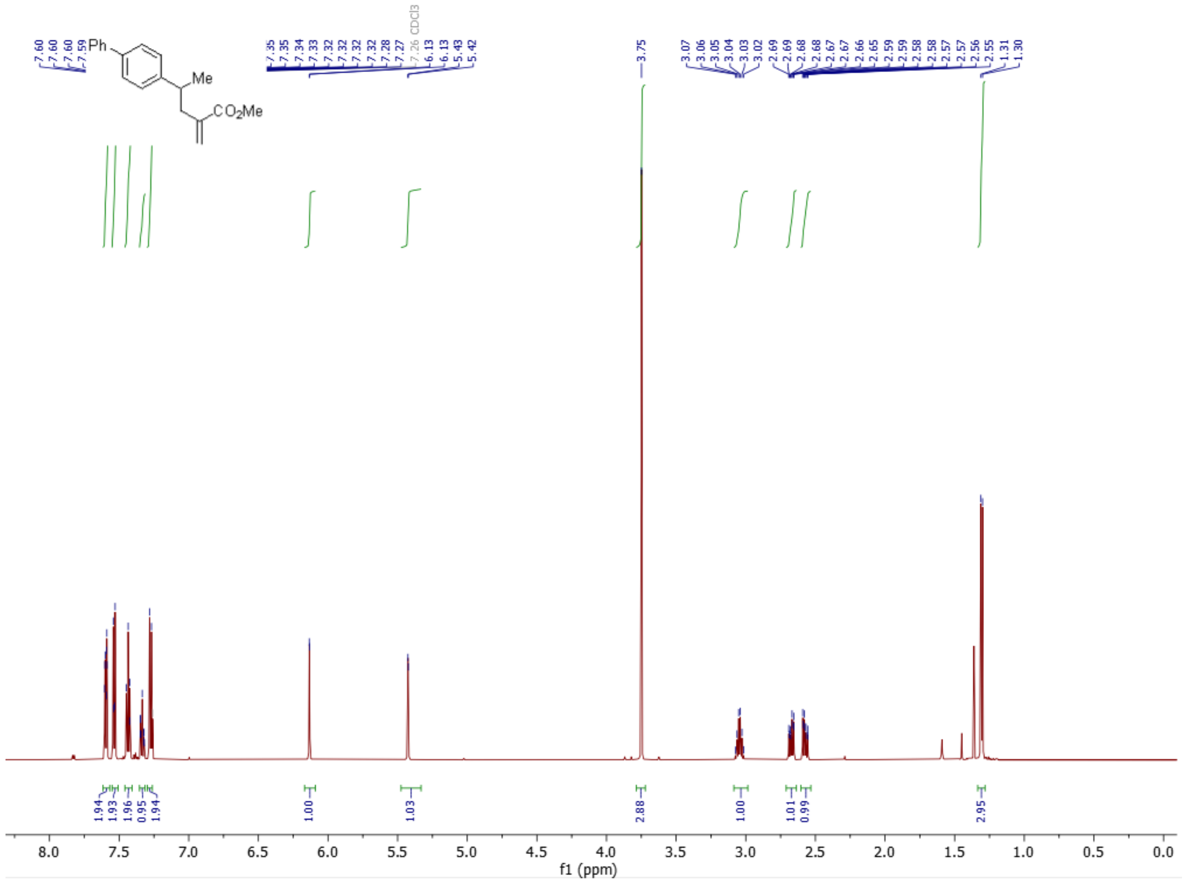

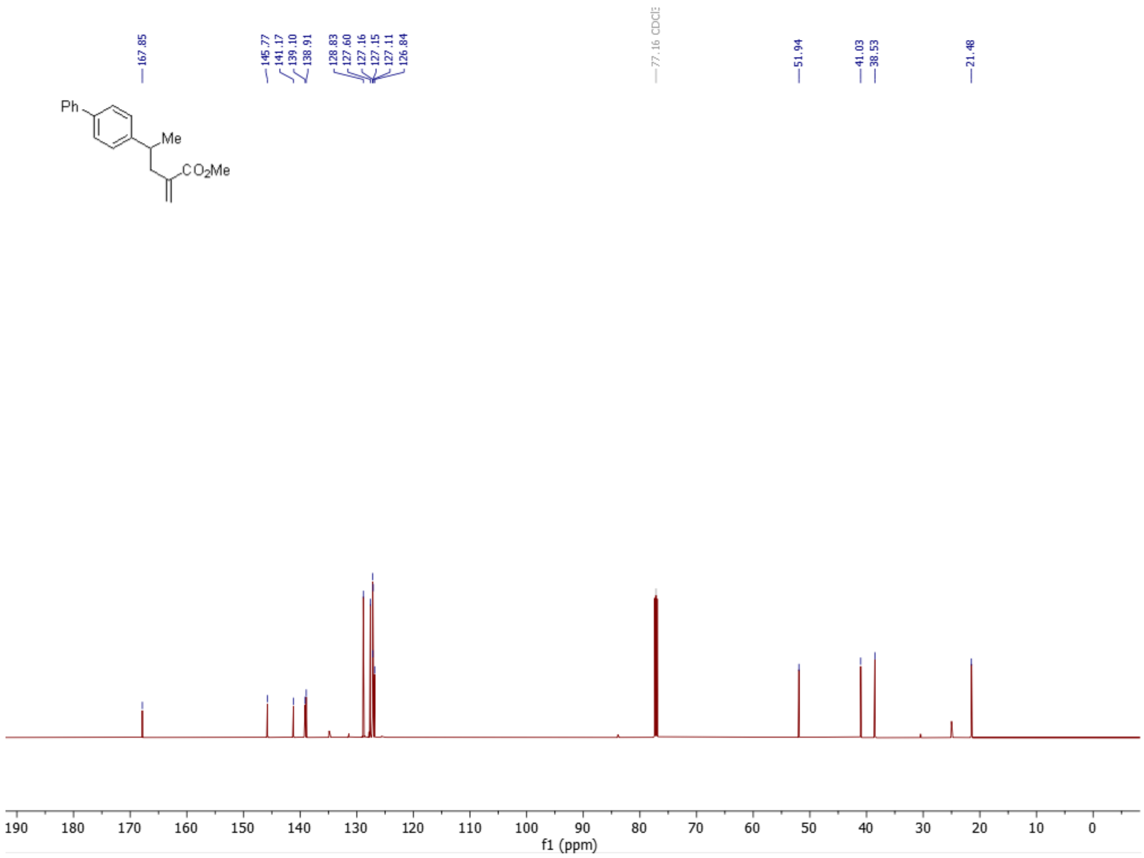

Supplement: Supplementary file 1 — ja4c03686_si_001.pdf [file ja4c03686_si_001.pdf]
